# Asymmetric Ketone Diene Coupling via Stereodivergent Copper Catalysis

**DOI:** 10.1021/jacs.5c06735

**Published:** 2026-02-02

**Authors:** Jiaming Liu, Zheye Zhang, Diego Troya, Ming Chen

**Affiliations:** † School of Chemistry & Materials, Jiangsu Provincial Key Laboratory of Green & Functional Materials and Environmental Chemistry, 38043 Yangzhou University, Yangzhou, Jiangsu 225000, China; ‡ Department of Chemistry, 1757 Virginia Tech, Blacksburg, Virginia 24061, United States

## Abstract

We report herein the stereodivergent and enantioselective coupling of ketones with dienes. Under the developed conditions, the reactions of 1,3-butadienyltriisopropylsilane with ketones provide *syn*-(*E*)-adducts with excellent enantioselectivities via an α-silyl-(*Z*)-allylcopper intermediate. By contrast, the reactions between 1,3-dienylboronate and ketones involve an α-boryl-(*E*)-allylcopper intermediate to give *anti*-(*Z*)-adducts with excellent optical purity. The stereodivergence and enantioselectivity of the reactions originate from the distinct allylcopper species involved in the ketone addition step. DFT computational studies provide further evidence to support the analyses.

## Graphical abstract

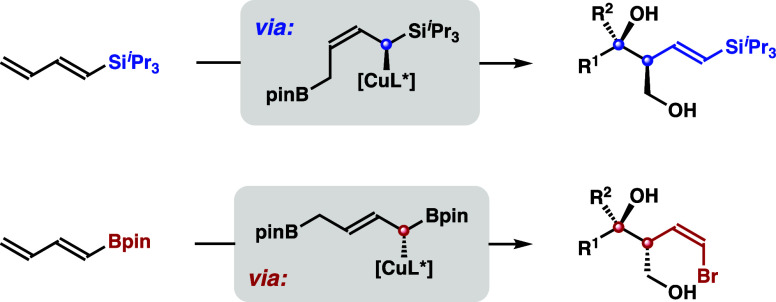

## Introduction

The development of chemical transformations with precise control of the stereochemical outcomes is an active research area in modern organic synthesis.^1^ For chiral molecules with more than one stereochemical element, however, it remains a significant challenge to develop methods that permit the access to different stereoisomers selectively. While asymmetric catalysis has been successful to produce one set of stereoisomers, generation of other stereoisomers with high selectivity is not a trivial task in many cases. Recently, stereodivergent catalysis has emerged as a promising strategy to address this problem.^2^ By subtle modifications of the catalytic systems, substrate structures, or reaction parameters, stereodivergent catalysis could allow for the production of various stereoisomers with high selectivity. For instance, through directed evolution of the enzymatic systems, stereodivergent biocatalysis has been successfully implemented to selectively form stereoisomers.^3^ By mimicking the active site of the enzymatic systems, small organic molecule catalysts have been utilized to attain the desired stereodivergence.^4^ In parallel, stereodivergent catalysis via transition metal complexes is also receiving significant attention.^5^ Owing to the ease of reaction parameter modification and the generally promiscuous nature of the reactions, transition metal-catalyzed stereodivergent synthesis has arguably become one of the most adopted approaches to modulate the stereochemical outcomes among all available strategies.

Enantioenriched tertiary homoallylic alcohols are valuable building blocks for chemical synthesis, natural product synthesis in particular.^6^ Over the past two decades, a significant amount of effort has been devoted to developing methods for asymmetric synthesis of these molecules.^7^ Conventional approaches to enantioenriched tertiary homoallylic alcohols rely on asymmetric ketone addition with stoichiometrically preformed allylmetal reagents. Recent studies in this research area, however, have emphasized the generation of reactive allylation species catalytically from readily available starting materials.^8^ As part of our research program in developing catalytic asymmetric transformations for organic synthesis, we became interested in the enantioselective preparation of tertiary homoallylic alcohols bearing two contiguous stereogenic centers and a stereodefined alkene group (e.g., **I** and **II** in Scheme 1). Inspired by recent development on Cu-catalyzed asymmetric functionalization of π-systems,^9–11^ we envisioned an enantioselective ketone diene coupling approach to synthesize the targeted tertiary homoallylic alcohols. However, several challenges associated with this approach are anticipated. As outlined in Scheme 1, the addition of copper complex [L*Cu-Bpin] to diene **A** should proceed via 1,2-addition to generate initial adduct **B** (or/and its enantiomer), which could equilibrate with copper complexes **C**–**E** through reversible 1,3-metalloshifts. Ketone addition with nucleophilic allylic copper species **B**–**E** could, in theory, form eight products. If the face selectivity of the [L*Cu-Bpin] addition to diene **A** is not perfect, the reaction could result in 16 possible products. At the outset, it is not apparent whether it is feasible to identify an appropriate catalytic system to control the regio-, diastereo-, and enantioselectivity as well as the alkene geometry of the reaction product. Despite these potential obstacles, we developed and report herein enantioselective and stereodivergent coupling of ketones with dienylsilane **1a** or dienylboronate **4** (Scheme 1). It is highly remarkable that the stereodivergence of the reaction originates from the distinct allylcopper species involved in the ketone addition step. The reactions with diene **1a** or **4** proceeded via copper intermediate, **E** or **C**, respectively, to form *syn*-*E*-adduct **I** or *anti*-*Z*-adduct **II** selectively. DFT studies provide further evidence to support these analyses. Products **I** and **II** contain a functionalized alkene unit and an alkylBpin group, providing useful handles for further derivatization.^12^

**Scheme 1:**
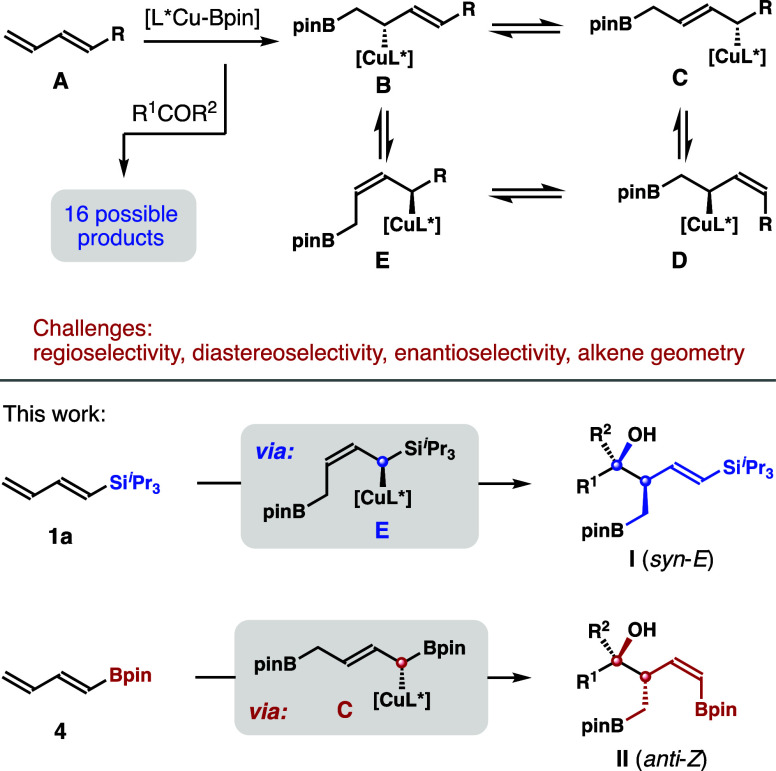
Proposed Asymmetric Ketone Diene Coupling

## Results and Discussion

### Reaction Development for Asymmetric Ketone Dienylsilane Coupling

We began our studies by identifying a proper catalytic system to couple diene **1** with acetophenone. In initial experiments, CuOAc, (*R*,*R*)-*^i^* Pr-DuPhos, and NaO*^t^* Bu were identified as the optimal catalyst, ligand, and base, respectively (See the SI for details). Studies on the impact of the silyl group were conducted next, and the results are summarized in Table 1. In the presence of 10 mol % CuOAc, 12 mol % (*R*,*R*)-*^i^* Pr-DuPhos, 1.5 equiv of NaO*^t^* Bu and B_2_pin_2_, the reaction of *^i^* Pr_3_Si-substituted diene (**1a**) with acetophenone occurred at ambient temperature in Et_2_O. Subsequent oxidative workup gave *Z*-adduct **2** in 84% yield with 91% ee, 14:1 *E*-selectivity, and >20:1 diastereoselectivity (entry 1). Similar results were achieved for the Ph_3_Si-substituted diene (**1b**) with slightly diminished *E*-selectivity and yield (10:1, 69%, entry 2). The reaction with *^t^* BuMe_2_Si-substituted diene (**1c**) proceeded with excellent *E*-selectivity and diastereoselectivity, although the enantioselectivity decreased substantially (63% ee, entry 3). Poor *E*-selectivities were observed in the reactions with dienes **1d**–**g** (4:1 to 1:1, entries 4–7). We also evaluated whether the solvent has any effect on the selectivities of the reactions (entries 8–12). Among the solvents we surveyed, the highest enantioselectivities were achieved with THF and 1,4-dioxane (96% ee and 94% ee, entries 8 and 10). After considering the balance of the yield, diastereoselectivity, and optical purity, we decided to use 1,4-dioxane as the solvent to explore the reaction scope with *^i^* Pr_3_Si-substituted diene **1a**.

**Table 1:**
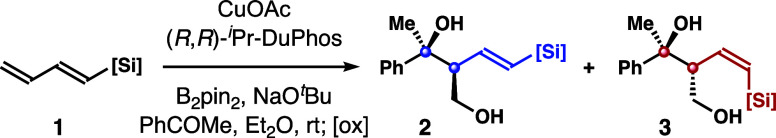
Evaluation of Reaction Conditions with Diene **1** ^a^ ^‑^ ^e^

entry	[Si]	solvent	*E*:*Z*(**2:3**)	dr (**2**)	yield (%)	ee (**2**) (%)
1	Si*^i^* Pr_3_ (**1a**)	Et_2_O	14:1	>20:1	84	91
2	Si*^i^* Ph_3_ (**1b**)	Et_2_O	10:1	>20:1	69	91
3	SiMe_2_ *^t^* Bu (**1c**)	Et_2_O	>20:1	>20:1	82	63
4	SiPh_2_Me (**1d**)	Et_2_O	4:1	>20:1	69	ND
5	SiEt_3_ (**1e**)	Et_2_O	2:1	9:1	62	ND
6	SiMe_2_Ph (**1f**)	Et_2_O	2:1	>20:1	86	ND
7	SiMe_2_Bn (**1g**)	Et_2_O	1:1	6:1	76	ND
8	Si*^i^* Pr_3_ (**1a**)	THF	10:1	>20:1	58	96
9	Si*^i^* Pr_3_ (**1a**)	MeO*^t^* Bu	10:1	>20:1	32	89
10	Si*^i^* Pr_3_ (**1a**)	1,4-dioxane	14:1	>20:1	69	94
11	Si*^i^* Pr_3_ (**1a**)	toluene	12:1	>20:1	75	83
12	Si*^i^* Pr_3_ (**1a**)	cyclohexane	4:1	>20:1	40	88

a Reaction conditions: dienylsilane **1** (0.1 mmol, 1.0 equiv), CuOAc (10 mol %), (*R*,*R*)-*^i^* Pr-DuPhos (12 mol %), B_2_pin_2_ (1.5 equiv), NaO*^t^* Bu (1.5 equiv), PhCOMe (1.5 equiv), Et_2_O (1.5 mL), rt; NaOH, H_2_O_2_, 0 °C.

b The *E*/*Z*-selectivities and diastereoselectivities were determined by ^1^H NMR analyses of crude reaction products.

c Yields of isolated combined products **2** and **3** are listed.

d Enantiopurities of **2** were determined by HPLC analysis using a chiral stationary phase.

e ND: not detected.

### Scope of *E*-Selective Asymmetric Dienylsilane Ketone Coupling

Table 2 summarizes the scope of ketones that underwent enantioselective coupling with diene **1a**. In the presence of 10 mol % CuOAc, 12 mol % (*R*,*R*)-*^i^* Pr-DuPhos, 1.5 equiv of NaO*^t^* Bu and B_2_pin_2_, the reaction occurred smoothly with a wide range of ketones. Subsequent oxidative workup gave diols **2a**–**o** with high *E*-selectivity and enantioselectivities. In all cases, the diastereoselectivities are uniformly excellent (>20:1). For example, the reaction with an aromatic ketone bearing a methyl group at the *para*-position of the arene provided diol **2b** in 77% yield with 17:1 *E*-selectivity and 93% ee. When the methyl group is replaced by a more electron-donating substituent, a methoxy group, diol **2c** was generated in 77% yield with 18:1 *E*-selectivity and 94% ee. Diminished *E*-selectivities (8:1) were observed in the reactions with aromatic ketones bearing a halogen atom (Cl, Br) at either the *para*- or *meta*-position. In these cases, diols **2d**–**f** were isolated in 66–73% yields with 90–95% ee. The reactions of aromatic ketones with other substitution patterns gave products **2g**–**h** in 79–86% yields with 10:1 to 20:1 *E*-selectivities and 91–95% ee. Aromatic ketones containing a heterocycle such as thiophene, benzothiophene, or benzofuran are also suitable substrates for the reactions, and diols **2i**–**l** were obtained in 65–84% yields with good *E*-selectivities and 90–95% ee. The reaction with β-naphthyl methyl ketone afforded diol **2m** in 80% yield with 17:1 *E*-selectivity and 94% ee. The alkyl group of aromatic ketones is not limited to a methyl group. The reaction with propiophenone furnished diol **2n** in 72% yield with >20:1 *E*-selectivity and 90% ee. The reaction with butyrophenone also occurred to give diol **2o** with >20:1 *E*-selectivity, albeit with diminished enantioselectivity and yield (85% ee, 56%). Poor results were obtained with aromatic ketones bearing a large alkyl group, cyclohexyl phenyl ketone, for instance. However, after a brief survey of the ligand, we discovered that, by replacing the (*R*,*R*)-*^i^* Pr-DuPhos ligand with (*R*,*R*)-Ph-BPE, the reaction produced diol **2p** in 60% yield and 96% ee with >20:1 *E*-selectivity. Similar results were achieved with symmetrical ketones, including aliphatic ketones. With (*R*,*R*)-Ph-BPE as the ligand, a variety of symmetrical ketones participated in the reactions with diene **1a** to afford diols **2q**–**v** in moderate to excellent yields with >20:1 *E*-selectivities and 94–99% ee. It should be noted that the reactivities of symmetrical acyclic aliphatic ketones under the standard conditions are low. However, complete conversion was achieved when the reactions were conducted at an elevated temperature (60 °C). For example, diol **2w** was obtained from 3-pentanone in 54% yield with >20:1 *E*-selectivities and 92% ee. In the case of unsymmetrical acyclic aliphatic ketone **2x**, the diastereoselectivity is poor (1:1). Decreasing the loadings to 5 mol % of CuOAc and 6 mol % of the ligand did not impact the reaction selectivities. The relative configuration of diols **2** was assigned as *syn* (the tertiary hydroxyl group is *syn* to the hydroxymethyl group) by NOE analyses of the acetonide derivatives. The structural assignment and absolute configuration of diols **2** were further corroborated by X-ray crystallography analyses (Table 2).

**Table 2:**
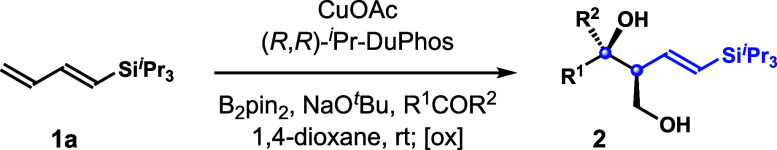

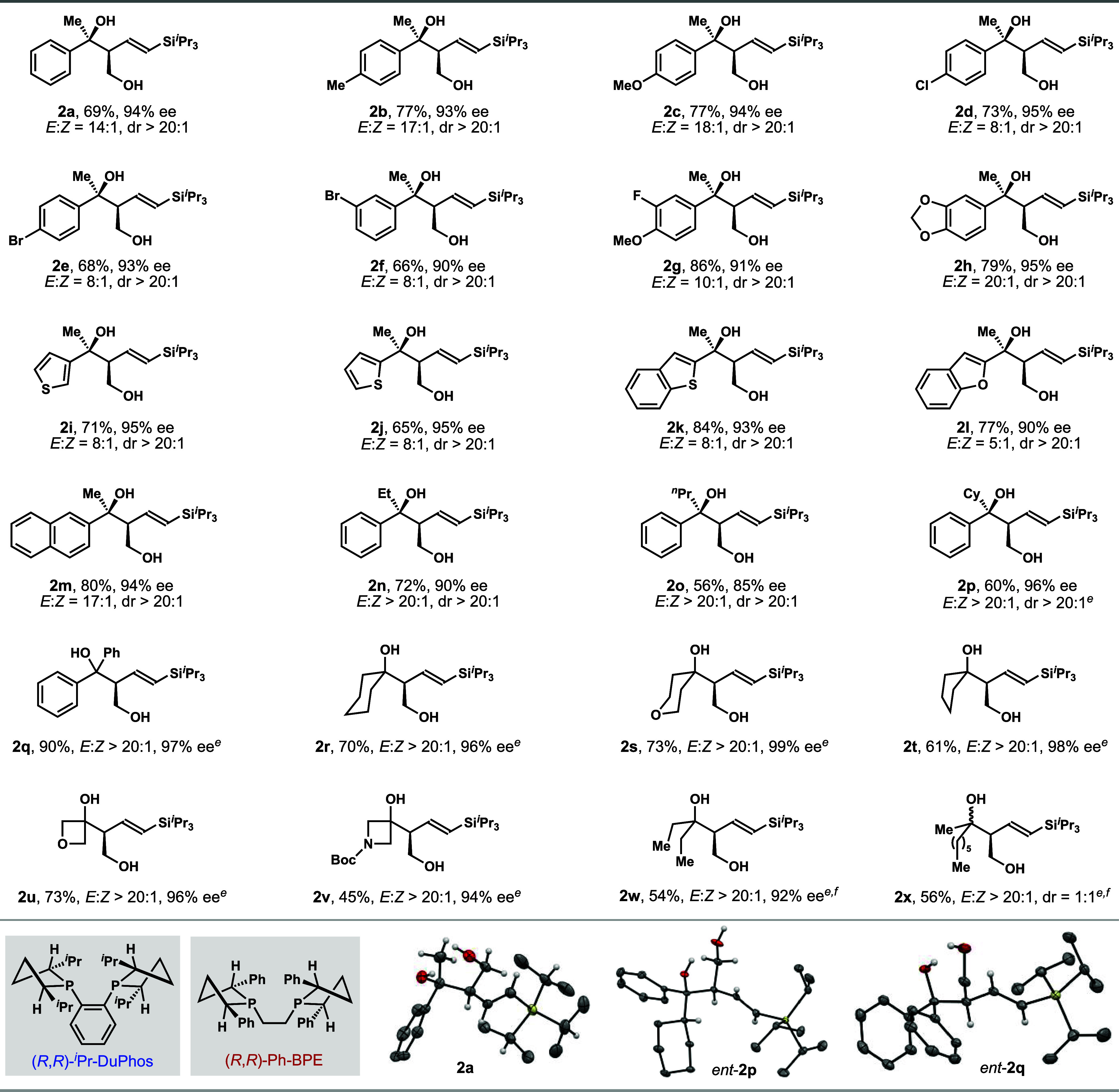
Scope of Asymmetric Coupling of Ketones with Dienylsilane **1a** ^a^ ^‑^ ^g^

a Reaction conditions: dienylsilane **1a** (0.1 mmol, 1.0 equiv), CuOAc (10 mol %), (*R*,*R*)-*^i^* Pr-DuPhos (12 mol %), B_2_pin_2_ (1.5 equiv), NaO*^t^* Bu (1.5 equiv), R^1^COR^2^ (1.5 equiv), 1,4-dioxane (1.5 mL), rt; NaOH, H_2_O_2_, THF-H_2_O, 0 °C. Reactions of several substrates with 5 mol % catalysts and 6 mol % ligands gave products **2** with similar levels of yields, *E*-selectivities, diastereo- and enantioselectivities.

b Diastereoselectivities and *Z*/*E*-selectivities were determined by ^1^H NMR analyses of the crude reaction mixtures.

c Yields of isolated products are listed.

d Enantiomeric excesses of **2** were determined by HPLC analysis using a chiral stationary phase.

e (*R*,*R*)-Ph-BPE (12 mol %) was used as the ligand.

f The reaction was conducted at 60 °C.

g (*S*,*S*)-Ph-BPE was used for the preparation of *ent*-**2p** and *ent*-**2q** for X-ray analyses.

### Reaction Development for Asymmetric Dienylboronate Ketone Coupling

Next, we were intrigued to learn whether the asymmetric diene ketone coupling procedure could be extended to dienylboronate **4**. As shown in Table 3, the reactions of 4-methylacetophenone with diene **4** were conducted to map out optimal conditions. The products were converted into diols **5b** and **6b** through sequential bromination and oxidation for ease of product isolation. Initial studies on ligand optimization revealed that reactions with axially chiral ligands, including (*R*)-BINAP and (*R*)-Segphos, and P-chiral ligands, such as DuanPhos and Quinox P, produced *E*-isomers **6b** as a mixture of *syn* and *anti* diastereomers with poor selectivity and moderate *E*-selectivity (entries 1–4). By contrast, when (*R*,*R*)-Ph-BPE was employed as the ligand, *Z*-isomer **5b** was obtained in >20:1 diastereoselectivity, 7:1 *Z*-selectivity, and 98% ee (entry 5). A lower *Z*-selectivity (4:1) was observed when (*R*,*R*)-*^i^* Pr-DuPhos was utilized (entry 6). Similar selectivity trends were observed when the reactions were conducted with acetophenone. A brief survey of the reaction medium revealed that Et_2_O is the optimal solvent (entries 7–10). Finally, the *Z*-selectivity was significantly improved when the reaction was conducted with KO*^t^* Bu as the base, affording product **5b** in 77% yield, >20:1 diastereoselectivity, 15:1 *Z*-selectivity, and 98% ee (entry 11). Reaction with (*R*,*R*)-*^i^* Pr-DuPhos as the ligand and KO*^t^* Bu as the base failed to improve the *Z*-selectivity (entry 12). In all cases, no product derived from copper species **B** or **D** (Scheme 1) was observed.

**Table 3:**
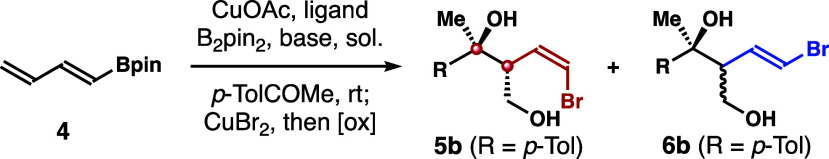
Evaluation of Reaction Conditions with Diene **4** ^b^ ^‑^ [Table-fn t3fn6]

entry	ligand	solvent	base	*Z*:*E* (**5:6**)	yield (%)	ee (**5**) (%)
1	**L**_**1**_	Et_2_O	NaO*^t^* Bu	1:4	67	ND
2	**L**_**2**_	Et_2_O	NaO*^t^* Bu	1:3	60	ND
3	**L**_**3**_	Et_2_O	NaO*^t^* Bu	1:4	11	ND
4	**L**_**4**_	Et_2_O	NaO*^t^* Bu	1:6	77	ND
5	**L**_**5**_	Et_2_O	NaO*^t^* Bu	7:1	70	98
6	**L**_**6**_	Et_2_O	NaO*^t^* Bu	4:1	74	98
7	**L**_**5**_	THF	NaO*^t^* Bu	6:1	84	98
8	**L**_**5**_	1,4-dioxane	NaO*^t^* Bu	6:1	88	98
9	**L**_**5**_	toluene	NaO*^t^* Bu	6:1	28	96
10	**L**_**5**_	hexane	NaO*^t^* Bu	6:1	56	97
11	**L**_**5**_	Et_2_O	KO*^t^* Bu	15:1	81	98
12	**L**_**6**_	Et_2_O	KO*^t^* Bu	5:1	77	98

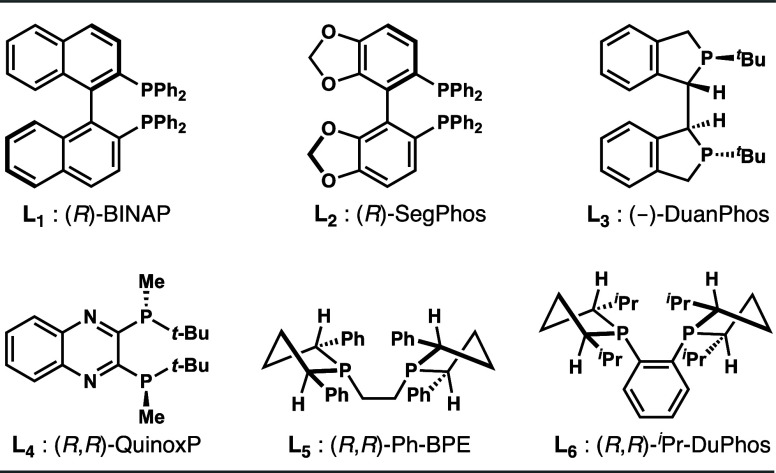

a Reaction conditions: dienylboronate **4** (0.1 mmol, 1.0 equiv), CuOAc (10 mol %), ligand (12 mol %), B_2_pin_2_ (1.5 equiv), base (1.5 equiv), 4-Me-PhCOMe (1.5 equiv), solvent (1.5 mL), rt; CuBr_2_ (3.0 equiv), THF-H_2_O, 70 °C; NaOH, H_2_O_2_, THF-H_2_O, 0 °C.

b The *E*/*Z*-selectivities and diastereoselectivities were determined by ^1^H NMR analyses of crude reaction products.

c Yields of isolated combined products **5b** and **6b** are listed.

d Enantiopurities of **5b** were determined by HPLC analysis.

e ND: not detected.

### Scope of *Z*-Selective Asymmetric Dienylboronate Ketone Coupling

The scope of the ketone that participated in the reactions with dienylboronate **4** was explored next. As shown in Table 4, a variety of ketones reacted with diene **4** to give diols **5** with excellent enantioselectivities, diastereoselectivities, and good to excellent *Z*-selectivities. The reactions tolerated aromatic ketones with different electronic properties and substitution patterns, and diols **5a**–**l** were obtained with >20:1 diastereoselectivity, 96–99% ee, and 7:1 to >20:1 *Z*-selectivity. It is worth noting that acyclic ketones bearing two different alkyl groups also worked well under the developed conditions. For instance, the reaction with 2-butanone formed diol **5m** in 10:1 diastereoselectivity and *Z*-selectivity with 99% ee. In this case, the catalyst system is capable of differentiating a methyl vs an ethyl group. Other representative aliphatic ketones, including 2-octanone, cyclopropyl methyl ketone, and 3,3-dimethyl-2-butanone, all reacted under the standard conditions to afford diols **5n**–**p** in 18:1 to >20:1 *Z*-selectivity and diastereoselectivity with 99% ee. The reactions can be performed with 5 mol % CuOAc and 6 mol % (*R*,*R*)-Ph-BPE to afford diols **5** with comparable results. Moreover, boronate product **8** can be isolated from the reactions (bottom panel, Table 4). After completion of the reaction, the initial adduct **7** contains alkyl- and *Z*-vinyl-boronate groups. Both boryl groups can cyclize with the tertiary alcohol group in **7** to form a mixture of 5- and 6-membered oxaboracycles, which complicates the product isolation processes. However, the Cu-mediated bromination of **7** selectively occurred at the *Z*-vinyl-boronate group. After spontaneous cyclization of the tertiary alcohol to the alkyl boronate group, five-membered oxaboracycles **8a**–**c** and **8g** were isolated in 63–85% yields, 96–99% ee with 10:1 to 16:1 *Z*-selectivities and >20:1 diastereoselectivities. The structure assignment of diols **5** and the absolute configuration were confirmed by X-ray crystallography analyses of *ent-* **5e**.

**Table 4:**
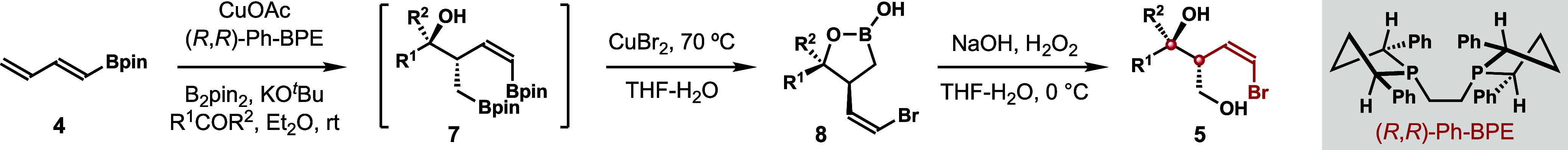

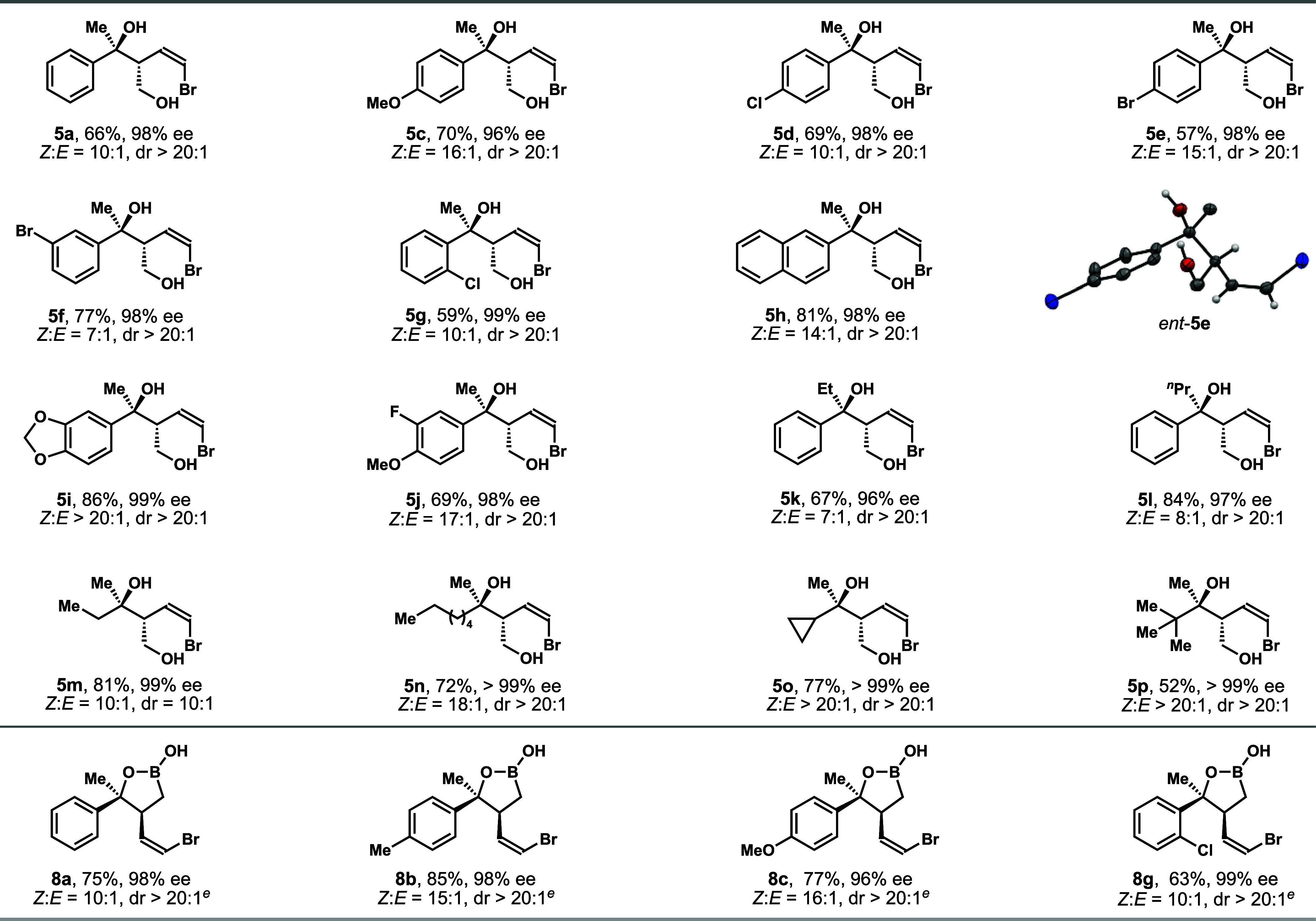
Scope of Asymmetric Coupling of Ketones with Dienylboronate **4** ^a^ ^‑^ ^f^

a Reaction conditions: dienylboronate **4** (0.1 mmol, 1.0 equiv), CuOAc (10 mol %), (*R*,*R*)-Ph-BPE (12 mol %), B_2_pin_2_ (1.5 equiv), KO*^t^* Bu (1.5 equiv), R^1^COR^2^ (1.5 equiv), Et_2_O (1.5 mL), rt; CuBr_2_ (3.0 equiv), THF-H_2_O, 70 °C; NaOH, H_2_O_2_, THF-H_2_O, 0 °C. Reactions of several substrates with 5 mol % CuOAc and 6 mol % (*R*,*R*)-Ph-BPE gave products **5** with similar yields, *Z*-selectivities, diastereo- and enantioselectivities.

b Diastereoselectivities and *Z*/*E*-selectivities were determined by ^1^H NMR analyses of the crude reaction mixtures.

c Yields of isolated products are listed.

d Enantiomeric excesses of **5** and **8** were determined by HPLC analysis using a chiral stationary phase.

e The reaction was conducted without oxidation.

f (*S*,*S*)-Ph-BPE was used for the preparation of *ent*-**5e** for X-ray analyses.

### Product Derivatization

Scheme 2 summarizes the derivatization studies conducted with the products obtained from these reactions. For example, the reaction of diene **1a** with acetophenone without oxidative workup formed 5-membered oxaboracycle **9** in 81% yield. Subsequent Pd-catalyzed Suzuki coupling of **9** with 4-bromoanisole afforded alcohol **10** in an 87% yield.^13^ The boryl group in **9** underwent homologation–oxidation to give diol **11** in a 72% yield.^14^ Vinylation of the boryl group in **9** also occurred to form product **12** in 73% yield.^15^ In addition, diol **2a** was converted into acetonide **13** in 92% yield, which provided structural support through NOE analyses. Protecting the hydroxyl groups of **2a** as TBS ethers afforded **14** in 78% yield. Iodination of the vinyl silane group of **14** with NIS produced vinyl iodide **15** in 57% yield,^16^ which could be used for cross-coupling with various nucleophiles. The reaction of 4-methyl-acetophenone with diene **4** formed the initial adduct **7b**, which was used directly for subsequent reactions. For instance, **7b** participated in Pd-catalyzed Suzuki coupling with ethyl *E*-iodoacrylate to give *Z*,*E*-diene **16** in 64% yield. Coupling of the alkyl boronate group was not detected. Similarly, Suzuki coupling of **7b** with ethyl *Z*-iodoacrylate delivered *Z*,*Z*-diene **17** in 70% yield. It is anticipated that the alkyl boronate group in **16** and **17** could engage in various reactions as shown with compound **9**. Additional support for the structural assignment of **5**/**8** was provided by NOE analyses of acetonide derivative **18**, which was produced from diol **5b**. Moreover, the vinyl bromide group in **18** can be used as a handle for cross-coupling reactions. For example, Pd-catalyzed coupling of **18** with phenyl boronic acid gave product **19** in 81% yield with perfect retention of the *Z*-olefin geometry. These derivatization studies highlight the synthetic versatility of the reaction products that could serve as valuable intermediates for organic synthesis.

**Scheme 2:**
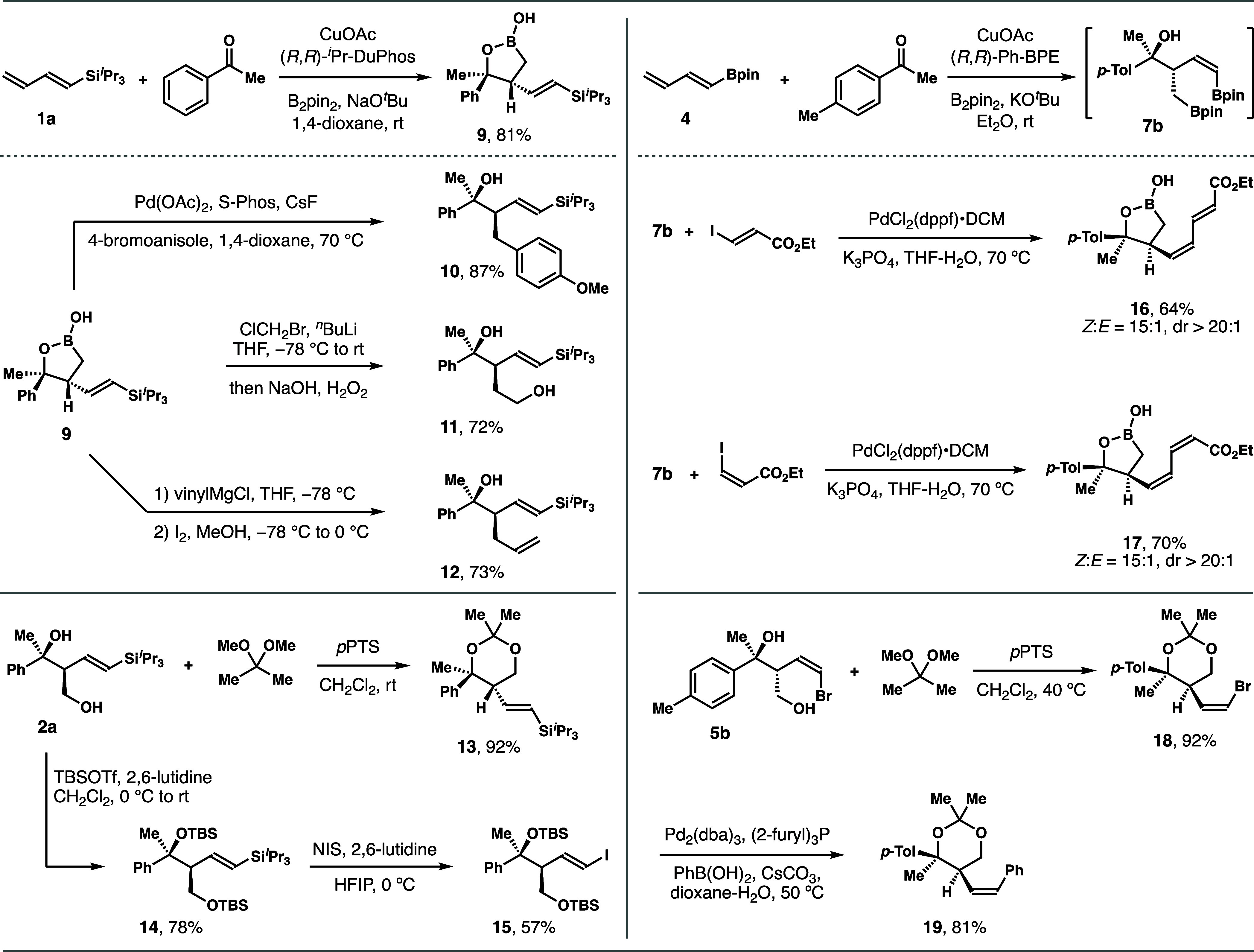
Derivatization of Reaction Products

### Reaction Pathway Analyses

As shown in Table 2, the reactions of ketones with dienylsilane **1a** form diol products **2** with an *E*-alkene group, and the tertiary hydroxyl group is *syn* to the hydroxymethyl group. By contrast, the reactions of ketones with dienylboronate **4** generate diols **5** with a *Z*-alkene group, and the tertiary hydroxyl group is *anti* to the hydroxymethyl group (Table 4). The stereodivergence in these reactions with dienes **1a** and **4** is unexpected and worth commenting. In particular, the trend persists when reactions were conducted with the same (*R*,*R*)-*^i^* Pr-DuPhos ligand under the same reaction conditions (Table 1, entry 1 and Table 3, entry 6). It has been shown that the addition of a bidentate phosphine ligand-ligated Cu-Bpin complex, L*Cu-Bpin, to 1,3-dienes occurs in a 1,2-addition manner.^9^ Depending on the rates of subsequent 1,3-metalloshifts, the process could in principle result in a mixture of four distinct allylcopper adducts, *E*-isomers *E*-**20**, *E*-**21**, and *Z*-isomers *Z*-**22** and *Z*-**23** (Scheme 3a). Reactions of allylic copper with carbonyl compounds are known to proceed by way of a chair-like, Zimmerman–Traxler transition state.^17^ The structural features of the major product diol **2a** (*syn*, bearing an *E*-vinyl silane unit) indicate that the allylation event should occur with allylcopper species *Z*-(*S*)-**23a** as the major reactive intermediate. The reaction should proceed via transition state **TS**-**3** to form product **2a** after oxidative workup (Scheme 3b). Similarly, the major reactive intermediate in the reaction with diene **4** should be allylic copper *E*-(*S*)-**21b**, where **TS**-**6** is the favored transition state en route to *anti*-*Z*-product **5a** (the tertiary hydroxyl group is *anti* to the hydroxymethyl group, Scheme 3b). The addition to ketone with allylic copper species *E*-**20** or *Z*-**22** did not occur in these reactions. Such a reactivity discrepancy of these allylic copper intermediates (**20**–**23**) is presumably due to the steric environment at the reactive site: a sterically more hindered R (Si*^i^* Pr_3_ or Bpin) group at the γ-positions of *E*-**20** and *Z*-**22** vs. a less bulky CH_2_Bpin group at the γ-positions of *E*-**21** and *Z*-**23**, which should disfavor the reactions from occurring via *E*-**20** or *Z*-**22**. Moreover, the electronic stabilization^18,19^ of the α-*^i^* Pr_3_Si or α-Bpin group in allyl copper intermediates *E*-**21** and *Z*-**23** could help to lower the energies of reaction transition states (e.g., **TS**-**3** and **TS**-**6**), which in turn will favor the reactions to proceed via intermediate *E*-**21** or *Z*-**23**. By contrast, such a stereoelectronic benefit is absent in the reaction transition states with allyl copper species *E*-(*R*)-**20** or *Z*-**22**.

**Scheme 3:**
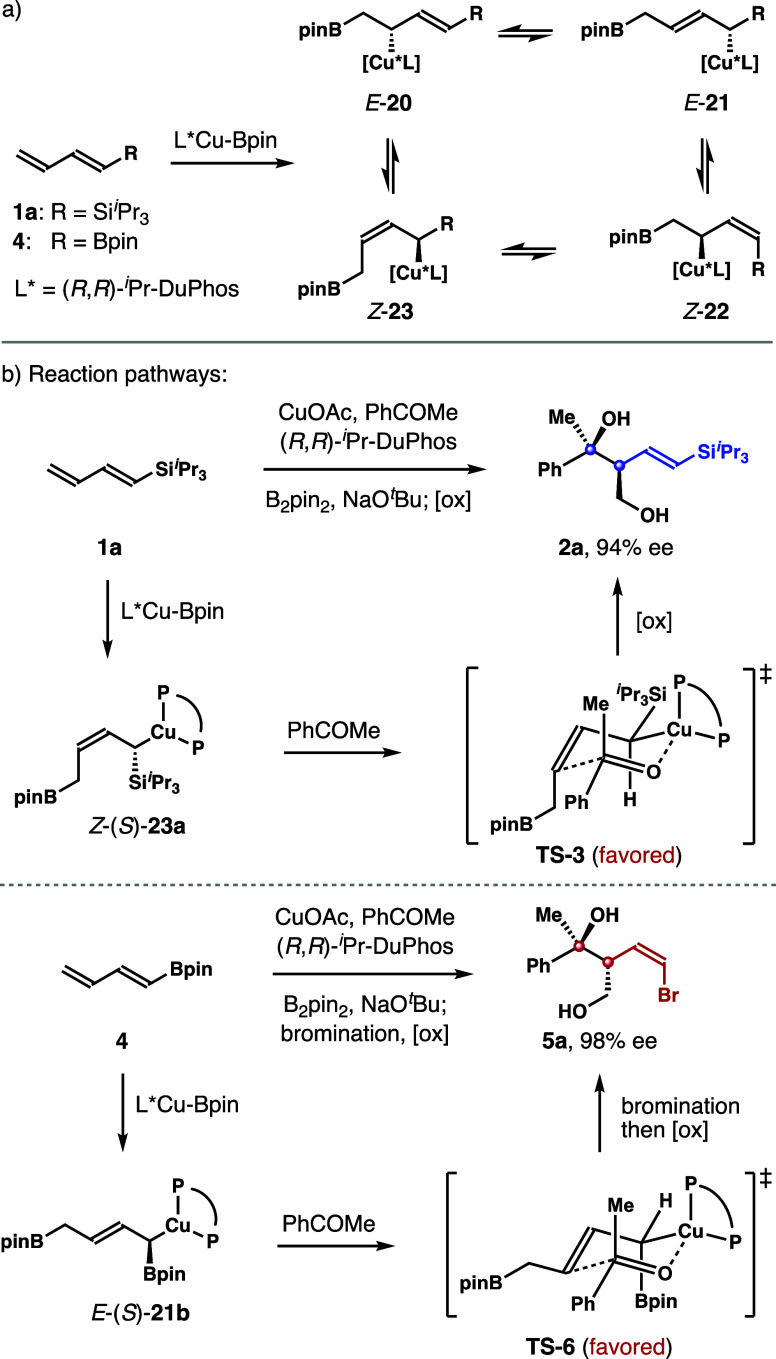
Analyses of the Reaction Pathways

### Computational Studies

To augment our analyses of the reaction pathways and, more importantly, to gain mechanistic insight into observed stereodivergence, DFT computational studies were conducted (see the SI for details). First, we computed the energies for enantioselective addition of the (*R*,*R*)-*^i^* Pr-DuPhos-ligated copper complex, L*Cu-Bpin, to diene **1a**. As shown in Scheme 4a, the addition to diene **1a** with an *s*-trans conformation via transition state **TS**-**1a** (bottom face as shown) produces adduct *E*-(*R*)-**20a**. The addition via the competing transition state, **TS**-**2a** (top face as shown), produces adduct *E*-(*S*)-**20a**. The results from computational studies showed that transition state **TS**-**2a** is 5 kJ/mol more energetic than **TS**-**1a** (Scheme 4b). The computational studies also revealed that the addition of L*Cu-Bpin could also occur to diene **1a** with the *s*-cis conformation, which is 17 kJ/mol thermodynamically less stable than *s*-trans diene **1a**. The addition to *s*-cis diene **1a** through **TS**-**1a′** (bottom face as shown) provides a transient ^3^η-Cu intermediate, which isomerizes to α-silyl allylcopper *Z*-(*S*)-**23a** through a low barrier (17 kJ/mol, not shown) 1,3-metalloshift. A similar reaction via competing transition state **TS**-**2a**′ (top face as shown) gives *Z*-(*R*)-**23a**, and this pathway is 21 kJ/mol more energetic than **TS**-**1a**′. Importantly, the barrier for *s*-trans to *s*-cis diene conversion is 33 kJ/mol, and the activation energy for the addition to *s*-cis diene **1a** via **TS**-**1a**′ is 12 kJ/mol after its binding to L*Cu-Bpin (Scheme 4b). The overall activation energy of this reaction pathway is thus limited by the isomerization and is much lower than that of **TS**-**1a** for the addition to *s*-trans diene **1a** (33 vs 54 kJ/mol). These results indicate that among the four potential reaction pathways from *s*-trans diene **1a**, the most favored one is *s*-trans to *s*-cis diene isomerization followed by diene addition via **TS**-**1a**′, which provides α-silyl allylcopper intermediate *Z*-(*S*)-**23a**. As shown in Scheme 4, we attribute the origin of such selectivity to the unfavorable steric interactions between the large *^i^* Pr_3_Si group of diene **1a** and the (*R*,*R*)-*^i^* Pr-DuPhos ligand on copper in **TS**-**1a** and **TS**-**2a**. Such steric interactions are absent in **TS**-**1a**′. On the other hand, it is conceivable that the same allylcopper intermediate, *Z*-(*S*)-**23a**, could also be generated via a 1,4-addition pathway to *s*-cis diene **1a** directly. However, the attempted location of the corresponding transition state failed even after extensive efforts. Overall, the addition of L*Cu-Bpin to *s*-cis diene **1a** is highly face selective and should predominantly proceed via favored transition state **TS**-**1a**′ to form *Z*-(*S*)-**23a** as the major product.

**Scheme 4:**
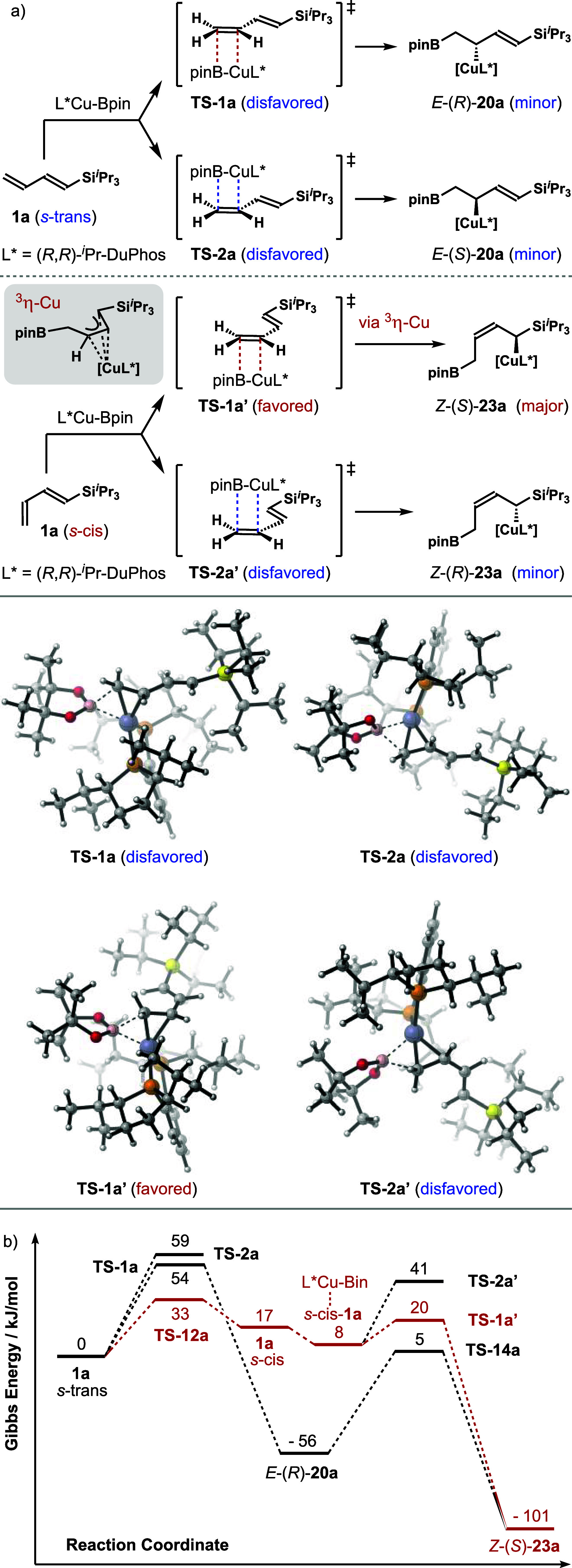
Borylcupration Reaction Pathways with **1a**

Owing to the reversibility of the 1,3-metalloshift, we initially thought that the initial diene addition adduct should equilibrate with other allylcopper species to generate an *E*- and *Z*-isomer mixture. In the case of dienylsilane **1a**, it was anticipated that *Z*-(*S*)-**23a** could equilibrate with *E*-(*R*)-**21a** via the intermediacy of *E*-(*R*)-**20a**. Both *Z*-(*S*)-**23a** and *E*-(*R*)-**21a** are potential reactive intermediates for the ketone addition event. As shown in Scheme 5a, addition of *Z*-(*S*)-**23a** to acetophenone should occur via transition state **TS**-**3** to produce *syn*-*E*-adduct **2a** upon oxidative workup. For the reaction with *E*-(*R*)-**21a**, two competing transition states, **TS**-**4** and **TS**-**5**, could be involved. The addition via **TS**-**4** forms *anti*-*Z*-product **24**, while the addition via **TS**-**5** gives *anti*-*E*-product **25**. The results from DFT calculations indicate that among four allylcopper intermediates, *Z*-(*S*)-**23a** is the most stable (Scheme 5b). The DFT data also indicate that although converting *Z*-(*S*)-**23a** to *E*-(*R*)-**20a** is viable through 1,3-metalloshifts (via **TS**-**14a**), the barrier of such a process is 106 kJ/mol, and this step is therefore slow. By contrast, the barrier for converting *E*-(*R*)-**20a** to *E*-(*R*)-**21a** is 6 kJ/mol (via **TS**-**13a**), and this step should be fast. Importantly, the activation energy for the ketone addition with *Z*-(*S*)-**23a** via transition state **TS**-**3** is 71 kJ/mol, which is 35 kJ/mol more favorable than the isomerization process to give *E*-(*R*)-**20a** (**TS**-**3** vs. **TS**-**14a**). Meanwhile, ketone allylation with *E*-(*R*)-**21a** via the transition state **TS**-**4** (to generate *Z*-adduct **24**) is 13 kJ/mol higher in energy than that of **TS**-**3**. The pathway to adduct **25** via **TS**-**5** is the least favored. The results are fully consistent with the experimental data, as *syn*-*E*-adduct **2a** was obtained in high *E*-selectivity and excellent enantioselectivity. As shown in Scheme 5, ketone addition with *Z*-(*S*)-**23a** via transition state **TS**-**3** occurs at the *Si* face of acetophenone, which matches the asymmetric induction of (*R*,*R*)-*^i^* Pr-DuPhos-ligated allylcopper for ketone addition.^20^ Moreover, this allylation process through **TS**-**3** could benefit from the stereoelectronic effect provided by the α-*^i^* Pr_3_Si group.^21^ Taken together, the overall reaction process with diene **1a** proceeded through the less stable *s*-cis conformer via a highly face-selective 1,2-addition pathway to provide a transient ^3^η-Cu intermediate, which isomerizes to α-silyl allylcopper *Z*-(*S*)-**23a**. The isomerization of *Z*-(*S*)-**23a** to *E*-(*R*)-**21a** (via the intermediacy of *E*-(*R*)-**20a**) is a minor pathway owing to the high energy barrier (**TS**-**14a**). Instead, the addition of *Z*-(*S*)-**23a** to the ketone substrate via **TS**-**3** is the predominant pathway that provides *syn*-*E*-adduct **2a** with high selectivity.

**Scheme 5:**
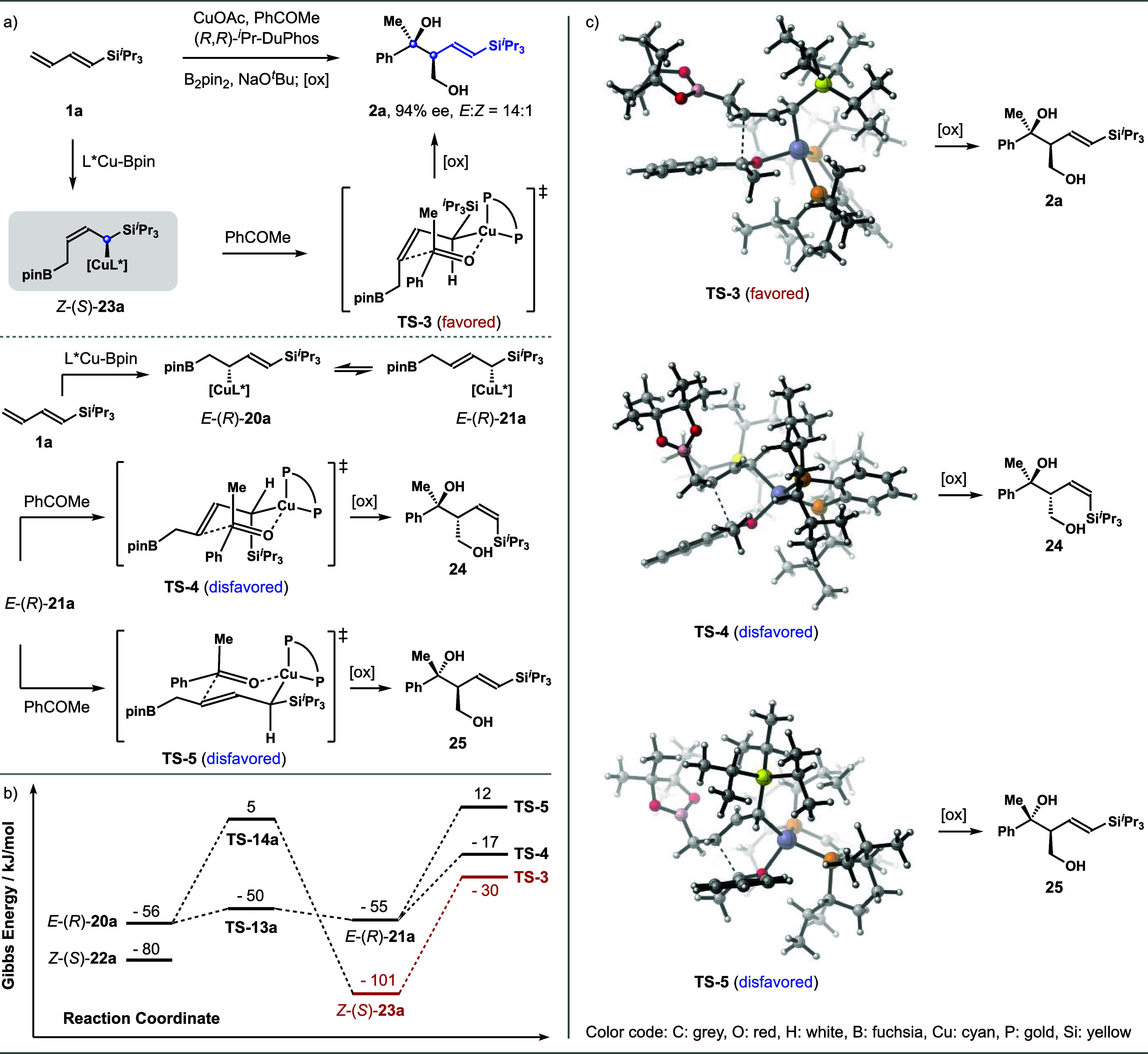
Calculated Structures and Relative Energies for the Ketone Addition Transition States Involving Diene **1a**

To obtain mechanistic insight into the observed stereodivergence, DFT calculations were conducted for the reactions involving dienylboronate **4** next. As shown in Scheme 6a, the addition of the (*R*,*R*)-*^i^* Pr-DuPhos-ligated Cu-Bpin complex to the two faces of dienylboronate **4** through the *s*-trans conformer via **TS**-**1b** and **TS**-**2b** leads to two products, *E*-(*R*)-**20b** and *E*-(*S*)-**20b**. The results from the DFT calculations revealed that the activation barriers for **TS**-**1b** and **TS**-**2b** are 6 and 11 kJ/mol, respectively. The corresponding barrier for *s*-trans to *s*-cis diene interconversion is 34 kJ/mol. Therefore, in the case of dienylboronate **4**, the *s*-trans to *s*-cis diene conversion process is not competitive, and the diene addition with L*Cu-Bpin proceeded via the *s*-trans conformer of **4** directly, which is in sharp contrast to the case of dienylsilane **1a**. We attribute this discrepancy to the much smaller size of the Bpin group in diene **4** compared to the large *^i^* Pr_3_Si group in diene **1a**. As a result, there is a minimal steric interaction between the (*R*,*R*)-*^i^* Pr-DuPhos-ligated copper catalyst and Bpin group of diene **4** in the transition states of the L*Cu-Bpin addition to the *s*-trans conformer of **4**. However, the energy difference between transition states **TS**-**1b** and **TS**-**2b** is 5 kJ/mol, favoring **TS**-**1b** (bottom face addition, Scheme 6a). The relatively small energy difference reflects about a 7–8 fold face selectivity at ambient temperature, which should result in the formation of a 7–8:1 mixture of *E*-(*R*)-**20b** and *E*-(*S*)-**20b**.

**Scheme 6:**
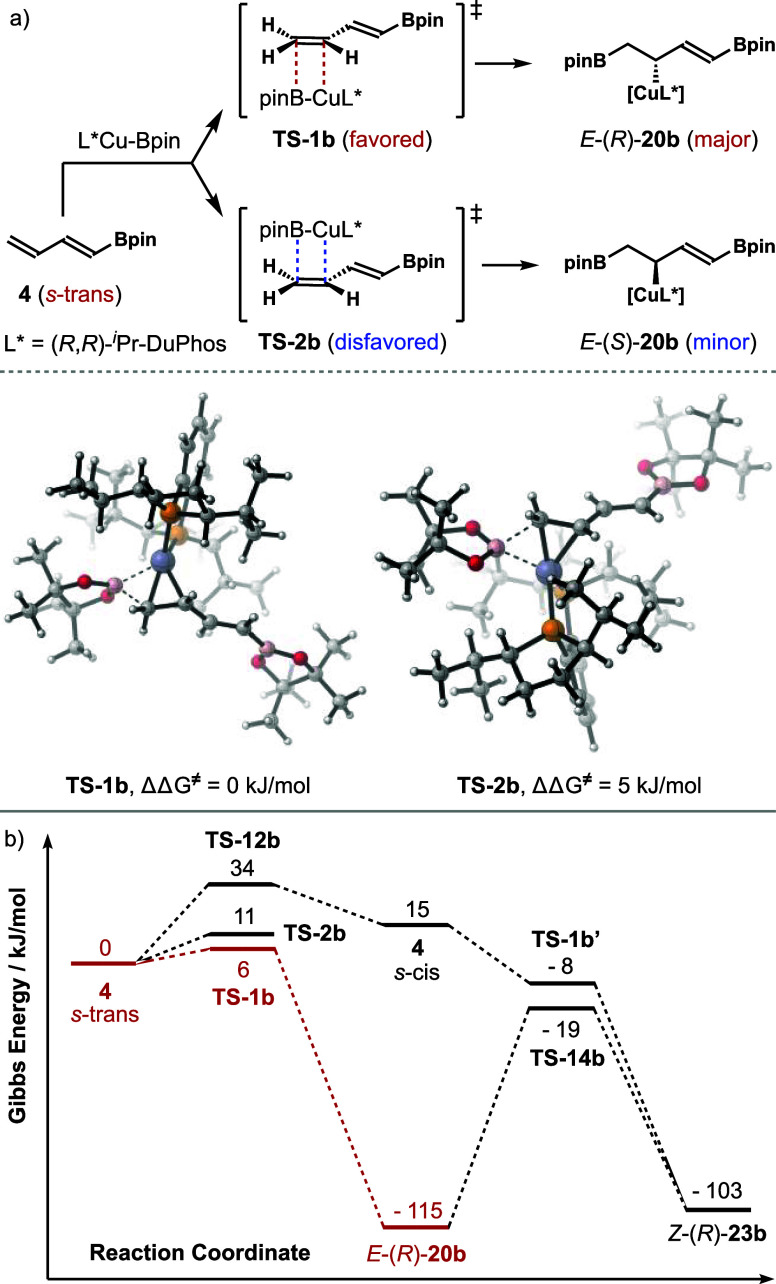
Mechanistic Analyses of the Borylcupration Reaction Pathways with Dienylboronate **4**

Next, we looked into the energy profiles of the isomerization processes of allylcopper intermediate *E*-(*R*)-**20b** to other allylcopper intermediates, as copper species *E*-(*S*)-**21b** and *Z*-(*R*)-**23b** are the potentially reactive intermediates. The reaction of acetophenone with *E*-(*S*)-**21b** could proceed via **TS**-**6** and **TS**-**7** to generate diol products **5a** and **26**, respectively. The reaction with *Z*-(*R*)-**23b**, on the other hand, should form *syn*-*E*-adduct **27** via **TS**-**8**. As shown in Scheme 7b, the results from DFT calculations revealed that among the four allylic copper species, *E*-(*R*)-**20b** is the most stable. The energy barrier for the isomerization of *E*-(*R*)-**20b** to *E*-(*S*)-**21b** is 26 kJ/mol, and the energy barrier for the isomerization of *E*-(*R*)-**20b** to *Z*-(*R*)-**23b** is 96 kJ/mol. This trend is consistent with the case of dienylsilane **1a**. For the ketone allylation processes, the addition of *E*-(*S*)-**21b** at the *Si* face of acetophenone via transition state **TS**-**6** is more favorable by 29 kJ/mol than the *Re* face addition via **TS**-**7** (Scheme 7b). Again, this ketone addition mode (**TS**-**6**) matches the asymmetric induction of the (*R*,*R*)-*^i^* Pr-DuPhos-ligated allylcopper species. In addition, the electronically positive Bpin group is oriented in a pseudoaxial position in **TS**-**6**, which will not decrease the reactivity of *E*-(*S*)-**21b**, as the C–B bond is perpendicular to the π* orbital of the alkene unit.^23^ The reaction via competing transition **TS**-**7** occurs at the *Re* face of the ketone, and such a mode of addition is mismatched with the asymmetric induction of the allylcopper intermediate *E*-(*S*)-**21b**.^20,22^ Moreover, the Bpin group is now placed in a pseudoequatorial position, which is parallel to the π* orbital of the alkene group of *E*-(*S*)-**21b**. This spatial arrangement will decrease the reactivity of *E*-(*S*)-**21b** owing to the electropositive nature of the Bpin group. Therefore, **TS**-**7** was strongly disfavored. Meanwhile, the addition of *Z*-(*R*)-**23b** to acetophenone via **TS**-**8** is disfavored by 12 kJ/mol compared to **TS**-**6**. Taken together, the overall reaction process with diene **4** proceeded through the more stable *s*-trans conformer via a 1,2-addition pathway to provide allylcopper intermediate *E*-(*R*)-**20b** (the initial addition process also produces a minor isomer *E*-(*S*)-**20b**). Then, *E*-(*R*)-**20b** undergoes reversible 1,3-metalloshifs to give reactive allylic copper intermediate *E*-(*S*)-**21b**, which adds to the ketone substrate via favored transition state **TS**-**6** to give *anti*-*Z*-adduct **5a**.

**Scheme 7:**
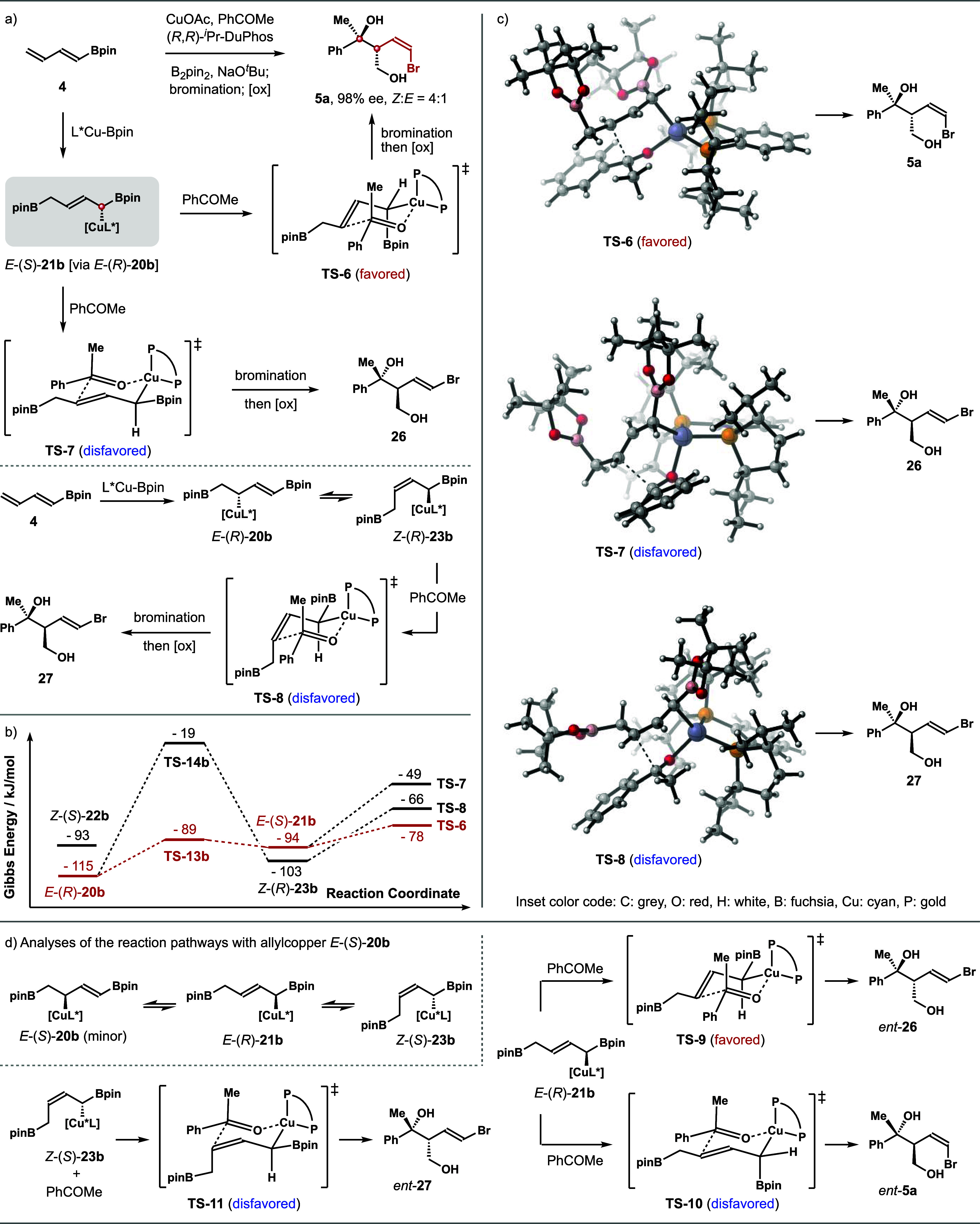
Calculated Structures and Energies for Ketone Addition Transition States Involving Dienylboronate **4**

As aforementioned, the addition of the L*Cu-Bpin complex to dienylboronate **4** forms a mixture of *E*-(*R*)-**20b** and *E*-(*S*)-**20b** with *E*-(*R*)-**20b** as the major adduct. The minor component, *E*-(*S*)-**20b**, could also engage in the ketone addition step via *E*-(*R*)-**21b** and/or *Z*-(*S*)-**23b** (Scheme 7d). Close inspection of transition states **TS**-**9**, **TS**-**10,** and **TS**-**11** indicates that **TS**-**9** should be the most favored transition state. **TS**-**10** and **TS**-**11** should be energetically less favorable, as they operate (*Re* face addition) against the asymmetric induction of the corresponding allylcopper species. As a result, *anti*-*E*-adduct *ent*-**26** should be produced from *E*-(*R*)-**21b**, which was derived from *E*-(*S*)-**20b**. The moderate face selectivity of the diene addition step with L*Cu-Bpin (ΔΔG^⧧^ = 5 kJ/mol, Scheme 6a) ultimately leads to moderate *Z*-selectivity, which is consistent with the results of product **5a** shown in entry 6 of Table 3 (the *Z*-selectivity was improved when (*R*,*R*)-Ph-BPE was employed as the ligand as shown in entry 11 of Table 3, presumably due to the improved face selectivity in the diene addition step). It is worth pointing out that the addition of the (*R*,*R*)-*^i^* Pr-DuPhos-ligated copper complex, L*Cu-Bpin, to diene **4** forms a pair of diastereomeric intermediates *E*-(*R*)-**20b** and *E*-(*S*)-**20b**. Both intermediates could react with the ketone via *E-*(*S*)*-* **21b** and *E-*(*R*)*-* **21b** to give diastereomeric products **5a** and *ent*-**26**. Therefore, the 5 kJ/mol energy difference between transition states **TS-1b** and **TS-2b** reflects the ratio of these two diastereomers, not their enantiopurities.

## Conclusions

In summary, we developed a Cu-catalyzed stereodivergent and enantioselective coupling of ketones with dienes. Under the developed conditions, the reactions of 1,3-butadienyl-triisopropylsilane with ketones provided *syn*-(*E*)-adducts with high *E*-selectivities and excellent regio-, diastereo-, and enantioselectivities. By contrast, the reactions of dienylboronate with ketones gave *anti*-(*Z*)-adducts with high *Z*-selectivities and excellent regio- and diastereoselectivities as well as optical purities. Detailed reaction pathway analyses revealed that the stereodivergence and enantioselectivity of the reaction originate from the distinct allylcopper species involved in the ketone addition step. DFT studies buttress the reaction pathway analyses. The orthogonal reactivities of the functional groups embedded in reaction products permit diverse chemoselective transformations, thereby providing a valuable platform for further derivatization. Synthetic applications of this method are currently ongoing.

## Supplementary Material



































































## Notes

1a-s-cis (XYZ)
1a-s-trans (XYZ)
4-s-cis (XYZ)
4-s-trans (XYZ)
E-R-20a (XYZ)
E-R-20b (XYZ)
E-R-21a (XYZ)
E-S-21b (XYZ)
NMR spectra for all new compounds (PDF)
HPLC analyses of optical purities (PDF)
TS-12a (XYZ)
TS-12b (XYZ)
TS-13a (XYZ)
TS-13b (XYZ)
TS-14a (XYZ)
TS-14b (XYZ)
TS-1a (XYZ)
TS-1a' (XYZ)
TS-1b (XYZ)
TS-1b'(XYZ)
TS-2a (XYZ)
TS-2a' (XYZ)
TS-2b (XYZ)
TS-3 (XYZ)
TS-4 (XYZ)
TS-5 (XYZ)
TS-6 (XYZ)
TS-7 (XYZ)
TS-8 (XYZ)
Z-R-23b (XYZ)
Z-S-22a (XYZ)
Z-S-22b (XYZ)
Z-S-23a (XYZ)
