# Supplementary material for: Asymmetric Ketone Diene Coupling via Stereodivergent Copper Catalysis
Source: J Am Chem Soc. 2026 Feb 2;148(5):4899–912. doi: 10.1021/jacs.5c06735 (PMC12903869; doi:10.1021/jacs.5c06735)
Supplement: Supplementary file 9 [file ja5c06735_si_009.pdf]

Asymmetric Ketone Diene Coupling via Stereodivergent Copper Catalysis

Jiaming Liu,[+] *Zheye Zhang,*[#] *Diego Troya*[#] *and Ming Chen**[#]

[+] School of Chemistry & Materials, Jiangsu Provincial Key Laboratory of Green & Functional Materials and Environmental Chemistry, Yangzhou University, Yangzhou, Jiangsu 225000, China

[#] Department of Chemistry, Virginia Tech, Blacksburg, Virginia 24061, United States

E-mail: mzc0102@vt.edu

Supporting Information: Experimental Procedures, Tabulated Spectroscopic Data, ^{1}H and ^{13}C Spectra of New Compounds

General Experimental Details. All reaction solvents were purified by passing through a solvent column composed of activated A-1 alumina. Unless indicated otherwise, all reactions were conducted under an atmosphere of argon using flame-dried or oven-dried (140 °C) glassware. The term "concentrated under reduced pressure" refers to the removal of solvents and other volatile materials using a rotary evaporator with the water bath temperature below 30 °C, followed by removal of residual solvent at high vacuum (< 0.2 mbar).

Proton nuclear magnetic resonance (^{1}H NMR) spectra were acquired on commercial instruments at 400, 500 and 600 MHz. Carbon-13 nuclear magnetic resonance (^{13}C NMR) spectra were acquired at 101, 126 and 151 MHz. The proton signal for the residual non-deuterated solvent (δ 7.26 for $CHCl_3$) was used as an internal reference for ^{1}H NMR spectra. For ^{13}C NMR spectra, chemical shifts are reported relative to the δ 77.36 resonance of $CHCl_3$. Coupling constants are reported in Hz. Optical rotations were measured on a Perkin Elmer 241 Automatic Polarimeter. High-resolution mass spectra were recorded on a commercial high-resolution mass spectrometer.

Analytical thin layer chromatography (TLC) was performed on Kieselgel 60 F254 glass plates precoated with a 0.25 mm thickness of silica gel. The TLC plates were visualized with UV light and/or by staining with Hanessian solution (ceric sulfate and ammonium molybdate in aqueous sulfuric acid) or $KMnO_4$. Column chromatography was generally performed using Kieselgel 60 (230-400 mesh) silica gel, typically using a 50-100:1 weight ratio of silica gel to crude product.

Table SI-1: Ligand optimization for the reactions with diene 1a

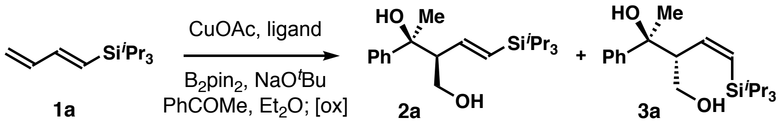

entry	L	*E*:*Z* (**2a**:**3a**)	dr (**2a**)	yield (%)	ee (**2a**) (%)
1	$\mathbf{L_1}$	2:1	2:1	ND	ND
2	$\mathbf{L_2}$	2:1	8:1	ND	ND
3	$\mathbf{L_3}$	1:1	4:1	ND	ND
4	$\mathbf{L_4}$	ND	1:2	ND	ND
5	$\mathbf{L_5}$	ND	ND	low conv.	ND
6	$\mathbf{L_6}$	1:1	> 20:1	88	ND
7	$\mathbf{L_7}$	14:1	> 20:1	84	91
8	$\mathbf{L_8}$	2:1	7:1	ND	ND

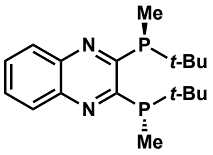

L$_1$: (*S*,*S*)-QuinoxP

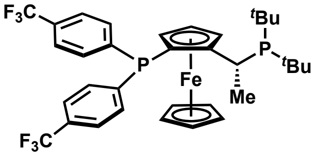

L$_2$: Josiphos SL-J011-1

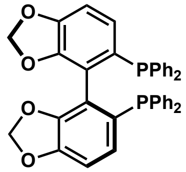

L$_3$: (*S*)-SegPhos

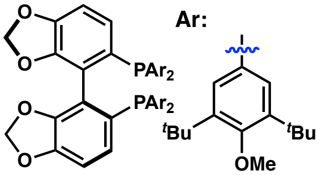

L$_4$: (*S*)-DTBM-SegPhos

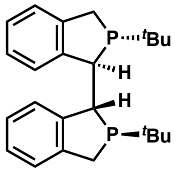

L$_5$: (+)-DuanPhos

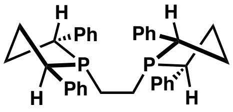

L$_6$: (*R*,*R*)-Ph-BPE

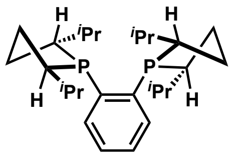

L$_7$: (*R*,*R*)-^{i}Pr-DuPhos

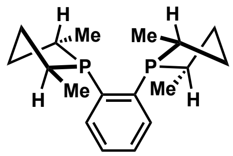

L$_8$: (*R*,*R*)-Me-DuPhos

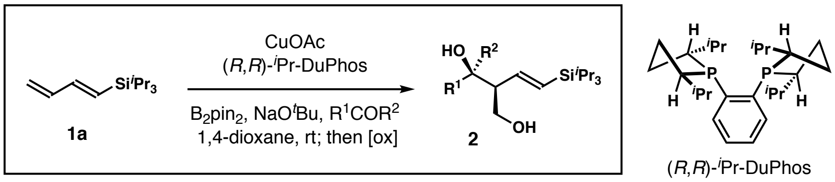

General procedure A for the syntheses of diols 2 from diene 1a: In an N_2-filled glove box, CuOAc (1 mg, 0.01 mmol, 10 mol %), (*R,R*)-^{i}Pr-DuPhos (5 mg, 0.012 mmol, 12 mol %), NaO^{t}Bu (14 mg, 0.15 mmol, 1.5 equiv), 1,4-dioxane (1.0 mL), and a Teflon-coated magnetic stirring bar were sequentially added into a reaction vial. The resulting mixture was stirred at ambient temperature for 15 min. B_2Pin_2 (38 mg, 0.15 mmol, 1.5 equiv) was added, and the mixture was stirred for 10 min. Then a solution of dienylsilane **1a** [1] (21 mg, 0.10 mmol, 1.0 equiv) and ketone (0.15 mmol, 1.5 equiv) in 1,4-dioxane (0.5 mL) were added in one portion to the reaction vial. The reaction mixture was stirred at ambient temperature inside the glove box and the reaction progress was monitored by ^1H NMR analysis. After complete consumption of dienylsilane **1a**, the reaction mixture was filtered through a short pad of silica gel and Celite. The filtrate was concentrated under reduced pressure. The resulting crude mixture was dissolved in THF (5 mL). Then an aqueous solution of NaOH (3N, 3 mL) was added followed by slow addition of 30% H_2O_2 (3 mL) at 0 °C. The resulting mixture was stirred vigorously for 2 h at 0 °C. EtOAc (5 mL) and brine (5 mL) was added. The organic layer was separated and the aqueous layer was extracted with EtOAc (5 mL x 3). The combined organic extracts were dried over anhydrous sodium sulfate, filtered, and concentrated under reduced pressure. Purification of the crude product was performed by flash column chromatography (gradient elution with hexane and ethyl acetate, 10:1 to 5:1) to give diol product **2**.

General procedure B for the syntheses of diols 2 from diene 1a: The same procedure as general procedure A with (*R,R*)-Ph-BPE (6 mg, 0.012 mmol, 12 mol %) as the ligand.

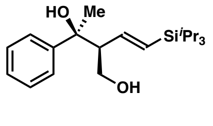

(2*S*,3*S*)-3-phenyl-2-((*E*)-2-(triisopropylsilyl)vinyl)butane-1,3-diol (2a) Prepared according to general procedure A. The crude mixture was purified by flash column to give compound **2a** as a white solid in 69% yield (24 mg, *E:Z* = 14:1, dr > 20:1). Enantiomeric excess was determined by HPLC analysis to be 94% ee (254 nm, 25 °C); t_1 = 4.64 min, t_2 = 5.01 min [(Chiralpak IC) hexane/i-PrOH, 90:10, 1.0 mL/min]; $[\alpha]_D^{20}$ = −1.0 (c 1.0, $CHCl_3$); A 5 mmol-scale reaction was conducted with 2.5 mol % CuOAc and 3 mol % (*R,R*)-^{i}Pr-DuPhos, affording

2a in 72% yield (1.255 g) and 94% ee. ^{1}H NMR (500 MHz, $CDCl_3$) δ 7.45 (d, *J* = 7.5 Hz, 2H), 7.35 (dd, *J* = 7.6, 7.6 Hz, 2H), 7.23 – 7.26 (m, 1H), 6.26 (dd, *J* = 19.0, 8.7 Hz, 1H), 5.69 (d, *J* = 19.0 Hz, 1H), 3.65 (dd, *J* = 10.6, 4.7 Hz, 1H), 3.60 (dd, *J* = 10.8, 5.0 Hz, 1H), 2.96 (brs, 1H), 2.58 – 2.62 (m, 1H), 1.96 (brs, 1H), 1.07 – 1.13 (m, 3H), 1.55 (s, 3H), 1.04 (d, *J* = 5.2 Hz, 9H), 1.03 (d, *J* = 6.7 Hz, 9H). ^{13}C NMR (126 MHz, $CDCl_3$) δ 147.8, 146.0, 130.2, 128.6, 127.1, 125.2, 77.3 (*via* HMBC), 64.7, 59.5, 28.8, 19.0, 11.2. HRMS (ESI$^+$): m/z for $C_{21}H_{36}O_2SiNa$ $[M+Na]^+$ calcd. 371.2382, found: 371.2374.

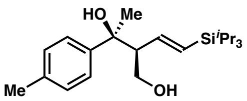

(2*S*,3*S*)-3-(*p*-tolyl)-2-((*E*)-2-(triisopropylsilyl)vinyl)butane-1,3-diol (2b) Prepared according to general procedure A. The crude mixture was purified by column chromatography to give compound **2b** as colorless oil in 77% yield (28 mg, *E:Z* = 17:1, dr > 20:1). Enantiomeric excess was determined by HPLC analysis to be 93% ee (254 nm, 25 °C); t_1 = 5.57 min, t_2 = 6.01 min [(Chiralpak ID) hexane/i-PrOH, 95:5, 1.0 mL/min]; $[\alpha]_D^{20}$ = −1.7 (c 1.1, $CHCl_3$); A 0.2 mmol-scale reaction was conducted with 5 mol % CuOAc and 6 mol % (*R,R*)-^{i}Pr-DuPhos, affording **2b** in 73% yield (53 mg) and 93% ee. ^{1}H NMR (600 MHz, $CDCl_3$) δ 7.32 (d, *J* = 8.3 Hz, 2H), 7.15 (d, *J* = 7.9 Hz, 2H), 6.24 (dd, *J* = 19.0, 8.7 Hz, 1H), 5.68 (d, *J* = 19.0 Hz, 1H), 3.65 (dd, *J* = 10.8, 4.9 Hz, 1H), 3.60 (dd, *J* = 10.7, 5.0 Hz, 1H), 2.85 (brs, 1H), 2.57 – 2.60 (m, 1H), 2.34 (s, 3H), 1.98 (brs, 1H), 1.54 (s, 3H), 1.07 – 1.11 (m, 3H), 1.03 – 1.04 (m, 18H). ^{13}C NMR (126 MHz, $CDCl_3$) δ 146.1, 144.9, 136.7, 130.1, 129.2, 125.1, 77.3 (*via* HMBC), 64.7, 59.6, 28.9, 21.3, 19.0, 11.2. HRMS (ESI$^+$): m/z for $C_{22}H_{38}O_2SiNa$ $[M+Na]^+$ calcd.385.2539, found: 385.2534.

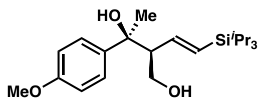

(2*S*,3*S*)-3-(4-methoxyphenyl)-2-((*E*)-2-(triisopropylsilyl)vinyl)butane-1,3-diol (2c) Prepared according to general procedure A. The crude mixture was purified by column chromatography to give compound **2c** as colorless oil in 77% yield (29 mg, *E:Z* = 18:1, dr > 20:1). Enantiomeric excess was determined by HPLC analysis to be 94% ee (254 nm, 25 °C); t_1 = 8.74 min, t_2 = 9.40 min [(Chiralpak IC) hexane/i-PrOH, 95:5, 1.0 mL/min]; $[\alpha]_D^{20}$ = −1.9 (c 0.8, $CHCl_3$); ^{1}H NMR (600 MHz, $CDCl_3$) δ 7.36 (d, *J* = 8.9 Hz, 2H), 6.87 (d, *J* = 8.9 Hz, 2H), 6.20 (dd, *J* = 19.0, 8.6 Hz, 1H), 5.66 (dd, *J* = 19.0, 0.9 Hz, 1H), 3.81 (s, 3H), 3.68 (dd, *J* = 10.7, 5.1 Hz, 1H), 3.61 (dd, *J* = 10.7, 5.1 Hz, 1H), 2.56 – 2.60 (m, 1H), 1.54 (s, 3H), 1.07 – 1.12 (m, 3H), 1.02 – 1.04 (m, 18H). ^{13}C NMR (126 MHz, $CDCl_3$) δ 158.7, 146.0, 140.0, 130.0, 126.4, 113.9, 77.3 (*via* HMBC), 64.7, 59.7, 55.6, 28.6, 19.0, 11.2. HRMS (ESI$^+$): m/z for $C_{22}H_{38}O_3SiNa$ $[M+Na]^+$ calcd. 401.2488, found: 401.2481.

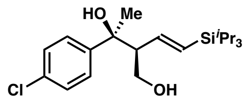

(2*S*,3*S*)-3-(4-chlorophenyl)-2-((*E*)-2-(triisopropylsilyl)vinyl)butane-1,3-diol (2d) Prepared according to general procedure A. The crude mixture was purified by flash column chromatography to give compound **2d** as colorless oil in 73% yield (28 mg, *E:Z* = 8:1, dr > 20:1). Enantiomeric excess was determined by HPLC analysis to be 95% ee (254 nm, 25 °C); t_1 = 4.97 min, t_2 = 5.29 min [(Chiralpak ID) hexane/i-PrOH, 95:5, 1.0 mL/min]; $[\alpha]_D^{20}$ = −1.9 (c 0.8, $CHCl_3$); ^{1}H NMR (500 MHz, $CDCl_3$) δ 7.39 (d, *J* = 8.5 Hz, 2H), 7.31 (d, *J* = 8.5 Hz, 2H), 6.23 (dd, *J* = 19.0, 8.7 Hz, 1H), 5.69 (d, *J* = 19.0 Hz, 1H), 3.65 – 3.69 (m, 1H), 3.59 – 3.63 (m, 1H), 3.22 (s, 1H), 2.54 – 2.58 (m, 1H), 1.90 (t, *J* = 5.2 Hz, 1H), 1.52 (s, 3H), 1.06 – 1.16 (m, 3H), 1.02 – 1.05 (m, 18H). ^{13}C NMR (126 MHz, $CDCl_3$) δ 146.5, 145.5, 132.9, 130.6, 128.6, 126.9, 77.3 (*via* HMBC), 64.8, 59.3, 28.7, 19.0, 11.2. HRMS (EI^+): m/z for $C_{21}H_{35}ClO_2SiNa$ $[M+Na]^+$ calcd. 405.1993, found: 405.1984.

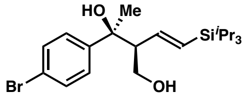

(2*S*,3*S*)-3-(4-bromophenyl)-2-((*E*)-2-(triisopropylsilyl)vinyl)butane-1,3-diol (2e) Prepared according to general procedure A. The crude mixture was purified by column chromatography to give compound **2e** as colorless oil in 68% yield (29 mg, *E:Z* = 8:1, dr > 20:1). Enantiomeric excess was determined by HPLC analysis to be 93% ee (254 nm, 25 °C); t_1 = 6.27 min, t_2 = 6.71 min [(Chiralpak IC) hexane/i-PrOH, 95:5, 1.0 mL/min]; $[\alpha]_D^{20}$ = −1.9 (c 1.2, $CHCl_3$); ^{1}H NMR (600 MHz, $CDCl_3$) δ 7.46 (d, *J* = 8.7 Hz, 2H), 7.33 (d, *J* = 8.7 Hz, 2H), 6.23 (dd, *J* = 19.0, 8.7 Hz, 1H), 5.69 (d, *J* = 19.1 Hz, 1H), 3.65 – 3.68 (m, 1H), 3.59 – 3.62 (m, 1H), 3.21 (s, 1H), 2.54 – 2.57 (m, 1H), 1.88 (s, 1H), 1.52 (s, 3H), 1.07 – 1.11 (m, 3H), 1.03 – 1.04 (m, 18H). ^{13}C NMR (126 MHz, $CDCl_3$) δ 147.1, 145.4, 131.6, 130.6, 127.2, 121.0, 77.3 (*via* HMBC), 64.9, 59.2, 28.6, 19.0, 11.2. HRMS (ESI^+): m/z for $C_{21}H_{35}BrO_2SiNa$ $[M+Na]^+$ calcd. 449.1487, found: 449.1480.

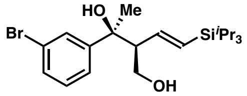

(2*S*,3*S*)-3-(3-bromophenyl)-2-((*E*)-2-(triisopropylsilyl)vinyl)butane-1,3-diol (2f) Prepared according to general procedure A. The crude mixture was purified by column chromatography to give compound **2f** as colorless oil in 66% yield (28 mg, *E:Z* = 8:1, dr > 20:1). Enantiomeric excess was determined by HPLC analysis to be 90% ee (254 nm, 25 °C); t_1 = 8.69 min, t_2 = 9.70 min [(Chiralpak IC) hexane/i-PrOH, 97:3, 1.0 mL/min]; $[\alpha]_D^{20}$ = −1.6 (c 1.0, $CHCl_3$); ^{1}H NMR (500 MHz, $CDCl_3$) δ 7.61 (s, 1H), 7.38 (dd, *J* = 7.9, 1.9 Hz, 2H), 7.21 (dd, *J* = 7.9, 7.9 Hz, 1H), 6.24 (dd, *J* = 19.1, 8.6 Hz, 1H), 5.71 (d, *J* = 18.9 Hz, 1H), 3.65 – 3.70 (m, 1H), 3.60 – 3.64 (m, 1H), 3.27 (s, 1H), 2.54 – 2.58 (m, 1H), 1.86 (t, *J* = 5.2 Hz, 1H), 1.52 (s, 3H), 1.08 – 1.15 (m, 3H), 1.03 – 1.05 (m, 18H). ^{13}C NMR (126 MHz,

$CDCl_3$) δ 150.5, 145.4, 130.7, 130.2, 130.1, 128.7, 124.0, 123.0, 77.3 (*via* HMBC), 64.9, 59.2, 28.7, 19.0, 11.2. HRMS (ESI$^-$): m/z for $C_{22}H_{36}BrO_4Si$ [M+HCOO]$^-$ calcd. 471.1566, found: 471.1566.

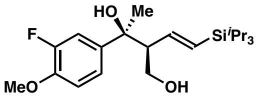

(2*S*,3*S*)-3-(3-fluoro-4-methylphenyl)-2-((*E*)-2-(triisopropylsilyl)vinyl)butane-1,3-diol (2g) Prepared according to general procedure A. The crude mixture was purified by flash column chromatography to give compound **2g** as colorless oil in 86% yield (34 mg, *E:Z* = 10:1, dr > 20:1). Enantiomeric excess was determined by HPLC analysis to be 91% ee (254 nm, 25 °C); t_1 = 8.52 min, t_2 = 9.13 min [(Chiralpak IC) hexane/i-PrOH, 95:5, 1.0 mL/min]; $[\alpha]_D^{20}$ = −1.4 (c 2.0, $CHCl_3$); ^{1}H NMR (500 MHz, $CDCl_3$) δ 7.17 – 7.19 (m, 1H), 7.12 – 7.14 (m, 1H), 6.91 – 6.94 (m, 1H), 6.19 (dd, *J* = 18.8, 8.6 Hz, 1H), 5.67 (d, *J* = 19.0 Hz, 1H), 3.88 (s, 3H), 3.66 – 3.71 (m, 1H), 3.60 – 3.64 (m, 1H), 3.14 (s, 1H), 2.52 – 2.56 (m, 1H), 1.96 (t, *J* = 5.3 Hz, 1H), 1.52 (s, 3H), 1.05 – 1.12 (m, 3H), 1.02 – 1.04 (m, 18H). ^{13}C NMR (126 MHz, $CDCl_3$) δ 152.5 (d, *J* = 245.0 Hz), 146.6 (d, *J* = 10.6 Hz), 145.6, 141.3 (d, *J* = 5.1 Hz), 130.4, 120.9 (d, *J* = 3.2 Hz), 113.7 (d, *J* = 19.8 Hz), 113.4 (d, *J* = 2.3 Hz), 76.8, 64.8, 59.4, 56.7, 28.4, 19.0, 11.1. HRMS (ESI$^+$): m/z for $C_{22}H_{37}FO_3Si$ [M+Na]$^+$ calcd. 419.2394, found: 419.2386.

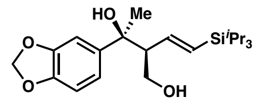

(2*S*,3*S*)-3-(benzo[*d*][1,3]dioxol-5-yl)-2-((*E*)-2-(triisopropylsilyl)vinyl)butane-1,3-diol (2h) Prepared according to general procedure A. The crude mixture was purified by column chromatography to give compound **2h** as colorless oil in 79% yield (31 mg, *E:Z* = 20:1, dr > 20:1). Enantiomeric excess was determined by HPLC analysis to be 95% ee (254 nm, 25 °C); t_1 = 10.1 min, t_2 = 13.4 min [(Chiralpak IC) hexane/i-PrOH, 95:5, 1.0 mL/min]; $[\alpha]_D^{20}$ = −1.9 (c 1.6, $CHCl_3$); ^{1}H NMR (500 MHz, $CDCl_3$) δ 6.95 (d, *J* = 1.8 Hz, 1H), 6.90 (dd, *J* = 8.1, 1.8 Hz, 1H), 6.77 (d, *J* = 8.2 Hz, 1H), 6.20 (dd, *J* = 18.9, 8.7 Hz, 1H), 5.95 (s, 2H), 5.67 (d, *J* = 18.9 Hz, 1H), 3.62 – 3.70 (m, 2H), 2.99 (s, 1H), 2.53 – 2.57 (m, 1H), 1.98 (t, *J* = 5.4 Hz, 1H), 1.52 (s, 3H), 1.06 – 1.13 (m, 3H), 1.02 – 1.04 (m, 18H). ^{13}C NMR (126 MHz, $CDCl_3$) δ 148.0, 146.6, 145.9, 142.2, 130.2, 118.4, 108.2, 106.4, 101.3, 77.2 (*via* HMBC), 64.8, 59.6, 28.7, 19.0, 11.2. HRMS (ESI$^+$): m/z for $C_{22}H_{36}O_4SiNa$ [M+Na]$^+$ calcd. 415.2281, found: 415.2273.

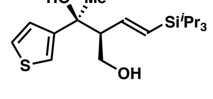

(2*S*,3*S*)-3-(thiophen-3-yl)-2-((*E*)-2-(triisopropylsilyl)vinyl)butane-1,3-diol (2i) Prepared according to general procedure A. The crude mixture was purified by column chromatography to give compound **2i**

as colorless oil in 71% yield (25 mg, E:Z = 8:1, dr > 20:1). Enantiomeric excess was determined by HPLC analysis to be 95% ee (254 nm, 25 °C); t_1 = 6.72 min, t_2 = 7.32 min [(Chiralpak IC) hexane/i-PrOH, 95:5, 1.0 mL/min]; $[\alpha]_D^{20}$ = −1.0 (c 2.0, $CHCl_3$); ^{1}H NMR (500 MHz, $CDCl_3$) δ 7.29 (dd, J = 5.1, 3.0 Hz, 1H), 7.19 (dd, J = 3.0, 1.4 Hz, 1H), 7.03 (dd, J = 5.0, 1.5 Hz, 1H), 6.14 (dd, J = 19.0, 8.7 Hz, 1H), 5.67 (d, J = 19.1 Hz, 1H), 3.72 – 3.77 (m, 1H), 3.64 – 3.68 (m, 1H), 2.97 (s, 1H), 2.58 – 2.62 (m, 1H), 2.06 (t, J = 5.5 Hz, 1H), 1.56 (s, 3H), 1.05 – 1.09 (m, 3H), 1.01 – 1.03 (m, 18H). ^{13}C NMR (126 MHz, $CDCl_3$) δ 149.9, 145.6, 130.3, 126.4, 125.8, 120.3, 76.3, 64.7, 59.4, 28.1, 19.0, 11.1. HRMS (ESI$^+$): m/z for $C_{19}H_{34}O_2SSiNa$ $[M+Na]^+$ calcd. 377.1946, found: 377.1940.

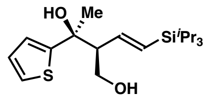

(2*S*,3*S*)-3-(thiophen-2-yl)-2-((*E*)-2-(triisopropylsilyl)vinyl)butane-1,3-diol (2j) Prepared according to general procedure A. The crude mixture was purified by column chromatography to give compound **2j** as colorless oil in 65% yield (23 mg, E:Z = 8:1, dr > 20:1). Enantiomeric excess was determined by HPLC analysis to be 95% ee (254 nm, 25 °C); t_1 = 6.91 min, t_2 = 7.54 min [(Chiralpak IC) hexane/i-PrOH, 95:5, 1.0 mL/min]; $[\alpha]_D^{20}$ = −0.4 (c 1.5, $CHCl_3$); ^{1}H NMR (600 MHz, $CDCl_3$) δ 7.20 (d, J = 5.0 Hz, 1H), 6.95 (dd, J = 5.1, 3.4 Hz, 1H), 6.90 (d, J = 3.5 Hz, 1H), 6.13 (dd, J = 19.0, 8.6 Hz, 1H), 5.69 (d, J = 19.0 Hz, 1H), 3.80 – 3.84 (m, 1H), 3.75 – 3.78 (m, 1H), 3.41 (s, 1H), 2.63 – 2.67 (m, 1H), 2.01 (t, J = 5.4 Hz, 1H), 1.63 (s, 3H), 1.05 – 1.08 (m, 3H), 1.01 – 1.03 (m, 18H). ^{13}C NMR (126 MHz, $CDCl_3$) δ 153.4, 145.2, 130.5, 127.1, 124.3, 122.9, 76.7, 64.9, 60.4, 28.9, 19.0, 11.1. HRMS (ESI$^+$): m/z for $C_{19}H_{34}O_2SSiNa$ $[M+Na]^+$ calcd. 377.1946, found: 377.1939.

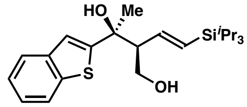

(2*S*,3*S*)-3-(benzo[*b*]thiophen-2-yl)-2-((*E*)-2-(triisopropylsilyl)vinyl)butane-1,3-diol (2k) Prepared according to general procedure A. The crude mixture was purified by column chromatography to give compound **2k** as colorless oil in 84% yield (34 mg, E:Z = 8:1, dr > 20:1). Enantiomeric excess was determined by HPLC analysis to be 93% ee (254 nm, 25 °C); t_1 = 7.03 min, t_2 = 7.60 min [(Chiralpak ID) hexane/i-PrOH, 95:5, 1.0 mL/min]; $[\alpha]_D^{20}$ = −1.3 (c 1.2, $CHCl_3$); ^{1}H NMR (600 MHz, $CDCl_3$) δ 7.79 (d, J = 8.0 Hz, 1H), 7.68 (d, J = 7.6 Hz, 1H), 7.32 (dd, J = 6.8, 6.8 Hz, 1H), 7.26 – 7.29 (m, 1H), 7.15 (s, 1H), 6.23 (dd, J = 19.0, 8.7 Hz, 1H), 5.74 (d, J = 19.0 Hz, 1H), 3.81 – 3.87 (m, 3H), 2.68 – 2.71 (m, 1H), 2.04 (s, 1H), 1.67 (s, 3H), 1.05 – 1.09 (m, 3H), 1.00 – 1.02 (m, 18H). ^{13}C NMR (126 MHz, $CDCl_3$) δ 154.1, 145.0, 140.2, 139.7, 130.7, 124.5, 124.2, 123.7, 122.6, 119.6, 76.9, 65.1, 59.7, 29.0, 18.9, 11.1. HRMS (ESI$^+$): m/z for $C_{23}H_{36}O_2SSiNa$ $[M+Na]^+$ calcd. 427.2103, found: 427.2096.

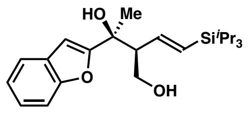

(2*S*,3*S*)-3-(benzofuran-2-yl)-2-((*E*)-2-(triisopropylsilyl)vinyl)butane-1,3-diol (2l) Prepared according to general procedure A. The crude mixture was purified by flash column chromatography to give compound **2l** as colorless oil in 77% yield (30 mg, *E:Z* = 5:1, dr > 20:1). Enantiomeric excess was determined by HPLC analysis to be 90% ee (254 nm, 25 °C); t_l = 7.81 min, t_2 = 8.76 min [(Chiralpak IC) hexane/i-PrOH, 95:5, 1.0 mL/min]; $[\alpha]_D^{20}$ = −2.1 (c 0.9, $CHCl_3$); ^1H NMR (600 MHz, $CDCl_3$) δ 7.52 (d, *J* = 7.8 Hz, 1H), 7.42 (d, *J* = 8.9 Hz, 1H), 7.23 – 7.26 (m, 1H), 7.20 (dd, *J* = 7.4, 7.4 Hz, 1H), 6.68 (s, 1H), 6.18 (dd, *J* = 19.0, 8.8 Hz, 1H), 5.76 (d, *J* = 18.0 Hz, 1H), 3.84 (dd, *J* = 10.9, 5.1 Hz, 1H), 3.73 (dd, *J* = 10.8, 5.0 Hz, 1H), 3.46 (brs, 1H), 2.87 – 2.90 (m, 1H), 1.92 (brs, 1H), 1.63 (s, 3H), 1.03 – 1.08 (m, 3H), 0.99 – 1.09 (m, 18H). ^{13}C NMR (126 MHz, $CDCl_3$) δ 162.5, 155.1, 144.7, 131.1, 128.6, 124.2, 123.1, 121.3, 111.5, 102.6, 74.8, 64.9, 57.3, 25.6, 18.9, 11.1. HRMS (ESI^+): m/z for $C_{23}H_{36}O_3SiNa$ $[M+Na]^+$ calcd. 411.2331, found: 411.2327.

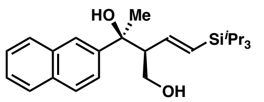

(2*S*,3*S*)-3-(naphthalen-2-yl)-2-((*E*)-2-(triisopropylsilyl)vinyl)butane-1,3-diol (2m) Prepared according to general procedure A. The crude mixture was purified by column chromatography to give compound **2m** as colorless oil in 80% yield (32 mg, *E:Z* = 17:1, dr > 20:1). Enantiomeric excess was determined by HPLC analysis to be 94% ee (254 nm, 25 °C); t_l = 6.30 min, t_2 = 9.48 min [(Chiralpak IC) hexane/i-PrOH, 90:10, 1.0 mL/min]; $[\alpha]_D^{20}$ = −1.9 (c 1.2, $CHCl_3$); ^1H NMR (600 MHz, $CDCl_3$) δ 7.95 (s, 1H), 7.18 – 7.84 (m, 3H), 7.53 (dd, *J* = 8.6, 1.9 Hz, 1H), 7.45 – 7.49 (m, 2H), 6.32 (dd, *J* = 19.0, 8.7 Hz, 1H), 5.73 (d, *J* = 19.0 Hz, 1H), 3.68 (dd, *J* = 10.7, 4.7 Hz, 1H), 3.63 (dd, *J* = 10.7, 4.9 Hz, 1H), 3.18 (brs, 1H), 2.71 – 2.74 (m, 1H), 1.94 (brs, 1H), 1.64 (s, 3H), 1.06 – 1.12 (m, 3H), 1.02 – 1.04 (m, 18H). ^{13}C NMR (126 MHz, $CDCl_3$) δ 145.9, 145.3, 133.5, 132.7, 130.3, 128.5, 128.3, 127.8, 126.5, 126.1, 123.8 (two overlapping carbon signals), 77.5, 64.9, 59.1, 28.9, 19.0, 11.2. HRMS (ESI^+): m/z for $C_{25}H_{38}O_2SiNa$ $[M+Na]^+$ calcd. 421.2539, found: 421.2537.

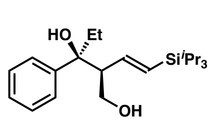

(2*S*,3*S*)-3-phenyl-2-((*E*)-2-(triisopropylsilyl)vinyl)pentane-1,3-diol (2n) Prepared according to general procedure A. The crude mixture was purified by flash column chromatography to give compound **2n** as colorless oil in 72% yield (26 mg, *E:Z* > 20:1, dr > 20:1. Enantiomeric excess was determined by HPLC analysis to be 90% ee (254 nm, 25 °C); t_l = 5.20 min, t_2 = 5.49 min [(Chiralpak IC) hexane/i-PrOH, 95:5, 1.0 mL/min]; $[\alpha]_D^{20}$ = −1.7 (c 1.1, $CHCl_3$); ^1H NMR (600 MHz, $CDCl_3$) δ 7.39 – 7.40 (m, 2H), 7.34 – 7.36 (m, 2H), 7.23 – 7.26 (m, 1H), 6.40 (dd, *J* = 19.0, 8.8 Hz, 1H), 5.79 (d, *J* = 19.1 Hz, 1H), 3.53 – 3.59 (m, 2H), 2.96 (s,

1H), 2.57 – 2.61 (m, 1H), 1.88 – 1.94 (m, 1H), 1.81 – 1.87 (m, 1H), 1.73 (dd, *J* = 6.1, 5.0 Hz, 1H), 1.11 – 1.17 (m, 3H), 1.05 – 1.08 (m, 18H), 0.64 (t, *J* = 7.4 Hz, 3H). ^{13}C NMR (126 MHz, $CDCl_3$) δ 146.5, 145.2, 130.2, 128.5, 126.8, 125.9, 79.7, 64.8, 59.4, 34.1, 19.1, 11.2, 7.7. HRMS (ESI$^+$): m/z for $C_{22}H_{38}O_2SiNa$ $[M+Na]^+$ calcd. 385.2539, found: 385.2532.

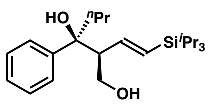

(2*S*,3*S*)-3-phenyl-2-((*E*)-2-(triisopropylsilyl)vinyl)hexane-1,3-diol (2o) Prepared according to general procedure A. The crude mixture was purified by flash column chromatography to give compound **2o** as colorless oil in 56% yield (21 mg, *E:Z* > 20:1, dr > 20:1). Enantiomeric excess was determined by HPLC analysis to be 85% ee (254 nm, 25 °C); t_1 = 5.05 min, t_2 = 5.38 min [(Chiralpak IC) hexane/i-PrOH, 95:5, 1.0 mL/min]; $[\alpha]_D^{20}$ = −1.3 (c 1.5, $CHCl_3$); ^{1}H NMR (600 MHz, $CDCl_3$) δ 7.40 (d, *J* = 8.2 Hz, 2H), 7.34 (t, *J* = 7.1 Hz, 2H), 7.24 (t, *J* = 8.0 Hz, 1H), 6.41 (dd, *J* = 19.1, 8.8 Hz, 1H), 5.79 (d, *J* = 19.0 Hz, 1H), 3.53 – 3.59 (m, 2H), 2.96 (s, 1H), 2.56 – 2.59 (m, 1H), 1.82 – 1.87 (m, 1H), 1.75 – 1.80 (m, 1H), 1.70 (t, *J* = 5.5 Hz, 1H), 1.12 – 1.23 (m, 4H), 1.05 – 1.09 (m, 18H), 0.83 – 0.90 (m, 1H), 0.79 (t, *J* = 7.4 Hz, 3H). ^{13}C NMR (126 MHz, $CDCl_3$) δ 146.5, 145.7, 130.3, 128.5, 126.8, 125.7, 79.5, 64.7, 59.5, 44.2, 19.1, 19.0, 16.6, 14.7, 11.2. HRMS (ESI$^+$): m/z for $C_{23}H_{40}O_2SiNa$ $[M+Na]^+$ calcd. 399.2695, found: 399.2687.

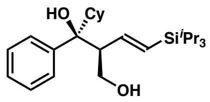

(1*R*,2*R*)-1-cyclohexyl-1-phenyl-2-((*E*)-2-(triisopropylsilyl)vinyl)propane-1,3-diol (2p) Prepared according to general procedure B. The crude mixture was purified by flash column chromatography to give compound **2p** as a white solid in 60% yield (25 mg, *E:Z* > 20:1, dr > 20:1). Enantiomeric excess was determined by HPLC analysis to be 96% ee (254 nm, 25 °C); t_1 = 4.41 min, t_2 = 4.95 min [(Chiralpak ID) hexane/i-PrOH, 95:5, 1.0 mL/min]; $[\alpha]_D^{20}$ = −2.0 (c 0.3, $CHCl_3$); A 0.2 mmol-scale reaction was conducted with 5 mol % CuOAc and 6 mol % (*S,S*)-Ph-BPE, affording *ent*-**2p** in 66% yield (55 mg) and 96% ee. ^{1}H NMR (500 MHz, $CDCl_3$) δ 7.40 (d, *J* = 8.5 Hz, 2H), 7.33 (dd, *J* = 7.9, 7.9 Hz, 2H), 7.24 – 7.26 (m, 1H), 6.47 (dd, *J* = 19.0, 8.7 Hz, 1H), 5.89 (d, *J* = 18.6 Hz, 1H), 3.57 – 3.65 (m, 2H), 3.18 (s, 1H), 2.97 – 3.00 (m, 1H), 1.88 – 1.90 (m, 1H), 1.74 – 1.80 (m, 2H), 1.64 – 1.67 (m, 2H), 1.52 – 1.54 (m, 2H), 1.12 – 1.19 (m, 4H), 1.09 (d, *J* = 6.0 Hz, 9H), 1.08 (d, *J* = 6.7 Hz, 9H), 0.85 – 0.93 (m, 3H), 0.34 – 0.42 (m, 1H). ^{13}C NMR (126 MHz, $CDCl_3$) δ 146.8, 143.3, 130.1, 127.9, 126.9, 126.8, 81.0, 65.4, 55.0, 47.5, 27.7, 27.0, 26.9, 26.8, 26.7, 19.1, 11.2. HRMS (ESI$^+$): m/z for $C_{26}H_{44}O_2SiNa$ $[M+Na]^+$ calcd. 439.3008, found: 439.3001.

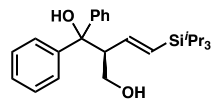

(*R*,*E*)-1,1-diphenyl-2-(2-(triisopropylsilyl)vinyl)propane-1,3-diol (2q) Prepared according to general procedure B. The crude mixture was purified by flash column chromatography to give compound **2q** as a white solid in 90% yield (37 mg, *E:Z* > 20:1). Enantiomeric excess was determined by HPLC analysis to be 97% ee (254 nm, 25 °C); t_1 = 8.00 min, t_2 = 10.2 min [(Chiralpak IC) hexane/i-PrOH, 95:5, 1.0 mL/min]; $[\alpha]_D^{20}$ = −0.7 (c 1.6, $CHCl_3$); A 0.2 mmol-scale reaction was conducted with 5 mol % CuOAc and 6 mol % (*S,S*)-Ph-BPE, affording *ent*-**2q** in 85% yield (70 mg) and 97% ee. ^{1}H NMR (600 MHz, $CDCl_3$) δ 7.55 (d, *J* = 7.3 Hz, 2H), 7.48 (d, *J* = 7.4 Hz, 2H), 7.31 (dd, *J* = 7.8, 7.8 Hz, 2H), 7.24 (dd, *J* = 7.9, 7.9 Hz, 2H), 7.19 (dd, *J* = 7.3, 7.3 Hz, 1H), 7.12 (dd, *J* = 7.4, 7.4 Hz, 1H), 6.23 (dd, *J* = 19.3, 7.6 Hz, 1H), 5.79 (d, *J* = 19.2 Hz, 1H), 3.83 – 3.85 (m, 2H), 3.82 (s, 1H), 3.43 – 3.46 (m, 1H), 1.70 (t, *J* = 5.7 Hz, 1H), 0.96 – 1.03 (m, 3H), 0.93 (d, *J* = 6.7 Hz, 9H), 0.90 (d, *J* = 7.1 Hz, 9H). ^{13}C NMR (126 MHz, $CDCl_3$) δ 146.8, 146.24, 146.21, 129.9, 128.7, 128.4, 127.0, 126.8, 126.3, 125.9, 80.5, 64.8, 55.8, 18.9, 18.8, 11.1. HRMS (ESI$^+$): m/z for $C_{26}H_{38}O_2SiNa$ $[M+Na]^+$ calcd. 433.2539, found: 433.2531.

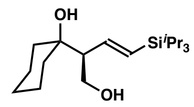

(*R*,*E*)-1-(1-hydroxy-4-(triisopropylsilyl)but-3-en-2-yl)cyclohexan-1-ol (2r) Prepared according to general procedure B. The crude mixture was purified by column chromatography to give compound **2r** as colorless oil in 70% yield (23 mg, *E:Z* > 20:1). Enantiomeric excess was determined by HPLC analysis to be 96% ee (254 nm, 25 °C); t_1 = 7.45 min, t_2 = 9.08 min [(Chiralpak ID) hexane/i-PrOH, 95:5, 1.0 mL/min]; $[\alpha]_D^{20}$ = −0.7 (c 2.4, $CHCl_3$); ^{1}H NMR (600 MHz, $CDCl_3$) δ 6.06 (dd, *J* = 18.9, 9.0 Hz, 1H), 5.70 (d, *J* = 18.9 Hz, 1H), 3.87 – 3.91 (m, 1H), 3.76 – 3.80 (m, 1H), 2.32 – 2.35 (m, 2H), 2.05 (s, 1H), 1.64 – 1.67 (m, 1H), 1.52 – 1.62 (m, 7H), 1.39 – 1.43 (m, 1H), 1.18 – 1.22 (m, 1H), 1.07 – 1.14 (m, 3H), 1.04 – 1.05 (m, 18H). ^{13}C NMR (126 MHz, $CDCl_3$) δ 146.3, 130.0, 74.1, 63.4, 59.2, 37.1, 34.9, 26.1, 22.1, 21.9, 19.0, 11.2. HRMS (ESI$^+$): m/z for $C_{19}H_{38}O_2SiNa$ $[M+Na]^+$ calcd. 349.2539, found: 349.2533.

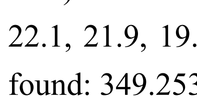

(*R*,*E*)-4-(1-hydroxy-4-(triisopropylsilyl)but-3-en-2-yl)tetrahydro-2*H*-pyran-4-ol (2s) Prepared according to general procedure B. The crude mixture was purified by column chromatography to give compound **2s** as colorless oil in 73% yield (24 mg, *E:Z* > 20:1). Enantiomeric excess was determined by HPLC analysis to be 99% ee (254 nm, 25 °C); t_1 = 11.0 min, t_2 = 12.4 min [(Chiralpak ID) hexane/i-PrOH, 95:5, 1.0 mL/min]; $[\alpha]_D^{20}$ = −1.7 (c 0.8, $CHCl_3$); ^{1}H NMR (500 MHz, $CDCl_3$) δ 6.08 (dd, *J* = 19.0, 8.9 Hz, 1H), 5.74 (d, *J* = 18.9 Hz, 1H), 3.89 (*app.* d, *J* = 5.6

Hz, 2H), 3.75 – 3.83 (m, 4H), 2.73 (brs, 1H), 2.28 – 2.32 (m, 1H), 1.94 (brs, 1H), 1.76 – 1.82 (m, 1H), 1.66 – 1.72 (m, 1H), 1.53 – 1.59 (m, 2H), 1.07 – 1.12 (m, 3H), 1.04 – 1.05 (m, 18H). ^{13}C NMR (126 MHz, $CDCl_3$) δ 145.2, 130.9, 71.2, 63.8, 63.7, 63.6, 59.3, 37.3, 35.6, 19.02, 19.00, 11.1. HRMS (ESI^+): m/z for $C_{18}H_{36}O_3SiNa$ $[M+Na]^+$ calcd. 351.2331, found: 351.2327.

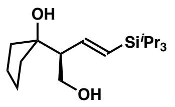

(*R*,*E*)-1-(1-hydroxy-4-(triisopropylsilyl)but-3-en-2-yl)cyclopentan-1-ol (2t) Prepared according to general procedure B. The crude mixture was purified by column chromatography to give compound **2t** as colorless oil in 61% yield (19 mg, *E:Z* > 20:1). Enantiomeric excess was determined by HPLC analysis to be 98% ee (254 nm, 25 °C); t_1 = 7.92 min, t_2 = 9.54 min [(Chiralpak ID) hexane/i-PrOH, 95:5, 1.0 mL/min]; $[\alpha]_D^{20}$ = −1.2 (c 0.4, $CHCl_3$); ^{1}H NMR (600 MHz, $CDCl_3$) δ 6.11 (dd, *J* = 19.0, 8.6 Hz, 1H), 5.74 (dd, *J* = 19.0, 0.9 Hz, 1H), 3.86 – 3.87 (m, 2H), 2.36 – 2.40 (m, 1H), 2.21 (t, *J* = 5.7 Hz, 1H), 2.05 (s, 1H), 1.78 – 1.84 (m, 2H), 1.68 – 1.73 (m, 2H), 1.63 – 1.64 (m, 4H), 1.08 – 1.13 (m, 3H), 1.03 – 1.04 (m, 18H). ^{13}C NMR (151 MHz, $CDCl_3$) δ 146.5, 129.5, 84.9, 64.9, 58.2, 39.6, 38.5, 24.1, 23.9, 19.0, 11.1. HRMS (ESI^+): m/z for $C_{18}H_{37}O_2Si$ $[M+H]^+$ calcd. 313.2563, found: 313.2559.

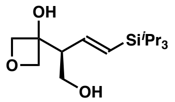

(*R*,*E*)-3-(1-hydroxy-4-(triisopropylsilyl)but-3-en-2-yl)oxetan-3-ol (2u) Prepared according to general procedure B. The crude mixture was purified by column chromatography to give compound **2u** as colorless oil in 73% yield (22 mg, *E:Z* > 20:1). Enantiomeric excess was determined by HPLC analysis to be 96% ee (254 nm, 25 °C); t_1 = 6.49 min, t_2 = 7.12 min [(Chiralpak IC) hexane/i-PrOH, 95:5, 1.0 mL/min]; $[\alpha]_D^{20}$ = −0.8 (c 0.6, $CHCl_3$); ^{1}H NMR (500 MHz, $CDCl_3$) δ 6.12 (dd, *J* = 19.0, 8.3 Hz, 1H), 5.84 (d, *J* = 19.1 Hz, 1H), 4.63 (*app.* s, 2H), 4.61 (d, *J* = 7.2 Hz, 1H), 4.55 (d, *J* = 6.9 Hz, 1H), 3.93 (dd, *J* = 10.8, 4.7 Hz, 1H), 3.88 (dd, *J* = 10.8, 6.0 Hz, 1H), 3.52 (brs, 1H), 2.79 – 2.83 (m, 1H), 1.79 (brs, 1H), 1.07 – 1.16 (m, 3H), 1.04 (d, *J* = 6.9 Hz, 18H). ^{13}C NMR (126 MHz, $CDCl_3$) δ 143.3, 131.1, 83.9, 82.7, 77.0, 63.5, 54.3, 18.94, 18.92, 11.1. HRMS (ESI^+): m/z for $C_{16}H_{33}O_3Si$ $[M+H]^+$ calcd. 301.2199, found: 301.2195.

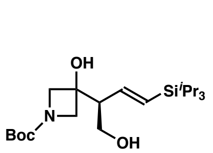

***tert*-butyl (*R*,*E*)-3-hydroxy-3-(1-hydroxy-4-(triisopropylsilyl)but-3-en-2-yl)azetidine-1-carboxylate (2v)** Prepared according to general procedure B. The crude mixture was purified by flash column chromatography to give compound **2v** as colorless oil in 45% yield (18 mg, *E:Z* > 20:1). Enantiomeric excess was determined by HPLC analysis to be 94% ee (254 nm, 25 °C); t_1

= 8.38 min, t_2 = 9.77 min [(Chiralpak IG) hexane/i-PrOH, 95:5, 1.0 mL/min]; $[\alpha]_D^{20}$ = −0.9 (c 3.0, $CHCl_3$); ^1H NMR (600 MHz, $CDCl_3$) δ 6.06 (dd, J = 19.3, 8.3 Hz, 1H), 5.83 (d, J = 19.0 Hz, 1H), 3.92 – 3.97 (m, 3H), 3.86 – 3.90 (m, 1H), 3.84 (d, J = 9.2 Hz, 1H), 3.77 (d, J = 9.4 Hz, 1H), 3.62 (s, 1H), 2.59 – 2.62 (m, 1H), 1.83 (t, J = 5.0 Hz, 1H), 1.43 (s, 9H), 1.07 – 1.14 (m, 3H), 1.03 (d, J = 6.8 Hz, 18H). ^{13}C NMR (151 MHz, $CDCl_3$) δ 156.6, 143.2, 131.4, 79.9, 72.9, 63.8, 54.8, 28.7, 19.0, 18.9, 11.1.

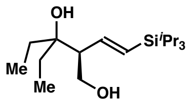

(*R*,*E*)-3-ethyl-2-(2-(triisopropylsilyl)vinyl)pentane-1,3-diol (2w) Prepared according to general procedure B. The crude mixture was purified by column chromatography to give compound **2w** as colorless oil in 54% yield (17 mg, *E:Z* > 20:1). Enantiomeric excess was determined by HPLC analysis to be 92% ee (254 nm, 25 °C); t_1 = 7.09 min, t_2 = 10.0 min [(Chiralpak IG) hexane/i-PrOH, 95:5, 1.0 mL/min]; ^1H NMR (600 MHz, $CDCl_3$) δ 6.10 (dd, J = 18.9, 9.0 Hz, 1H), 5.72 (d, J = 18.9 Hz, 1H), 3.85 (dd, J = 10.7, 5.8 Hz, 1H), 3.80 (dd, J = 10.8, 6.0 Hz, 1H), 2.43 – 2.47 (m, 1H), 1.48 – 1.73 (m, 5H), 1.07 – 1.13 (m, 3H), 1.04 (d, J = 6.9 Hz, 18H), 0.90 (t, J = 7.5 Hz, 3H), 0.87 (t, J = 7.5 Hz, 3H). ^{13}C NMR (151 MHz, $CDCl_3$) δ 146.4, 129.7, 76.9, 63.8, 55.8, 29.39, 29.35, 19.0, 11.2, 7.8, 7.7.

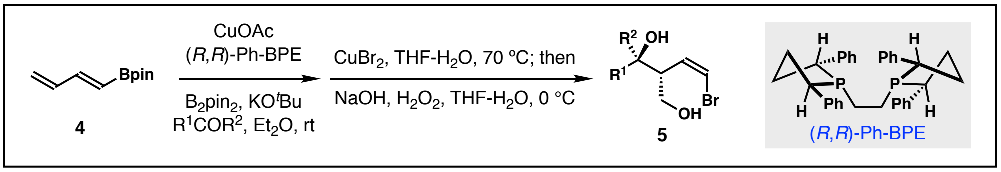

General procedure for the syntheses of diol 5 from diene 4: In an N_2-filled glove box, CuOAc (1 mg, 0.01 mmol, 10 mol %), (*R,R*)-Ph-BPE (6 mg, 0.012 mmol, 12 mol %), KO^{t}Bu (17 mg, 0.15 mmol, 1.5 equiv), Et_2O (1.0 mL), and a Teflon-coated magnetic stirring bar were sequentially added into a reaction vial. The resulting mixture was stirred at ambient temperature for 15 min. B_2Pin_2 (38 mg, 0.15 mmol, 1.5 equiv) was added and the mixture was stirred for 10 min. Then a solution of dienylboronate **4** [2] (18 mg, 0.10 mmol, 1.0 equiv) and ketone (0.15 mmol, 1.5 equiv) in Et_2O (0.5 mL) were added to the reaction mixture in one portion. The resulting reaction mixture was stirred at ambient temperature inside the glove box and the reaction progress was monitored by ^1H NMR analysis. After complete consumption of dienylboronate **4**, the reaction mixture was filtered through a short pad of silica gel and Celite. The filtrate was concentrated under reduced pressure. Then THF (0.5 mL) and H_2O (0.5 mL) were added to the crude reaction mixture followed by addition of $CuBr_2$ (67 mg, 0.3 mmol, 3.0 equiv). The

resulting mixture was stirred at 70 °C for 12 h. After cooling to ambient temperature, H_2O (2 mL) was added to the vial, and the mixture was extracted with Et_2O (3 mL x 3). The combined organic layers were concentrated under reduced pressure. The crude reaction product was dissolved in THF (0.5 mL). An aqueous solution of NaOH (3N, 3 mL) was added to the reaction mixture followed by slow addition of 30% H_2O_2 (3 mL) at 0 °C. The reaction was stirred vigorously for 2 h. Then EtOAc (5 mL) and brine (5 mL) was added. The organic layer was separated and the aqueous layer was extracted with EtOAc (5 mL x 3). The combined organic extracts were dried over anhydrous sodium sulfate, filtered, and concentrated under reduced pressure. Purification of the crude product was performed by flash column chromatography (gradient elution with hexane and ethyl acetate, 5:1 to 2:1) to give diol products **5**.

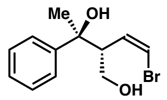

(2*R*,3*S*)-2-((*Z*)-2-bromovinyl)-3-phenylbutane-1,3-diol (5a) Prepared according to the general procedure. The crude mixture was purified by flash column chromatography to give compound **5a** in 66% yield (18 mg, *Z*:*E* = 10:1, dr > 20:1) as colorless oil. Enantiomeric excess was determined by HPLC analysis to be 98% ee (254 nm, 25 °C); t_1 = 8.10 min, t_2 = 9.37 min [(Chiralpak IA) hexane/i-PrOH, 90:10, 1.0 mL/min]; A 5 mmol-scale reaction was conducted with 2.5 mol % CuOAc and 3 mol % (*R,R*)-Ph-BPE, affording **5a** in 75% yield (1.017 g) and 98% ee. ^{1}H NMR (400 MHz, $CDCl_3$) δ 7.40 – 7.42 (m, 2H), 7.32 – 7.36 (m, 2H), 7.24 – 7.28 (m, 1H), 6.23 (d, *J* = 7.2 Hz, 1H), 6.02 (dd, *J* = 9.8, 7.1 Hz, 1H), 3.87 – 3.92 (m, 1H), 3.77 – 3.83 (m, 1H), 3.21 – 3.26 (m, 1H), 3.22 (s, 1H), 2.37 (t, *J* = 5.3 Hz, 1H), 1.70 (s, 3H). ^{13}C NMR (126 MHz, $CDCl_3$) δ 145.7, 132.5, 128.4, 127.3, 125.5, 110.9, 77.6 (*via* HMBC), 63.6, 51.6, 29.6. HRMS (ESI$^+$): m/z for $C_{12}H_{15}BrNa_2O_2$ $[M+2Na-H]^+$ calcd. 314.9967, found: 314.9975.

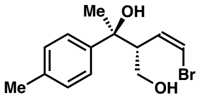

(2*R*,3*S*)-2-((*Z*)-2-bromovinyl)-3-(*p*-tolyl)butane-1,3-diol (5b) Prepared according to the general procedure. The crude mixture was purified by flash column chromatography to give compound **5b** in 81% yield (23 mg, *Z*:*E* = 15:1, dr > 20:1) as colorless oil. Enantiomeric excess was determined by HPLC analysis to be 98% ee (254 nm, 25 °C); t_1 = 7.45 min, t_2 = 9.75 min [(Chiralpak IA) hexane/i-PrOH, 90:10, 1.0 mL/min]; ^{1}H NMR (500 MHz, $CDCl_3$) δ 7.29 (d, *J* = 8.4 Hz, 2H), 7.15 (d, *J* = 7.8 Hz, 2H), 6.24 (d, *J* = 7.2 Hz, 1H), 5.98 (dd, *J* = 9.9, 7.2 Hz, 1H), 3.85 (dd, *J* = 11.1, 4.1 Hz, 1H), 3.78 (dd, *J* = 11.0, 5.9 Hz, 1H), 3.20 – 3.24 (m, 1H), 3.15 (brs, 1H), 2.49 (brs, 1H), 2.34 (s, 3H), 1.67 (s, 3H). ^{13}C NMR (126 MHz, $CDCl_3$) δ 142.7,

137.0, 132.7, 129.1, 125.5, 110.9, 77.6, 63.6, 51.7, 29.7, 21.3. HRMS (ESI$^-$): m/z for $C_{13}H_{17}BrClO_2$ $[M+Cl]^-$ calcd. 319.0106, found: 319.0099.

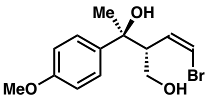

(2*R*,3*S*)-2-((*Z*)-2-bromovinyl)-3-(4-methoxyphenyl)butane-1,3-diol (5c) Prepared according to the general procedure. The crude mixture was purified by flash column chromatography to give compound **5c** in 70% yield (21 mg, *Z*:*E* = 16:1, dr > 20:1) as colorless oil. Enantiomeric excess was determined by HPLC analysis to be 96% ee (254 nm, 25 °C); t_1 = 9.85 min, t_2 = 10.9 min [(Chiralpak ID) hexane/i-PrOH, 90:10, 1.0 mL/min]; $[\alpha]_D^{20}$ = −1.9 (c 1.2, $CHCl_3$); ^{1}H NMR (400 MHz, $CDCl_3$) δ 7.32 (d, *J* = 8.9 Hz, 2H), 6.87 (d, *J* = 8.9 Hz, 2H), 6.25 (d, *J* = 7.2 Hz, 1H), 5.97 (dd, *J* = 9.8, 7.2 Hz, 1H), 3.75 – 3.87 (m, 2H), 3.81 (s, 3H), 3.19 – 3.24 (m, 1H), 3.11 (s, 1H), 2.43 (t, *J* = 5.3 Hz, 1H), 1.67 (s, 3H). ^{13}C NMR (126 MHz, $CDCl_3$) δ 158.8, 137.8, 132.6, 126.8, 113.7, 110.9, 77.4 (*via* HMBC), 63.6, 55.6, 51.8, 29.7. HRMS (ESI$^+$): m/z for $C_{13}H_{17}BrNaO_3$ $[M+Na]^+$ calcd. 323.0253, found: 323.0253.

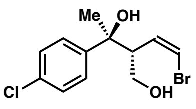

(2*R*,3*S*)-2-((*Z*)-2-bromovinyl)-3-(4-chlorophenyl)butane-1,3-diol (5d) Prepared according to the general procedure. The crude mixture was purified by flash column chromatography to give compound **5d** in 69% yield (21 mg, *Z*:*E* = 10:1, dr > 20:1) as colorless oil. Enantiomeric excess was determined by HPLC analysis to be 98% ee (254 nm, 25 °C); t_1 = 8.31 min, t_2 = 12.1 min [(Chiralpak IA) hexane/i-PrOH, 90:10, 1.0 mL/min]; ^{1}H NMR (500 MHz, $CDCl_3$) δ 7.34 (d, *J* = 8.9 Hz, 2H), 7.30 (d, *J* = 8.9 Hz, 2H), 6.23 (d, *J* = 7.2 Hz, 1H), 6.05 (dd, *J* = 9.8, 7.2 Hz, 1H), 3.96 – 4.00 (m, 1H), 3.79 – 3.84 (m, 1H), 3.50 (s, 1H), 3.15 – 3.19 (m, 1H), 2.22 (t, *J* = 5.1 Hz, 1H), 1.68 (s, 3H). ^{13}C NMR (126 MHz, $CDCl_3$) δ 144.6, 133.1, 132.3, 128.5, 127.0, 111.1, 77.6 (*via* HMBC), 63.7, 51.3, 29.6. HRMS (ESI$^-$): m/z for $C_{13}H_{15}BrClO_4$ $[M+HCOO]^-$ calcd. 348.9848, found: 348.9845.

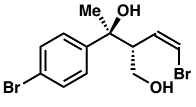

(2*R*,3*S*)-3-(4-bromophenyl)-2-((*Z*)-2-bromovinyl)butane-1,3-diol (5e) Prepared according to the general procedure. The crude mixture was purified by flash column chromatography to give compound **5e** in 57% yield (20 mg, *Z*:*E* = 15:1, dr > 20:1) as a white solid. Enantiomeric excess was determined by HPLC analysis to be 98% ee (254 nm, 25 °C); t_1 = 10.4 min, t_2 = 13.1 min [(Chiralpak ID) hexane/i-PrOH, 95:5, 1.0 mL/min]; A 0.2 mmol-scale reaction was conducted with 5 mol % CuOAc and 6 mol % (*S,S*)-Ph-BPE, affording *ent*-**5e** in 63% yield (44 mg) and 98% ee. ^{1}H NMR (500 MHz, $CDCl_3$) δ 7.45 (d, *J* = 8.7 Hz, 2H), 7.28 (d, *J* = 8.9 Hz, 2H), 6.23 (d, *J* = 7.3 Hz, 1H), 6.05 (dd, *J* = 9.8, 7.2 Hz, 1H), 3.96 – 4.00

(m, 1H), 3.79 – 3.84 (m, 1H), 3.52 (s, 1H), 3.15 – 3.19 (m, 1H), 2.22 (t, *J* = 5.1 Hz, 1H), 1.67 (s, 3H). ^{13}C NMR (126 MHz, $CDCl_3$) δ 145.2, 132.3, 131.4, 127.4, 121.2, 111.1, 77.6, 63.7, 51.3, 29.6. HRMS (ESI$^+$): for $C_{12}H_{13}Br_2Na_2O_2$ [M+2Na–H]$^+$ calcd. 392.9072, found: 392.9067.

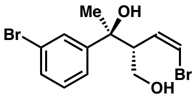

(2*R*,3*S*)-3-(3-bromophenyl)-2-((*Z*)-2-bromovinyl)butane-1,3-diol (5f) Prepared according to the general procedure. The crude mixture was purified by column chromatography to give compound **5f** in 77% yield (27 mg, *Z*:*E* = 7:1, dr > 20:1) as colorless oil. Enantiomeric excess was determined by HPLC analysis to be 98% ee (254 nm, 25 °C); t_1 = 12.0 min, t_2 = 15.6 min [(Chiralpak IA) hexane/i-PrOH, 95:5, 1.0 mL/min]; ^{1}H NMR (400 MHz, $CDCl_3$) δ 7.59 (dd, *J* = 1.9, 1.9 Hz, 1H), 7.36 – 7.39 (m, 1H), 7.30 – 7.32 (m, 1H), 7.20 (dd, *J* = 7.9, 7.9 Hz, 1H), 6.24 (d, *J* = 7.2 Hz, 1H), 6.08 (dd, *J* = 9.8, 7.2 Hz, 1H), 3.97 – 4.02 (m, 1H), 3.80 – 3.85 (m, 1H), 3.54 (s, 1H), 3.15 – 3.20 (m, 1H), 2.21 (t, *J* = 5.1 Hz, 1H), 1.68 (s, 3H). ^{13}C NMR (126 MHz, $CDCl_3$) δ 148.5, 132.1, 130.3, 130.0, 128.8, 124.2, 122.8, 111.1, 77.1 (*via* HMBC), 63.6, 51.3, 29.6. HRMS (ESI$^+$): m/z for $C_{12}H_{13}Br_2Na_2O_2$ [M+2Na–H]$^+$ calcd. 392.9072, found: 392.9068.

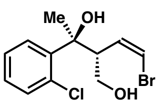

(2*R*,3*S*)-2-((*Z*)-2-bromovinyl)-3-(2-chlorophenyl)butane-1,3-diol (5g) Prepared according to the general procedure. The crude mixture was purified by column chromatography to give compound **5g** in 59% yield (18 mg, *Z*:*E* = 10:1, dr > 20:1) as colorless oil. Enantiomeric excess was determined by HPLC analysis to be 99% ee (254 nm, 25 °C); t_1 = 11.0 min, t_2 = 14.2 min [(Chiralpak IA) hexane/i-PrOH, 95:5, 1.0 mL/min]; A 0.2 mmol-scale reaction was conducted with 5 mol % CuOAc and 6 mol % (*R,R*)-Ph-BPE, affording **5g** in 64% yield (39 mg) and 99% ee. ^{1}H NMR (400 MHz, $CDCl_3$) δ 7.75 (dd, *J* = 7.9, 1.8 Hz, 1H), 7.32 (dd, *J* = 7.8, 1.5 Hz, 1H), 7.23 – 7.27 (m, 1H), 7.15 – 7.19 (m, 1H), 6.29 (dd, *J* = 9.2, 7.1 Hz, 1H), 6.13 (dd, *J* = 7.2, 0.9 Hz, 1H), 4.27 – 4.32 (m, 1H), 3.99 – 4.04 (m, 1H), 3.81 – 3.85 (m, 1H), 3.77 (s, 1H), 2.12 (dd, *J* = 6.0, 4.4 Hz, 1H), 1.92 (s, 3H). ^{13}C NMR (126 MHz, $CDCl_3$) δ 143.3, 132.9, 131.6, 131.0, 128.7, 128.3, 127.0, 110.2, 77.4 (*via* HMBC), 63.7, 47.1, 27.0. HRMS (ESI$^+$): m/z for $C_{12}H_{15}BrClO_2$ [M+H]$^+$ calcd. 304.9938, found: 304.9949.

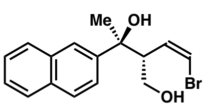

(2*R*,3*S*)-2-((*Z*)-2-bromovinyl)-3-(naphthalen-2-yl)butane-1,3-diol (5h) Prepared according to the general procedure. The crude mixture was purified by column chromatography to give compound **5h** in 81% yield (26 mg, *Z*:*E* = 14:1, dr > 20:1) as colorless oil. Enantiomeric excess was determined

by HPLC analysis to be 98% ee (254 nm, 25 °C); t_1 = 8.89 min, t_2 = 12.3 min [(Chiralpak IA) hexane/i-PrOH, 90:10, 1.0 mL/min]; ^{1}H NMR (500 MHz, $CDCl_3$) δ 7.90 (d, *J* = 1.9 Hz, 1H), 7.81 – 7.86 (m, 3H), 7.45 – 7.50 (m, 3H), 6.19 (d, *J* = 7.2 Hz, 1H), 6.09 (dd, *J* = 9.8, 7.2 Hz, 1H), 3.97 – 4.00 (m, 1H), 3.85 – 3.89 (m, 1H), 3.49 (s, 1H), 3.31 – 3.35 (m, 1H), 2.43 (t, *J* = 5.2 Hz, 1H), 1.79 (s, 3H). ^{13}C NMR (126 MHz, $CDCl_3$) δ 143.4, 133.3, 132.7, 132.6, 128.6, 128.1, 127.8, 126.5, 126.2, 124.1, 123.9, 110.9, 77.6 (*via* HMBC), 63.7, 51.5, 29.7. HRMS (ESI$^+$): m/z for $C_{16}H_{17}BrNaO_2$ $[M+Na]^+$ calcd. 343.0304, found: 343.0310.

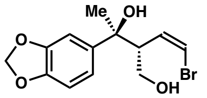

(2*R*,3*S*)-3-(benzo[*d*][1,3]dioxol-5-yl)-2-((*Z*)-2-bromovinyl)butane-1,3-diol (5i) Prepared according to the general procedure. The crude mixture was purified by column chromatography to give compound **5i** in 86% yield (27 mg, *Z*:*E* > 20:1, dr > 20:1) as colorless oil. Enantiomeric excess was determined by HPLC analysis to be 99% ee (254 nm, 25 °C); t_1 = 13.3 min, t_2 = 14.4 min [(Chiralpak IA) hexane/i-PrOH, 90:10, 1.0 mL/min]; ^{1}H NMR (500 MHz, $CDCl_3$) δ 6.92 (d, *J* = 1.8 Hz, 1H), 6.86 (dd, *J* = 8.2, 1.8 Hz, 1H), 6.77 (d, *J* = 8.1 Hz, 1H), 6.25 (d, *J* = 7.3 Hz, 1H), 6.02 (dd, *J* = 9.8, 7.2 Hz, 1H), 5.95 (s, 2H), 3.86 – 3.90 (m, 1H), 3.77 – 3.82 (m, 1H), 3.30 (s, 1H), 3.14 – 3.18 (m, 1H), 2.41 (t, *J* = 5.3 Hz, 1H), 1.65 (s, 3H). ^{13}C NMR (126 MHz, $CDCl_3$) δ 147.8, 146.7, 140.1, 132.6, 118.7, 110.9, 108.1, 106.6, 101.4, 77.4 (*via* HMBC), 63.6, 51.8, 29.8. HRMS (ESI$^+$): m/z for $C_{13}H_{15}BrNaO_4$ $[M+Na]^+$ calcd. 337.0046, found: 337.0040.

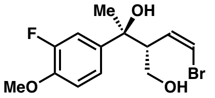

(2*R*,3*S*)-2-((*Z*)-2-bromovinyl)-3-(3-fluoro-4-methoxyphenyl)butane-1,3-diol (5j) Prepared according to the general procedure. The crude mixture was purified by column chromatography to give compound **5j** in 69% yield (22 mg, *Z*:*E* = 17:1, dr > 20:1) as colorless oil. Enantiomeric excess was determined by HPLC analysis to be 98% ee (254 nm, 25 °C); t_1 = 20.1 min, t_2 = 21.7 min [(Chiralpak ID) hexane/i-PrOH, 95:5, 1.0 mL/min]; ^{1}H NMR (500 MHz, $CDCl_3$) δ 7.14 – 7.17 (m, 1H), 7.08 – 7.10 (m, 1H), 6.91 (dd, *J* = 8.6, 8.6 Hz, 1H), 6.25 (d, *J* = 7.2 Hz, 1H), 6.03 (dd, *J* = 9.8, 7.2 Hz, 1H), 3.88 – 3.94 (m, 1H), 3.88 (s, 3H), 3.77 – 3.81 (m, 1H), 3.47 (s, 1H), 3.13 – 3.17 (m, 1H), 2.35 (t, *J* = 5.1 Hz, 1H), 1.66 (s, 3H). ^{13}C NMR (151 MHz, $CDCl_3$) δ 152.2 (d, *J* = 244.9 Hz), 146.6 (d, *J* = 10.8 Hz), 139.2 (d, *J* = 5.0 Hz), 132.4, 121.2 (d, *J* = 3.3 Hz), 113.8 (d, *J* = 19.3 Hz), 113.1 (d, *J* = 2.2 Hz), 111.0, 76.9, 63.6, 56.6, 51.5, 29.6. HRMS (ESI$^+$): for $C_{13}H_{15}BrFNa_2O_3$ $[M+2Na-H]^+$ calcd. 362.9979, found: 362.9977.

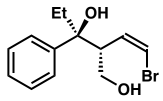

(2*R*,3*S*)-2-((*Z*)-2-bromovinyl)-3-phenylpentane-1,3-diol (5k) Prepared according to the general procedure. The crude mixture was purified by column chromatography to give compound **5k** in 67% yield (19 mg, *Z*:*E* = 7:1, dr > 20:1) as colorless oil. Enantiomeric excess was determined by HPLC analysis to be 96% ee (254 nm, 25 °C); t_1 = 12.8 min, t_2 = 15.1 min [(Chiralpak IA) hexane/i-PrOH, 95:5, 1.0 mL/min]; ^{1}H NMR (500 MHz, $CDCl_3$) δ 7.32 – 7.37 (m, 4H), 7.23 – 7.26 (m, 1H), 6.21 (d, *J* = 7.0 Hz, 1H), 6.01 (dd, *J* = 9.8, 7.2 Hz, 1H), 3.85 – 3.89 (m, 1H), 3.76 – 3.81 (m, 1H), 3.23 – 3.27 (m, 1H), 3.25 (s, 1H), 2.39 (t, *J* = 5.3 Hz, 1H), 2.02 – 2.07 (m, 2H), 0.75 (t, *J* = 7.4 Hz, 3H). ^{13}C NMR (126 MHz, $CDCl_3$) δ 143.4, 132.6, 128.4, 127.2, 126.2, 110.6, 80.1, 63.5, 51.1, 33.4, 7.8. HRMS (ESI$^+$): for $C_{13}H_{16}BrNa_2O_2$ $[M+2Na-H]^+$ calcd. 329.0124, found: 329.0114.

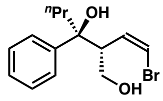

(2*R*,3*S*)-2-((*Z*)-2-bromovinyl)-3-phenylhexane-1,3-diol (5l) Prepared according to the general procedure. The crude mixture was purified by column chromatography to give compound **5l** in 84% yield (25 mg, *Z*:*E* = 8:1, dr > 20:1) as colorless oil. Enantiomeric excess was determined by HPLC analysis to be 97% ee (254 nm, 25 °C); t_1 = 11.6 min, t_2 = 13.1 min [(Chiralpak IA) hexane/i-PrOH, 95:5, 1.0 mL/min]; ^{1}H NMR (600 MHz, $CDCl_3$) δ 7.32 – 7.36 (m, 4H), 7.23 – 7.26 (m, 1H), 6.20 (d, *J* = 7.2 Hz, 1H), 6.03 (dd, *J* = 9.9, 7.2 Hz, 1H), 3.89 – 3.92 (m, 1H), 3.76 – 3.80 (m, 1H), 3.30 (s, 1H), 3.22 – 3.26 (m, 1H), 2.41 (t, *J* = 5.3 Hz, 1H), 1.93 – 2.01 (m, 2H), 1.27 – 1.36 (m, 1H), 0.95 – 1.03 (m, 1H), 0.87 (t, *J* = 7.3 Hz, 3H). ^{13}C NMR (126 MHz, $CDCl_3$) δ 143.9, 132.6, 128.4, 127.1, 126.0, 110.6, 79.9, 63.5, 51.2, 43.3, 16.8, 14.8. HRMS (ESI$^+$): m/z for $C_{14}H_{19}BrNaO_2$ $[M+Na]^+$ calcd. 321.0461, found: 321.0465.

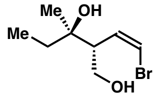

(2*R*,3*R*)-2-((*Z*)-2-bromovinyl)-3-methylpentane-1,3-diol (5m) Prepared according to the general procedure. The crude mixture was purified by column chromatography to give compound **5m** as colorless oil in 81% yield (18 mg, *Z*:*E* = 10:1, dr = 10:1). Enantiomeric excess was determined by HPLC analysis to be 99% ee (254 nm, 25 °C) t_1 = 8.99 min, t_2 = 15.2 min [(Chiralpak ID) hexane/i-PrOH, 95:5, 1.0 mL/min]; A 0.2 mmol-scale reaction was conducted with 5 mol % CuOAc and 6 mol % (*R,R*)-Ph-BPE, affording **5m** in 78% yield (35 mg) and 99% ee. ^{1}H NMR (600 MHz, $CDCl_3$) δ 6.37 – 6.40 (m, 2H), 4.02 (ddd, *J* = 10.9, 4.1, 4.1 Hz, 1H), 3.85 (ddd, *J* = 10.9, 6.3, 4.6 Hz, 1H), 2.80 – 2.84 (m, 1H), 2.39 (s, 1H), 2.37 (dd, *J* = 6.3, 4.2 Hz, 1H), 1.52 (m, 2H), 1.30 (s, 3H), 0.91 (t, *J* = 7.5 Hz, 3H). ^{13}C NMR (126 MHz, $CDCl_3$) δ 133.3, 110.5, 75.9, 63.8, 49.5, 33.4, 25.3, 8.4. HRMS (ESI$^+$): m/z for $C_8H_{15}BrNaO_2$ $[M+Na]^+$ calcd. 245.0148, found: 245.0136.

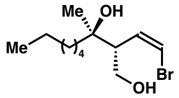

(2*R*,3*R*)-2-((*Z*)-2-bromovinyl)-3-methylnonane-1,3-diol (5n) Prepared according to the general procedure. The crude mixture was purified by column chromatography to give compound **5n** in 72% yield (20 mg, *Z*:*E* = 18:1, dr > 20:1) as colorless oil. Enantiomeric excess was determined by HPLC analysis to be > 99% ee (254 nm, 25 °C); t_1 = 6.62 min, t_2 = 9.30 min [(Chiralpak ID) hexane/i-PrOH, 95:5, 1.0 mL/min]; ^{1}H NMR (500 MHz, $CDCl_3$) δ 6.35 – 6.40 (m, 2H), 3.99 – 4.03 (m, 1H), 3.81 – 3.86 (m, 1H), 2.78 – 2.84 (m, 1H), 2.41 – 2.45 (m, 2H), 1.44 – 1.47 (m, 2H), 1.25 – 1.32 (m, 11H), 0.86 – 0.89 (m, 3H). ^{13}C NMR (151 MHz, $CDCl_3$) δ 133.3, 110.5, 75.7, 63.8, 49.7, 40.9, 32.1, 30.1, 26.0, 23.9, 22.9, 14.4. HRMS (ESI$^+$): m/z for $C_{12}H_{22}BrNa_2O_2$ $[M+2Na-H]^+$ calcd. 323.0593, found: 323.0595.

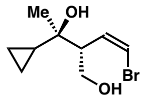

(2*R*,3*R*)-2-((*Z*)-2-bromovinyl)-3-cyclopropylbutane-1,3-diol (5o) Prepared according to the general procedure. The crude mixture was purified by column chromatography to give compound **5o** in 77% yield (18 mg, *Z*:*E* > 20:1, dr > 20:1) as colorless oil. Enantiomeric excess was determined by HPLC analysis to be > 99% ee (254 nm, 25 °C); t_1 = 6.02 min, t_2 = 7.75 min [(Chiralpak ID) hexane/i-PrOH, 90:10, 1.0 mL/min]; ^{1}H NMR (500 MHz, $CDCl_3$) δ 6.38 (d, *J* = 7.2 Hz, 1H), 6.22 (dd, *J* = 10.0, 7.2 Hz, 1H), 3.99 – 4.03 (m, 1H), 3.82 – 3.86 (m, 1H), 3.05 – 3.09 (m, 1H), 2.48 (t, *J* = 5.3 Hz, 1H), 2.38 (s, 1H), 1.17 (s, 3H), 0.93 – 0.98 (m, 1H), 0.53 – 0.58 (m, 1H), 0.37 – 0.47 (m, 2H), 0.27 – 0.32 (m, 1H). ^{13}C NMR (126 MHz, $CDCl_3$) δ 132.8, 110.9, 74.3, 63.6, 52.4, 26.3, 18.5, 1.3, 0.6. HRMS (ESI$^+$): m/z for $C_9H_{14}BrNa_2O_2$ $[M+2Na-H]^+$ calcd. 278.9967, found: 278.9960.

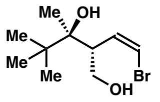

(2*R*,3*S*)-2-((*Z*)-2-bromovinyl)-3,4,4-trimethylpentane-1,3-diol (5p) Prepared according to the general procedure. The crude mixture was purified by column chromatography to give compound **5p** in 52% yield (13 mg, *Z*:*E* > 20:1, dr > 20:1) as colorless oil. Enantiomeric excess was determined by HPLC analysis to be > 99% ee (254 nm, 25 °C); t_1 = 5.52 min, t_2 = 6.25 min [(Chiralpak IA) hexane/i-PrOH, 90:10, 1.0 mL/min]; ^{1}H NMR (500 MHz, $CDCl_3$) δ 6.72 (dd, *J* = 9.6, 7.1 Hz, 1H), 6.28 (dd, *J* = 7.0, 0.8 Hz, 1H), 4.04 – 4.08 (m, 1H), 3.77 – 3.81 (m, 1H), 2.96 – 3.00 (m, 1H), 2.21 (s, 1H), 2.01 – 2.05 (m, 1H), 1.33 (s, 3H), 0.98 (s, 9H). ^{13}C NMR (126 MHz, $CDCl_3$) δ 136.9, 108.0, 78.6, 66.0, 47.0, 39.5, 26.9, 22.7. HRMS (ESI$^+$): m/z for $C_{10}H_{18}BrNa_2O_2$ $[M+2Na-H]^+$ calcd. 295.0280, found: 295.0288.

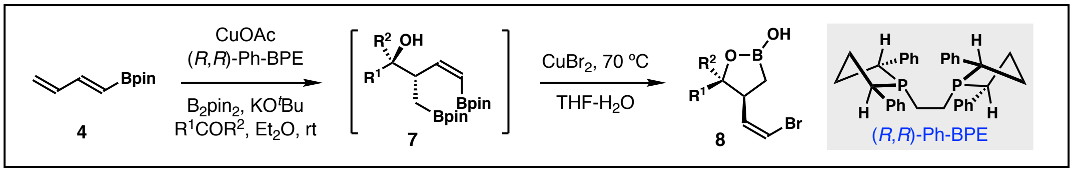

General procedure for the syntheses of compounds 8: In an N_2-filled glove box, CuOAc (1 mg, 0.01 mmol, 10 mol %), (*R,R*)-Ph-BPE (6 mg, 0.012 mmol, 12 mol %), KO*^t^*Bu (17 mg, 0.15 mmol, 1.5 equiv), Et_2O (1.0 mL), and a Teflon-coated magnetic stirring bar were sequentially added into a reaction vial. The resulting mixture was stirred at ambient temperature for 15 min. B_2Pin_2 (38 mg, 0.15 mmol, 1.5 equiv) was added and the mixture was stirred for 10 min. Then the solution of dienylboronate **4** (18 mg, 0.10 mmol, 1.0 equiv) and ketone (0.15 mmol, 1.5 equiv) in Et_2O (0.5 mL) were added to the reaction mixture in one portion. The resulting reaction mixture was stirred at ambient temperature inside the glove box and the reaction progress was monitored by ^{1}H NMR analysis. After complete consumption of dienylboronate **4**, the reaction mixture was filtered through a short pad of silica gel and Celite. The filtrate was concentrated under reduced pressure. Then THF (0.5 mL) and H_2O (0.5 mL) were added to the crude reaction product **7** followed by addition of $CuBr_2$ (67 mg, 0.3 mmol, 3.0 equiv). The resulting mixture was stirred at 70 °C for 12 h. After cooling to ambient temperature, H_2O (2 mL) was added to the vial, and the mixture was extracted with Et_2O (2 mL x 3). The combined organic layers were dried over anhydrous Na_2SO_4, filtered, and concentrated under reduced pressure. Purification of the crude product was performed by flash column chromatography (gradient elution with hexane and ethyl acetate, 10:1 to 5:1) to give products **8**.

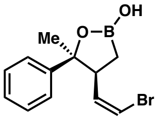

(4*S*,5*S*)-4-((*Z*)-2-bromovinyl)-5-methyl-5-phenyl-1,2-oxaborolan-2-ol (8a) Prepared according to the general procedure. The crude mixture was purified by flash column chromatography to give compound **8a** in 75% yield (21 mg, *Z*:*E* = 10:1, dr > 20:1) as colorless oil. Enantiomeric excess was determined by HPLC analysis of diol **5a** to be 98% ee (254 nm, 25 °C); A 0.2 mmol-scale reaction was conducted with 5 mol % CuOAc and 6 mol % (*R,R*)-Ph-BPE, affording **8a** in 71% yield (40 mg) with 98% ee. ^{1}H NMR (500 MHz, $CDCl_3$) δ 7.30 – 7.34 (m, 2H), 7.22 – 7.27 (m, 3H), 5.99 (d, *J* = 7.0 Hz, 1H), 5.43 (dd, *J* = 9.9, 7.0 Hz, 1H), 4.52 (s, 1H), 3.51 – 3.56 (m, 1H), 1.70 (s, 3H), 1.44 (dd, *J* = 17.0, 8.2 Hz, 1H), 0.86 (dd, *J* = 17.0, 7.7 Hz, 1H). ^{13}C NMR (126 MHz, $CDCl_3$) δ 143.7, 137.1, 128.3, 127.3, 125.6, 107.8, 87.7, 50.0, 30.1.

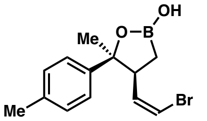

(4*S*,5*S*)-4-((*Z*)-2-bromovinyl)-5-methyl-5-(*p*-tolyl)-1,2-oxaborolan-2-ol (8b) Prepared according to the general procedure. The crude mixture was purified by flash column chromatography to give compound **8b** in 85% yield (25 mg, *Z*:*E* = 15:1, dr > 20:1) as colorless oil. Enantiomeric excess was determined by HPLC analysis of diol **5b** to be 98% ee (254 nm, 25 °C); ^{1}H NMR (500 MHz, $CDCl_3$) δ 7.11 – 7.15 (m, 4H), 6.00 (d, *J* = 7.0 Hz, 1H), 5.43 (dd, *J* = 9.8, 7.0 Hz, 1H), 4.56 (s, 1H), 3.48 – 3.53 (m, 1H), 2.33 (s, 3H), 1.68 (s, 3H), 1.41 (dd, *J* = 16.9, 8.1 Hz, 1H), 0.85 (dd, *J* = 16.9, 7.9 Hz, 1H). ^{13}C NMR (126 MHz, $CDCl_3$) δ 140.7, 137.2, 136.9, 129.0, 125.5, 107.7, 87.6, 50.0, 30.2, 21.3.

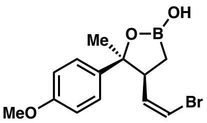

(4*S*,5*S*)-4-((*Z*)-2-bromovinyl)-5-(4-methoxyphenyl)-5-methyl-1,2-oxaborolan-2-ol (8c) Prepared according to the general procedure. The crude mixture was purified by flash column chromatography to give compound **8c** in 77% yield (24 mg, *Z*:*E* = 16:1, dr > 20:1) as colorless oil. Enantiomeric excess was determined by HPLC analysis of diol **5c** to be 96% ee (254 nm, 25 °C); ^{1}H NMR (600 MHz, $CDCl_3$) δ 7.17 (d, *J* = 8.9 Hz, 2H), 6.85 (d, *J* = 8.9 Hz, 2H), 6.00 (d, *J* = 7.0 Hz, 1H), 5.43 (dd, *J* = 9.9, 7.0 Hz, 1H), 4.53 (s, 1H), 3.80 (s, 3H), 3.47 – 3.52 (m, 1H), 1.67 (s, 3H), 1.41 (dd, *J* = 17.0, 8.2 Hz, 1H), 0.85 (dd, *J* = 17.0, 8.0 Hz, 1H). ^{13}C NMR (126 MHz, $CDCl_3$) δ 158.8, 137.2, 135.8, 126.8, 113.5, 107.7, 87.5, 55.6, 50.1, 30.2.

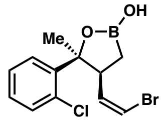

(4*S*,5*S*)-4-((*Z*)-2-bromovinyl)-5-(2-chlorophenyl)-5-methyl-1,2-oxaborolan-2-ol (8g) Prepared according to the general procedure. The crude mixture was purified by column chromatography to give compound **8g** in 63% yield (20 mg, *Z*:*E* = 10:1, dr > 20:1) as colorless oil. Enantiomeric excess was determined by HPLC analysis of diol **5g** to be 99% ee (254 nm, 25 °C); ^{1}H NMR (600 MHz, $CDCl_3$) δ 7.81 (dd, *J* = 7.9, 1.8 Hz, 1H), 7.30 (dd, *J* = 7.8, 1.4 Hz, 1H), 7.23 – 7.26 (m, 1H), 7.17 – 7.20 (m, 1H), 5.89 (dd, *J* = 7.1, 1.0 Hz, 1H), 5.61 (dd, *J* = 9.4, 7.1 Hz, 1H), 4.42 (s, 1H), 3.99 (dd, *J* = 9.1, 9.1 Hz, 1H), 1.77 (dd, *J* = 17.2, 9.0 Hz, 1H), 1.70 (s, 3H), 0.90 (d, *J* = 17.2 Hz, 1H). ^{13}C NMR (126 MHz, $CDCl_3$) δ 142.3, 136.5, 131.4, 131.0, 128.7, 127.9, 127.0, 107.8, 87.9, 47.3, 26.9.

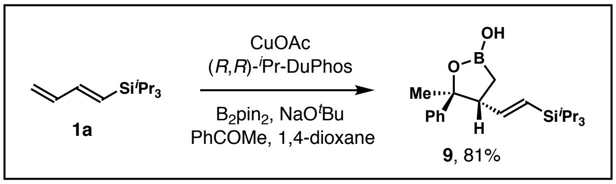

(4*R*,5*S*)-5-methyl-5-phenyl-4-((*E*)-2-(triisopropylsilyl)vinyl)-1,2-oxaborolan-2-ol (9): In an N_2-filled glove box, CuOAc (1 mg, 0.01 mmol, 10 mol %), (*R,R*)-^{i}Pr-DuPhos (5 mg, 0.012 mmol, 12 mol %), NaO^{t}Bu (14 mg, 0.15 mmol, 1.5 equiv), 1,4-dioxane (1.0 mL), and a Teflon-coated magnetic stirring bar were sequentially added into a reaction vial. The resulting mixture was stirred at ambient temperature for 15 min. B_2Pin_2 (38 mg, 0.15 mmol, 1.5 equiv) was added, and the mixture was stirred for 10 min. Then the solution of dienylsilane **1a** (21 mg, 0.10 mmol, 1.0 equiv) and acetophenone (18 mg, 0.15 mmol, 1.5 equiv) in 1,4-dioxane (0.5 mL) were added in one portion to the reaction vial. The reaction mixture was stirred at ambient temperature inside the glove box and the reaction progress was monitored by ^{1}H NMR analysis. After complete consumption of diene **1a**, the reaction mixture was filtered through a short pad of silica gel and Celite. The filtrate was concentrated under reduced pressure. The crude product was purified by flash column chromatography (gradient elution with hexane and ethyl acetate, 10:1 to 5:1) to give compound **9** as colorless oil in 81% yield (29 mg). ^{1}H NMR (600 MHz, $CDCl_3$) δ 7.43 (d, J = 8.5 Hz, 2H), 7.32 (d, J = 7.7 Hz, 2H), 7.23 – 7.28 (m, 1H), 6.14 (dd, J = 18.8, 7.8 Hz, 1H), 5.51 (d, J = 18.8 Hz, 1H), 4.66 (s, 1H), 2.87 – 2.91 (m, 1H), 1.22 (dd, J = 16.9, 8.1 Hz, 1H), 1.16 (dd, J = 16.9, 10.4 Hz, 1H), 1.05 – 1.14 (m, 21H). ^{13}C NMR (151 MHz, $CDCl_3$) δ 148.7, 147.9, 128.4, 127.1, 126.6, 124.7, 87.2, 57.9, 24.8, 19.00, 18.98, 11.3. HRMS (ESI$^+$): m/z for $C_{21}H_{34}BNa_2O_2Si$ $[M+2Na-H]^+$ calcd. 403.2211, found: 403.2212.

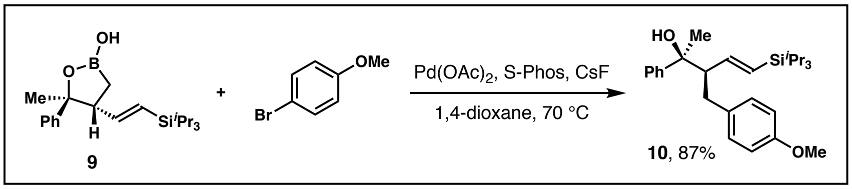

(2*S*,3*R*,*E*)-3-(4-methoxybenzyl)-2-phenyl-5-(triisopropylsilyl)pent-4-en-2-ol (10): A Teflon-coated magnetic stirring bar, $Pd(OAc)_2$ (1 mg, 0.005 mmol, 10 mol %), S-Phos (3 mg, 0.075 mmol, 15 mol %), CsF (23 mg, 0.15 mmol, 3.0 equiv) and 1,4-dioxane (0.5 mL) were added to an N_2-filled reaction vial. Then compound **9** (18 mg, 0.05 mmol, 1.0 equiv) and 4-bromoanisole (19 mg, 0.1 mmol, 2.0 equiv) were added to the reaction vial.

Then reaction mixture was kept stirring at 70 °C for 12 h. After complete consumption of **9**, the reaction mixture was diluted with diethyl ether (1.0 mL). The resulting mixture was filtered through a short pad of silica gel and Celite. The filtrate was concentrated under reduced pressure. The crude product was purified by column chromatography (gradient elution with hexane and ethyl acetate, 20:1 to 10:1) to give compound **10** as colorless oil in 87% yield (19 mg). ^{1}H NMR (500 MHz, $CDCl_3$) δ 7.48 (d, *J* = 7.2 Hz, 2H), 7.37 (dd, *J* = 7.8, 7.8 Hz, 2H), 7.24 – 7.28 (m, 1H), 6.86 (d, *J* = 8.5 Hz, 2H), 6.69 (d, *J* = 8.7 Hz, 2H), 5.90 (dd, *J* = 18.9, 8.7 Hz, 1H), 5.29 (d, *J* = 18.9 Hz, 1H), 3.72 (s, 3H), 2.61 – 2.66 (m, 2H), 2.40 (dd, *J* = 14.1, 11.7 Hz, 1H), 1.89 (brs, 1H), 1.59 (s, 3H), 0.90 – 0.99 (m, 3H), 0.92 (dd, *J* = 6.6, 3.5 Hz, 18H). ^{13}C NMR (126 MHz, $CDCl_3$) δ 157.9, 147.7, 147.4, 133.3, 130.3, 130.1, 128.5, 126.9, 125.5, 113.8, 76.5, 61.9, 55.6, 35.0, 29.7, 18.93, 18.89, 11.2. HRMS (ESI$^+$): m/z for $C_{28}H_{43}O_2Si$ [M+H]$^+$ calcd. 439.3027, found: 439.3047.

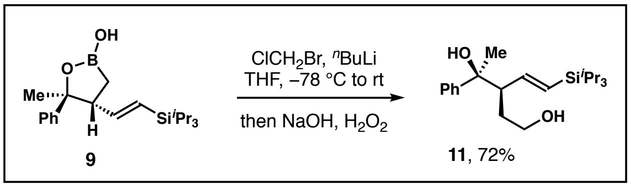

(3*R*,4*S*)-4-phenyl-3-((*E*)-2-(triisopropylsilyl)vinyl)pentane-1,4-diol (11): To a solution of compound **9** (36 mg, 0.1 mmol, 1.0 equiv) in THF (0.5 mL) was added $ClCH_2Br$ (16 µL, 0.25 mmol, 2.5 equiv) under argon. The resulting colorless solution was placed in an acetone-dry ice bath and cooled to −78 °C. After stirring for 10 min, *n*-BuLi (2.5 M in THF, 0.4 mL, 1.0 mmol, 10.0 equiv) was added slowly to the reaction vial at −78 °C. After stirring at −78 °C for 20 min, the cooling bath was removed. The reaction mixture was allowed to warm to ambient temperature and stirred for additional 6 h. Then the resulting mixture was cooled to 0 °C with an ice bath. Water (1.0 mL) and ethyl acetate (1.0 mL) were slowly added. The organic layer was separated and the aqueous layer was extracted with ethyl acetate (3 x 1 mL). The combined organic extracts were dried over anhydrous sodium sulfate, filtered, and concentrated under reduced pressure. The crude product was dissolved in Et_2O (1.0 mL), An aqueous solution of NaOH (3N, 3 mL) was added to the reaction mixture followed by slow addition of 30% H_2O_2 (3 mL) at 0 °C. The reaction was stirred vigorously for 2 h. Then EtOAc (5 mL) and brine (5 mL) was added. The organic layer was separated and the aqueous layer was extracted with EtOAc (5 mL x 3). The combined organic layers were dried over anhydrous magnesium sulfate, filtered, and concentrated under reduced pressure. The crude mixture was purified by flash column chromatography (gradient elution with hexane and ethyl acetate, 10:1 to 5:1)

to give compound **11** as colorless oil in 72% yield (26 mg). ^{1}H NMR (600 MHz, C_6D_6) δ 7.37 (d, *J* = 8.3 Hz, 2H), 7.22 (dd, *J* = 7.7, 7.7 Hz, 2H), 7.10 (dd, *J* = 7.9, 7.9 Hz, 1H), 6.14 (dd, *J* = 19.0, 8.9 Hz, 1H), 5.63 (d, *J* = 19.0 Hz, 1H), 3.40 – 3.44 (m, 1H), 3.27 – 3.31 (m, 1H), 2.56 – 2.59 (m, 1H), 1.93 (brs, 1H), 1.45 – 1.48 (m, 2H), 1.42 (s, 3H), 1.05 – 1.11 (m, 21H). ^{13}C NMR (151 MHz, $CDCl_3$) δ 148.4, 147.7, 129.5, 128.4, 126.9, 125.4, 76.2, 61.6, 56.2, 32.4, 29.4, 19.04, 19.02, 11.2. HRMS (ESI$^+$): m/z for $C_{22}H_{38}O_2SiNa$ $[M+Na]^+$ calcd. 385.2533, found: 385.2516.

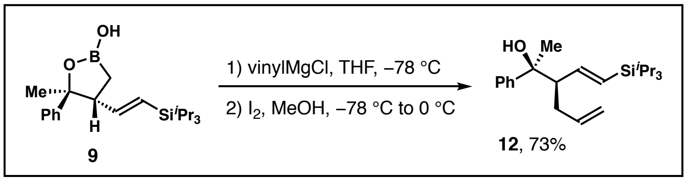

(2*S*,3*R*)-2-phenyl-3-((*E*)-2-(triisopropylsilyl)vinyl)hex-5-en-2-ol (12): To a solution of compound **9** (18 mg, 0.05 mmol, 1.0 equiv) in THF (0.5 mL) was added vinylmagnesium bromide solution (0.30 mL, 1.0 M in THF, 0.30 mmol, 6.0 equiv) at –78 °C. After stirring at –78 °C for 30 min, a solution of iodine (76 mg, 0.30 mmol, 6.0 equiv) in MeOH (0.5 mL) was added. The resulting mixture was kept stirring for 30 min at –78 °C. The reaction mixture was allowed to warm to 0 °C and stirred for additional 6 h. Then an aqueous solution of sat. $Na_2S_2O_3$ (5 mL) and Et_2O (5 mL) were added. The resulting mixture was stirred at ambient temperature for 30 min. The organic layer of the mixture was separated, and the aqueous layer was extracted with Et_2O (3 x 2 mL). The combined organic extracts were dried over anhydrous sodium sulfate, filtered, and concentrated under reduced pressure. The crude mixture was purified by flash column chromatography (gradient elution with hexane and ethyl acetate, 20:1 to 10:1) to give compound **12** as colorless oil in 73% yield (13 mg). ^{1}H NMR (600 MHz, $CDCl_3$) δ 7.40 (d, *J* = 8.3 Hz, 2H), 7.34 (dd, *J* = 7.7, 7.7 Hz, 2H), 7.23 (dd, *J* = 7.3, 7.3 Hz, 1H), 5.90 (dd, *J* = 18.9, 8.8 Hz, 1H), 5.58 – 5.65 (m, 1H), 5.57 (d, *J* = 18.8 Hz, 1H), 4.84 – 4.88 (m, 2H), 2.45 – 2.49 (m, 1H), 2.07 – 2.11 (m, 1H), 1.99 – 2.04 (m, 1H), 1.90 (s, 1H), 1.54 (s, 3H), 1.02 – 1.11 (m, 21H). ^{13}C NMR (151 MHz, $CDCl_3$) δ 148.1, 147.5, 137.8, 129.4, 128.4, 126.9, 125.5, 115.9, 76.4, 59.2, 34.0, 29.3, 19.02, 18.98, 11.2. HRMS (ESI$^+$): m/z for $C_{23}H_{39}OSi$ $[M+H]^+$ calcd. 359.2765, found: 359.2776.

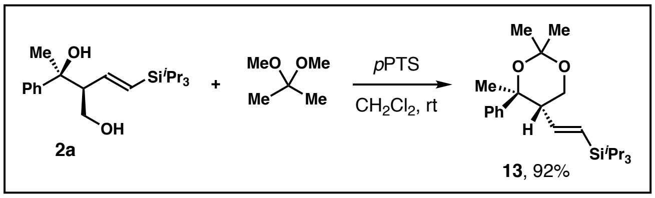

triisopropyl((*E*)-2-((4*S*,5*S*)-2,2,4-trimethyl-4-phenyl-1,3-dioxan-5-yl) vinyl)silane (13) To a solution of diol **2a** (29 mg, 0.1 mmol) in 2, 2-dimethoxypropane (1.0 mL) was added *p*PTS (2 mg) and acetone (0.2 mL). The reaction mixture was kept stirring at ambient temperature, and the progress was monitored by TLC analysis. After complete consumption of diol **2a**, the reaction mixture was filtered through a short pad of silica gel, and the filtrate was concentrated under reduced pressure. Purification of crude product was performed by flash column chromatography (gradient elution with hexane and ethyl acetate) to afford acetonide **13** in 92% yield (36 mg) as a colorless oil. ^{1}H NMR (500 MHz, $CDCl_3$) δ 7.48 – 7.50 (m, 2H), 7.29 – 7.32 (m, 2H), 7.20 – 7.23 (m, 1H), 6.08 (dd, *J* = 19.0, 8.3 Hz, 1H), 5.45 (d, *J* = 19.1 Hz, 1H), 3.92 – 3.93 (m, 2H), 2.79 – 2.83 (m, 1H), 1.59 (s, 3H), 1.53 (s, 3H), 1.34 (s, 3H), 1.00 – 1.08 (m, 21H). ^{13}C NMR (126 MHz, $CDCl_3$) δ 147.7, 145.5, 128.9, 128.2, 127.1, 126.2, 99.2, 77.3, 62.3, 51.1, 29.0, 28.1, 26.2, 19.0, 18.9, 11.2. HRMS (ESI$^+$): m/z for $C_{24}H_{40}O_2NaSi$ $[M+Na]^+$ calcd. 411.2690, found: 411.2689.

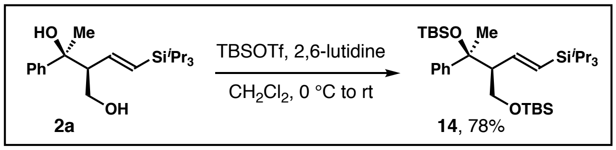

(5*S*,6*S*)-2,2,3,3,5,9,9,10,10-nonamethyl-5-phenyl-6-((*E*)-2-(triisopropylsilyl)vinyl)-4,8 -dioxa-3,9-disilaundecane (14): To a solution of diol **2a** (35 mg, 0.1 mmol, 1.0 equiv) in dry CH_2Cl_2 (2.0 mL), 2,6-lutidine (41 μL, 0.35 mmol, 3.5 equiv) and TBSOTf (69 μL, 0.3 mmol, 3.0 equiv) were added sequentially at −78 °C. After complete consumption of diol **2a**, the resulting mixture was filtered through a pad of silica gel. The filtrate was concentrated under reduced pressure. The crude mixture was purified by flash column chromatography (gradient elution with hexane and ethyl acetate, 100:1 to 50:1) to give compound **14** as colorless oil in 78% yield (45 mg). ^{1}H NMR (600 MHz, $CDCl_3$) δ 7.36 (d, *J* = 8.1 Hz, 2H), 7.25 – 7.28 (m, 2H), 7.17 (dd, *J* = 7.3, 7.3 Hz, 1H), 5.94 (dd, *J* = 18.9, 8.6 Hz, 1H), 5.39 (d, *J* = 18.9 Hz, 1H), 3.72 (dd, *J* = 10.0, 8.6 Hz, 1H), 3.66 (dd, *J* = 10.0, 3.5 Hz, 1H), 2.43 – 2.46 (m, 1H), 1.59 (s, 3H), 0.96 – 1.02 (m, 30H), 0.80 (s, 9H), 0.04 (s, 3H), -0.10 (s, 3H), -0.10 (s, 3H), -0.18 (s, 3H). ^{13}C NMR (151 MHz, $CDCl_3$) δ 148.2,

147.5, 128.7, 127.9, 126.7, 126.1, 78.8, 64.0, 63.2, 26.9, 26.5, 26.3, 19.04, 19.02 (two overlapping carbon signals), 18.6, 11.3, -1.2, -2.2, -5.0, -5.1. HRMS (ESI$^+$): m/z for $C_{33}H_{64}O_2Si_3Na$ $[M+Na]^+$ calcd. 599.4106, found: 599.4105.

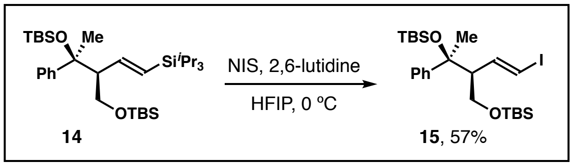

(5*S*,6*S*)-6-((*E*)-2-iodovinyl)-2,2,3,3,5,9,9,10,10-nonamethyl-5-phenyl-4,8-dioxa-3,9-disilaundecane (15): 2,6-Lutidine (14 mg, 0.13 mmol, 1.3 equiv) was added to a solution of the vinylsilane **14** (58 mg, 0.10 mmol, 1.0 equiv) in 1,1,1,3,3,3-hexafluoro-2-propanol (0.3 mL) at 0 °C. The reaction flask was covered with aluminum foil to protect the reaction from light, and NIS (67 mg, 0.3 mmol, 3.0 equiv) was added. After complete consumption of **14** (~20 min, monitored by TLC), a saturated solution of $Na_2S_2O_3$ (10 mL) was added to the reaction flask. The mixture was extracted with Et_2O (5 mL x 3). The combined organic layers were dried over Na_2SO_4, filtered, and concentrated under reduced pressure. The crude mixture was purified by column chromatography (gradient elution with hexane and ethyl acetate, 50:1 to 20:1) to give compound **15** as light yellow oil in 57% yield (31 mg). ^{1}H NMR (600 MHz, $CDCl_3$) δ 7.34 (d, *J* = 8.3 Hz, 2H), 7.30 (dd, *J* = 7.7, 7.7 Hz, 2H), 7.22 (dd, *J* = 7.2, 7.2 Hz, 1H), 6.43 (dd, *J* = 14.4, 9.6 Hz, 1H), 5.94 (d, *J* = 14.4 Hz, 1H), 3.57 (dd, *J* = 9.7, 9.6 Hz, 1H), 3.41 (dd, *J* = 10.2, 3.9 Hz, 1H), 2.43 – 2.47 (m, 1H), 1.59 (s, 3H), 1.43 (s, 1H), 0.99 (s, 9H), 0.81 (s, 9H), 0.12 (s, 3H), -0.06 (s, 3H), -0.09 (s, 6H). ^{13}C NMR (151 MHz, $CDCl_3$) δ 147.4, 145.8, 128.2, 127.0, 125.7, 78.3, 77.7, 62.4, 62.2, 28.2, 26.6, 26.2, 19.1, 18.5, -1.0, -2.0, -4.9, -5.1. HRMS (ESI$^+$): m/z for $C_{24}H_{43}O_2INaSi_2$ $[M+Na]^+$ calcd. 569.1738, found: 569.1736.

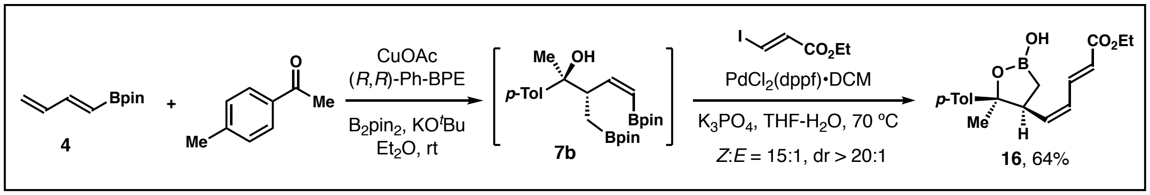

ethyl-(2*E*,4*Z*)-5-((4*S*,5*S*)-2-hydroxy-5-methyl-5-(*p*-tolyl)-1,2-oxaborolan-4-yl)penta-2,4-dienoate (16): In an N_2-filled glove box, CuOAc (1 mg, 0.01 mmol, 10 mol %), (*R,R*)-Ph-BPE (6 mg, 0.012 mmol, 12 mol %), KO^{t}Bu (17 mg, 0.15 mmol, 1.5 equiv), Et_2O (1.0 mL), and a Teflon-coated magnetic stirring bar were sequentially added into a

reaction vial. The resulting mixture was stirred at ambient temperature for 15 min. B_2Pin_2 (38 mg, 0.15 mmol, 1.5 equiv) was added, and the mixture was stirred for 10 min. Then a solution of dienylboronate **4** (18 mg, 0.10 mmol, 1.0 equiv) and ketone (0.15 mmol, 1.5 equiv) in Et_2O (0.5 mL) were added to the reaction vial in one portion. The reaction mixture was stirred at ambient temperature inside the glove box and the reaction progress was monitored by ^{1}H NMR analysis. After complete consumption of dienylboronate **4**, the reaction mixture was filtered through a short pad of silica gel and Celite. The filtrate was concentrated under reduced pressure. The crude mixture was transferred into a glove box and used without purification. In the glove box, $PdCl_2$(dppf)·DCM (8 mg, 0.01 mmol, 10 mol %), K_3PO_4 (28 mg, 0.13 mmol, 1.3 equiv), vinyl iodide (29 mg, 0.13 mmol, 1.3 equiv), THF (1 mL) and a stirring bar were sequentially added to crude mixture. The vial was sealed with rubber septum and removed from the glove box. Then water (0.1 mL) was added to the vial under an argon atmosphere. Then the vial was sealed with a cap containing a PTFE-lined silicone septum and stirred at 70 °C for 14 h. After completion of the reaction, the mixture was filtered through a short pad of Celite. Brine (1 mL) was added, and the mixture was extracted with Et_2O (1 mL x 3). The combined organic layers were dried over anhydrous Na_2SO_4, filtered, and concentrated under reduced pressure. Purification of the crude reaction product was performed by column chromatography (gradient elution with hexane and ethyl acetate) to give product **16** in 64% yield (20 mg, *Z*,*E*: *E*,*E* = 15:1) as colorless oil over two steps. ^{1}H NMR (600 MHz, $CDCl_3$) δ 7.64 (dd, *J* = 15.2, 11.7 Hz, 1H), 7.11 (d, *J* = 8.3 Hz, 2H), 7.08 (d, *J* = 8.3 Hz, 2H), 5.97 (dd, *J* = 11.5, 11.5 Hz, 1H), 5.88 (d, *J* = 14.8 Hz, 1H), 5.16 (d, *J* = 10.9, 10.9 Hz, 1H), 4.78 (s, 1H), 4.23 (q, *J* = 7.1 Hz, 2H), 3.50 – 3.55 (m, 1H), 2.33 (s, 3H), 1.66 (s, 3H), 1.36 (dd, *J* = 16.9, 8.0 Hz, 1H), 1.32 (t, *J* = 7.1 Hz, 3H), 0.86 (dd, *J* = 17.0, 8.4 Hz, 1H). ^{13}C NMR (126 MHz, $CDCl_3$) δ 167.5, 142.9, 140.3, 139.3, 136.8, 129.0, 126.1, 125.7, 122.7, 87.7, 60.8, 48.4, 29.9, 21.3, 14.7. HRMS (ESI$^+$): m/z for $C_{18}H_{24}BO_4$ $[M+H]^+$ calcd. 315.1762, found: 315.1747.

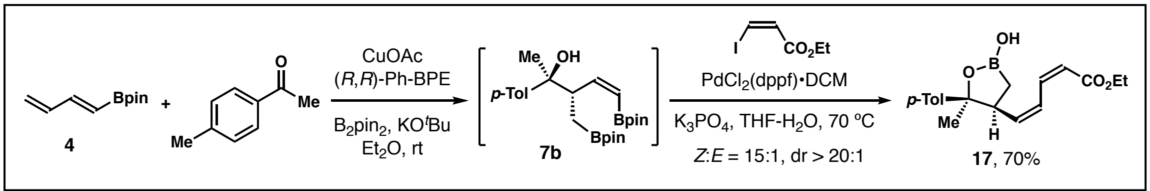

ethyl-(2*Z*,4*Z*)-5-((4*S*,5*S*)-2-hydroxy-5-methyl-5-(*p*-tolyl)-1,2-oxaborolan-4-yl)penta-2,4-dienoate (17): The procedure for the synthesis of compound **16** was utilized. *Z*-Vinyl iodide was used as the coupling partner. Purification of the crude reaction product was

performed by flash column chromatography (gradient elution with hexane and ethyl acetate) to give product **17** in 70% yield (22 mg, *Z*,*Z*: *E*,*Z* = 15:1) as colorless oil over two steps. ^{1}H NMR (600 MHz, $CDCl_3$) δ 7.17 (dd, *J* = 11.6, 11.6 Hz, 1H), 7.12 (d, *J* = 8.2 Hz, 2H), 7.08 (d, *J* = 8.2 Hz, 2H), 6.98 (dd, *J* = 11.8, 11.8 Hz, 1H), 5.73 (dd, *J* = 11.5, 1.4 Hz, 1H), 5.19 (dd, *J* = 10.9, 10.9 Hz, 1H), 4.74 (s, 1H), 4.18 (q, *J* = 7.1 Hz, 2H), 3.44 – 3.49 (m, 1H), 2.33 (s, 3H), 1.65 (s, 3H), 1.29 (t, *J* = 7.1 Hz, 3H), 1.23 – 1.27 (m, 1H), 0.87 (dd, *J* = 16.8, 9.5 Hz, 1H). ^{13}C NMR (151 MHz, $CDCl_3$) δ 166.8, 142.9, 139.9, 138.7, 136.9, 129.0, 125.8, 124.2, 118.9, 87.7, 60.4, 47.9, 29.7, 21.3, 14.6. HRMS (ESI$^+$): for $C_{18}H_{24}BO_4$ $[M+H]^+$ calcd. 315.1762, found: 315.1777.

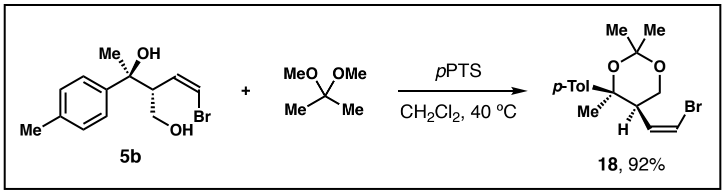

(4*S*,5*R*)-5-((*Z*)-2-bromovinyl)-2,2,4-trimethyl-4-(*p*-tolyl)-1,3-dioxane (18): To a solution of diol **5b** (29 mg, 0.1 mmol, 1.0 equiv) in 2,2-dimethoxypropane (1 mL) was added *p*PTS (2 mg) and dichloromethane (0.2 mL). The reaction mixture was kept stirring at 40 °C and the progress was monitored by TLC analysis (~1 h). After complete consumption of diol **5b**, the reaction mixture was filtered through a short pad of silica gel. The filtrate was concentrated under reduced pressure. Purification of the crude product was performed by flash column chromatography (gradient elution with hexane and ethyl acetate) to afford acetonide **18** in 92% yield (30 mg) as colorless oil. ^{1}H NMR (600 MHz, $CDCl_3$) δ 7.23 (d, *J* = 8.2 Hz, 2H), 7.10 (d, *J* = 8.1 Hz, 2H), 6.10 (dd, *J* = 9.3, 7.0 Hz, 1H), 5.97 (dd, *J* =7.0, 0.8 Hz, 1H), 4.43 (dd, *J* = 11.8, 2.8 Hz, 1H), 3.80 (dd, *J* = 11.8, 2.6 Hz, 1H), 3.00 (dt, *J* = 9.3, 2.5 Hz, 1H), 2.32 (s, 3H), 1.73 (s, 3H), 1.63 (s, 3H), 1.51 (s, 3H). ^{13}C NMR (151 MHz, $CDCl_3$) δ 144.9, 136.2, 135.3, 128.9, 124.7, 107.9, 99.2, 76.1, 61.5, 43.3, 31.5, 30.5, 25.4, 21.3. HRMS (ESI$^+$): m/z for $C_{16}H_{22}BrO_2$ $[M+H]^+$ calcd. 325.0798, found: 325.0797.

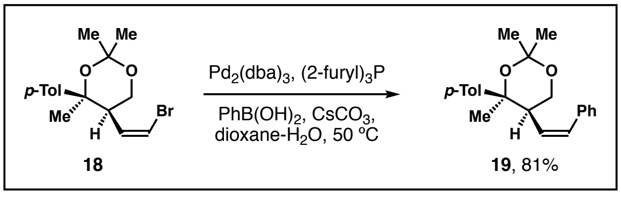

(4*S*,5*R*)-2,2,4-trimethyl-5-((*Z*)-styryl)-4-(*p*-tolyl)-1,3-dioxane (19): In a N_2-filled glove

box, to a reaction vial containing a stirring bar were added $Pd_2(dba)_3$ (1 mg, 0.0025 mmol, 2.5 mol %), $(2\text{-furyl})_3P$ (2 mg, 0.015 mmol, 15 mol %) and dioxane (0.5 mL), the mixture was stirred for 15 min at ambient temperature. Then vinyl bromide **18** (32 mg, 0.1 mmol, 1.0 equiv), phenyl boronic acid (16 mg, 0.13 mmol, 1.3 equiv) and Cs_2CO_3 (66 mg, 0.2 mmol, 2.0 equiv) were sequentially added. The vial was sealed with a rubber septum and removed from the glove box. Then water (100 μL) was added under an argon atmosphere. The mixture was stirred at 50 °C for 12 h. After completion of the reaction, the mixture was filtered through a short pad of Celite. Brine (1 mL) was added to the filtrate, and the mixture was extracted with Et_2O (1 mL x 3). The combined organic layers were dried over anhydrous Na_2SO_4, filtered, and concentrated under reduced pressure. Purification of the crude product was performed by column chromatography (gradient elution with hexane and ethyl acetate) to give product **19** in 81% yield (26 mg) as colorless oil. ^1H NMR (500 MHz, $CDCl_3$) δ 7.33 – 7.36 (m, 2H), 7.24 – 7.27 (m, 1H), 7.11 – 7.12 (m, 4H), 7.06 (d, *J* = 8.1 Hz, 2H), 6.29 (d, *J* = 11.8 Hz, 1H), 5.67 (dd, *J* = 11.8, 10.6 Hz, 1H), 4.44 (dd, *J* = 11.5, 3.4 Hz, 1H), 3.88 (dd, *J* = 11.5, 3.5 Hz, 1H), 3.02 (dt, *J* = 10.7, 3.5 Hz, 1H), 2.32 (s, 3H), 1.63 (s, 3H), 1.61 (s, 3H), 1.54 (s, 3H). ^{13}C NMR (126 MHz, $CDCl_3$) δ 144.9, 138.1, 135.9, 132.7, 129.5, 128.8, 128.7, 128.6, 127.0, 125.3, 99.2, 76.9, 62.9, 40.6, 31.1, 30.8, 26.1, 21.3. HRMS (ESI^+): m/z for $C_{22}H_{27}O_2$ $[M+H]^+$ calcd. 323.2006, found: 323.2015.

References:

1. Gao, S.; Chen, M. *Chem, Sci.* **2019**, *10*, 7554
2. Gao, S.; Chen, M. *Chem. Commun.* **2019**, *55*, 11199.

Transition State Analyses - Geometric Factors

To reveal the factors that control the selectivity, we have analyzed the contribution of various geometric factors to the transition-state energies of the competing pathways. Repulsive steric interactions have been estimated by measuring H-H distances between the catalyst, allyl, and ketone carbonyl groups. At the B3LYP/def2-tzvp/D3 level, H-H interactions become repulsive at distances below 2.10 Å (Figure SI-1). Tables SI-2 and SI-3 therefore list the values of intermolecular H-H contacts below 2.10 Å for the various transition states studied here as a way to estimate the importance of repulsive interactions at the transition states.

Conformational strain has been estimated by separating the allyl moiety from the catalyst, to which the coupling ketone is coordinated. When separating the two fragments, the dangling radicals emerging from the dissociation of the bond between the Cu atom and the allyl moiety are saturated with hydrogen atoms. All hydrogen atoms of the allyl, catalyst and carbonyl fragments have been optimized while holding fixed the geometries of the non-hydrogen atoms to capture specific conformational strains at each transition state.

For the reaction involving α-silyl allylcopper intermediate in Scheme 5, we see that transition state **TS-3** presents both a less repulsive landscape, and a more favorable conformational profile than **TS-4** and **TS-5** (Table SI-2). For **TS-3**, only one H-H intermolecular distance can be labeled as repulsive, while for **TS-4** and **TS-5**, two and three such H-H contacts exist, respectively. The geometry of the allyl moiety at the transition state is remarkably less strained for **TS-3**, reflecting that the location of the silyl group in the pseudo-equatorial position in the Zimmerman-Traxler transition state might not only stabilize the transition state through favorable orbital interactions, but is also less strained. The Cu-containing moiety is significantly more strained for **TS-5** compared to the other two transition states, contributing to the larger barrier along that pathway. These effects combine to make **TS-3** the lowest-energy transition state, followed by **TS-4** and **TS-5** (Scheme 5).

Table SI-2: Analyses of the repulsive H-H contacts and conformational-strain energies in the ketone coupling with α-silyl allylcopper species.

	**TS-3**	**TS-4**	**TS-5**
r(H-H) [a]	1.95	1.81, 2.01	2.01, 2.02, 2.09
E_{allyl} [b]	0	23.1	15.9
$E_{cat+carbonyl}$ [c]	3.6	0.0	17.2

[a] Intermolecular H-H distances below 2.10 Å (units: Å)
[b] Relative energy (kJ/mol) between allyl geometries at the geometry of the transition state.
[c] Relative energy (kJ/mol) between the catalyst geometries at the geometry of the transition state. The Cu catalyst is coordinated to the ketone carbonyl group that undergoes the reaction.

The steric and conformational-strain analyses for the reactions with α-boryl allylic copper species in Scheme 7 indicate that the environment in the lowest-energy transition state (**TS-6**) is less repulsive than for **TS-7** and **TS-8** (Table SI-3). Indeed, there is no H-H distance below the 2.10 Å for **TS-6**, while there are two for transition states **TS-7** and **TS-8**. The allyl moiety is also less strained for **TS-6** at the transition state. The most energetic transition state (**TS-5**) shows the largest strain in the catalyst moiety.

Table SI-3. Analyses of the repulsive H-H contacts and conformational-strain energies in the ketone coupling reaction with α-boryl allylcopper species.

	**TS-6**	**TS-7**	**TS-8**
r(H-H)[a]	None	1.99, 2.09	1.96, 2.01
E_{allyl}[b]	0	6.8	8.2
$E_{cat+carbonyl}$[c]	6.2	14.6	0.0

[a] Intermolecular H-H distances below 2.10 Å (units: Å)
[b] Relative energy (kJ/mol) between allyl geometries at the geometry of the transition state.
[c] Relative energy (kJ/mol) between catalyst geometries at the geometry of the transition state.

The Cu catalyst is coordinated to the ketone carbonyl group that undergoes the reaction.

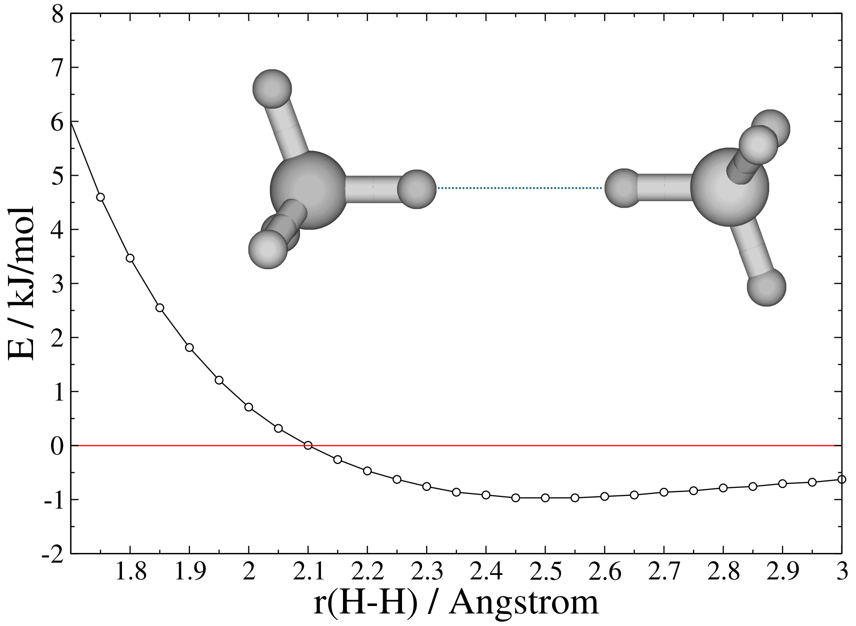

Figure SI-I: Intermolecular potential energy curve for interacting CH_4 molecules. The data correspond to a frozen scan of the H-H intermolecular distance highlighted in the inset at the B3LYP/def2-tzvp/D3 level.

Computational Details

Geometry optimizations and frequency calculations were conducted with the r^2SCAN-3c method using ORCA 6.0.1.[1-3] r^2SCAN-3c is a composite method based on the r^2SCAN meta-GGA density functional,[4] and considers D4 dispersion,[5] the modified def2-mTZVPP basis set of triple-z quality, and a counterpoise correction for the geometries.[6] The calculations used default cutoffs and integration grid together with the def2/J density-fitting set.[7] The reported energies correspond to 298 K Gibbs energies. Images were generated with CYLview20.[8]

References:

(1) Neese, F. The ORCA program system. *Wires Comput. Mol. Sci.* **2012**, *2*, 73-78. DOI: 10.1002/wcms.81.

(2) Neese, F. Software update: the ORCA program system, version 4.0. *Wires Comput. Mol. Sci.* **2018**, *8*, e1327. DOI: 10.1002/wcms.1327.

(3) Neese, F.; Wennmohs, F.; Becker, U.; Riplinger, C. The ORCA quantum chemistry program package. *J. Chem. Phys.* **2020**, *152*. DOI: 10.1063/5.0004608.

(4) Furness, J. W.; Kaplan, A. D.; Ning, J. L.; Perdew, J. P.; Sun, J. W. Accurate and Numerically Efficient r2SCAN Meta-Generalized Gradient Approximation. *J. Phys. Chem. Lett.* **2020**, *11*, 8208-8215. DOI: 10.1021/acs.jpclett.0c02405.

(5) Najibi, A.; Goerigk, L. DFT-D4 counterparts of leading meta-generalized-gradient approximation and hybrid density functionals for energetics and geometries. *J. Comput. Chem.* **2020**, *41*, 2562. DOI: 10.1002/jcc.26411.

(6) Kruse, H.; Grimme, S. A geometrical correction for the inter- and intra-molecular basis set superposition error in Hartree-Fock and density functional theory calculations for large systems. *J. Chem. Phys.* **2012**, *136*, 154101. DOI: 10.1063/1.3700154.

(7) Weigend, F. Accurate Coulomb-fitting basis sets for H to Rn. *Phys. Chem. Chem. Phys.* **2006**, *8*, 1057. DOI: 10.1039/b515623h.

(8) *CYLview20, Université de Sherbrooke,* http://www.cylview.org; 2020. (accessed 11/25/25).

1) *s*-trans to *s*-cis isomerization of dienes **1a** or **4** via **TS-12a** or **TS-12b**

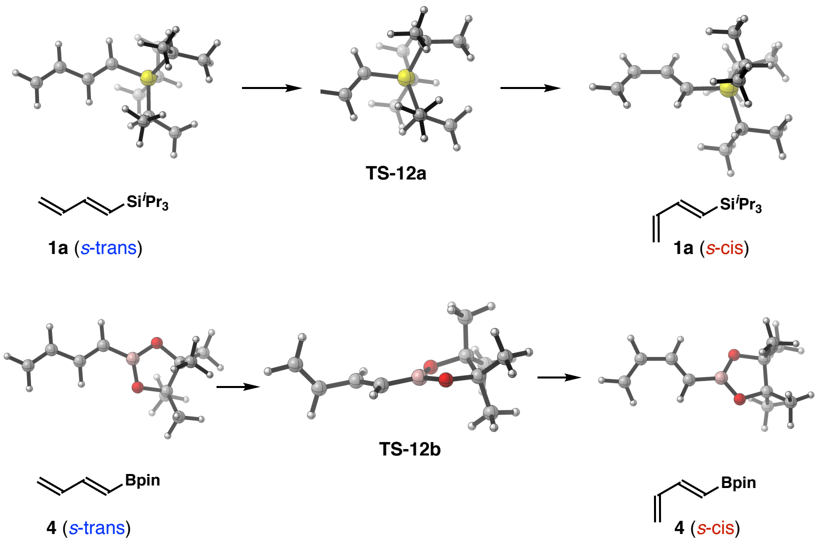

2) Isomerization of allylcopper intermediates derived from diene **1a**

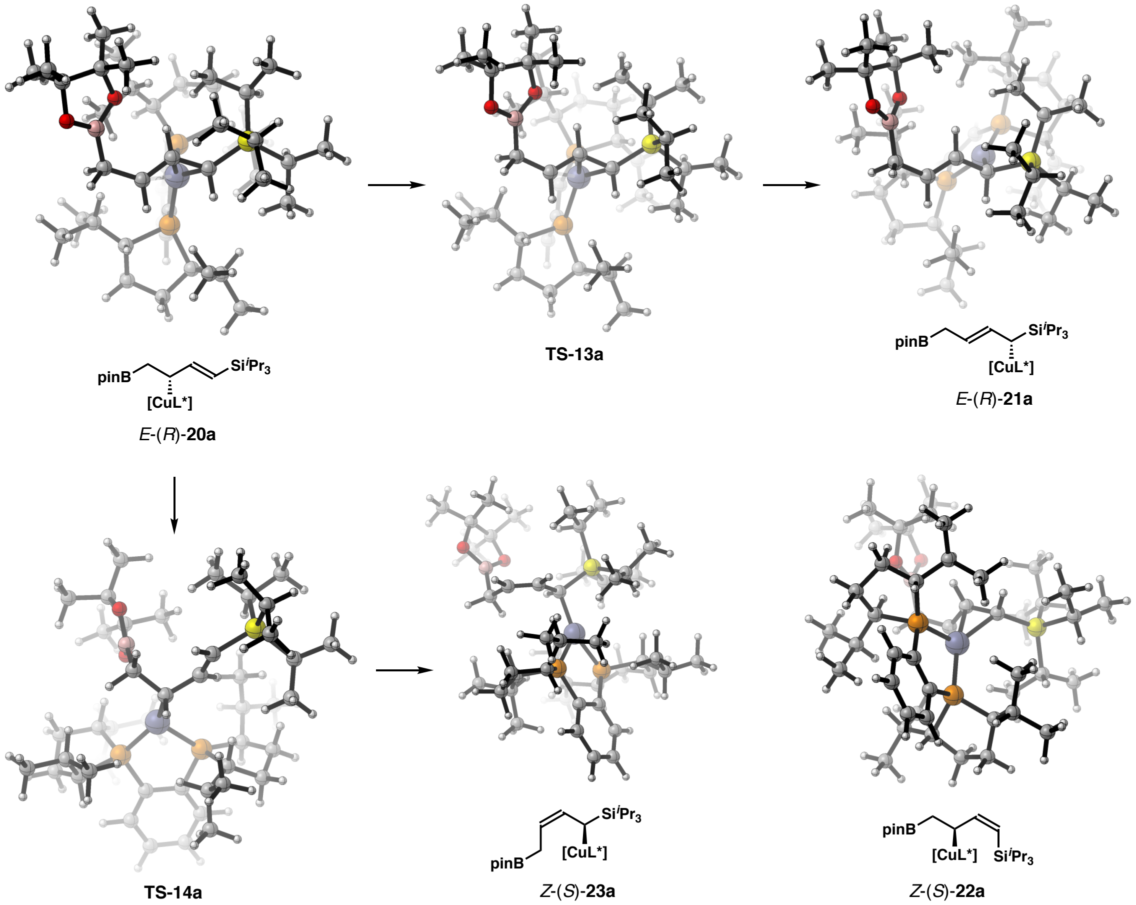

3) Isomerization of allylcopper intermediates derived from diene **4**

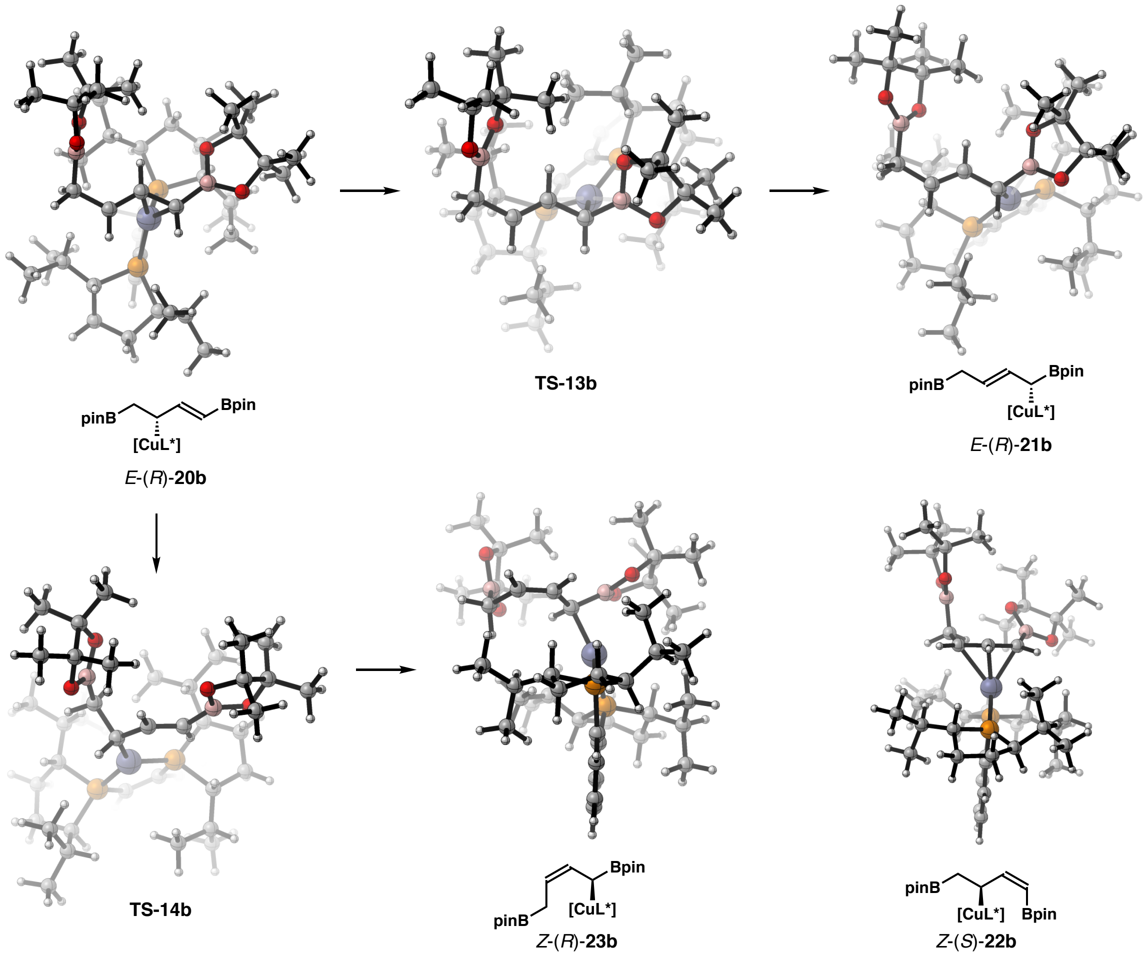

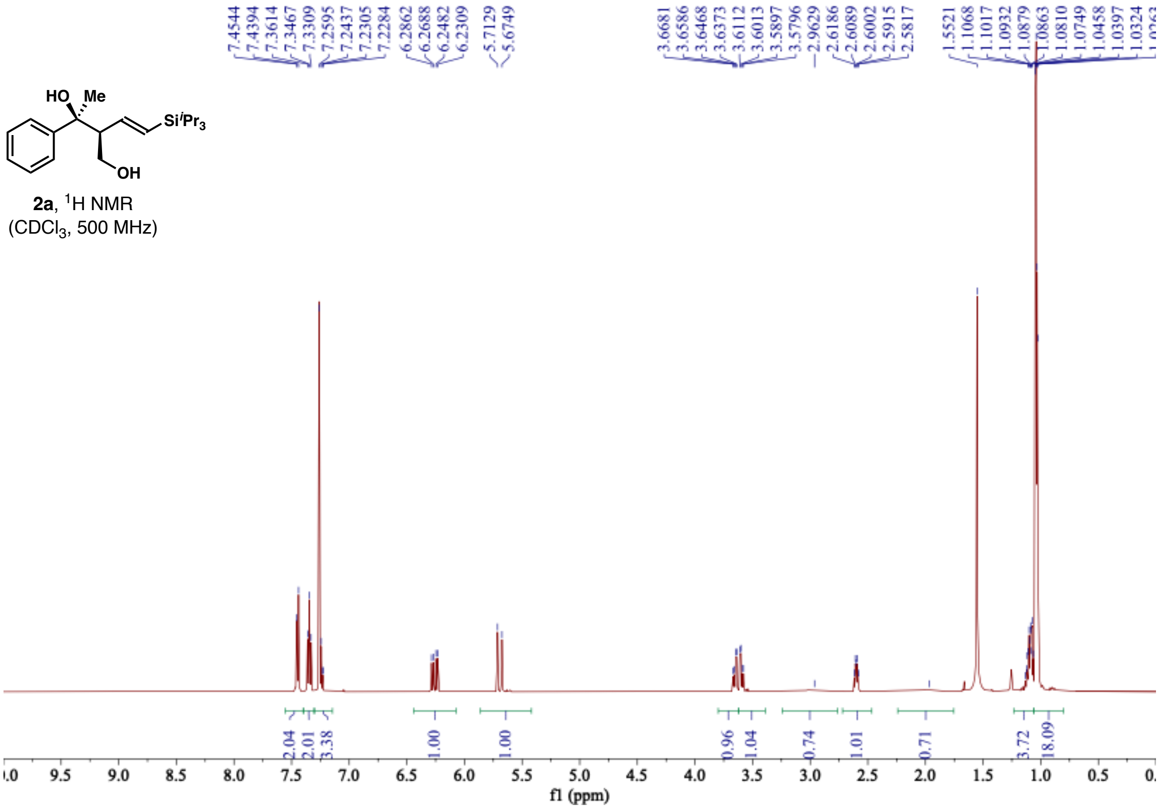

2a, ^{1}H NMR (CDCl$_3$, 500 MHz)

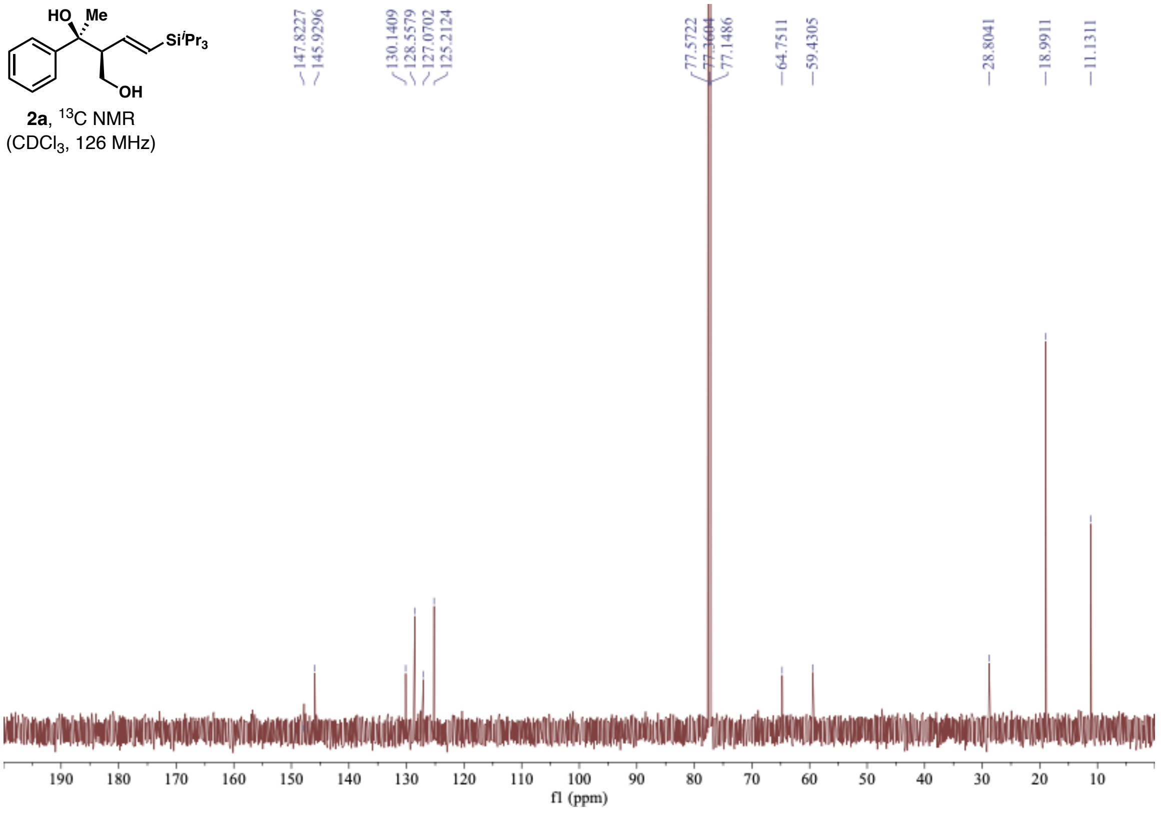

2a, ^{13}C NMR (CDCl$_3$, 126 MHz)

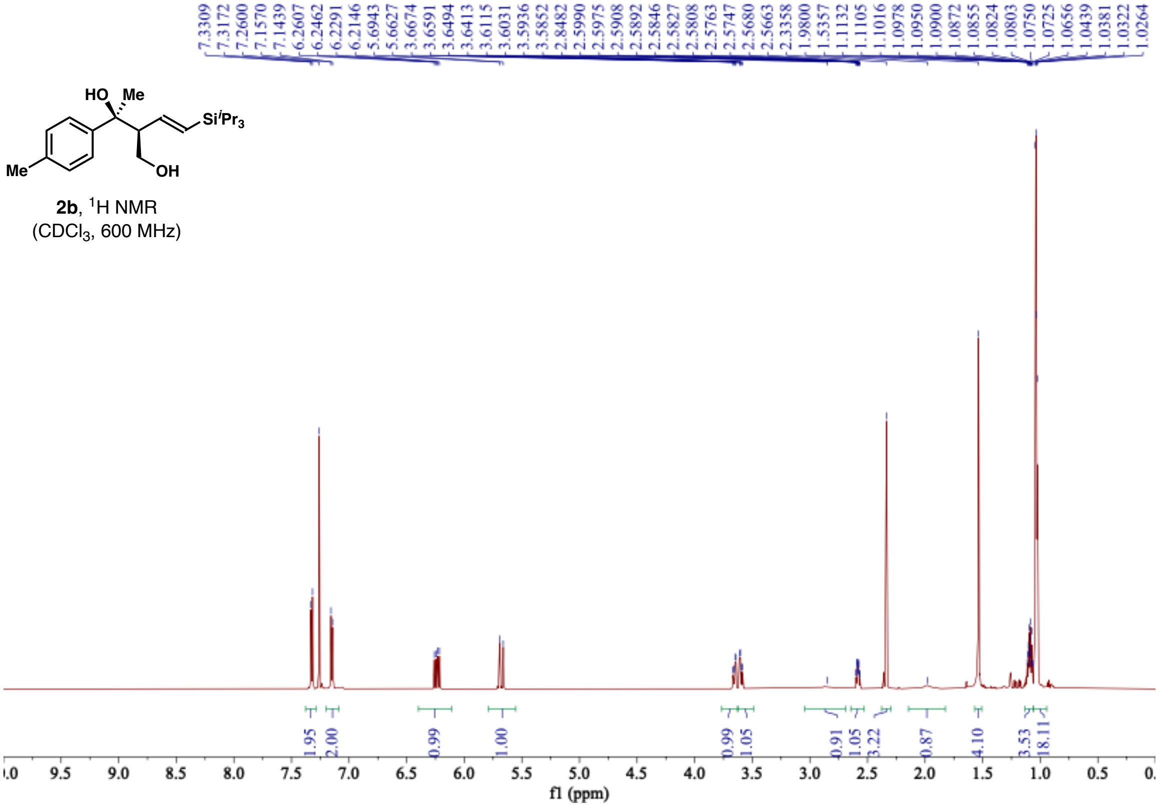

2b, ^{1}H NMR
(CDCl$_3$, 600 MHz)

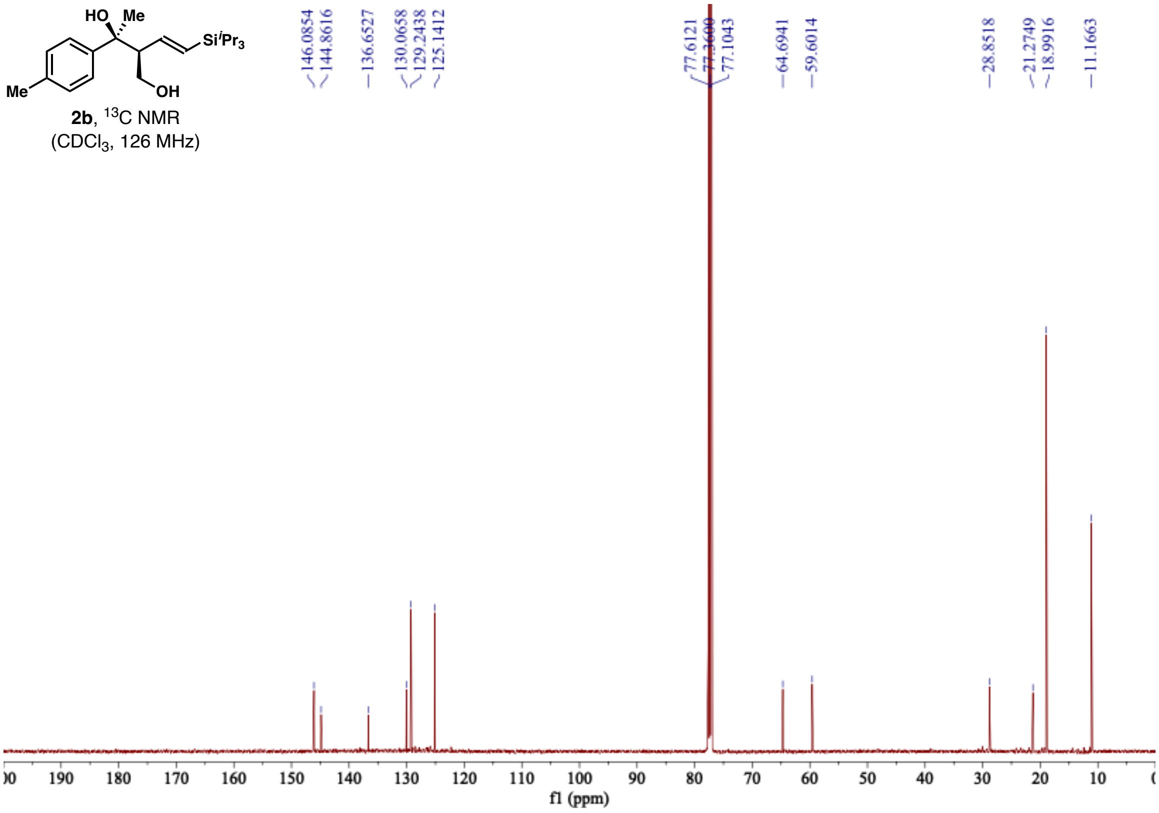

2b, ^{13}C NMR
(CDCl$_3$, 126 MHz)

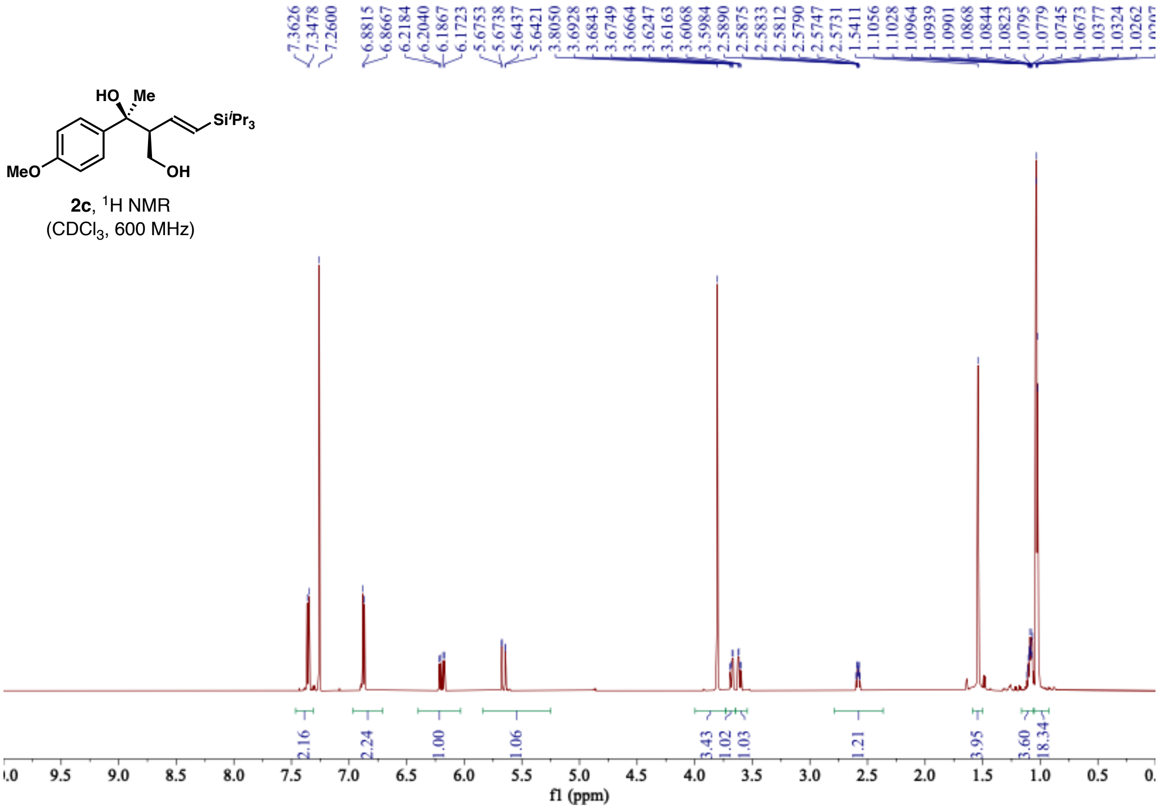
HO Me
Si^{i}Pr$_3$
MeO
OH
2c, ^{1}H NMR
(CDCl$_3$, 600 MHz)
f1 (ppm)

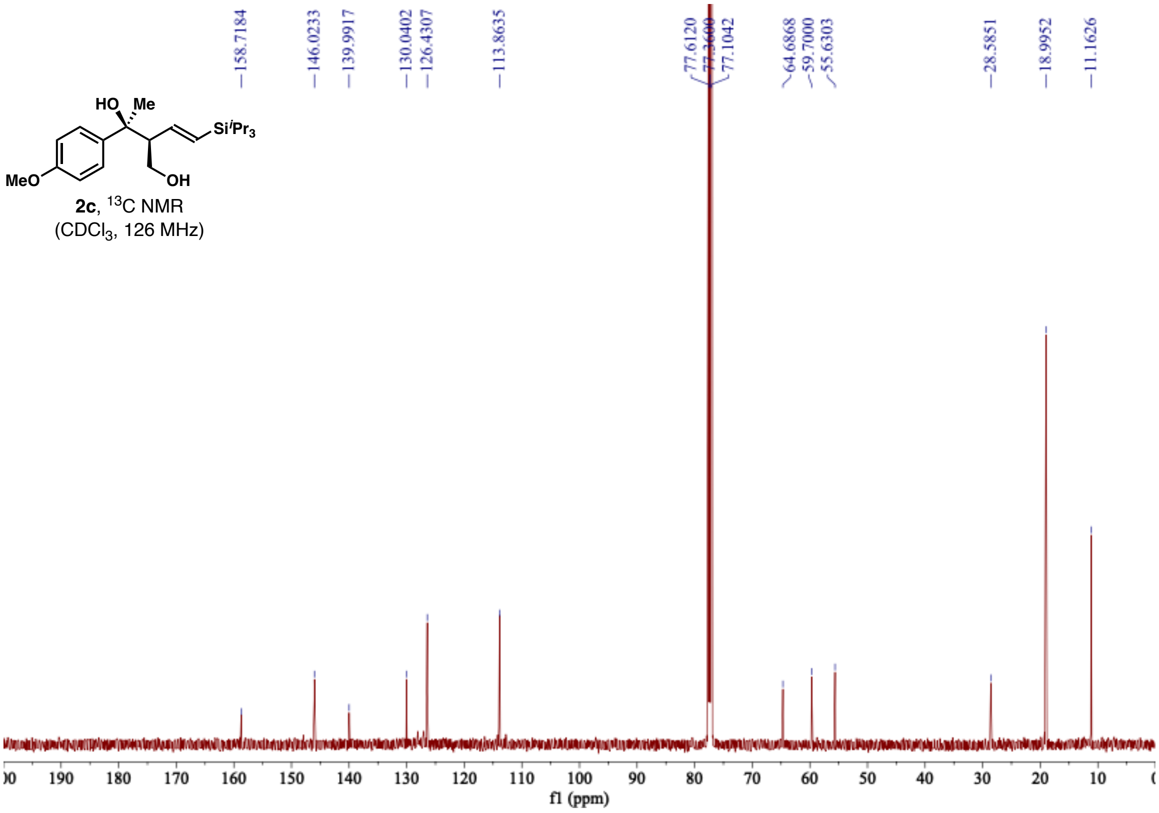
HO Me
Si^{i}Pr$_3$
MeO
OH
2c, ^{13}C NMR
(CDCl$_3$, 126 MHz)
f1 (ppm)

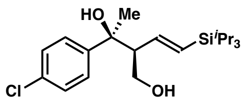

2d, ^{1}H NMR
($CDCl_3$, 500 MHz)

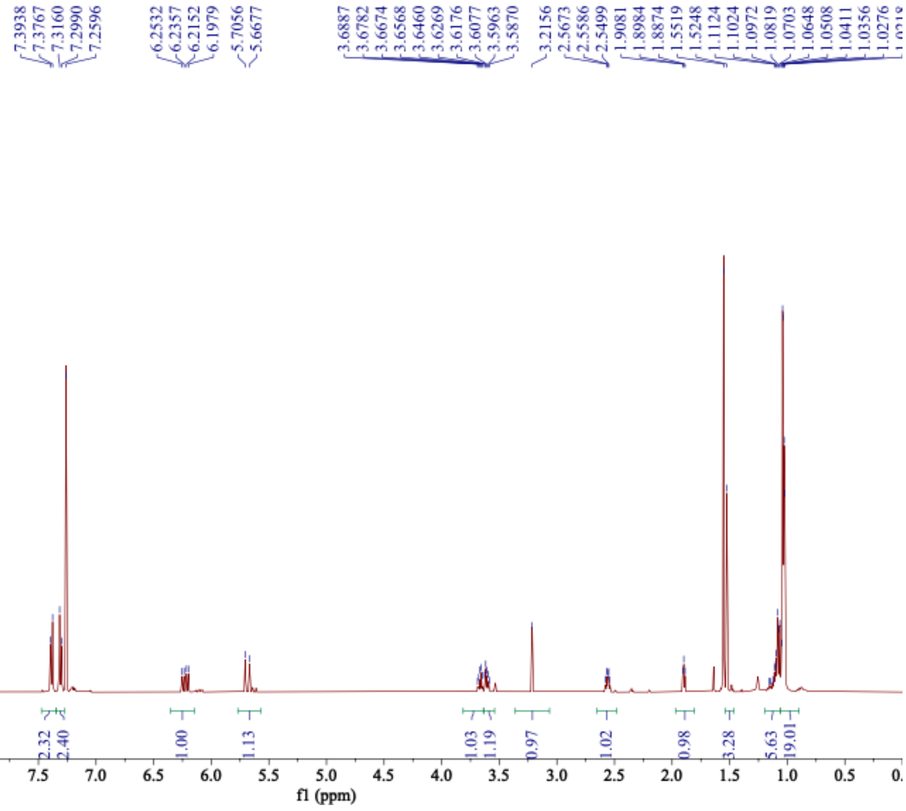

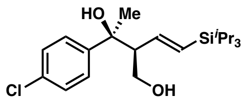

2d, ^{13}C NMR
($CDCl_3$, 126 MHz)

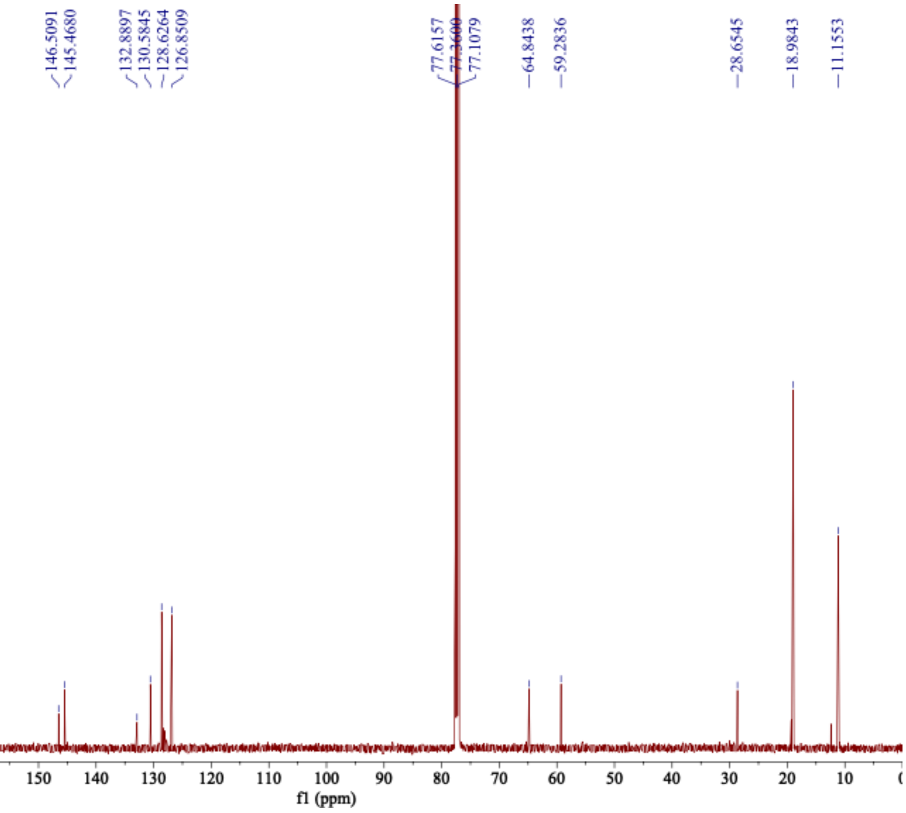

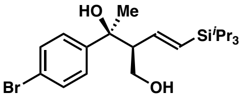

2e, ^{1}H NMR
($CDCl_3$, 600 MHz)

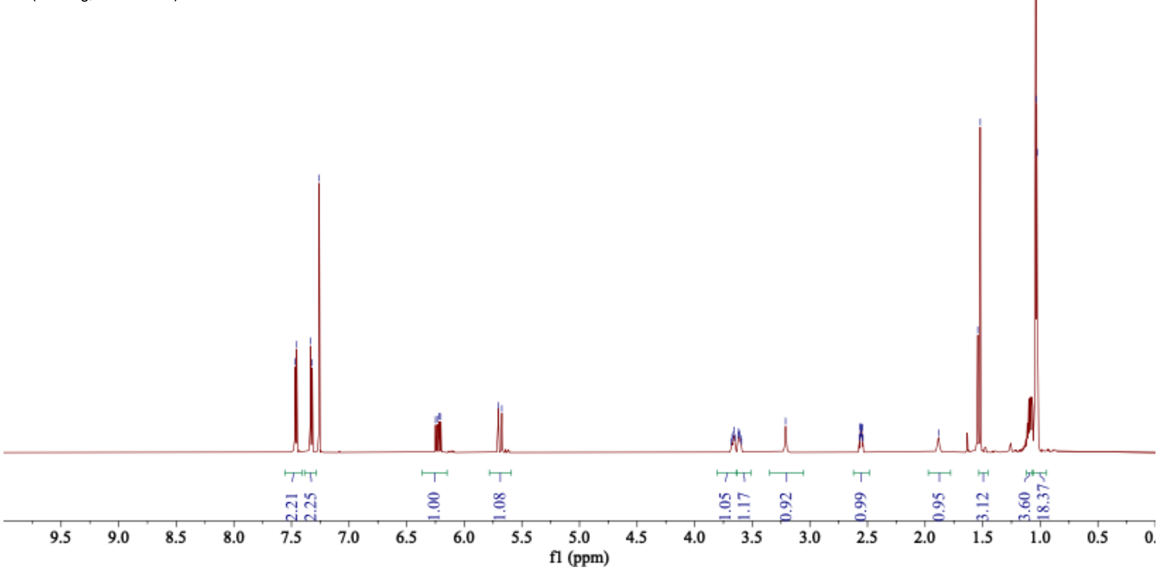

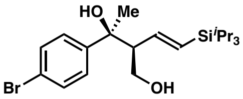

2e, ^{13}C NMR
($CDCl_3$, 126 MHz)

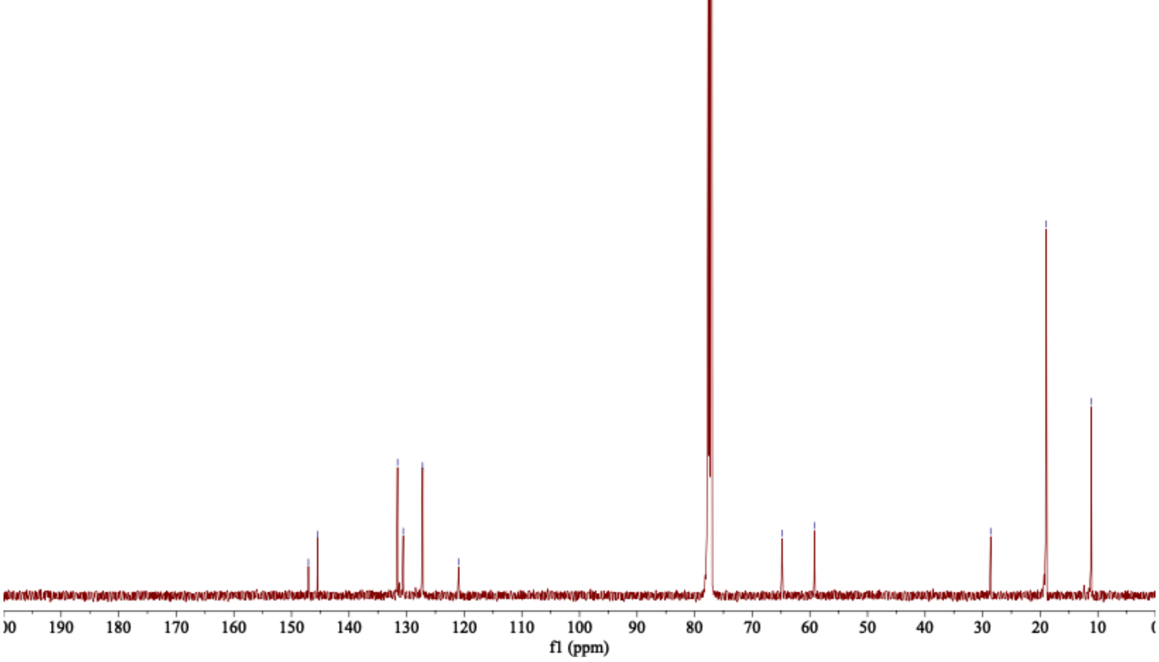

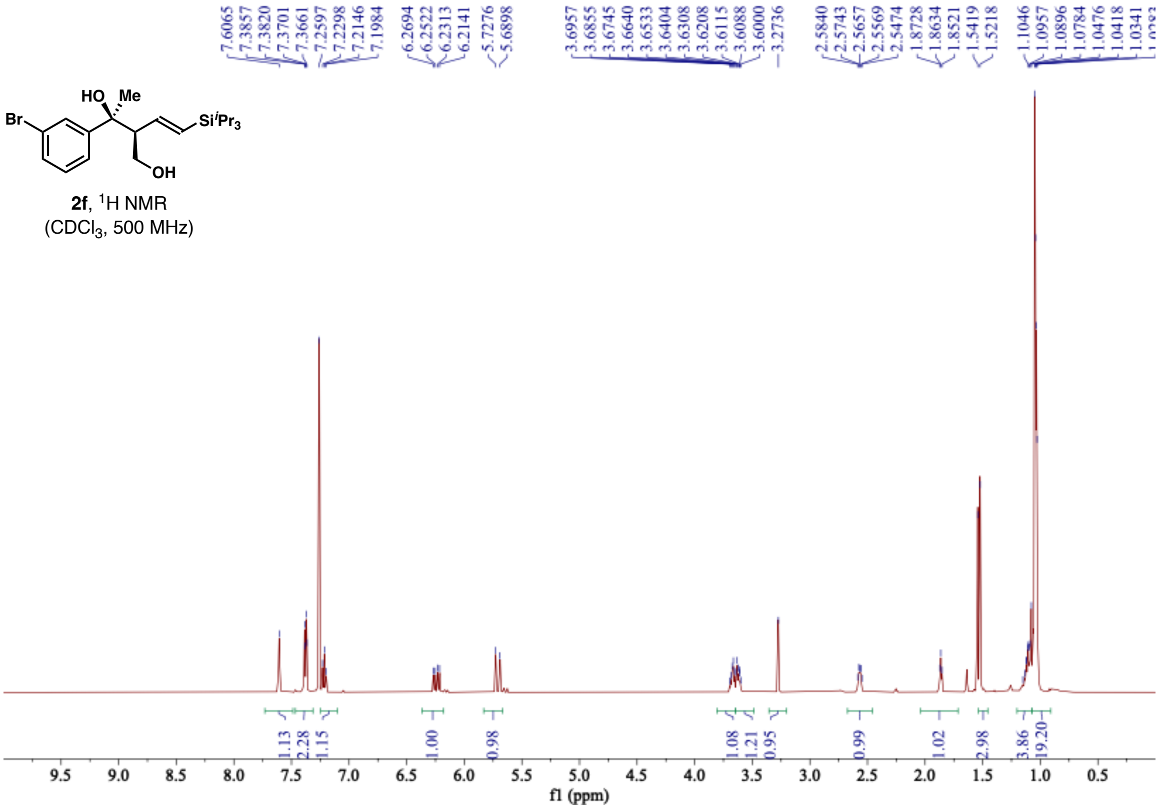
2f, 1H NMR
(CDCl3, 500 MHz)
f1 (ppm)

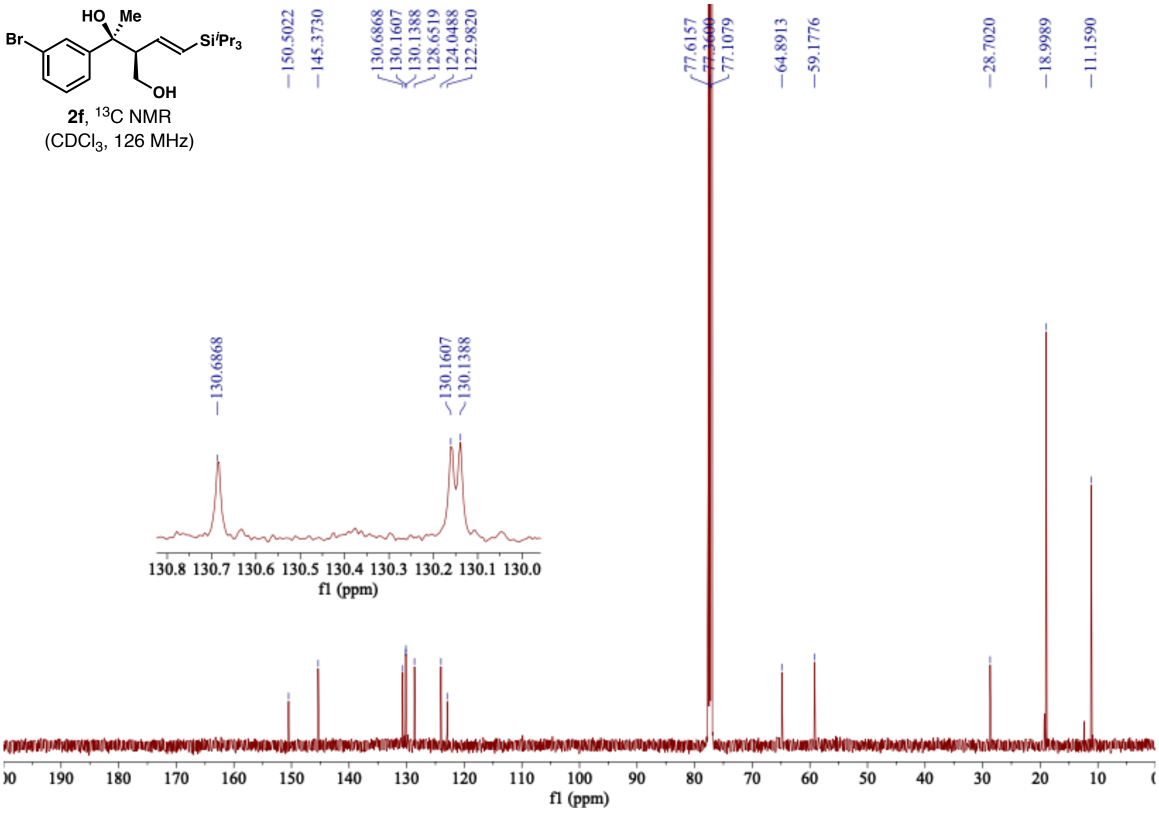
2f, 13C NMR
(CDCl3, 126 MHz)
150.5022
145.3730
130.6868
130.1607
130.1388
128.6519
124.0488
122.9820
77.6157
77.1079
64.8913
59.1776
28.7020
18.9989
11.1590
f1 (ppm)

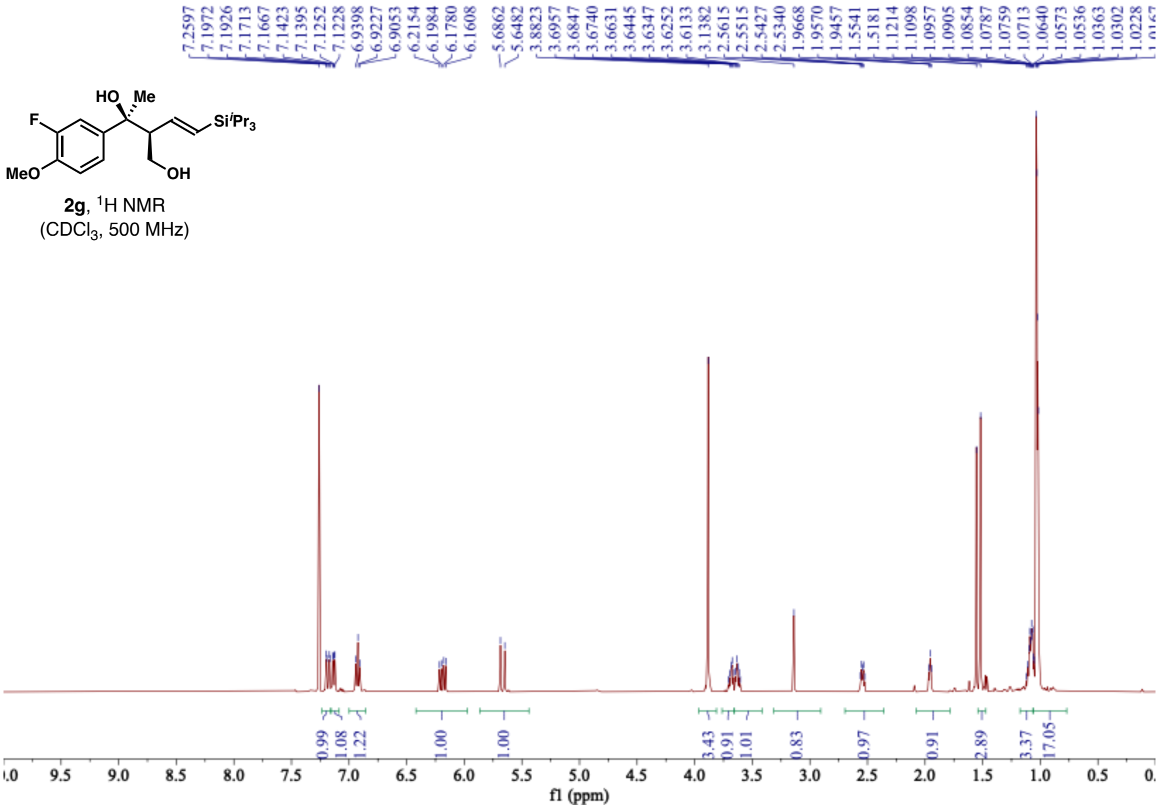

HO Me
F
SiiPr3
MeO
OH
2g, 1H NMR
(CDCl3, 500 MHz)
f1 (ppm)

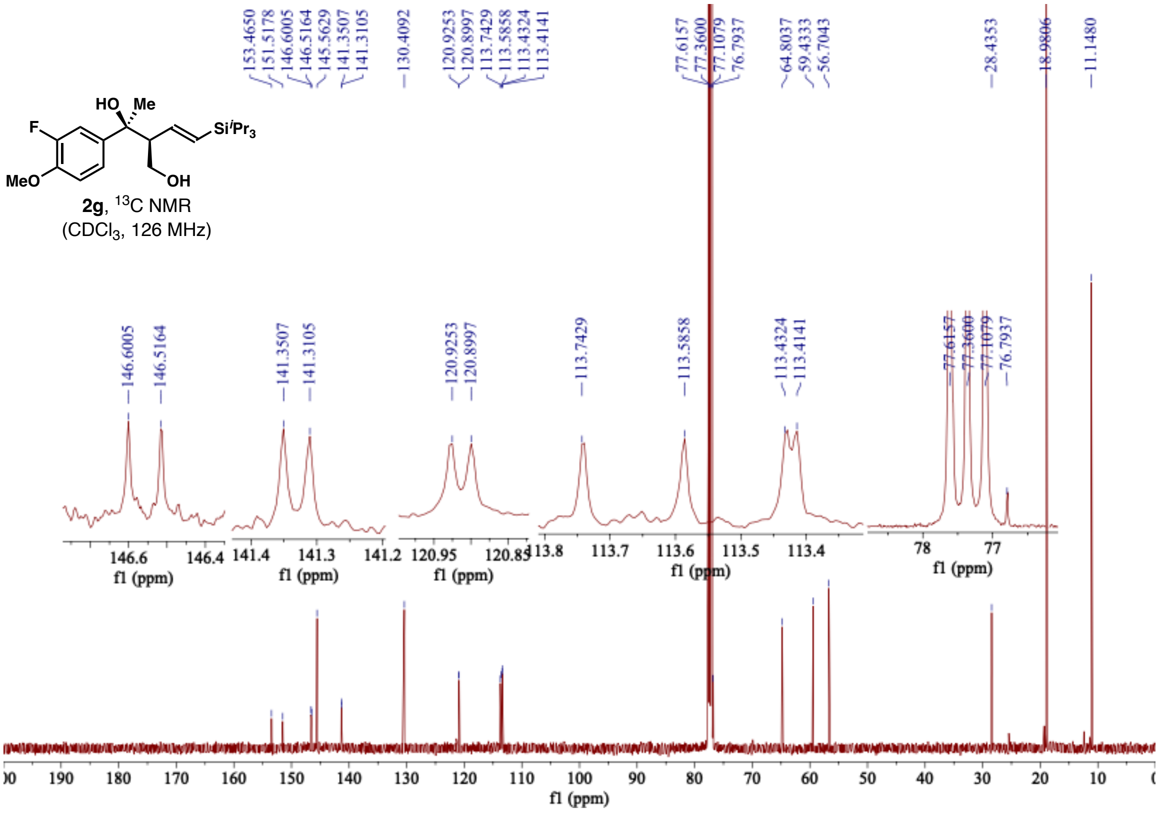

HO Me
F
SiiPr3
MeO
OH
2g, 13C NMR
(CDCl3, 126 MHz)
f1 (ppm)

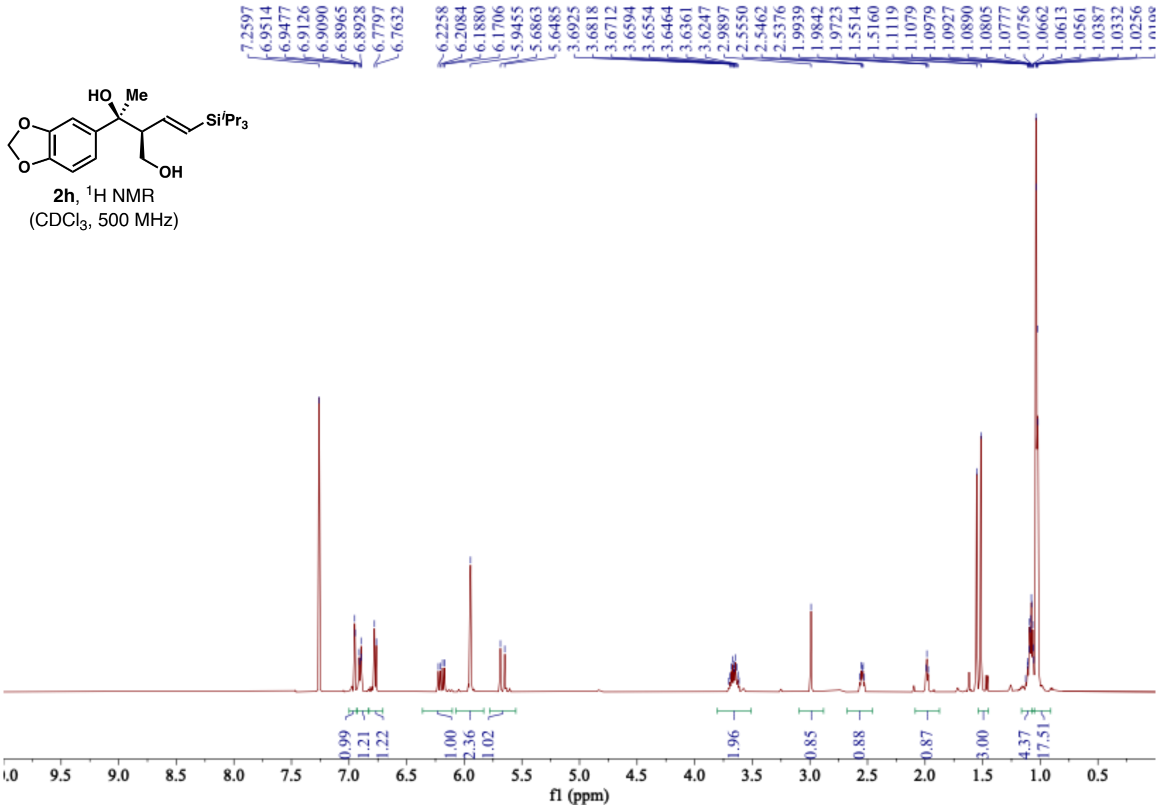

HO Me
Si^{i}Pr$_3$
OH
2h, ^{1}H NMR
($CDCl_3$, 500 MHz)
7.2597 6.9514 6.9477 6.9126 6.9090 6.8965 6.8928 6.7797 6.7632 6.2258 6.2084 6.1880 6.1706 5.9455 5.6863 5.6485 3.6925 3.6818 3.6712 3.6594 3.6554 3.6464 3.6361 3.6247 2.9897 2.5550 2.5462 2.5376 1.9939 1.9842 1.9723 1.5514 1.5160 1.1119 1.1079 1.0979 1.0927 1.0890 1.0805 1.0777 1.0756 1.0662 1.0613 1.0561 1.0387 1.0332 1.0256
0.99 1.21 1.22 1.00 2.36 1.02 1.96 0.85 0.88 0.87 3.00 4.37 17.51
9.5 9.0 8.5 8.0 7.5 7.0 6.5 6.0 5.5 5.0 4.5 4.0 3.5 3.0 2.5 2.0 1.5 1.0 0.5
f1 (ppm)

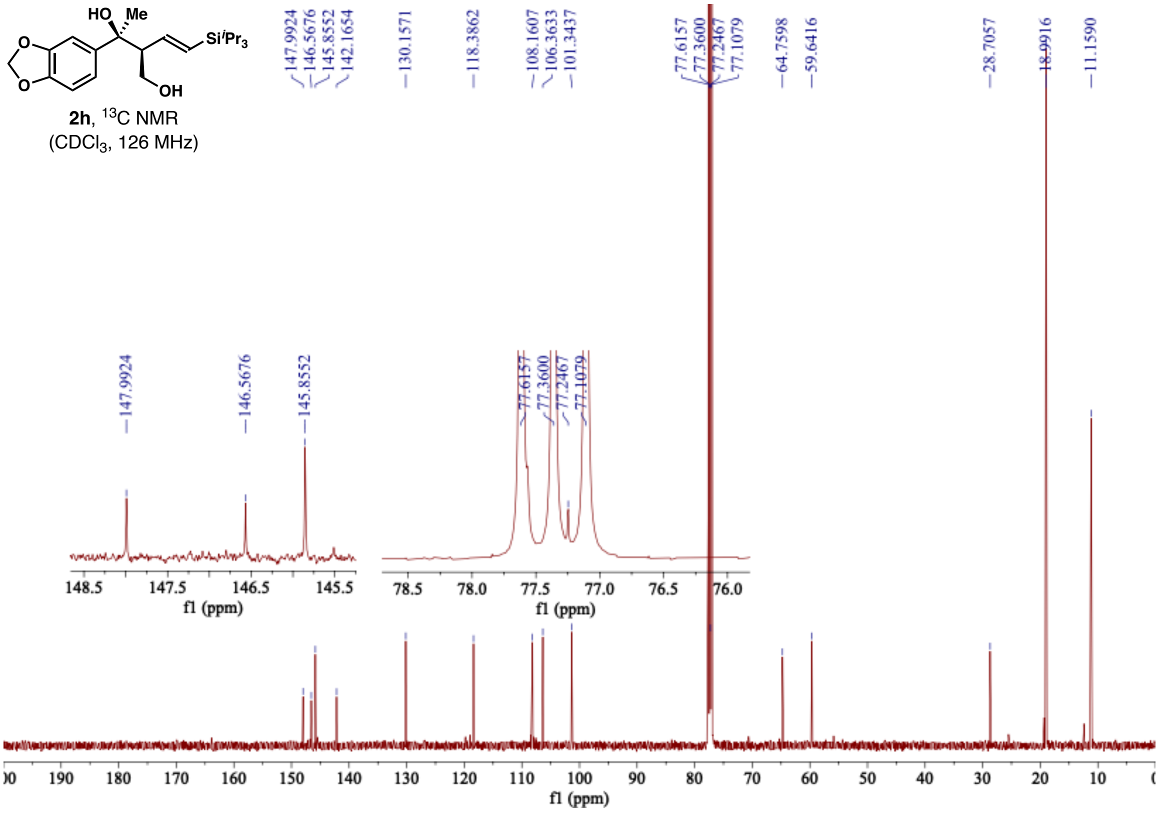

HO Me
Si^{i}Pr$_3$
OH
2h, ^{13}C NMR
($CDCl_3$, 126 MHz)
147.9924 146.5676 145.8552 142.1654 130.1571 118.3862 108.1607 106.3633 101.3437 77.6157 77.3600 77.2467 77.1079 64.7598 59.6416 28.7057 18.9916 11.1590
148.5 147.5 146.5 145.5
f1 (ppm)
78.5 78.0 77.5 77.0 76.5 76.0
f1 (ppm)
190 180 170 160 150 140 130 120 110 100 90 80 70 60 50 40 30 20 10
f1 (ppm)

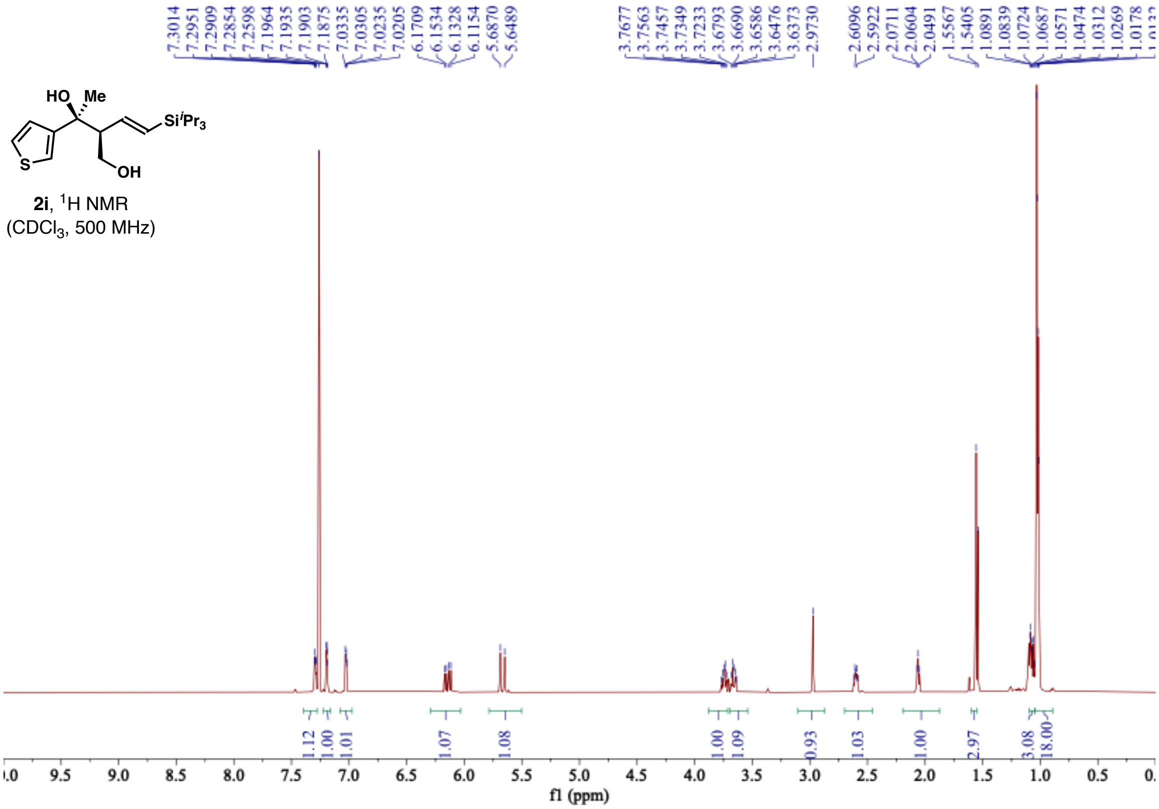

2i, ^{1}H NMR
(CDCl$_3$, 500 MHz)

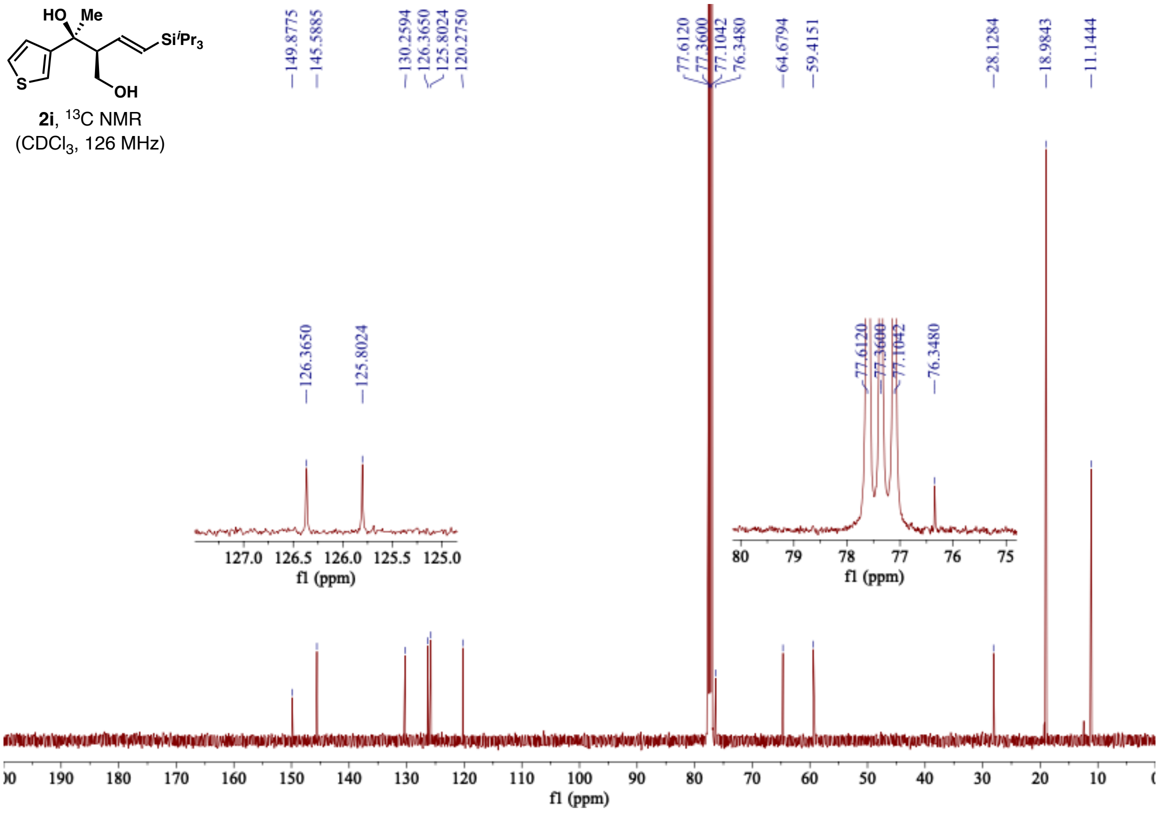

2i, ^{13}C NMR
(CDCl$_3$, 126 MHz)

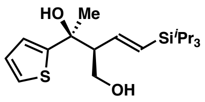

2j, ^{1}H NMR
(CDCl$_3$, 600 MHz)

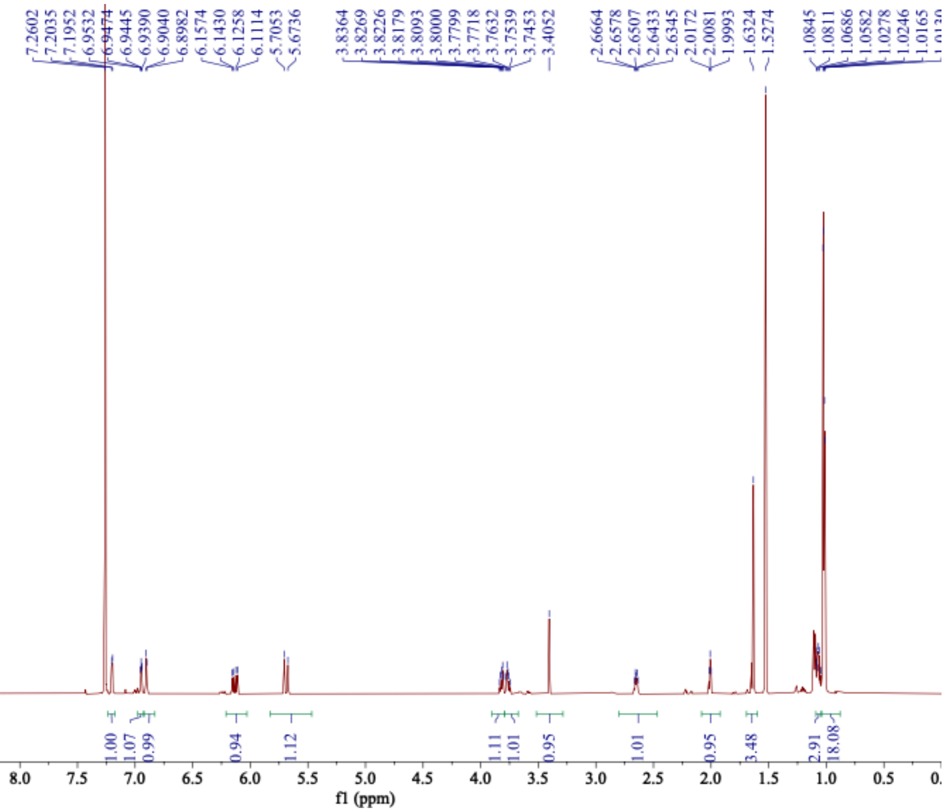

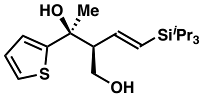

2j, ^{13}C NMR
(CDCl$_3$, 126 MHz)

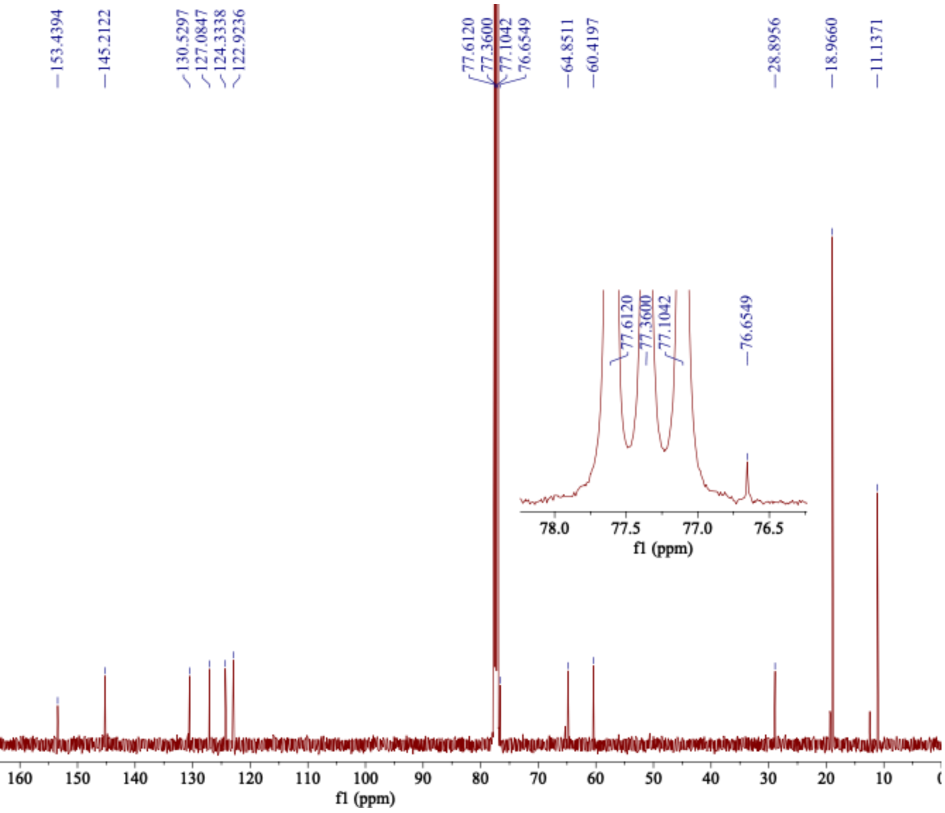

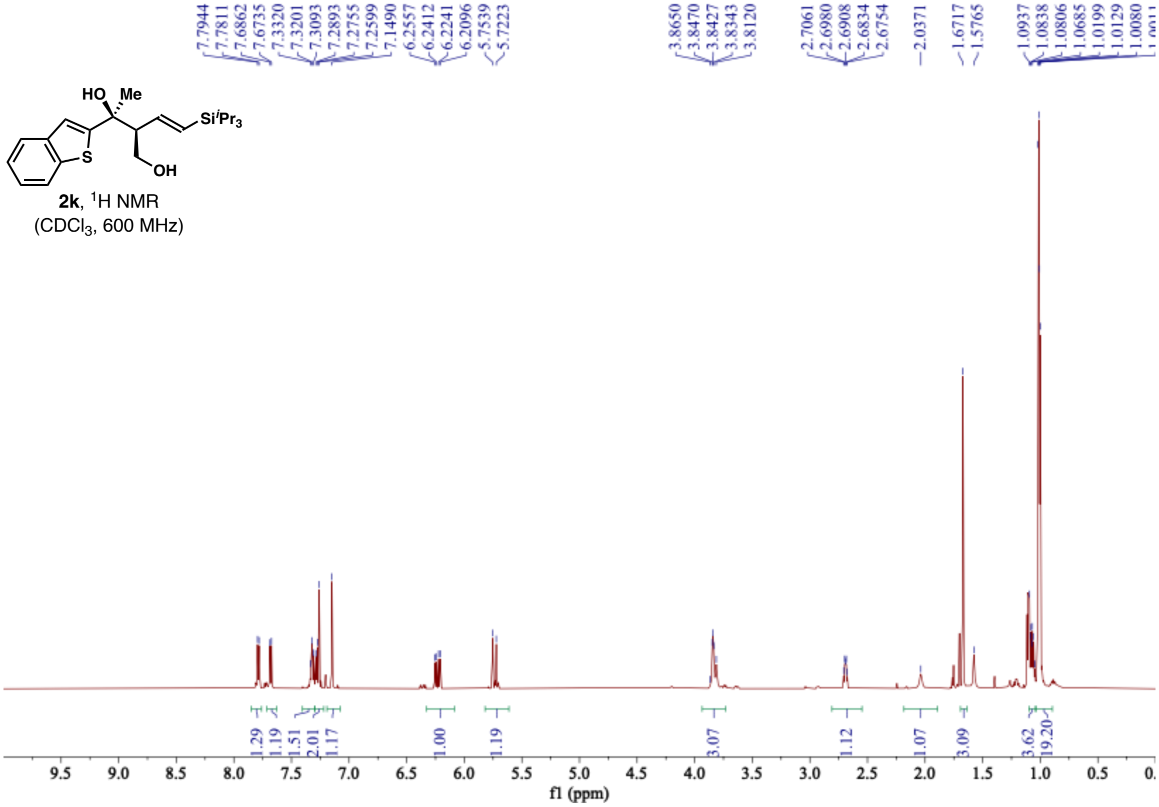

2k, ^{1}H NMR
(CDCl$_3$, 600 MHz)

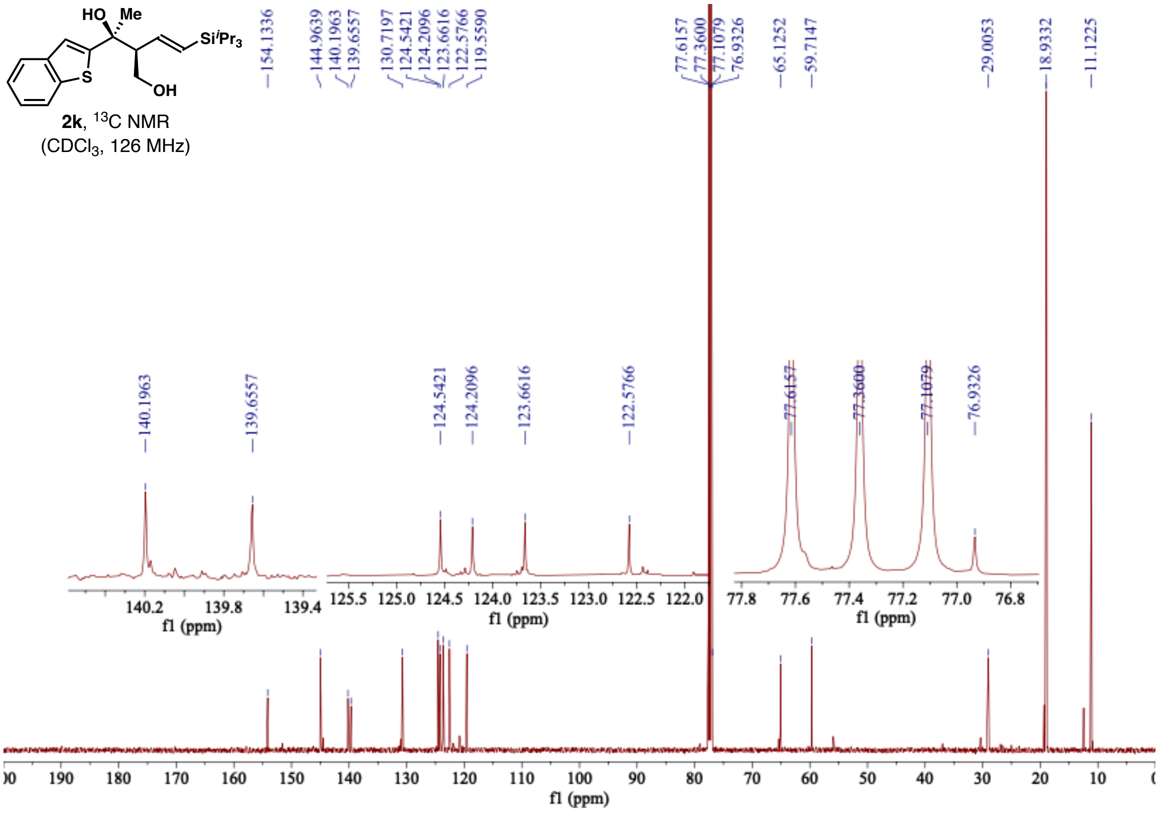

2k, ^{13}C NMR
(CDCl$_3$, 126 MHz)

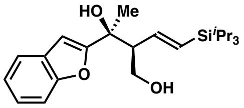

2l, ^{1}H NMR
($CDCl_3$, 600 MHz)

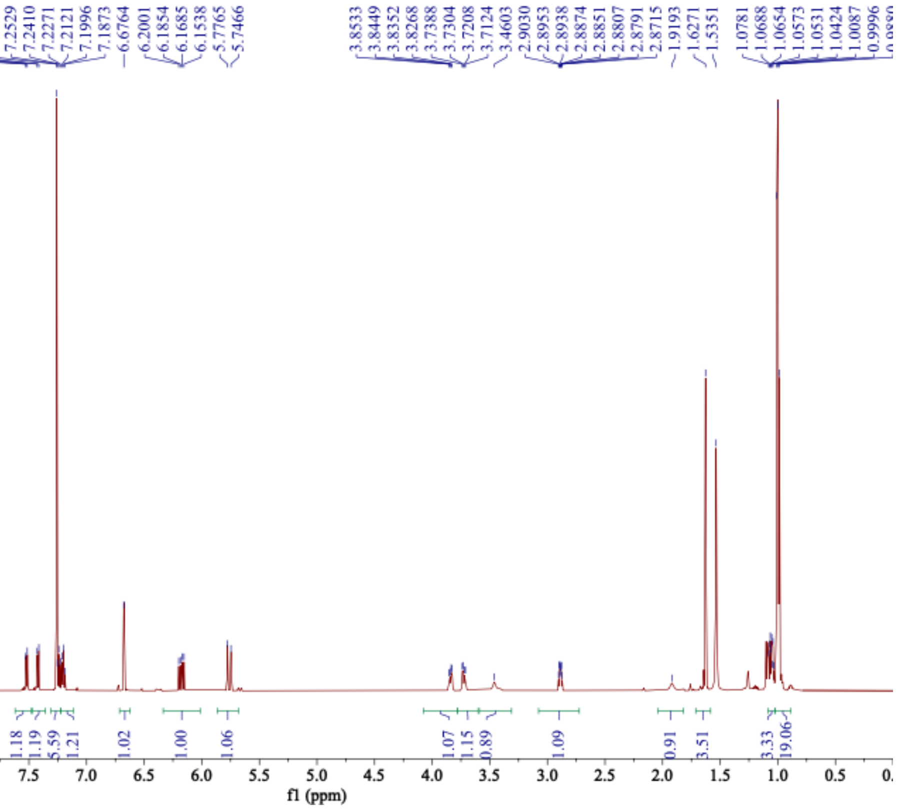

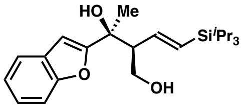

2l, ^{13}C NMR
($CDCl_3$, 126 MHz)

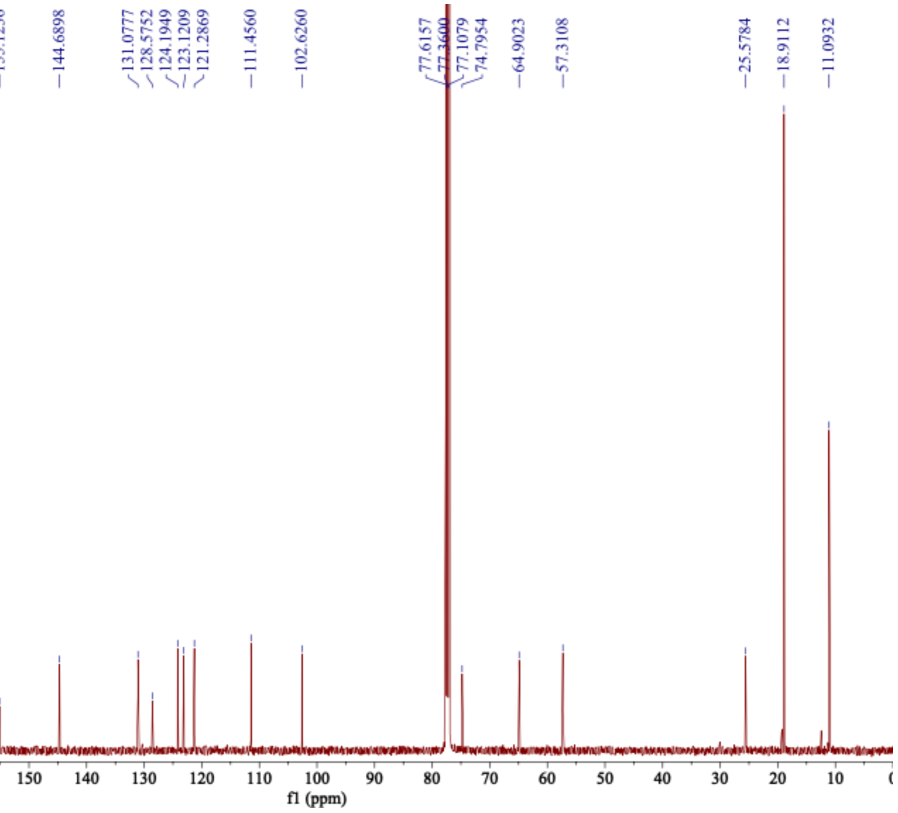

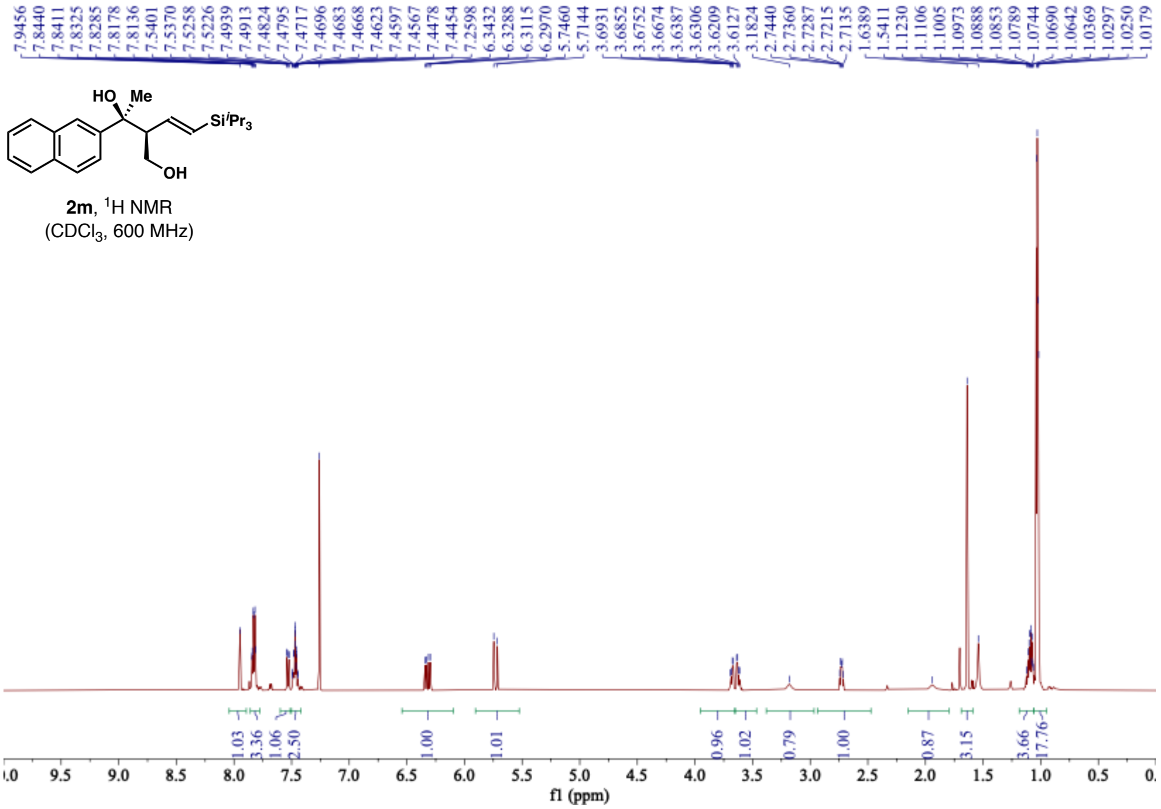

2m, ^{1}H NMR
($CDCl_3$, 600 MHz)

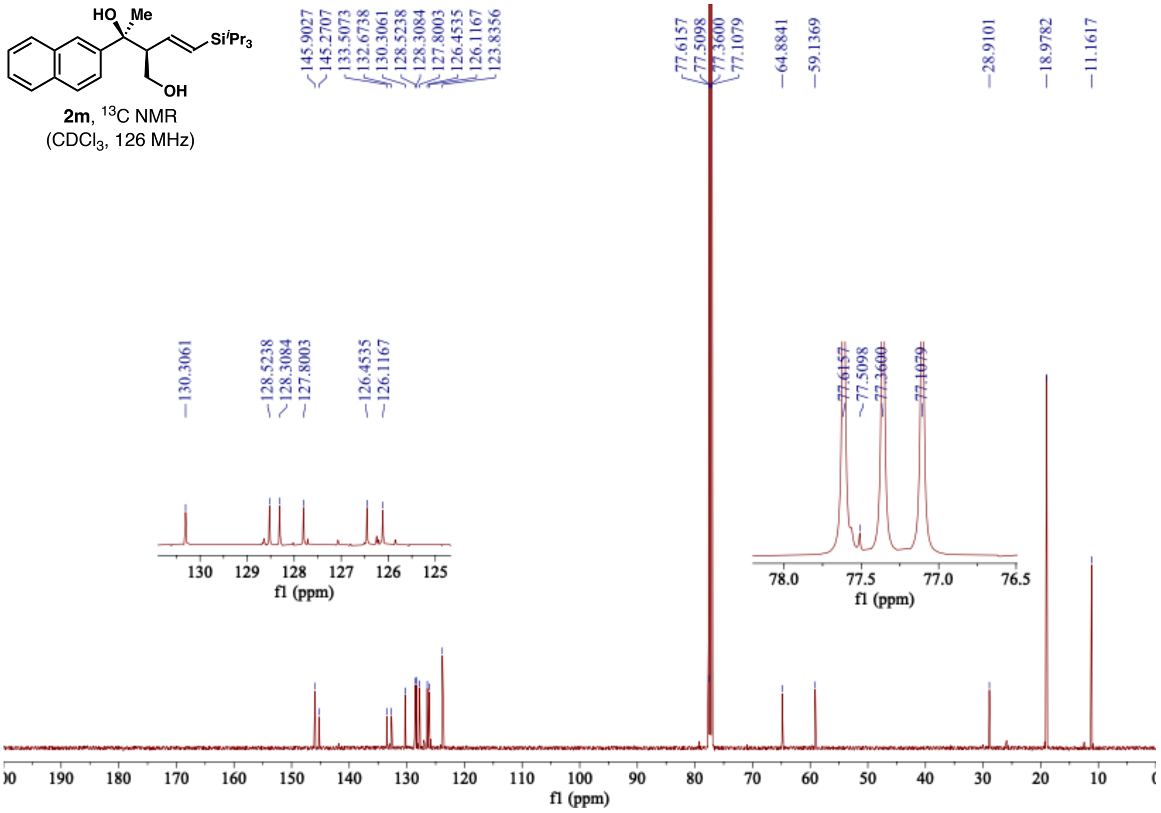

2m, ^{13}C NMR
($CDCl_3$, 126 MHz)

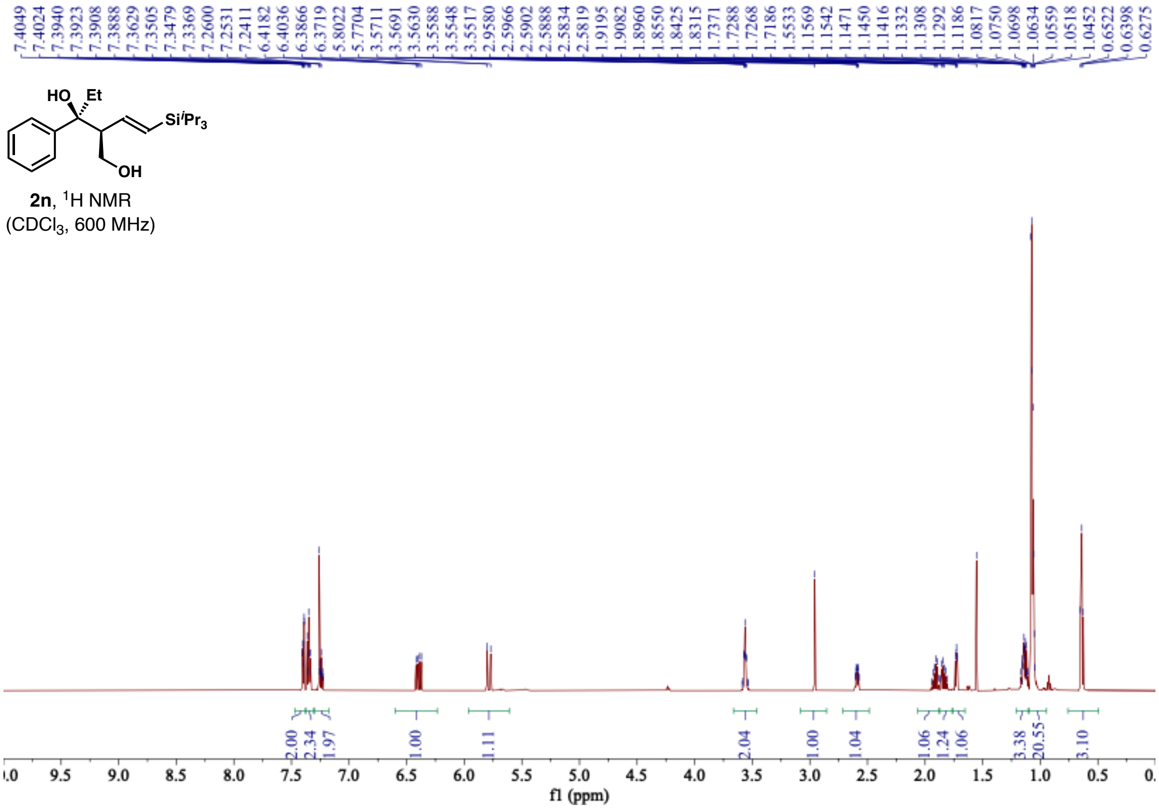

2n, ^{1}H NMR
(CDCl$_3$, 600 MHz)

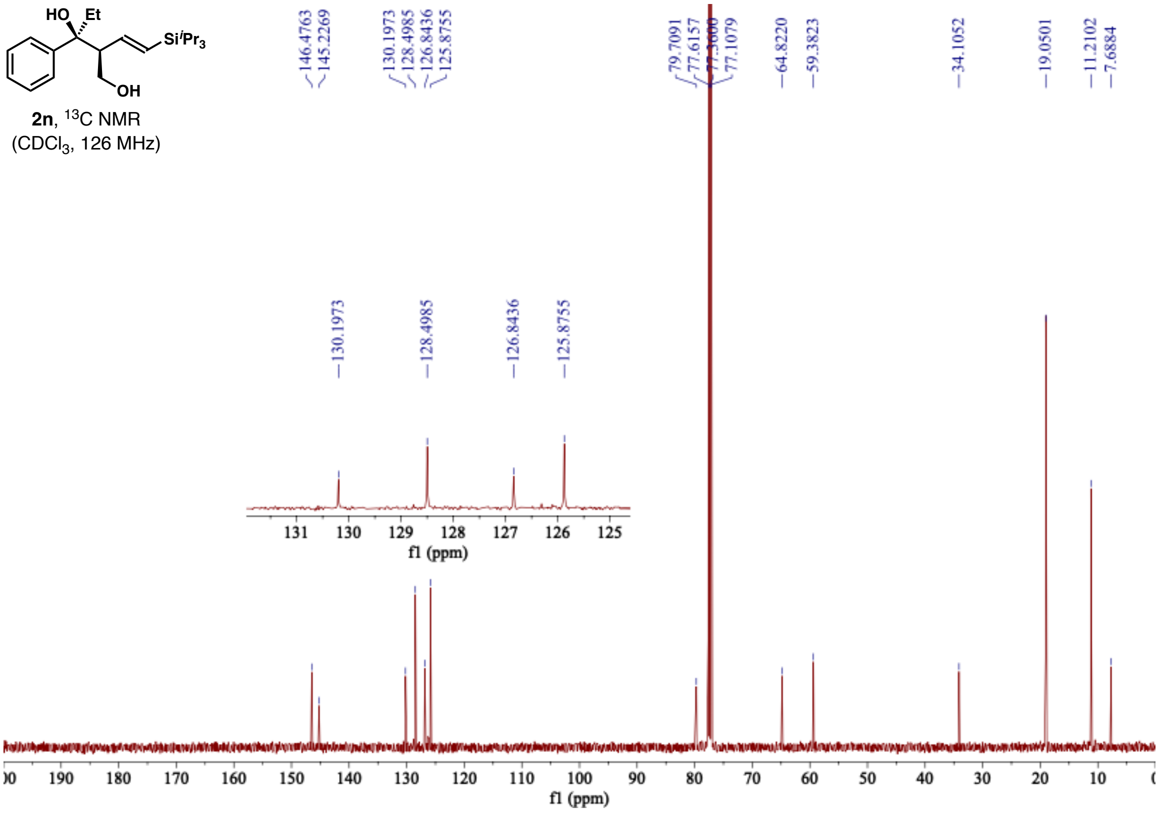

2n, ^{13}C NMR
(CDCl$_3$, 126 MHz)

2o, ^{1}H NMR
($CDCl_3$, 600 MHz)

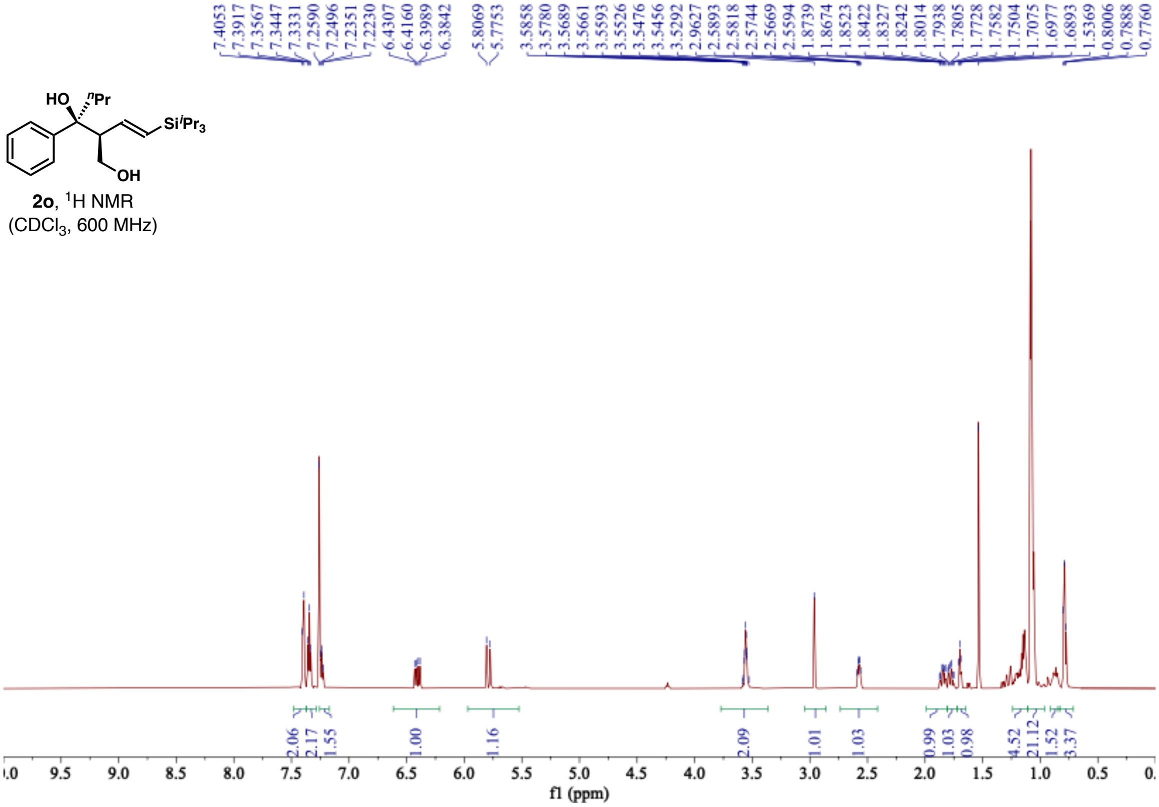

2o, ^{13}C NMR
($CDCl_3$, 126 MHz)

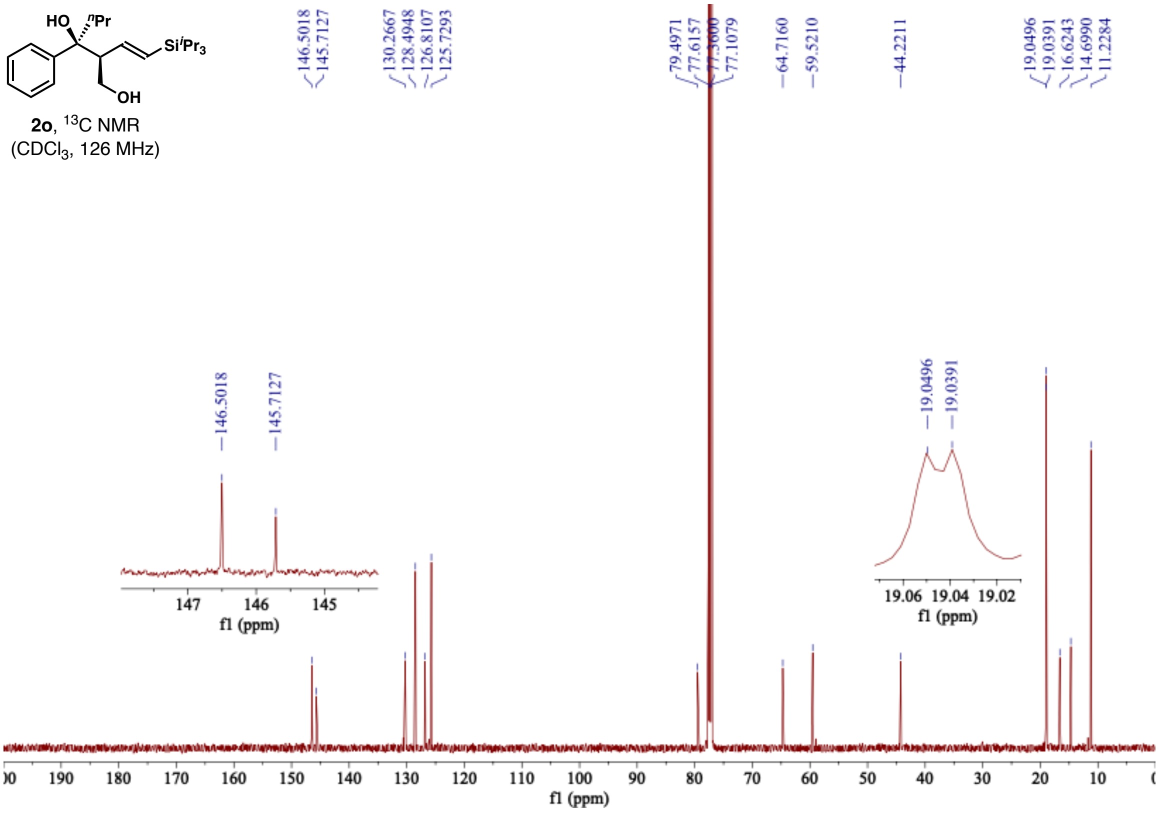

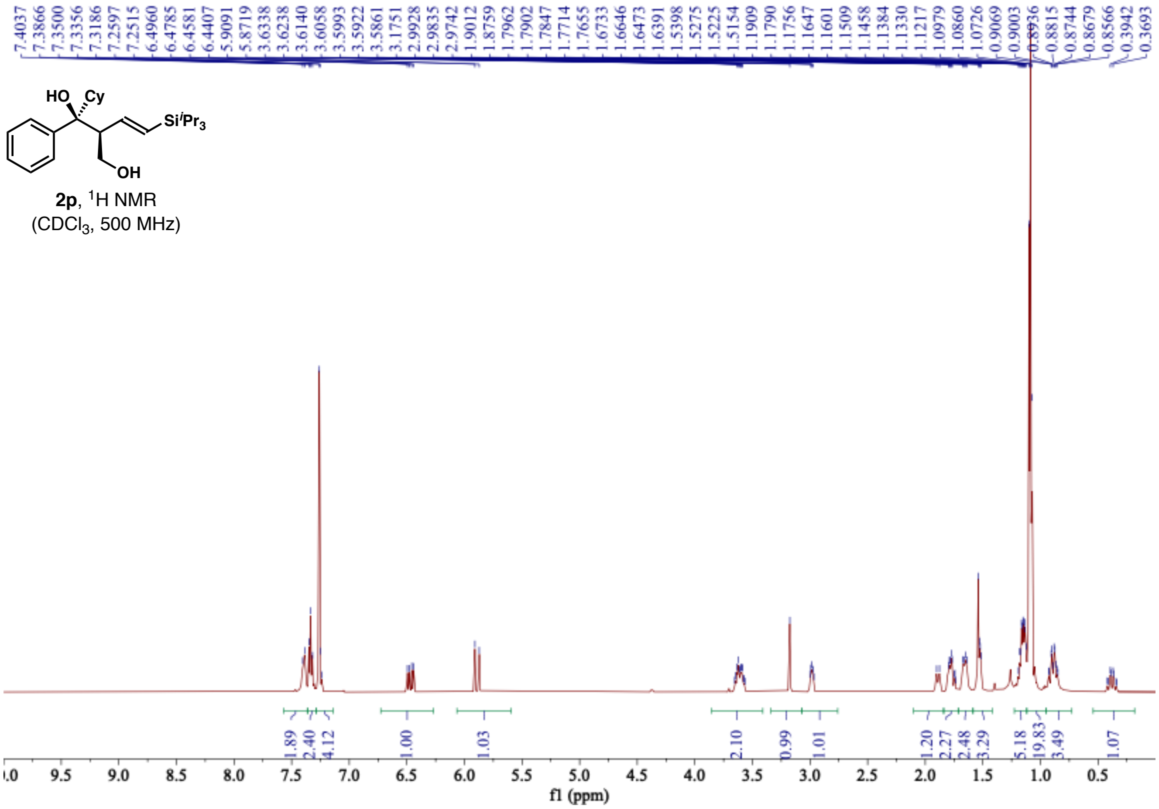
7.4037 7.3866 7.3500 7.3356 7.3186 7.2597 7.2515 6.4960 6.4785 6.4581 6.4407 5.9091 5.8719 3.6338 3.6238 3.6140 3.6058 3.5993 3.5922 3.5861 3.1751 2.9928 2.9835 2.9742 1.9012 1.8759 1.7962 1.7902 1.7847 1.7714 1.7655 1.6733 1.6646 1.6473 1.6391 1.5398 1.5275 1.5225 1.5154 1.1909 1.1790 1.1756 1.1647 1.1601 1.1509 1.1458 1.1384 1.1330 1.1217 1.0979 1.0860 1.0726 0.9069 0.9003 0.8936 0.8815 0.8744 0.8679 0.8566 0.3942 0.3693
HO
Cy
Si^{i}Pr$_3$
OH
2p, ^{1}H NMR
(CDCl$_3$, 500 MHz)
1.89 2.40 4.12 1.00 1.03 2.10 0.99 1.01 1.20 2.27 2.48 3.29 5.18 19.83 3.49 1.07
f1 (ppm)

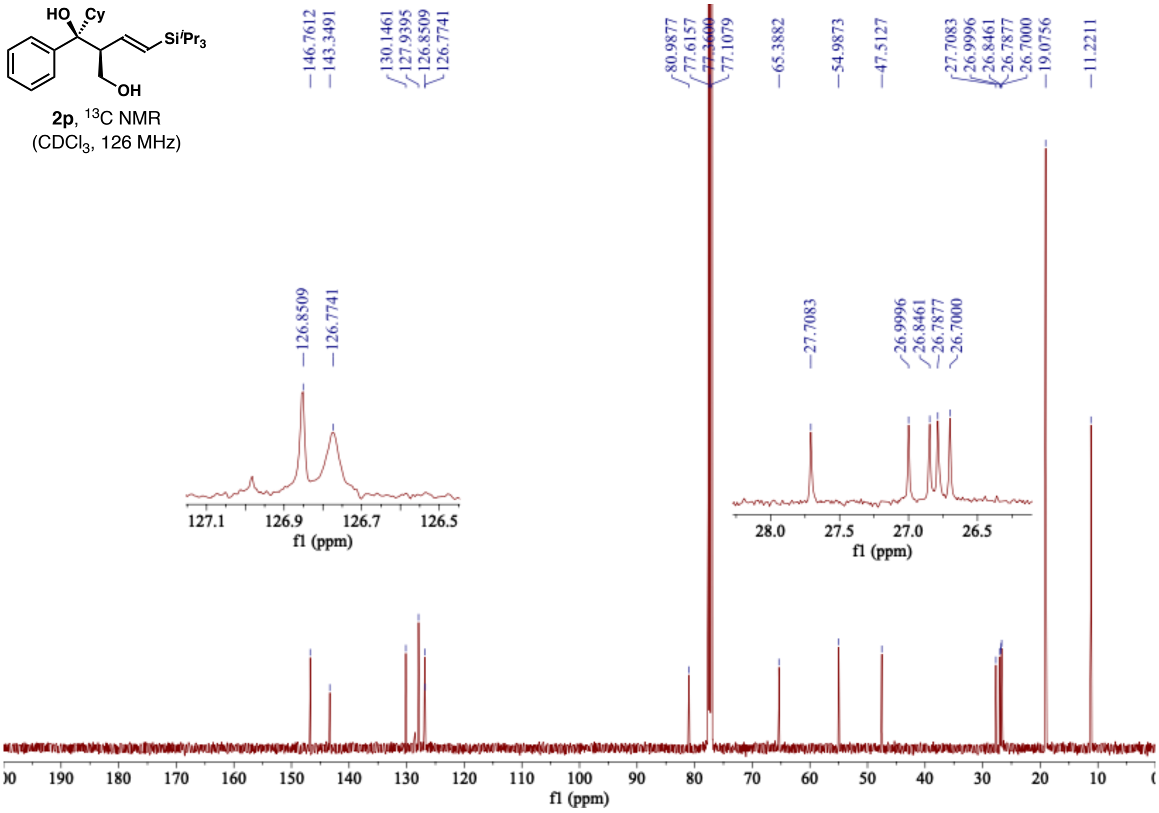
HO
Cy
Si^{i}Pr$_3$
OH
2p, ^{13}C NMR
(CDCl$_3$, 126 MHz)
146.7612 143.3491 130.1461 127.9395 126.8509 126.7741 80.9877 77.6157 77.3600 77.1079 65.3882 54.9873 47.5127 27.7083 26.9996 26.8461 26.7877 26.7000 19.0756 11.2211
126.8509 126.7741
f1 (ppm)
27.7083 26.9996 26.8461 26.7877 26.7000
f1 (ppm)
f1 (ppm)

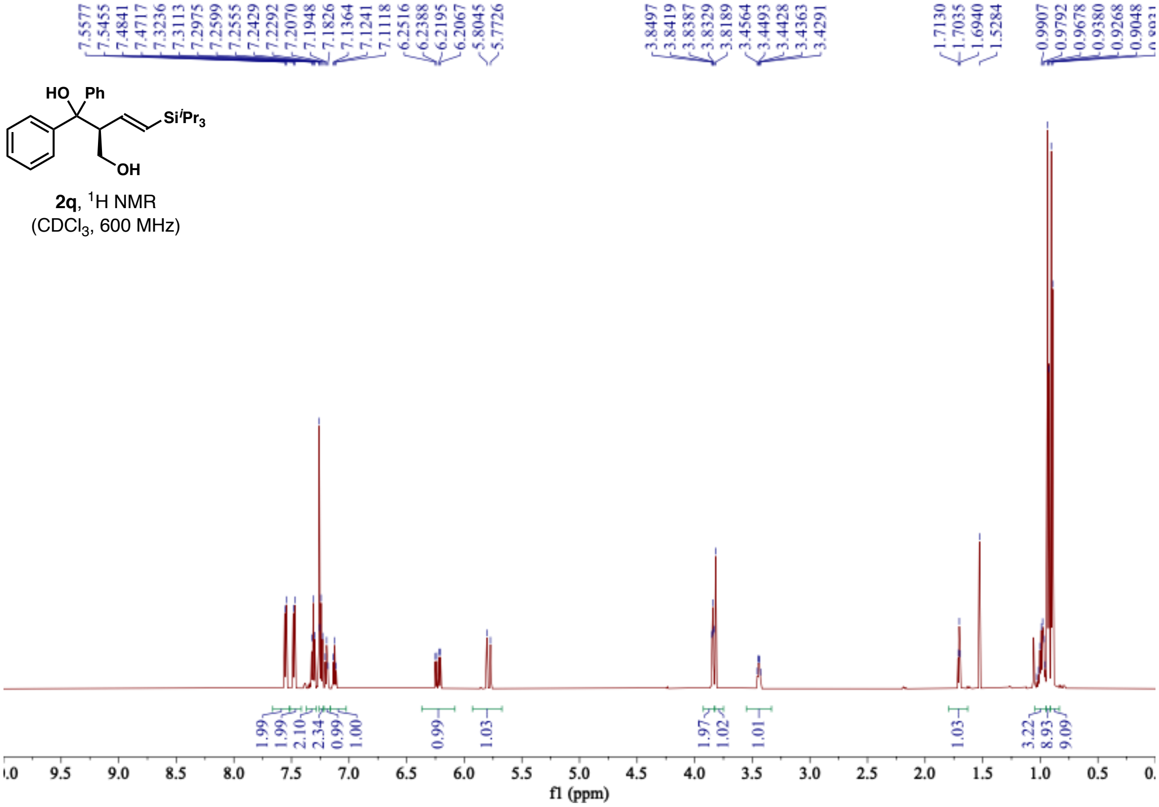

2q, ^{1}H NMR
($CDCl_3$, 600 MHz)

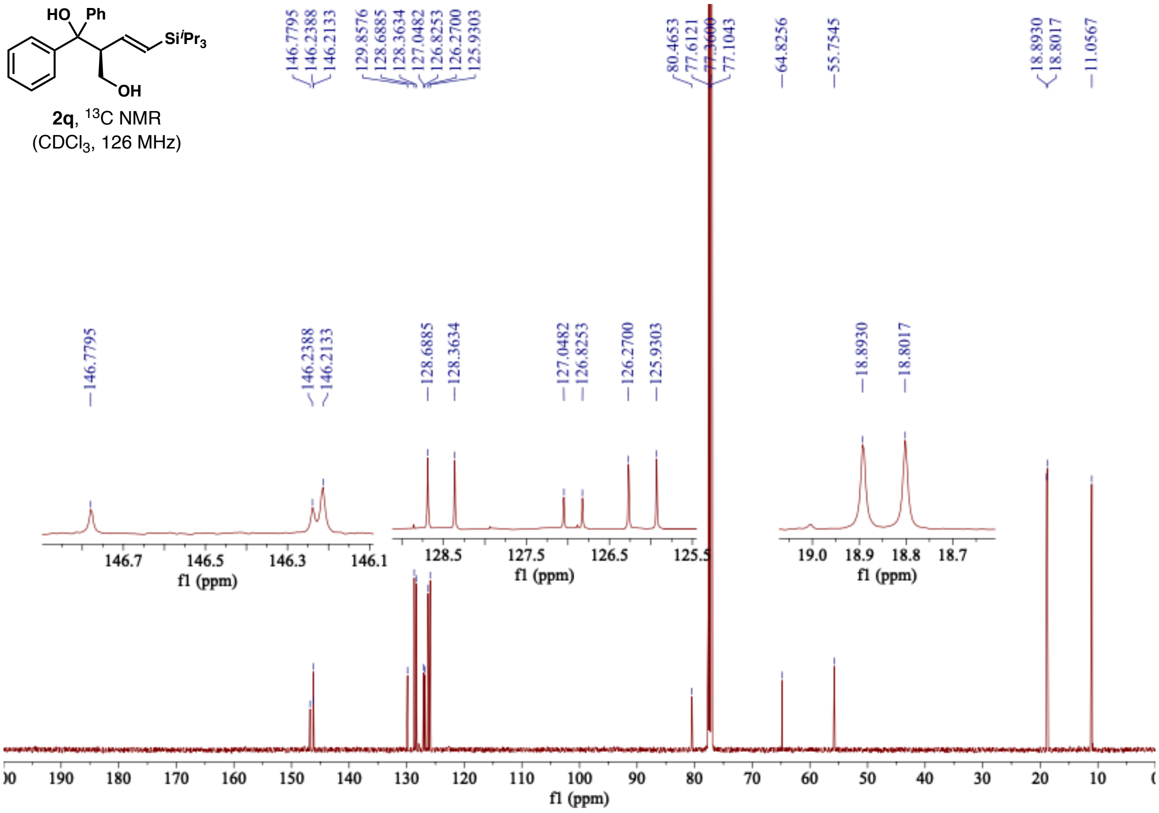

2q, ^{13}C NMR
($CDCl_3$, 126 MHz)

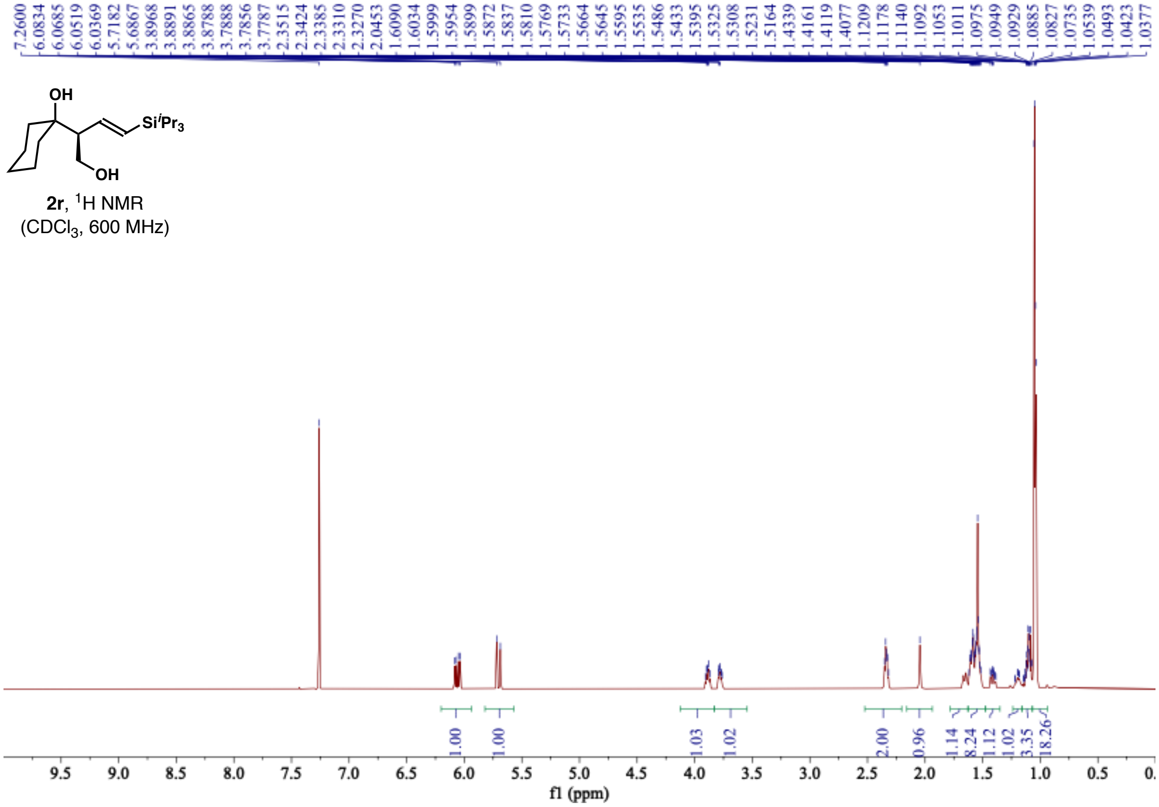

7.2600
6.0834
6.0685
6.0519
6.0369
5.7182
5.6867
3.8968
3.8891
3.8865
3.8788
3.7888
3.7856
3.7787
2.3515
2.3424
2.3385
2.3310
2.3270
2.0453
1.6090
1.6034
1.5999
1.5954
1.5899
1.5872
1.5837
1.5810
1.5769
1.5733
1.5664
1.5645
1.5595
1.5535
1.5486
1.5433
1.5395
1.5325
1.5308
1.5231
1.5164
1.4339
1.4161
1.4119
1.4077
1.1209
1.1178
1.1140
1.1092
1.1053
1.1011
1.0975
1.0949
1.0929
1.0885
1.0827
1.0735
1.0539
1.0493
1.0423
1.0377
OH
Si^{i}Pr$_3$
OH
2r, ^{1}H NMR
(CDCl$_3$, 600 MHz)
1.00
1.00
1.03
1.02
2.00
0.96
1.14
8.24
1.12
1.02
3.35
18.26
9.5 9.0 8.5 8.0 7.5 7.0 6.5 6.0 5.5 5.0 4.5 4.0 3.5 3.0 2.5 2.0 1.5 1.0 0.5
f1 (ppm)

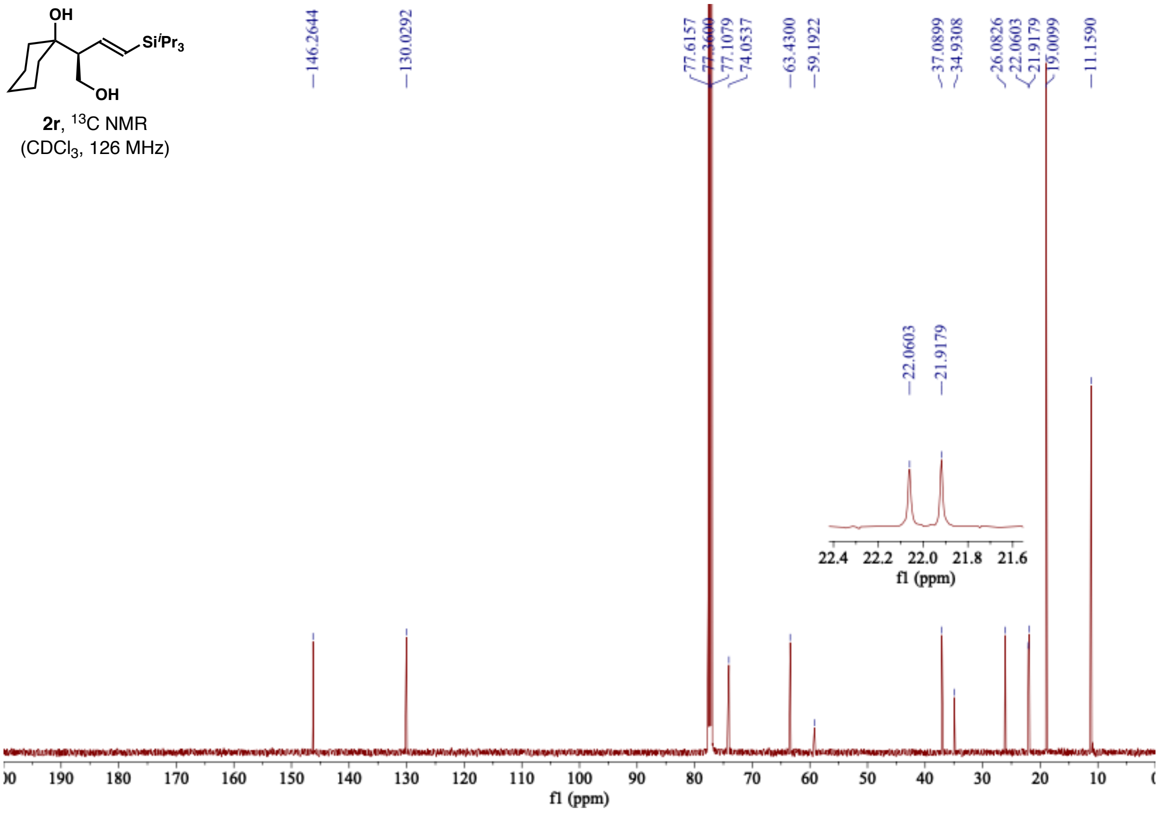

OH
Si^{i}Pr$_3$
OH
2r, ^{13}C NMR
(CDCl$_3$, 126 MHz)
146.2644
130.0292
77.6157
77.3600
77.1079
74.0537
63.4300
59.1922
37.0899
34.9308
26.0826
22.0603
21.9179
19.0099
11.1590
22.0603
21.9179
22.4 22.2 22.0 21.8 21.6
f1 (ppm)
190 180 170 160 150 140 130 120 110 100 90 80 70 60 50 40 30 20 10
f1 (ppm)

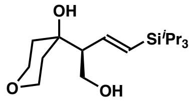

2s, ^{1}H NMR
(CDCl$_3$, 500 MHz)

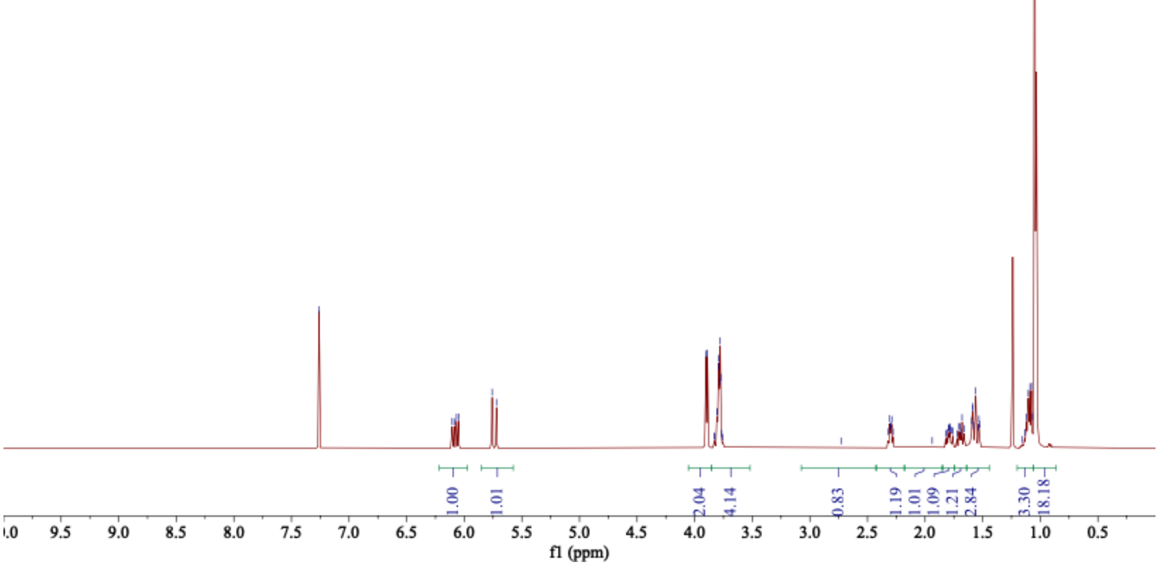

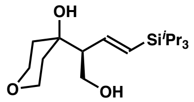

2s, ^{13}C NMR
(CDCl$_3$, 126 MHz)

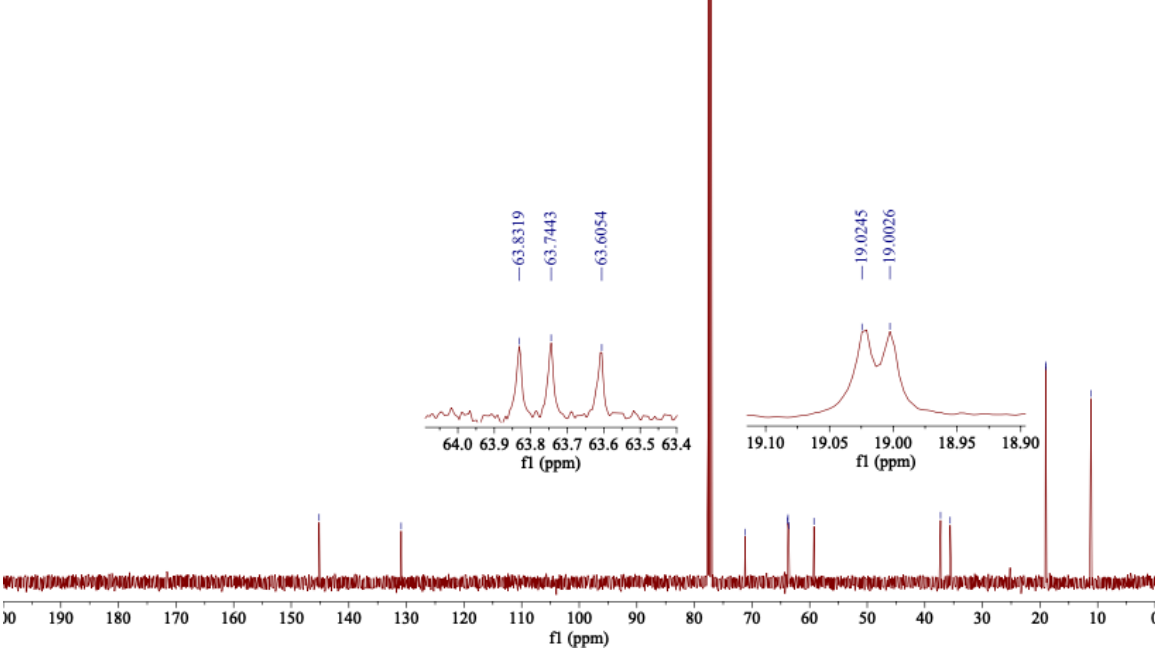

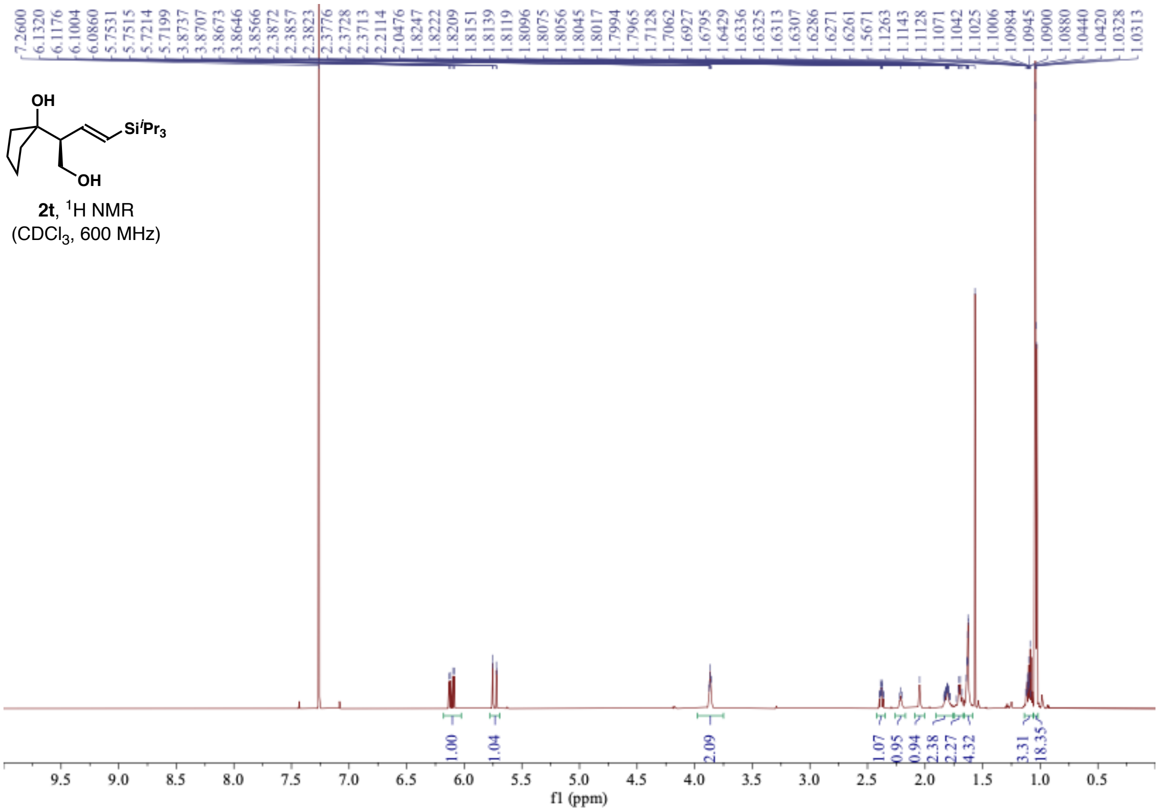

2t, ^{1}H NMR
(CDCl$_3$, 600 MHz)

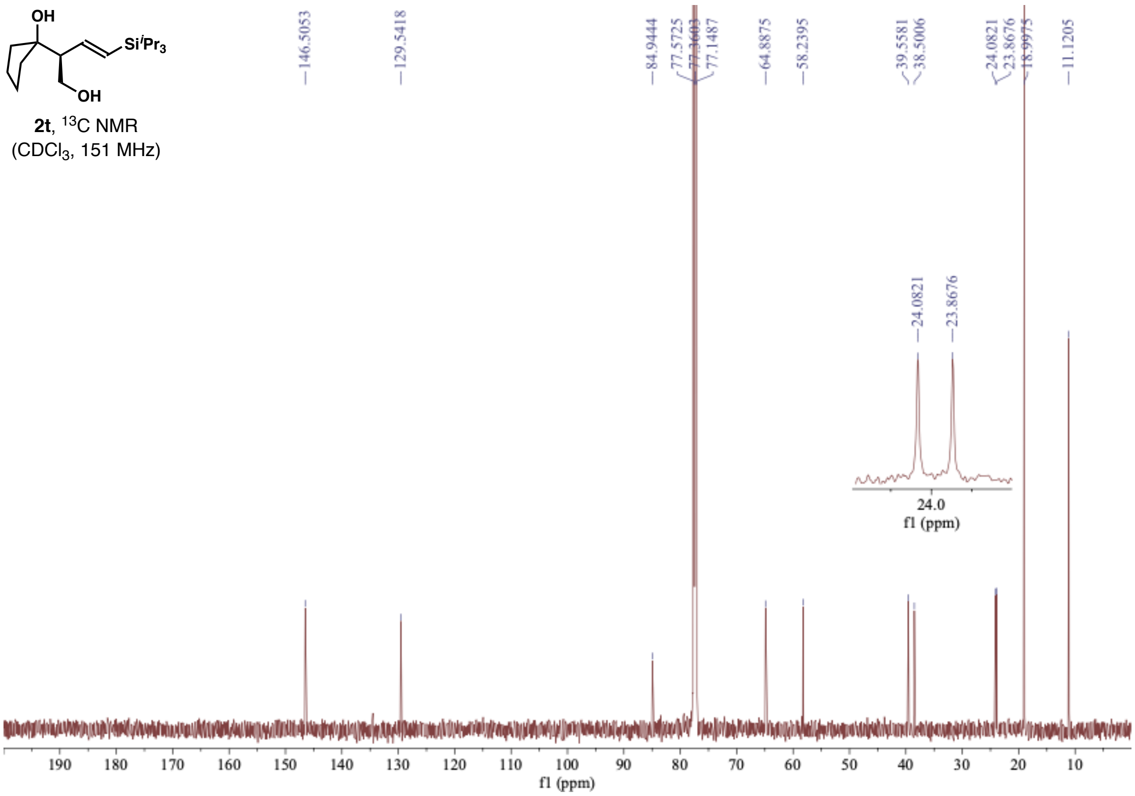

2t, ^{13}C NMR
(CDCl$_3$, 151 MHz)

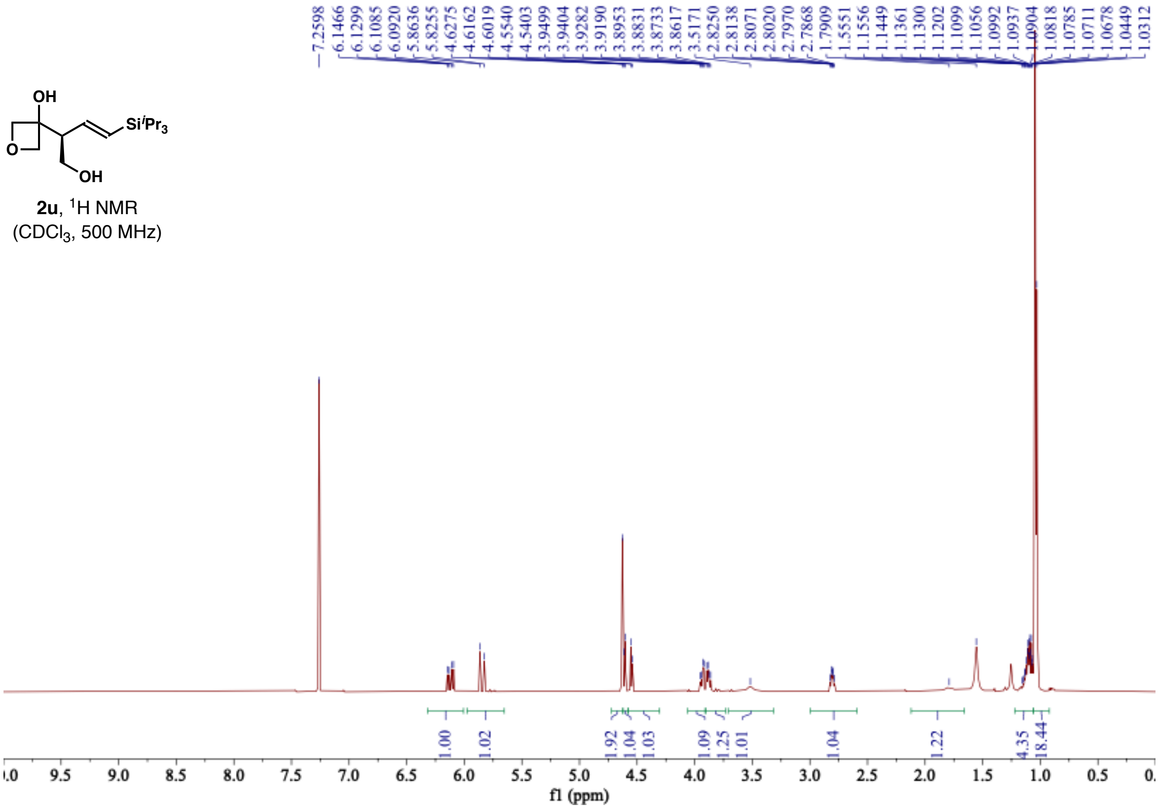

2u, ^{1}H NMR
($CDCl_3$, 500 MHz)

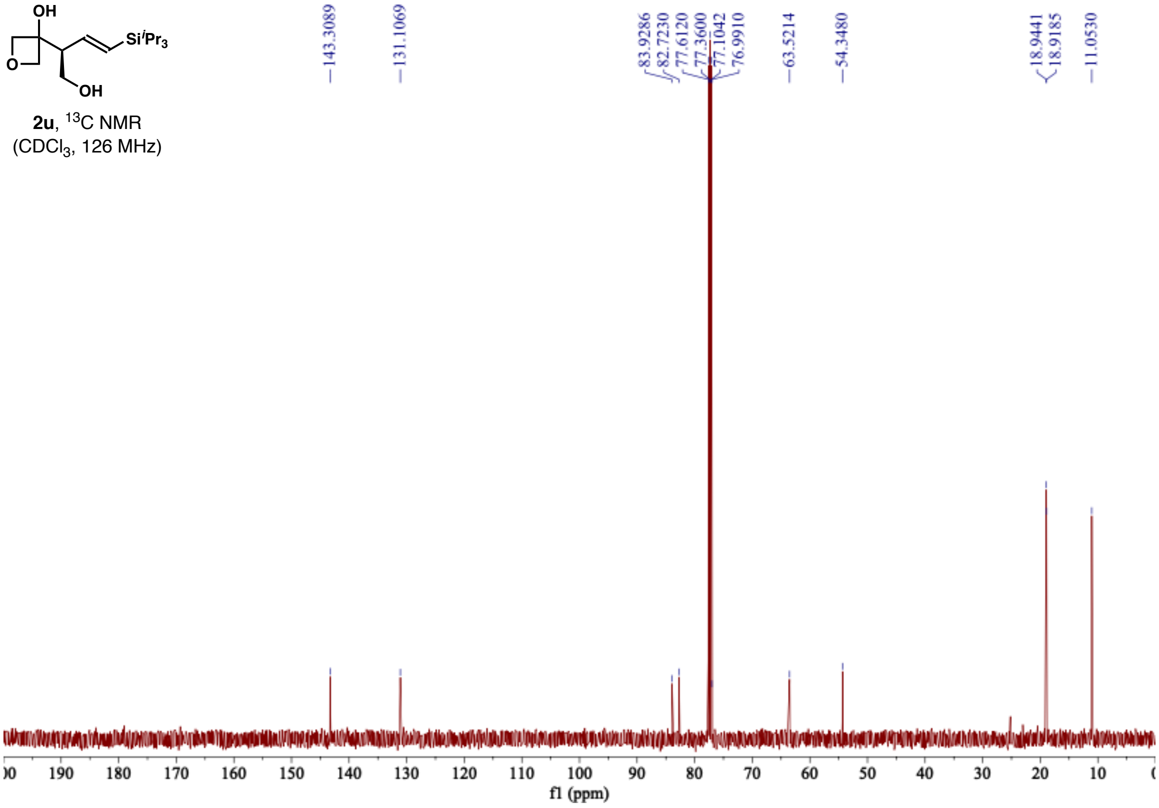

2u, ^{13}C NMR
($CDCl_3$, 126 MHz)

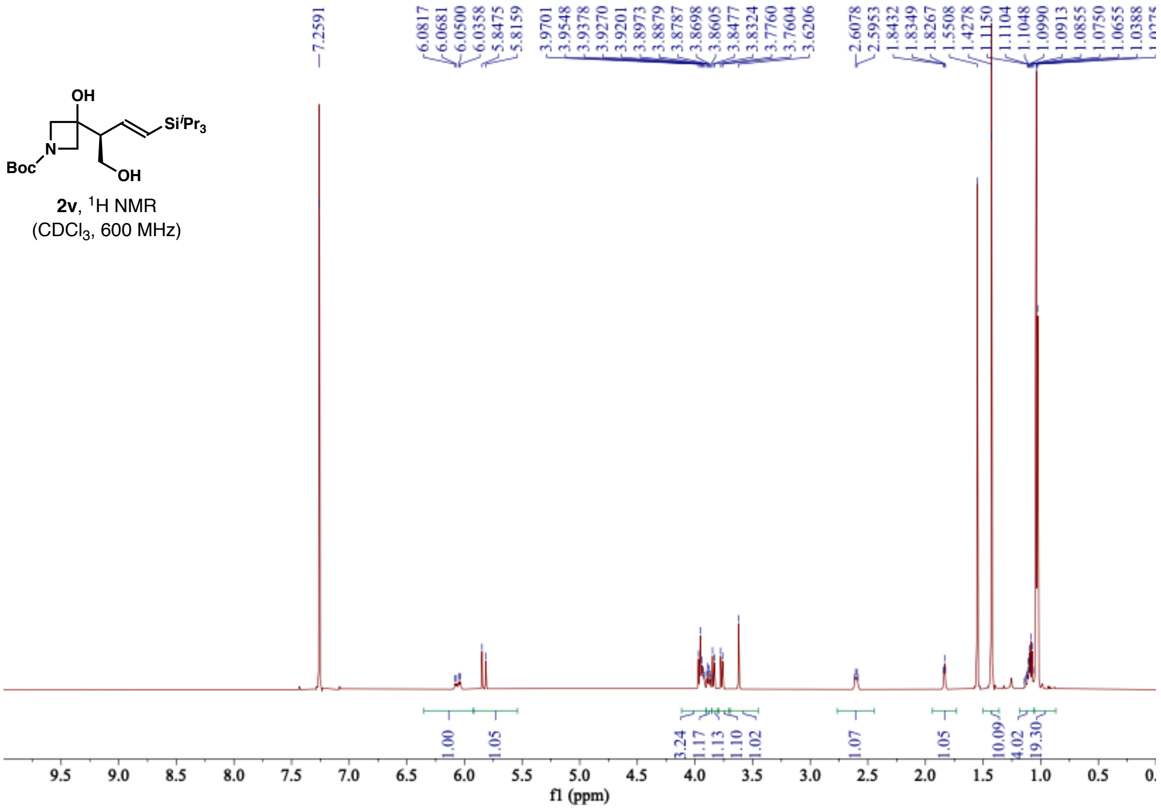

2v, ^{1}H NMR
($CDCl_3$, 600 MHz)

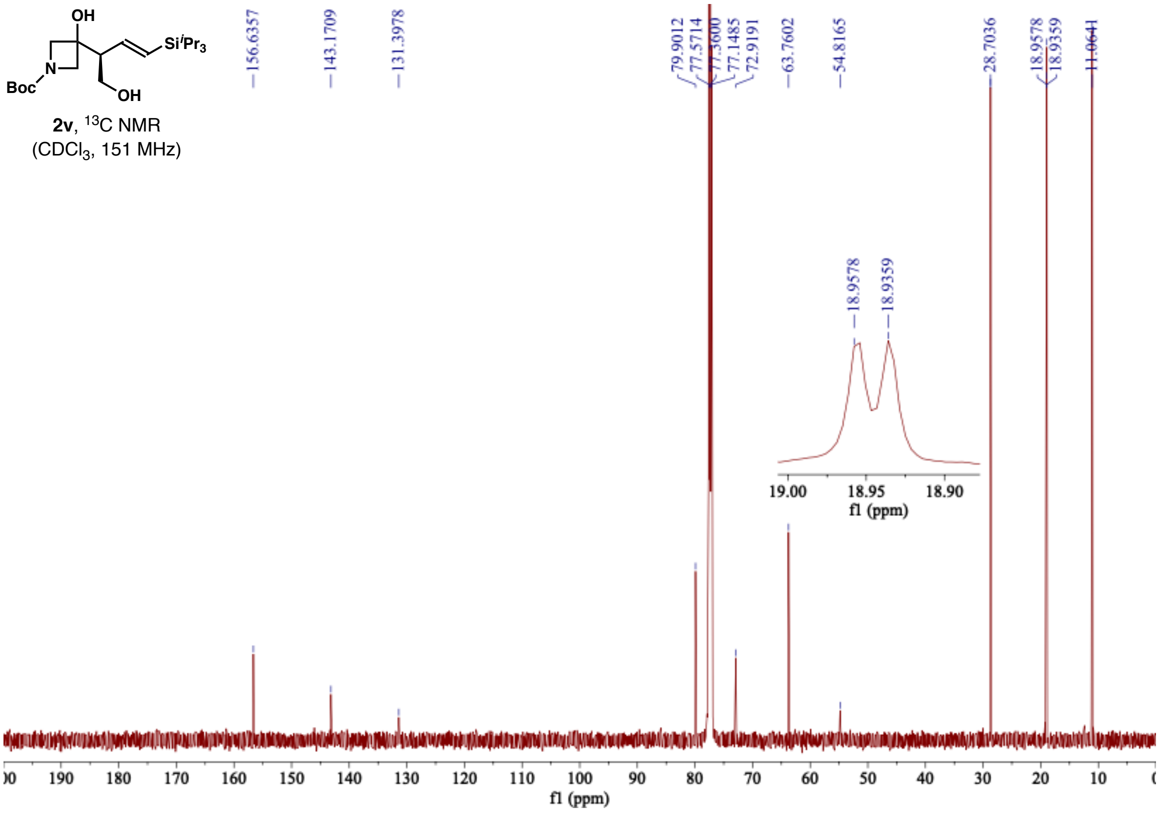

2v, ^{13}C NMR
($CDCl_3$, 151 MHz)

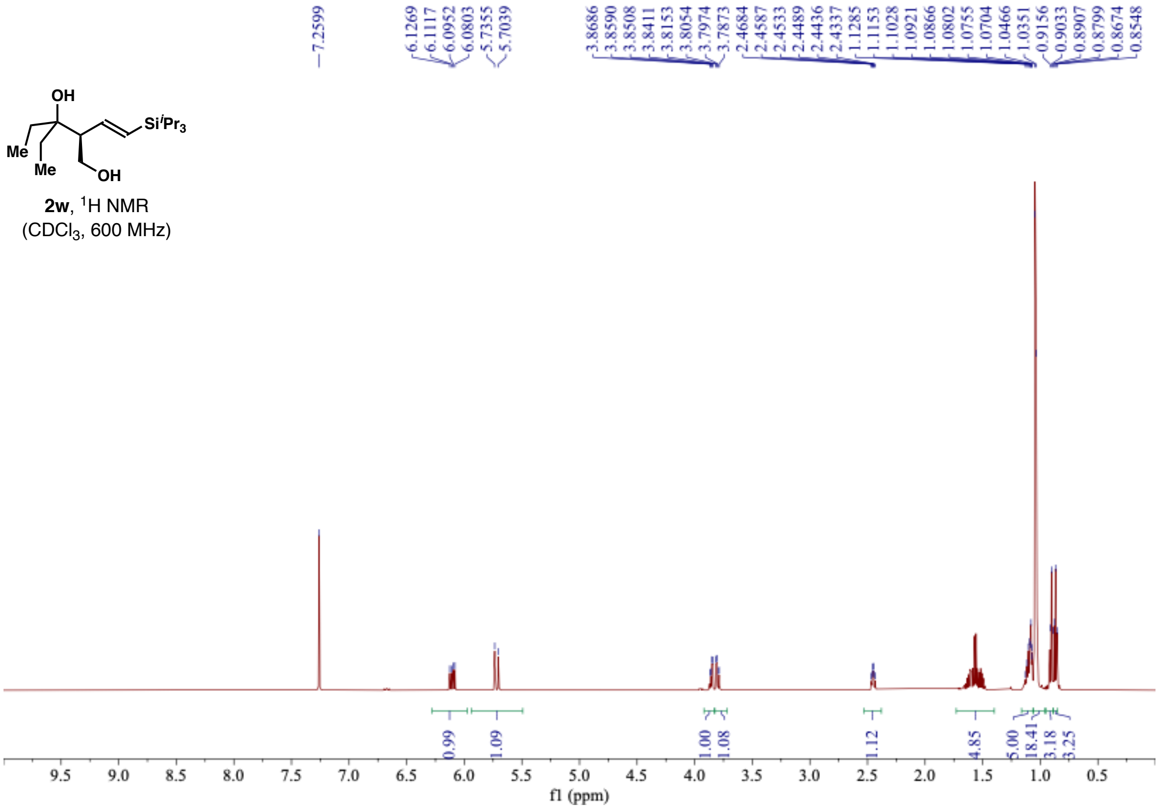

OH
Si^{i}Pr$_3$
Me
Me
OH
2w, ^{1}H NMR
(CDCl$_3$, 600 MHz)
7.2599
6.1269
6.1117
6.0952
6.0803
5.7355
5.7039
3.8686
3.8590
3.8508
3.8411
3.8153
3.8054
3.7974
3.7873
2.4684
2.4587
2.4533
2.4489
2.4436
2.4337
1.1285
1.1153
1.1028
1.0921
1.0866
1.0802
1.0755
1.0704
1.0466
1.0351
0.9156
0.9033
0.8907
0.8799
0.8674
0.8548
0.99
1.09
1.00
1.08
1.12
4.85
5.00
18.41
3.18
3.25
f1 (ppm)

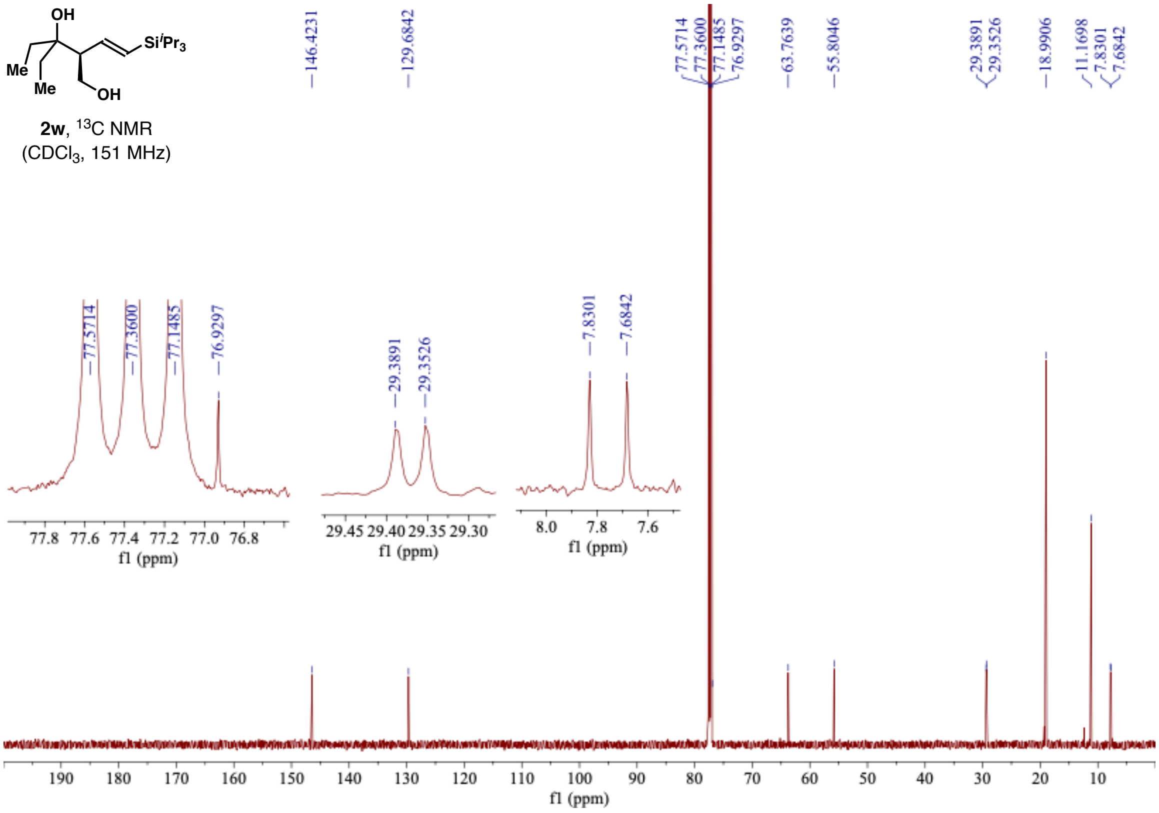

OH
Si^{i}Pr$_3$
Me
Me
OH
2w, ^{13}C NMR
(CDCl$_3$, 151 MHz)
146.4231
129.6842
77.5714
77.3600
77.1485
76.9297
63.7639
55.8046
29.3891
29.3526
18.9906
11.1698
7.8301
7.6842
f1 (ppm)

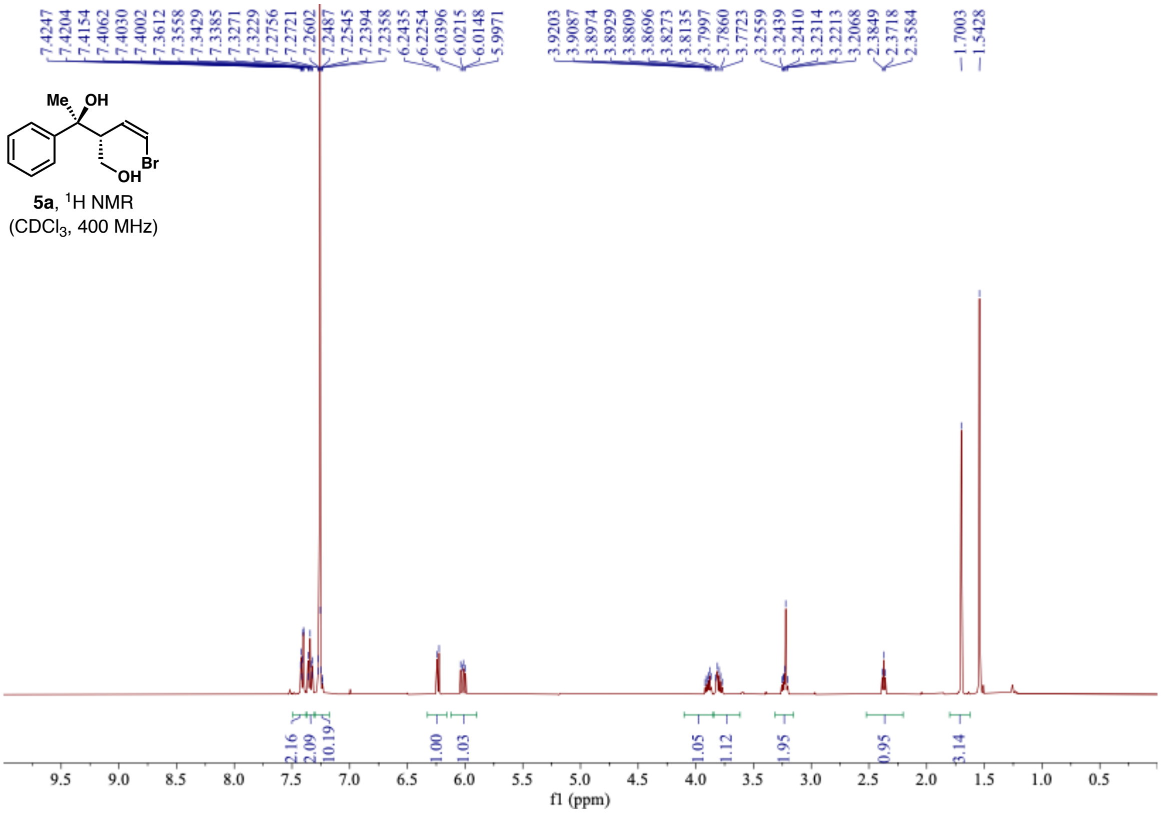

5a, ^{1}H NMR
($CDCl_3$, 400 MHz)

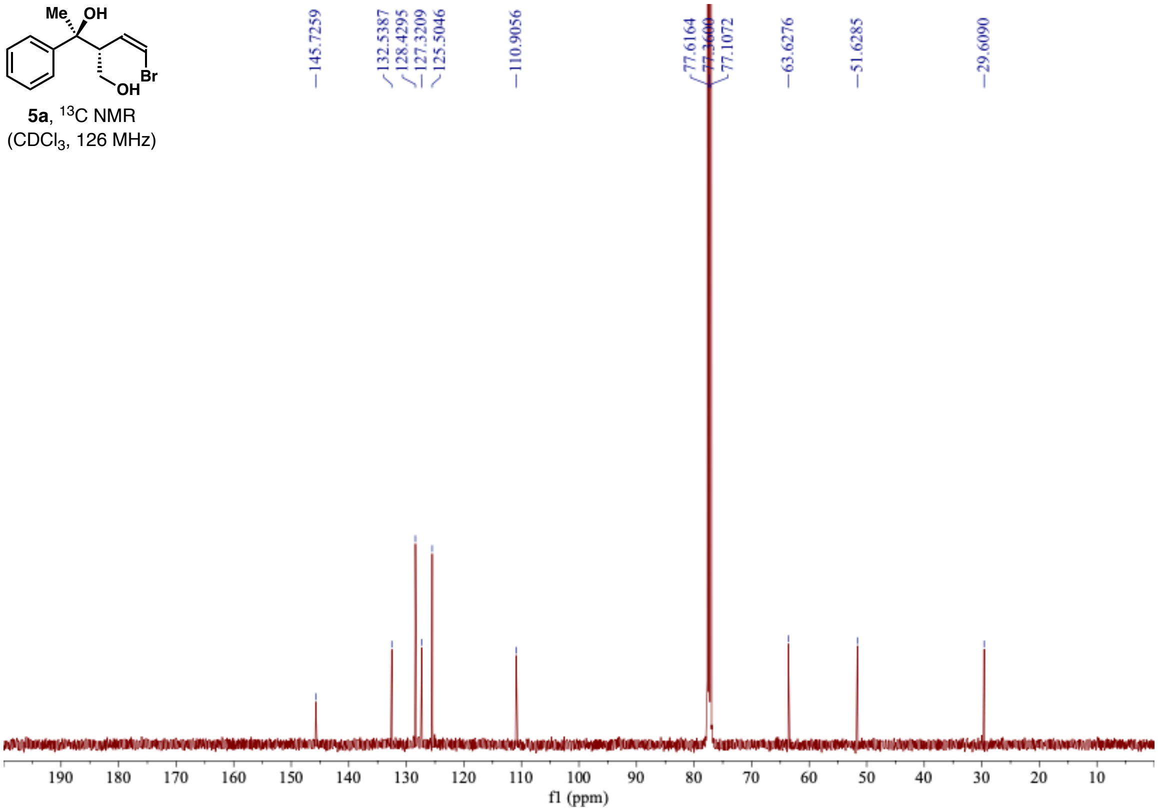

5a, ^{13}C NMR
($CDCl_3$, 126 MHz)

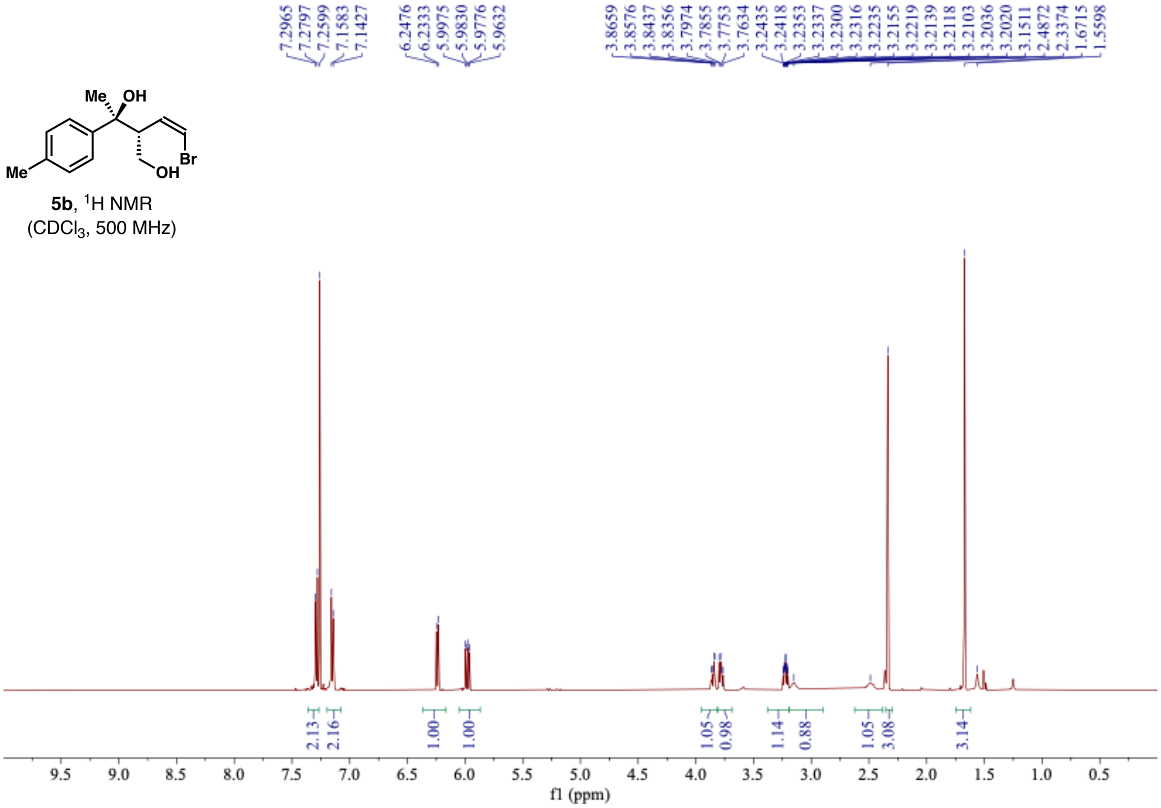

5b, ^{1}H NMR
($CDCl_3$, 500 MHz)

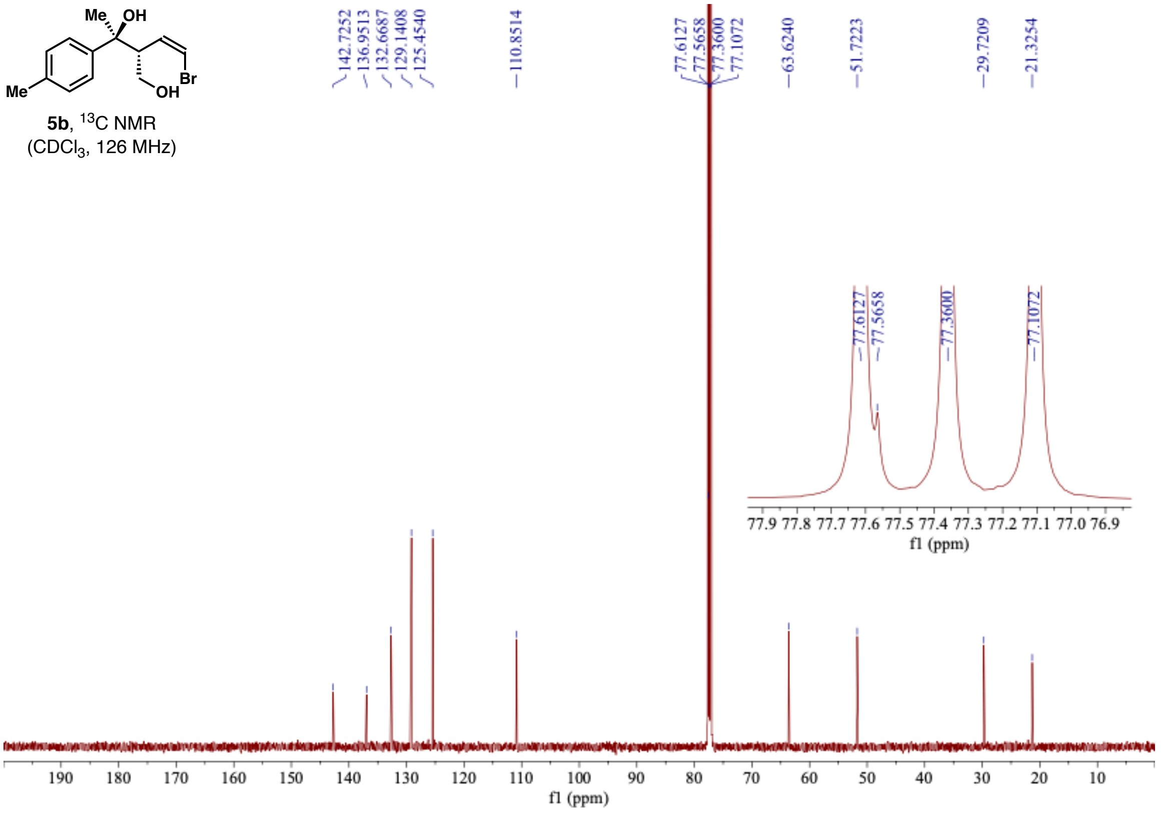

5b, ^{13}C NMR
($CDCl_3$, 126 MHz)

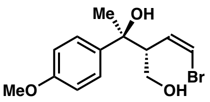

5c, ^{1}H NMR
($CDCl_3$, 400 MHz)

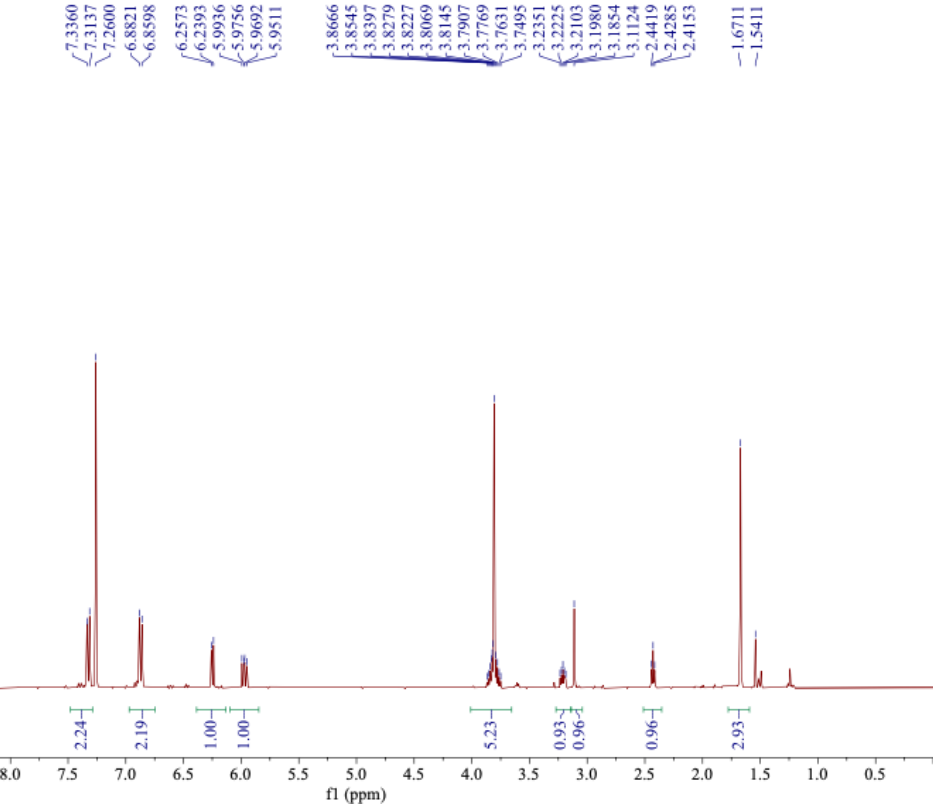

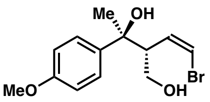

5c, ^{13}C NMR
($CDCl_3$, 126 MHz)

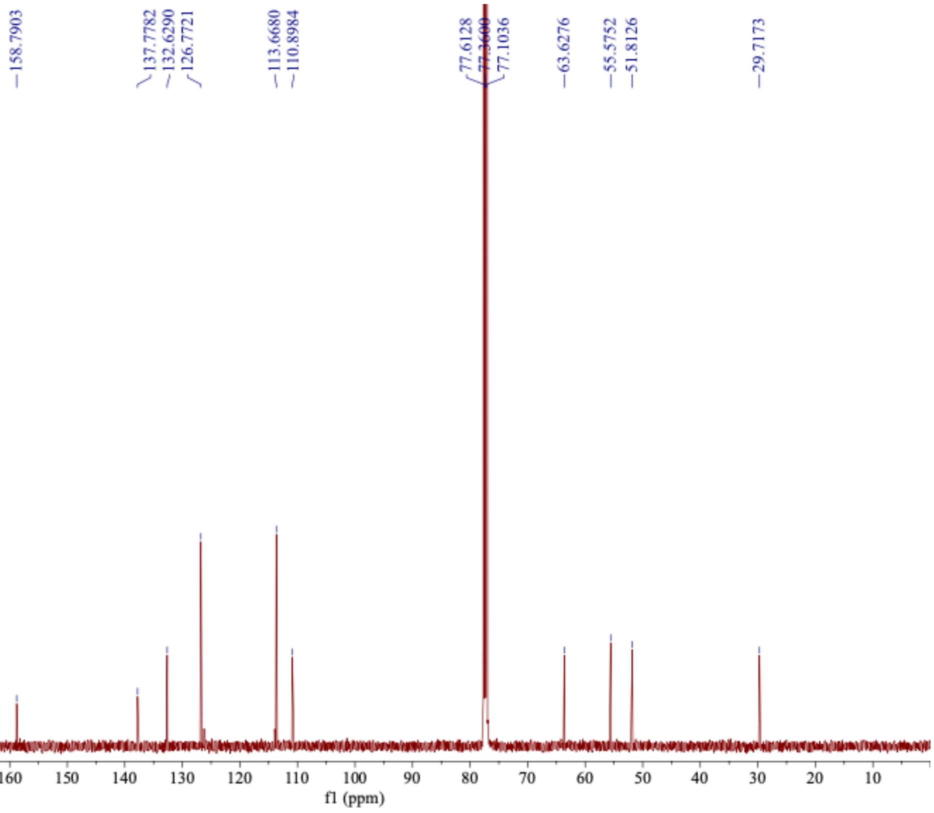

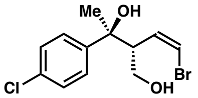

5d, ¹H NMR
(CDCl$_3$, 500 MHz)

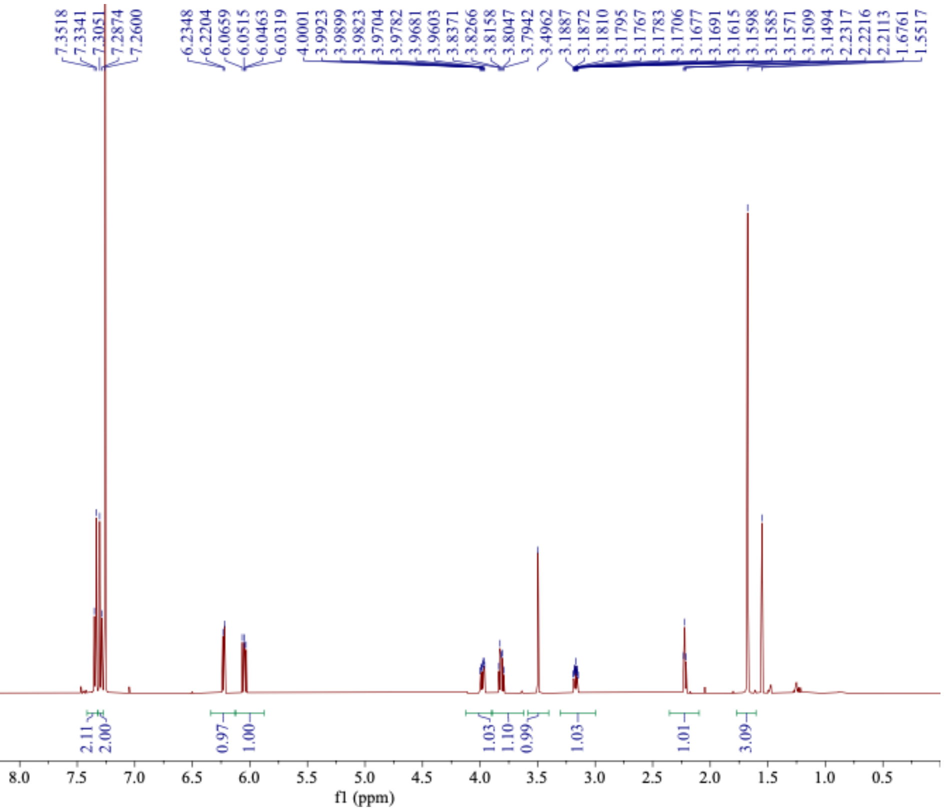

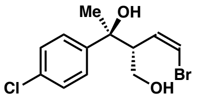

5d, ¹³C NMR
(CDCl$_3$, 126 MHz)

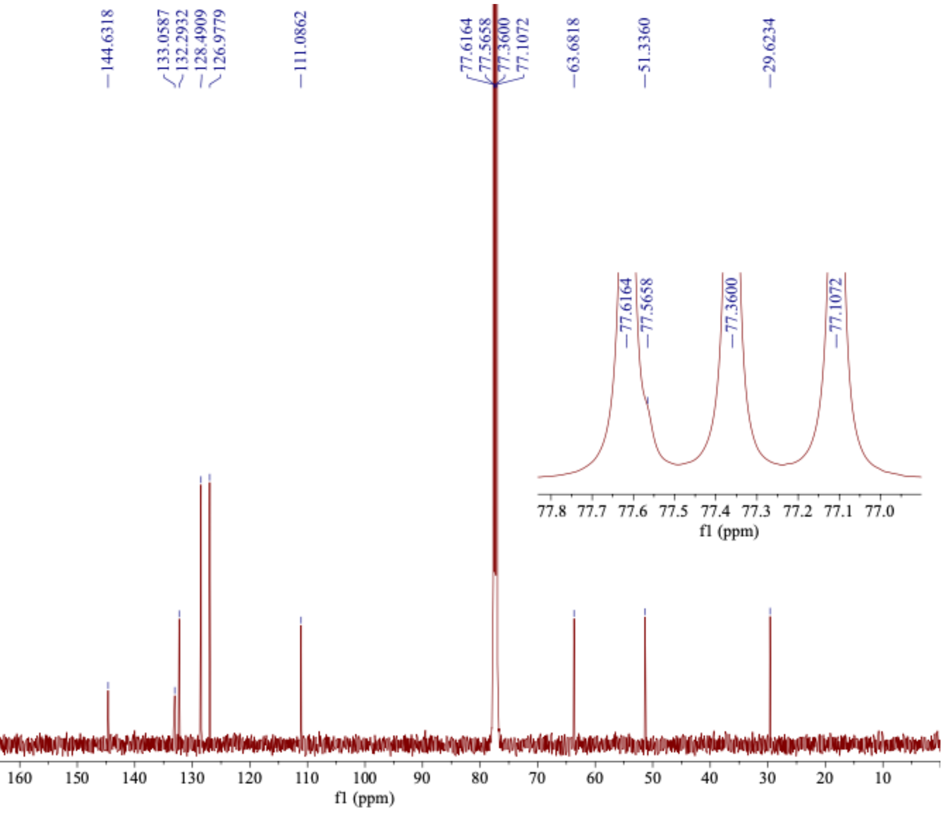

5e, ^{1}H NMR
($CDCl_3$, 500 MHz)

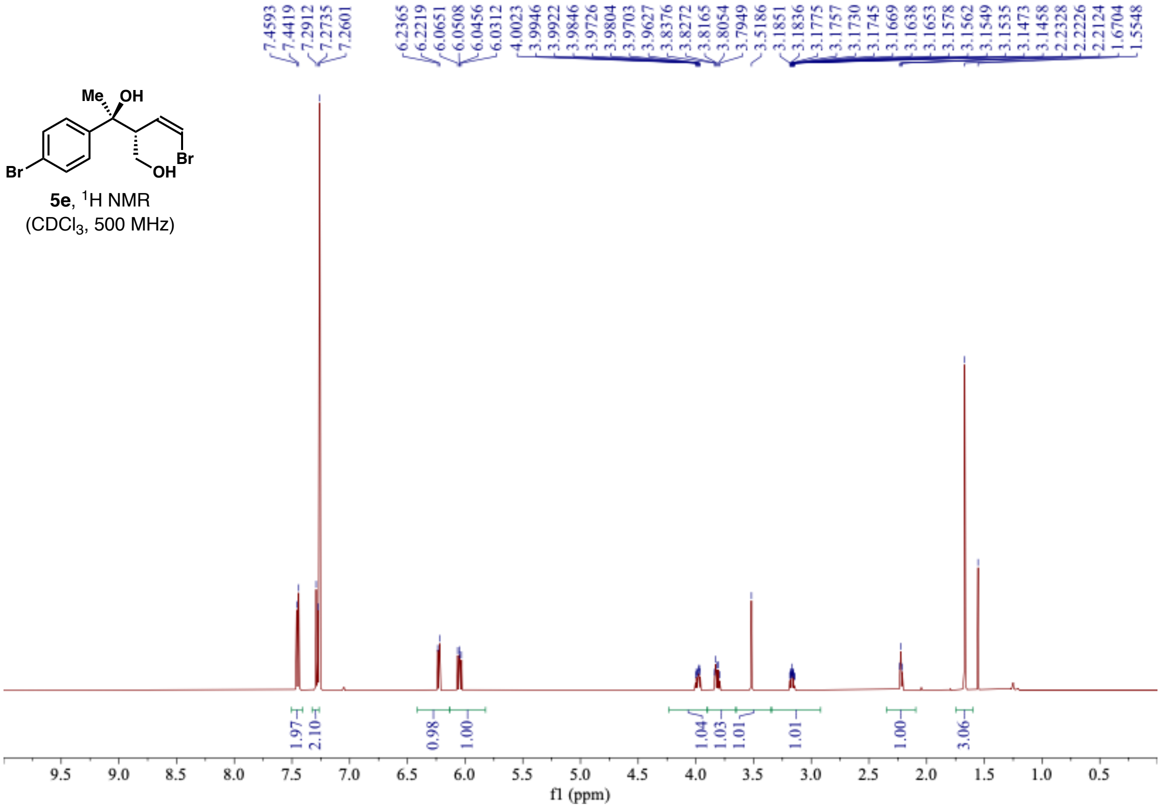

5e, ^{13}C NMR
($CDCl_3$, 126 MHz)

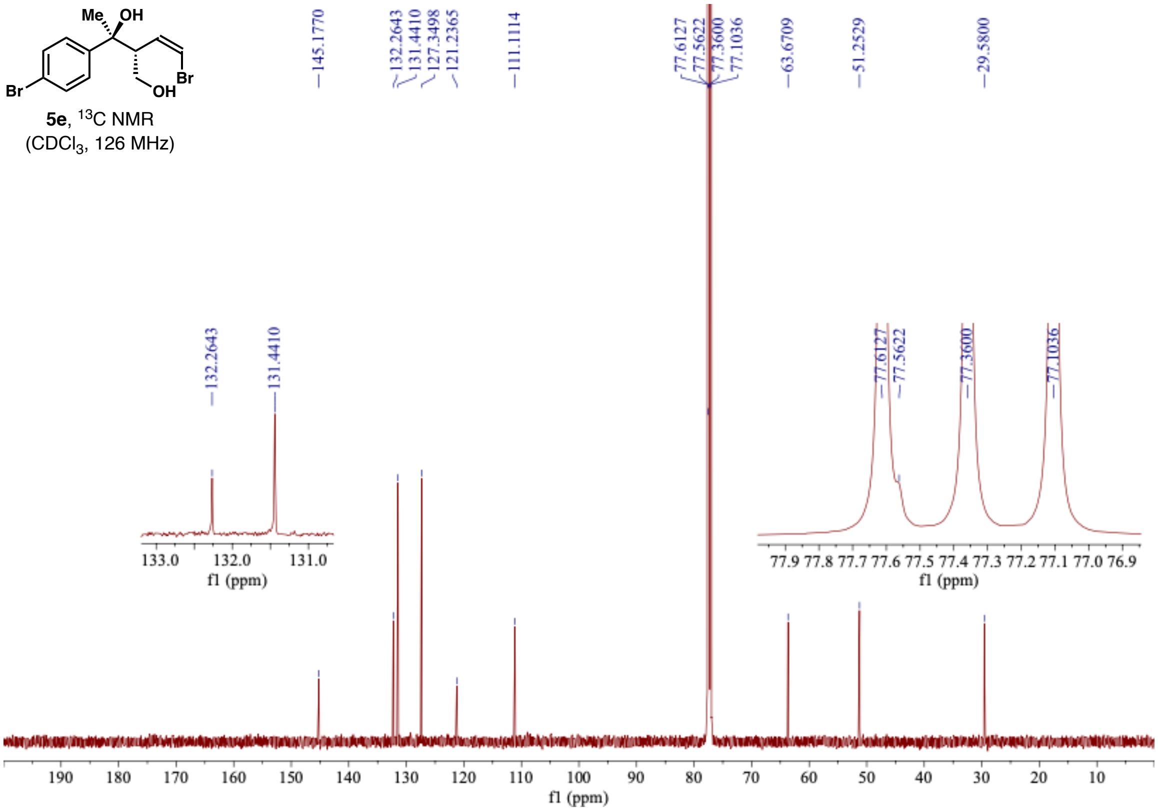

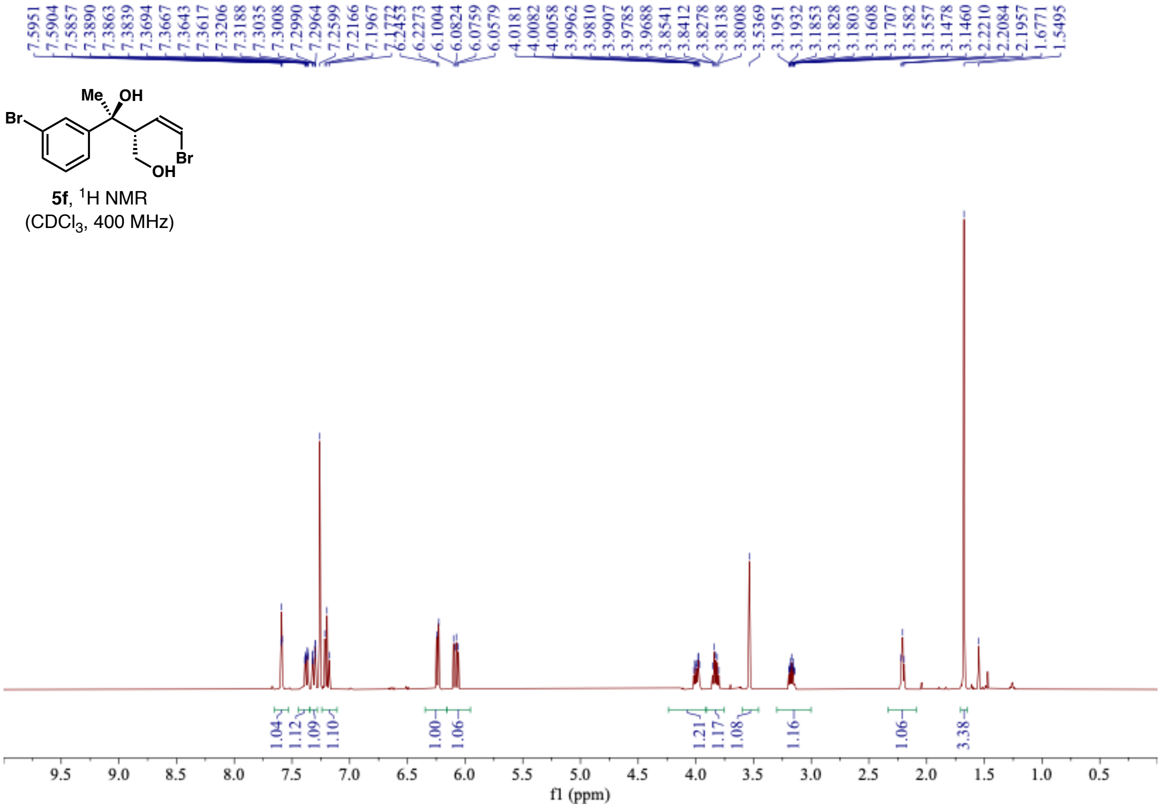

5f, ^{1}H NMR
($CDCl_3$, 400 MHz)

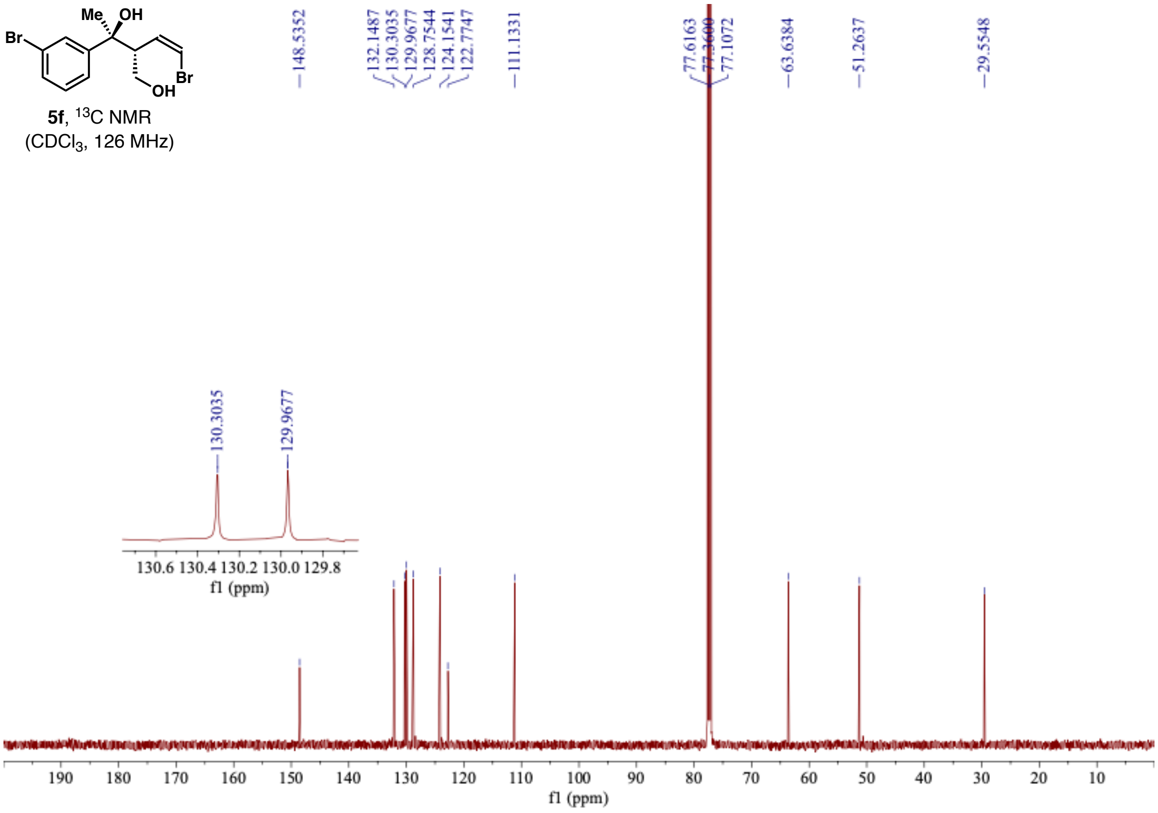

5f, ^{13}C NMR
($CDCl_3$, 126 MHz)

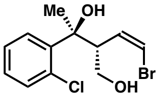

5g, ^{1}H NMR
($CDCl_3$, 400 MHz)

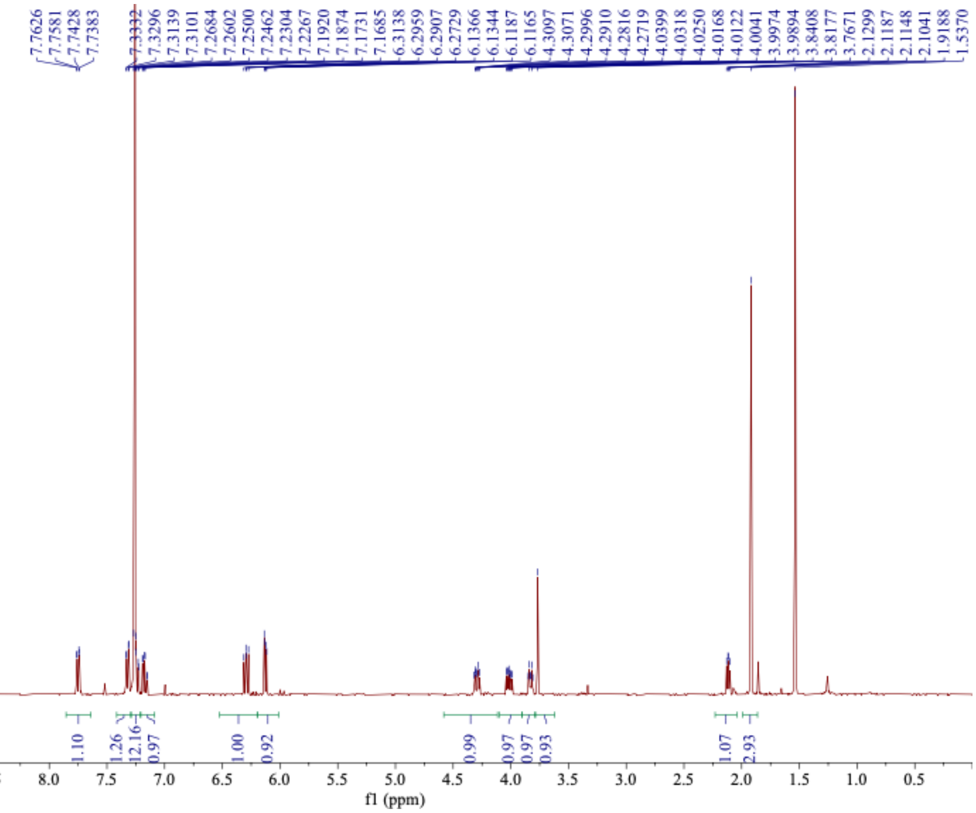

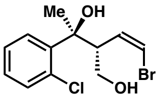

5g, ^{13}C NMR
($CDCl_3$, 126 MHz)

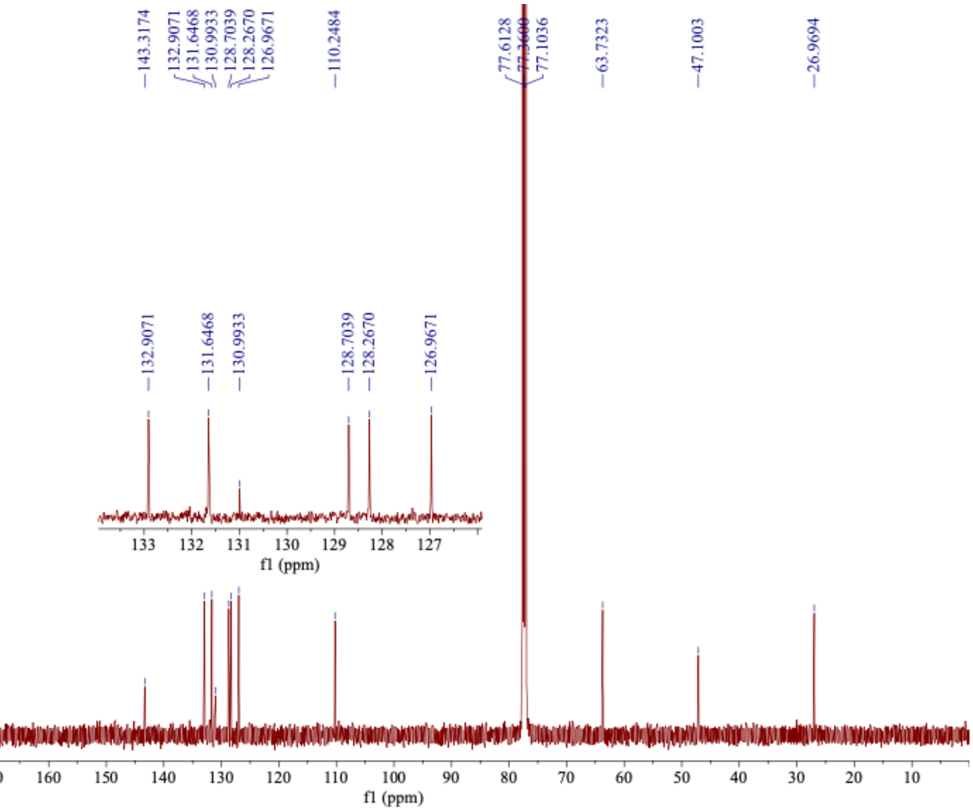

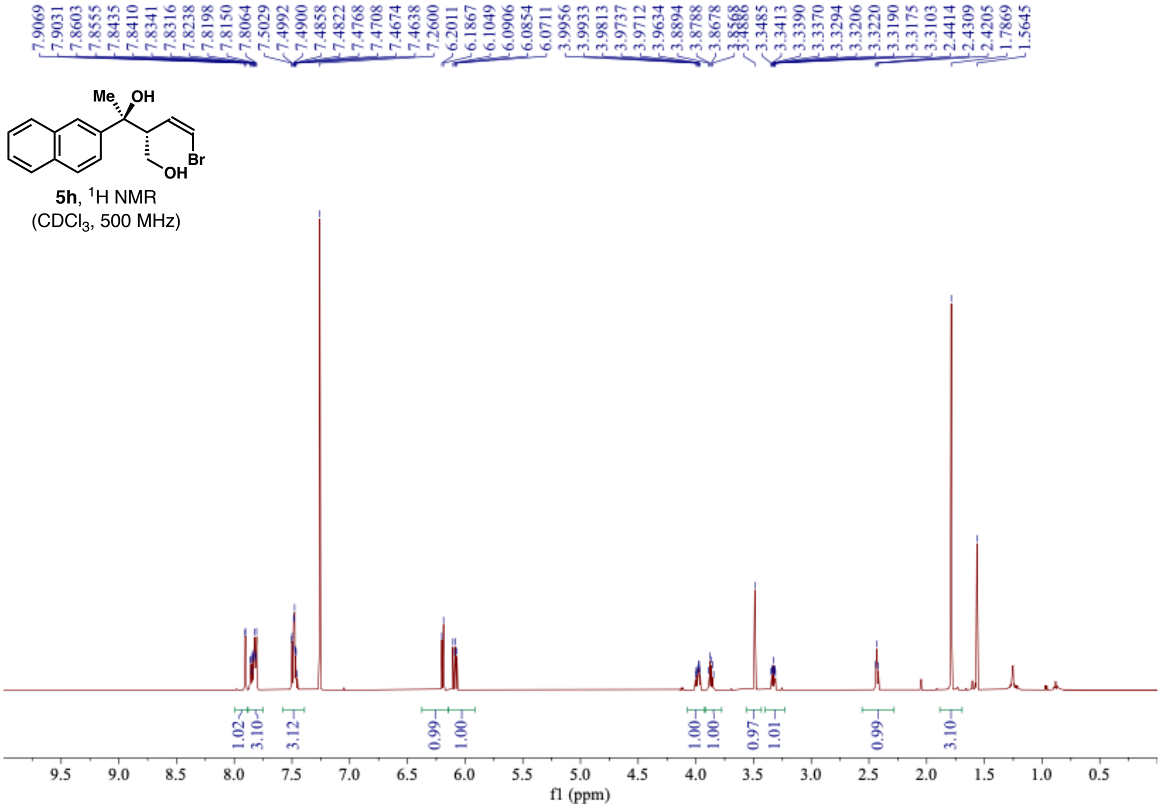

5h, ^{1}H NMR
($CDCl_3$, 500 MHz)

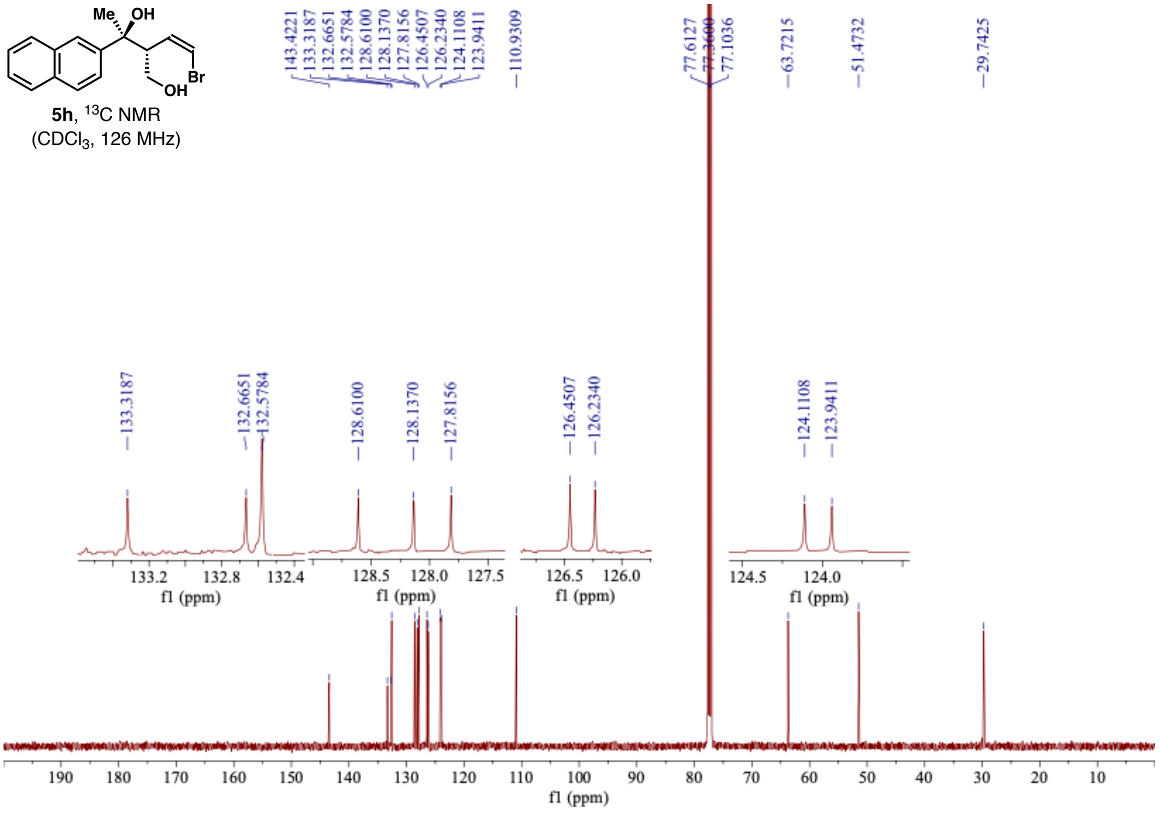

5h, ^{13}C NMR
($CDCl_3$, 126 MHz)

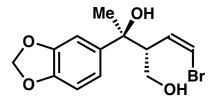

5i, ^{1}H NMR
($CDCl_3$, 500 MHz)

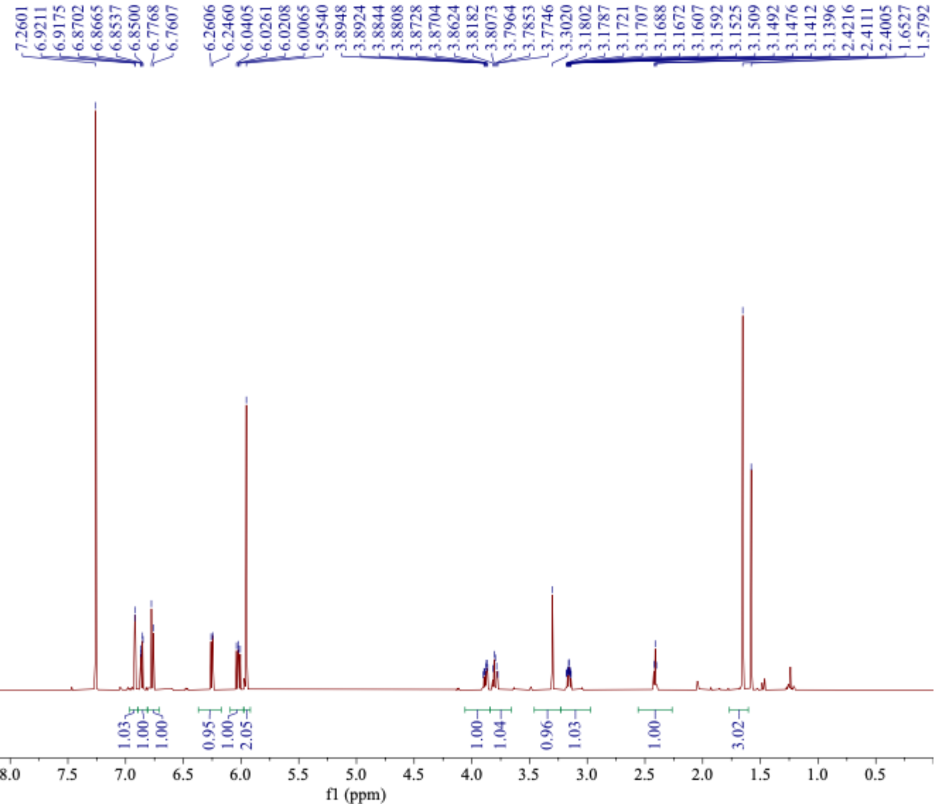

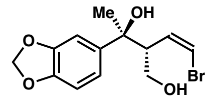

5i, ^{13}C NMR
($CDCl_3$, 126 MHz)

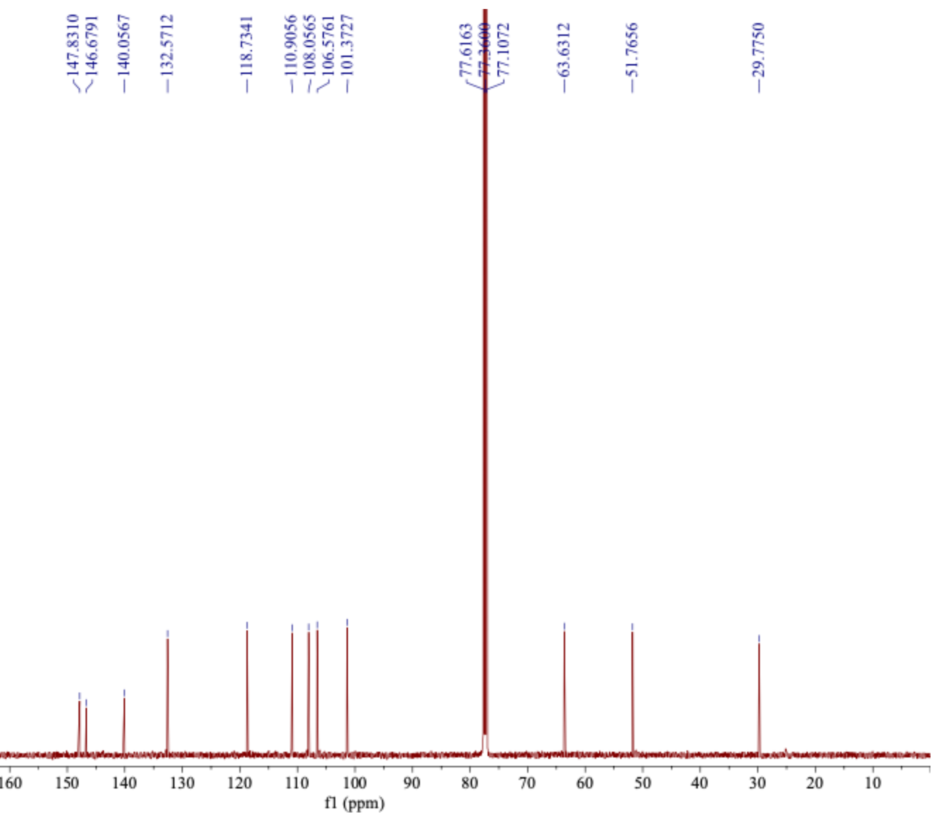

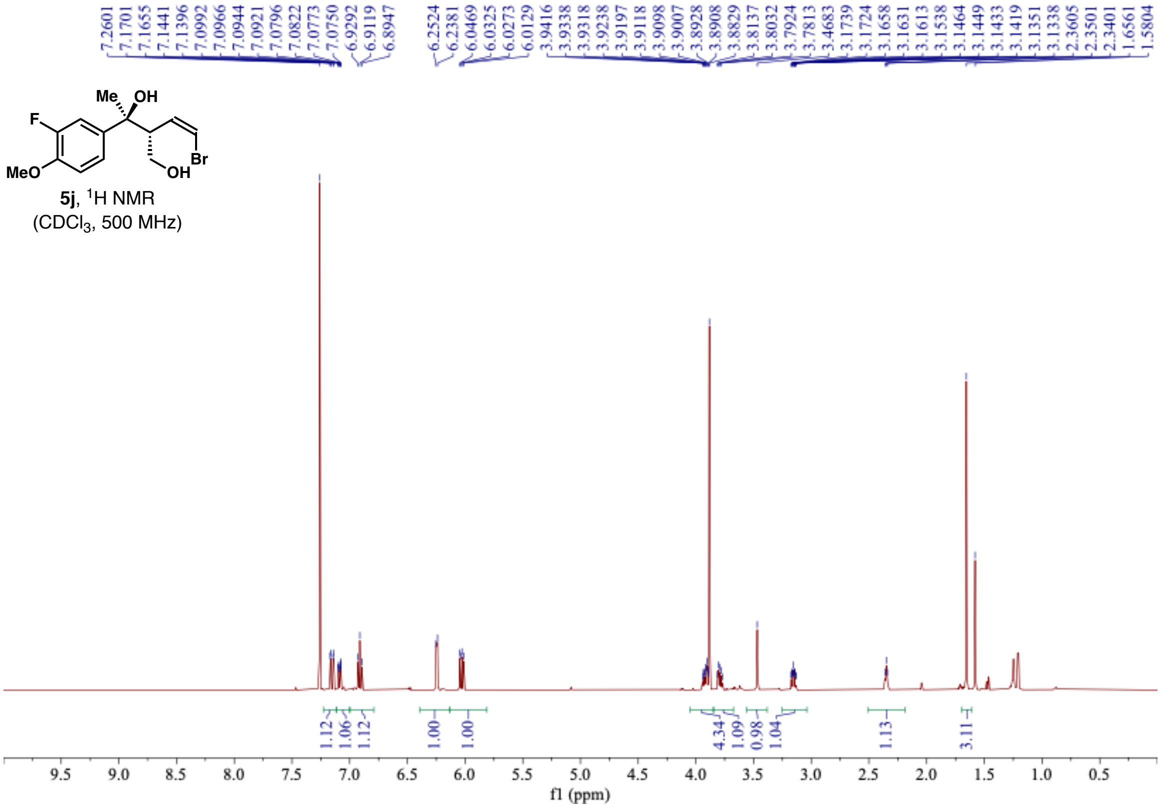

5j, ^{1}H NMR
($CDCl_3$, 500 MHz)

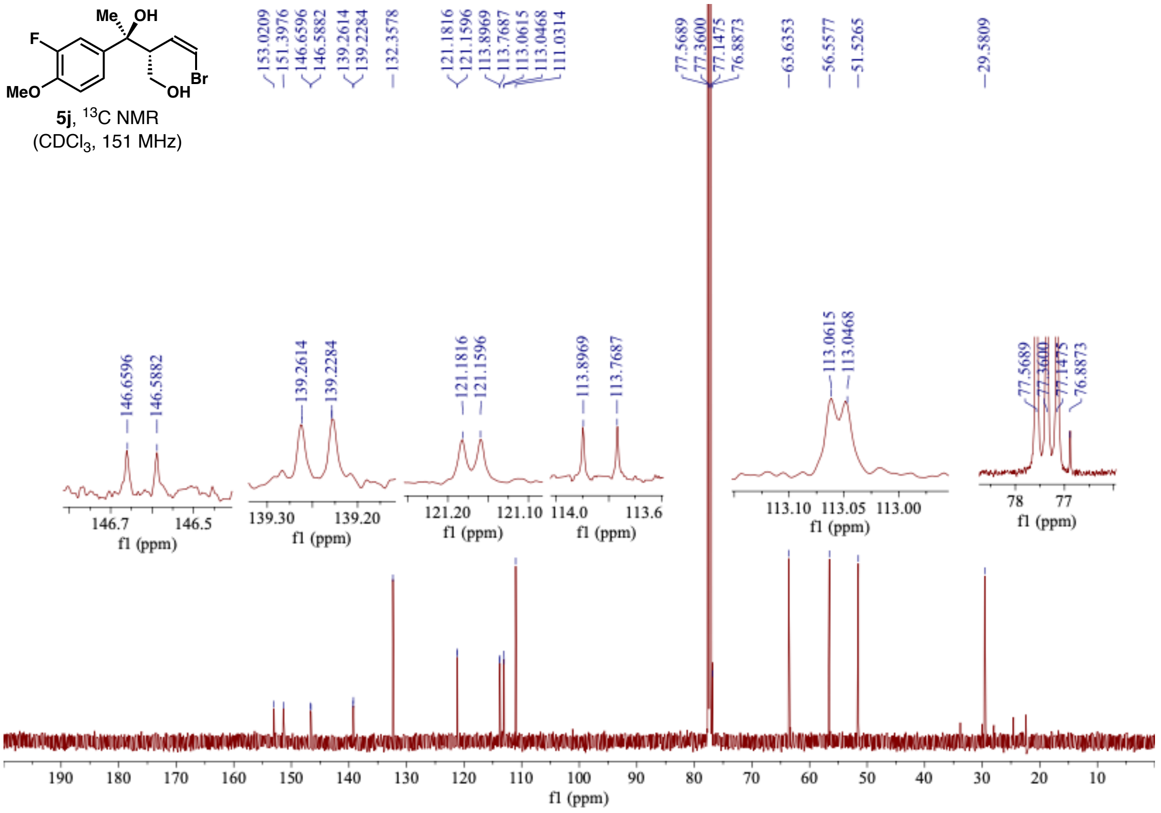

5j, ^{13}C NMR
($CDCl_3$, 151 MHz)

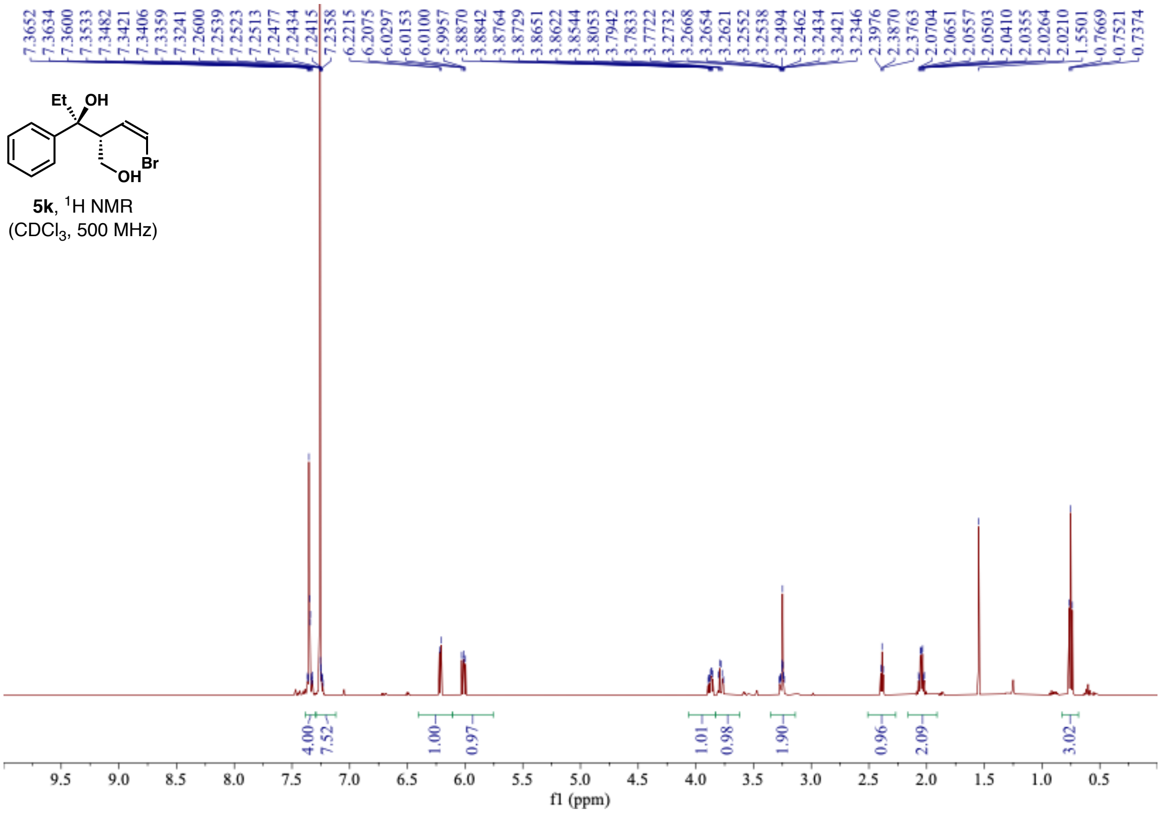

5k, ^{1}H NMR
($CDCl_3$, 500 MHz)

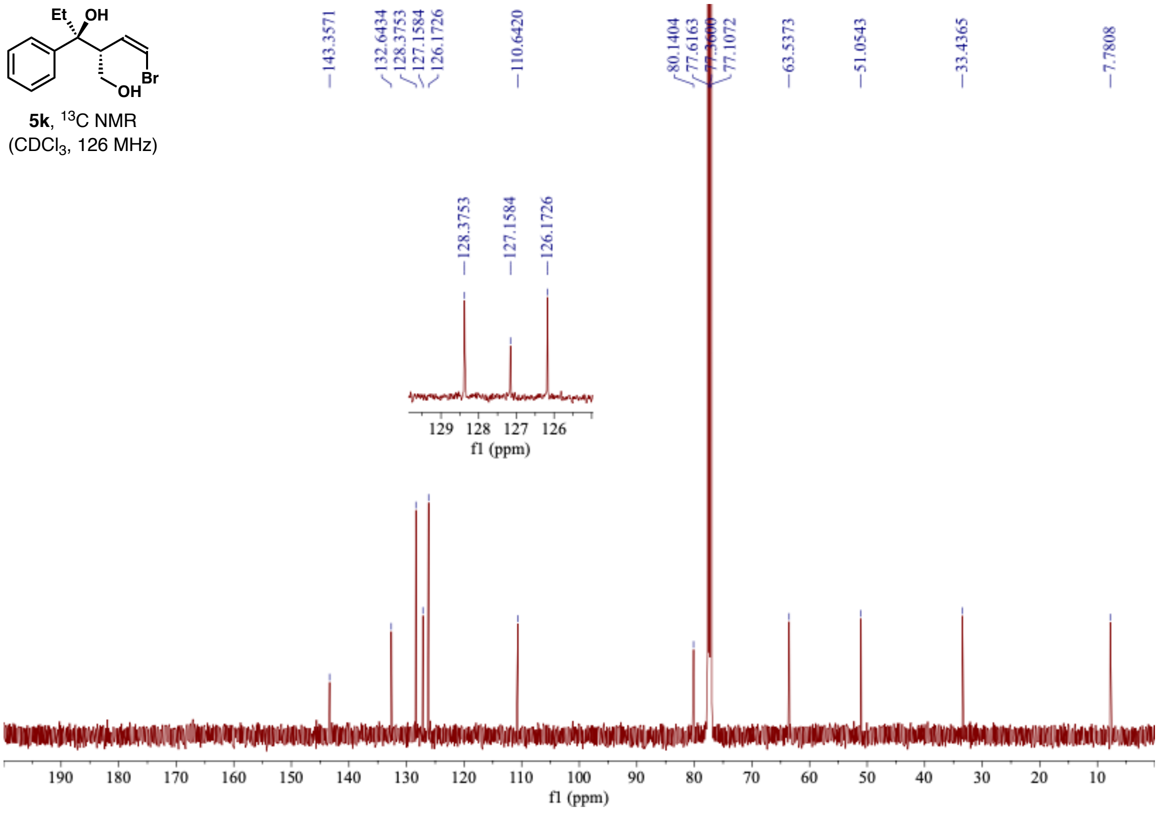

5k, ^{13}C NMR
($CDCl_3$, 126 MHz)

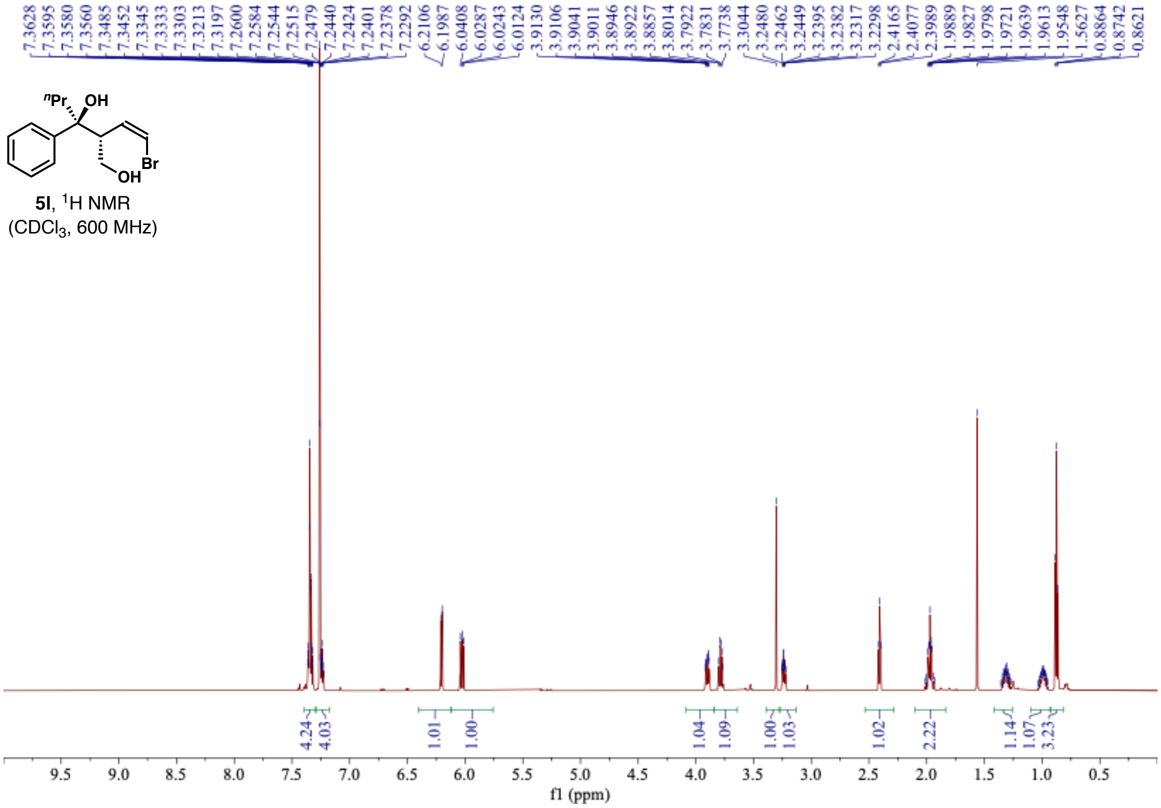

5l, ¹H NMR (CDCl$_3$, 600 MHz)

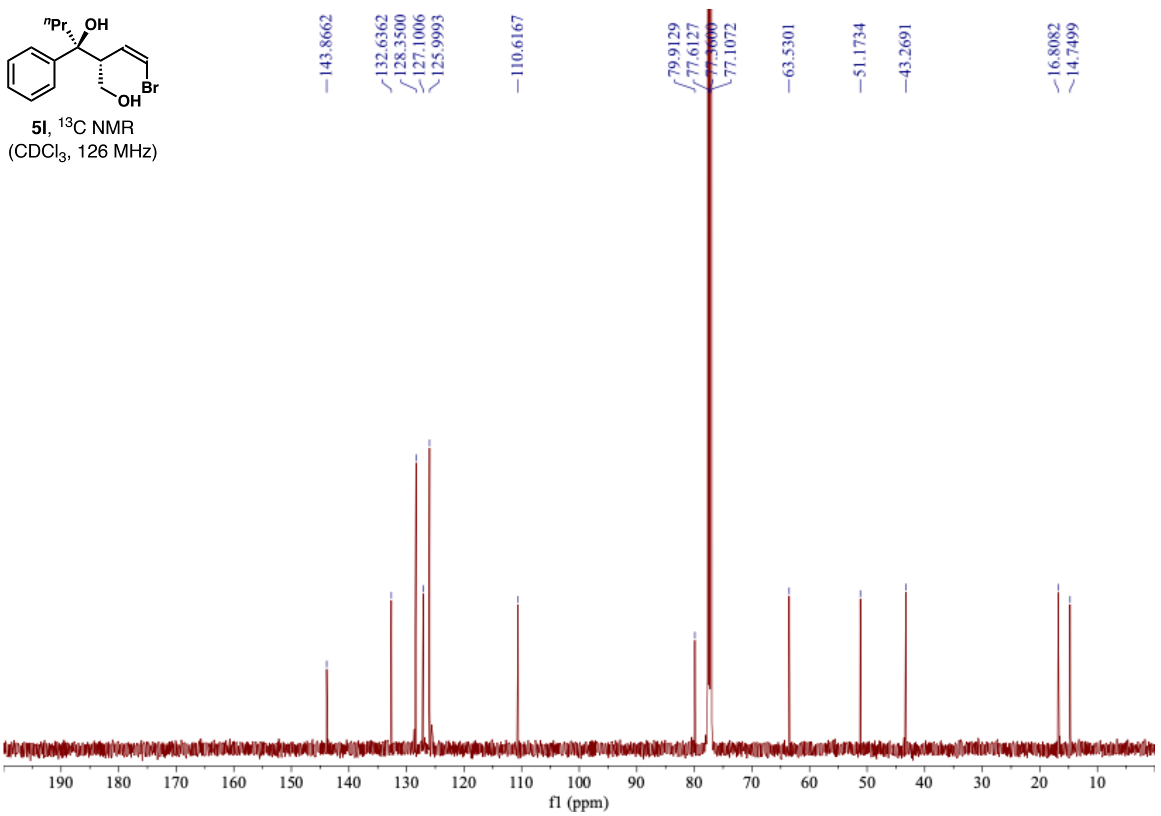

5l, ¹³C NMR (CDCl$_3$, 126 MHz)

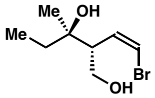

5m, ^{1}H NMR
($CDCl_3$, 600 MHz)

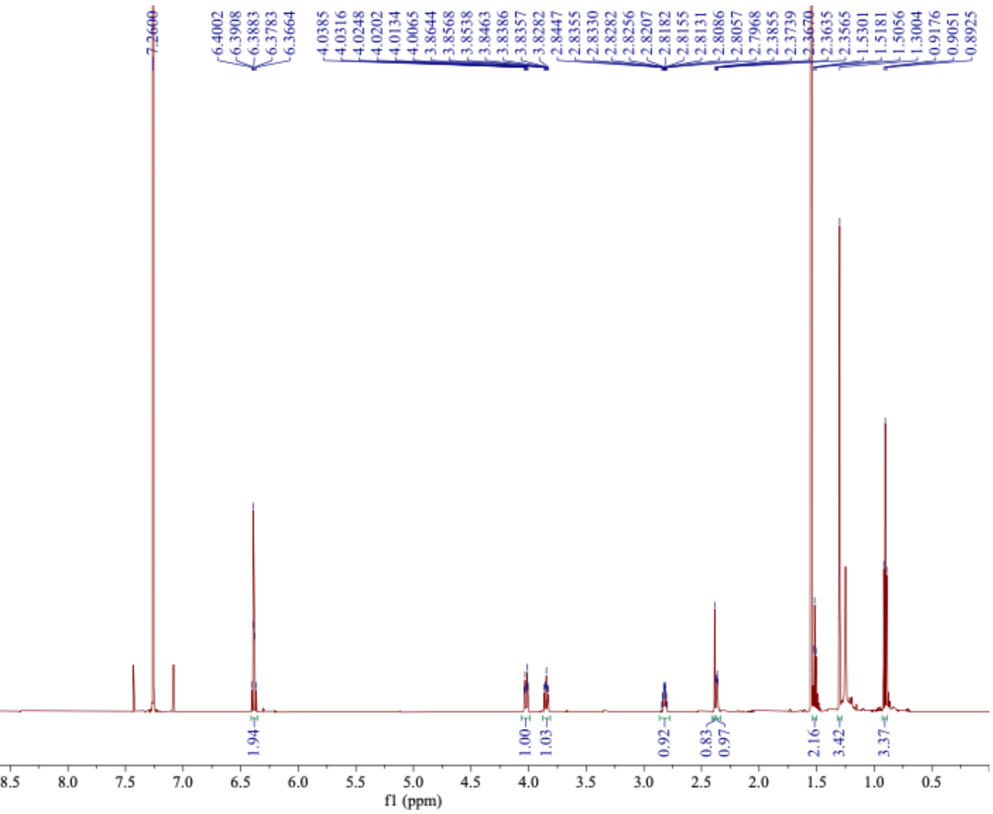

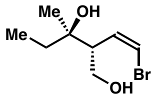

5m, ^{13}C NMR
($CDCl_3$, 126 MHz)

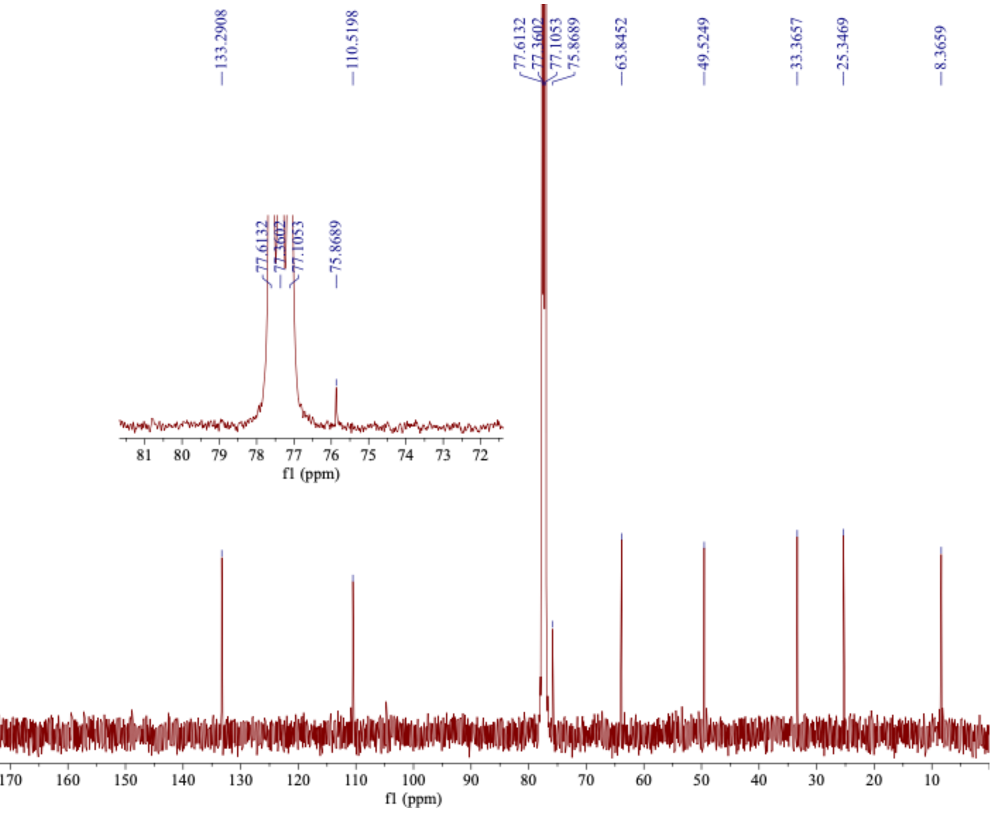

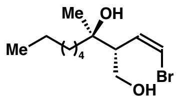

5n, ^{1}H NMR
($CDCl_3$, 500 MHz)

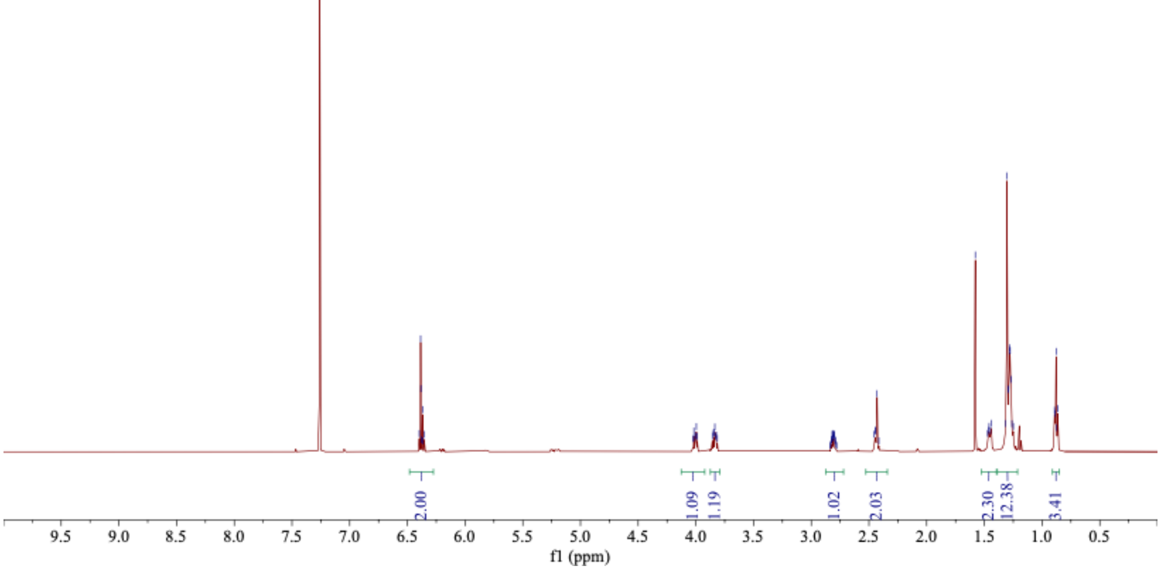

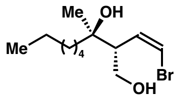

5n, ^{13}C NMR
($CDCl_3$, 151 MHz)

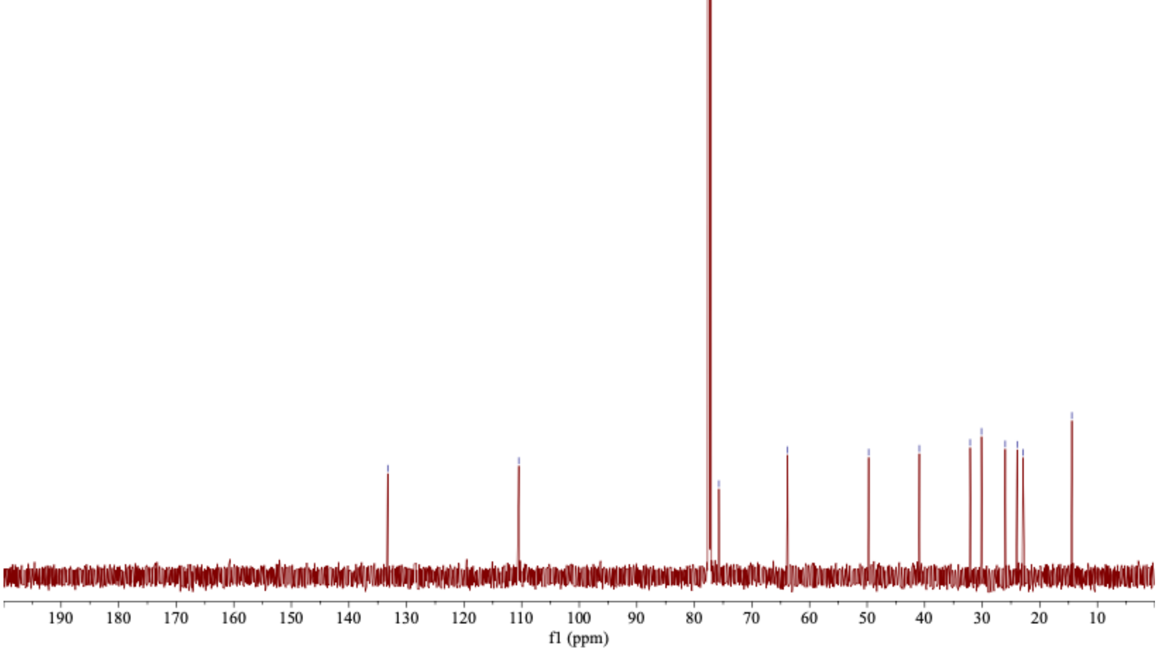

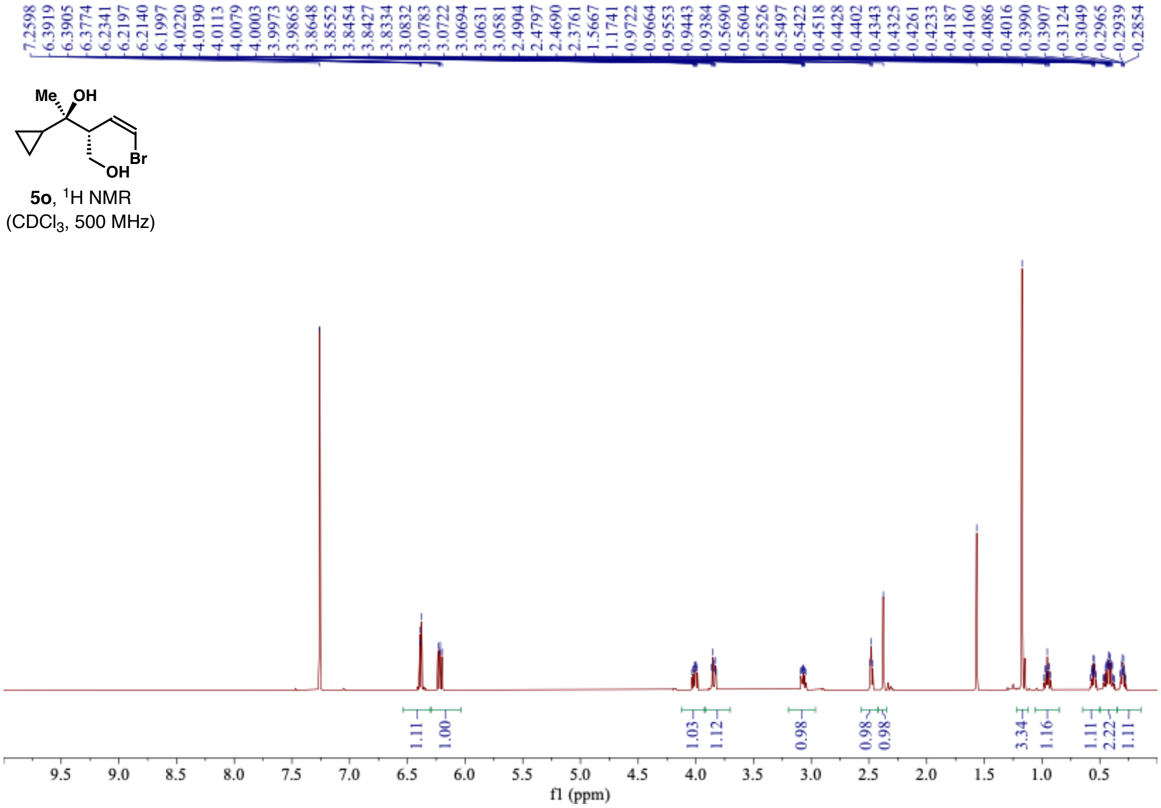

5o, ^{1}H NMR
($CDCl_3$, 500 MHz)

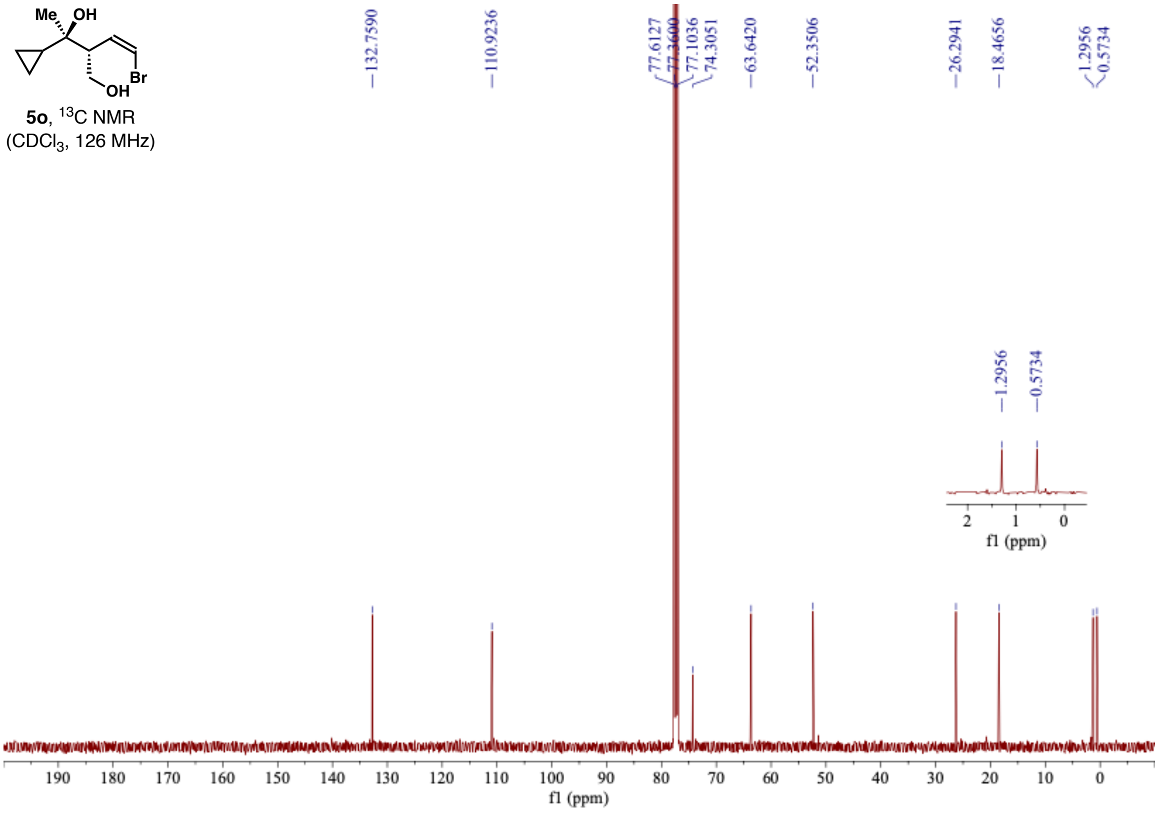

5o, ^{13}C NMR
($CDCl_3$, 126 MHz)

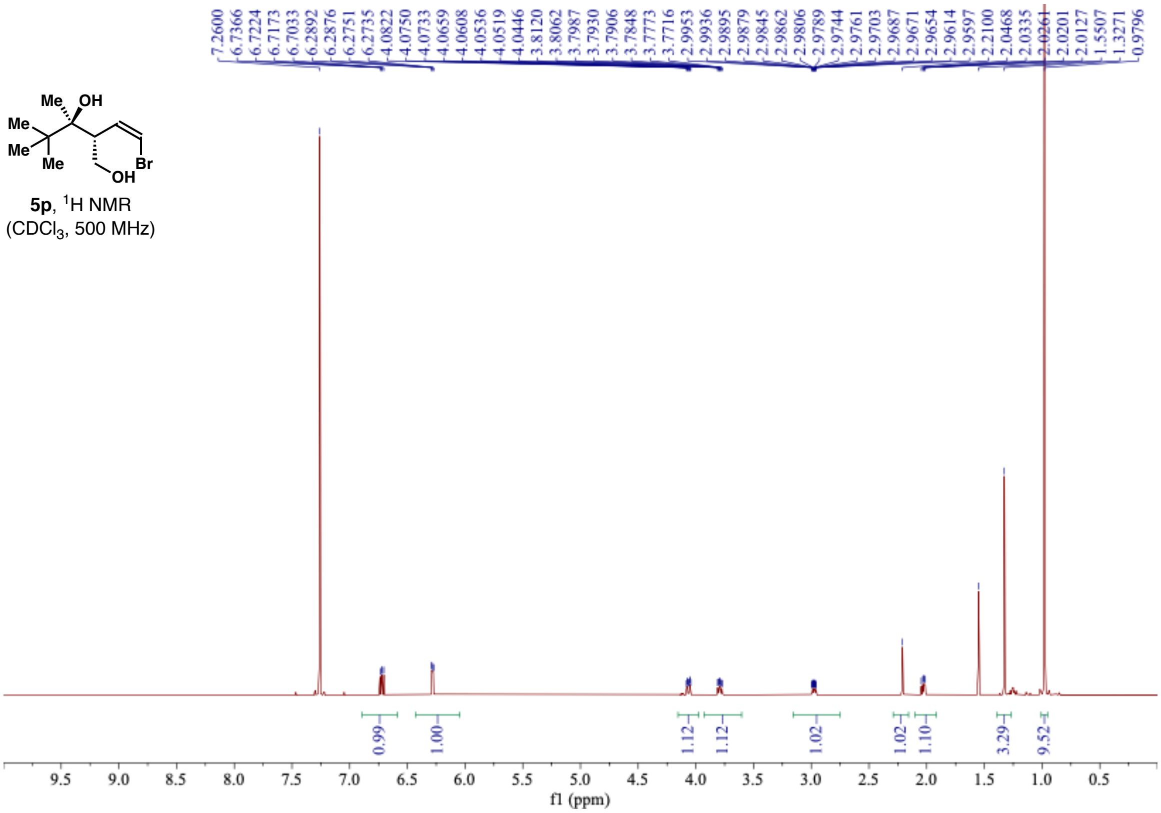

5p, ^{1}H NMR
($CDCl_3$, 500 MHz)

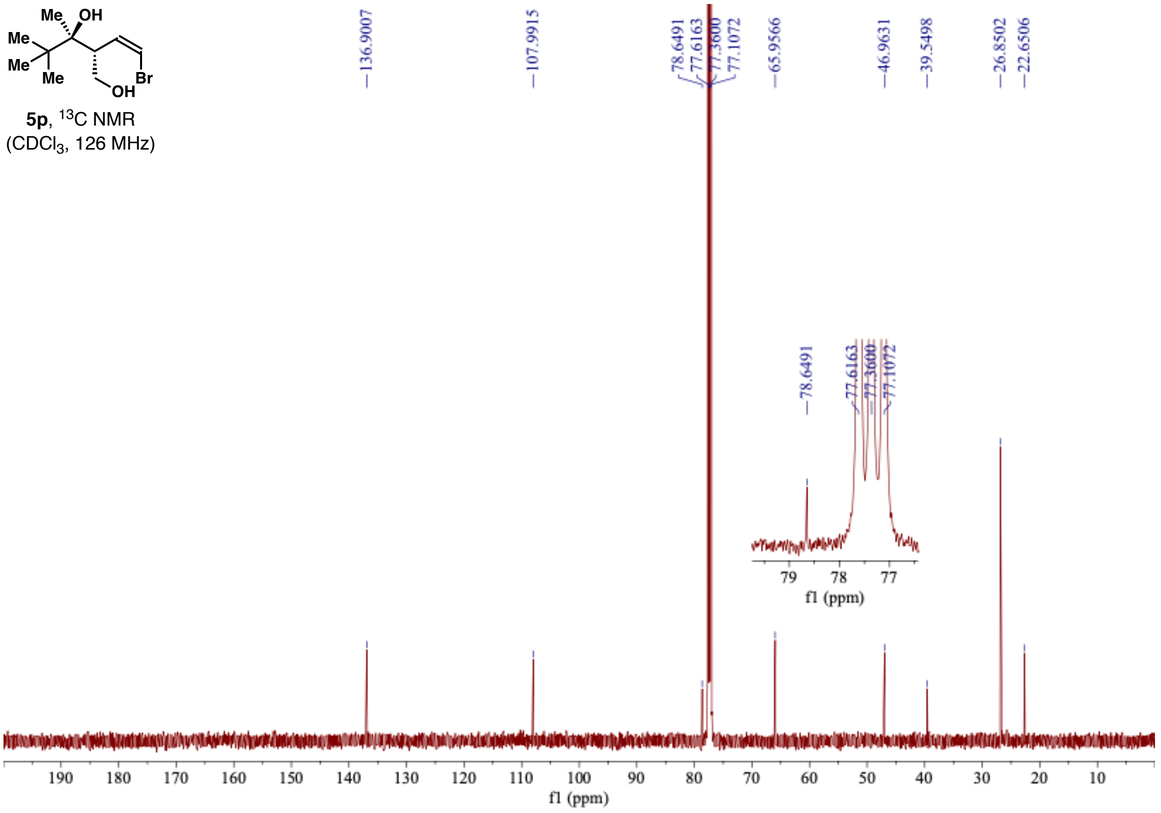

5p, ^{13}C NMR
($CDCl_3$, 126 MHz)

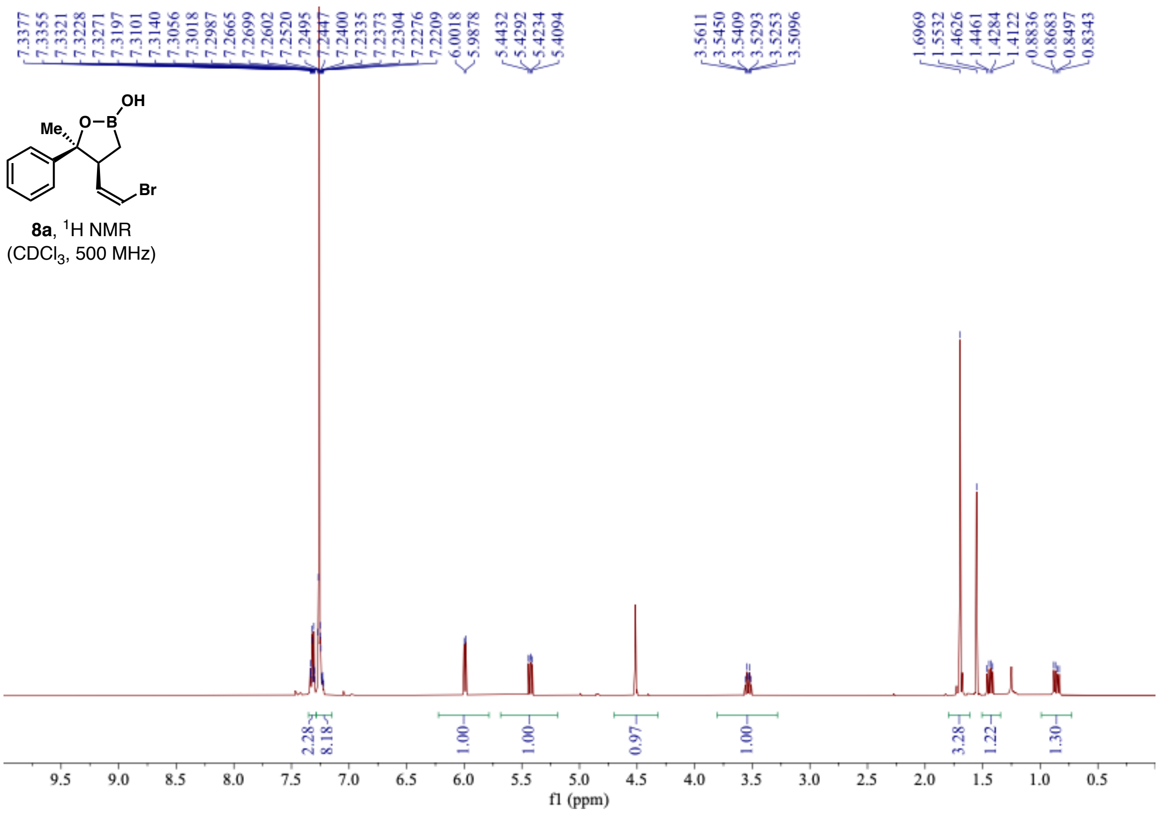
7.3377
7.3355
7.3321
7.3228
7.3271
7.3197
7.3140
7.3101
7.3056
7.3018
7.2987
7.2665
7.2699
7.2602
7.2520
7.2495
7.2447
7.2400
7.2335
7.2373
7.2304
7.2276
7.2209
6.0018
5.9878
5.4432
5.4292
5.4234
5.4094
3.5611
3.5450
3.5409
3.5293
3.5253
3.5096
1.6969
1.5532
1.4626
1.4461
1.4284
1.4122
0.8836
0.8683
0.8497
0.8343
OH
Me
O
B
Br
8a, 1H NMR
(CDCl3, 500 MHz)
2.28
8.18
1.00
1.00
0.97
1.00
3.28
1.22
1.30
9.5
9.0
8.5
8.0
7.5
7.0
6.5
6.0
5.5
5.0
4.5
4.0
3.5
3.0
2.5
2.0
1.5
1.0
0.5
f1 (ppm)

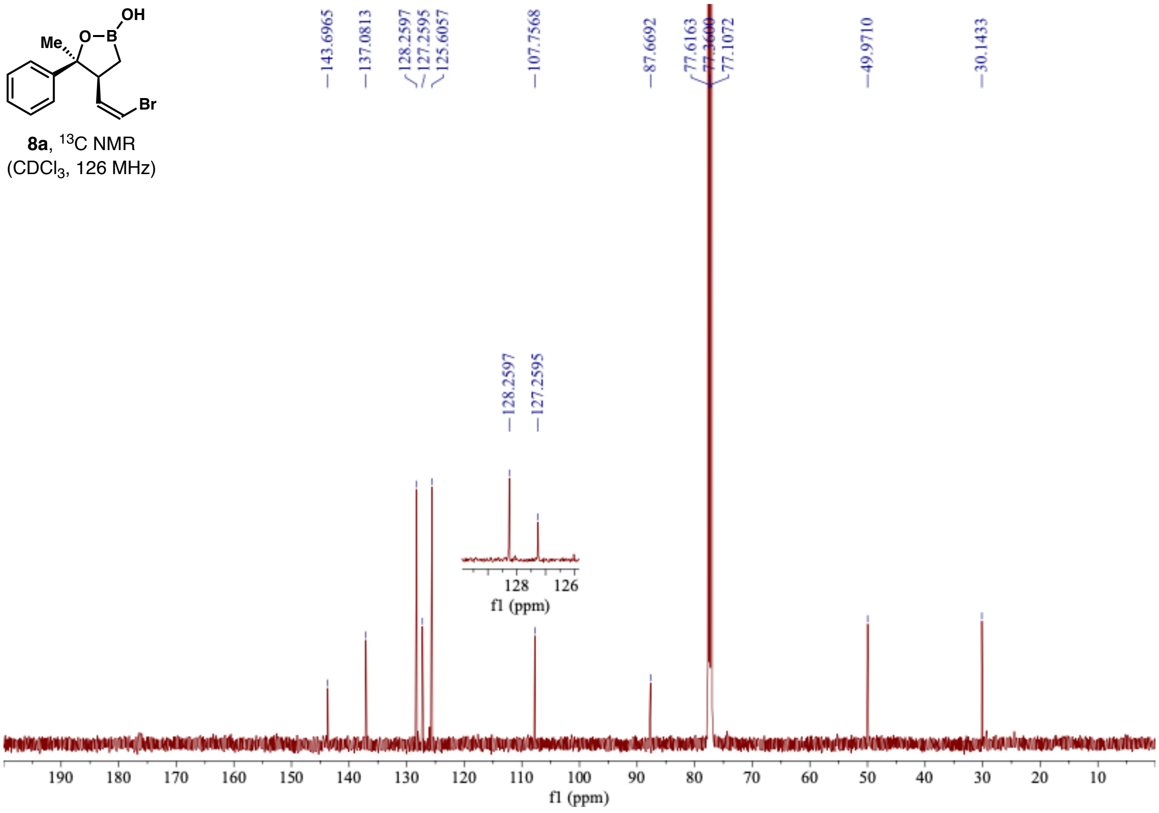
OH
Me
O
B
Br
8a, 13C NMR
(CDCl3, 126 MHz)
143.6965
137.0813
128.2597
127.2595
125.6057
107.7568
87.6692
77.6163
77.3600
77.1072
49.9710
30.1433
128.2597
127.2595
128
126
f1 (ppm)
190
180
170
160
150
140
130
120
110
100
90
80
70
60
50
40
30
20
10
f1 (ppm)

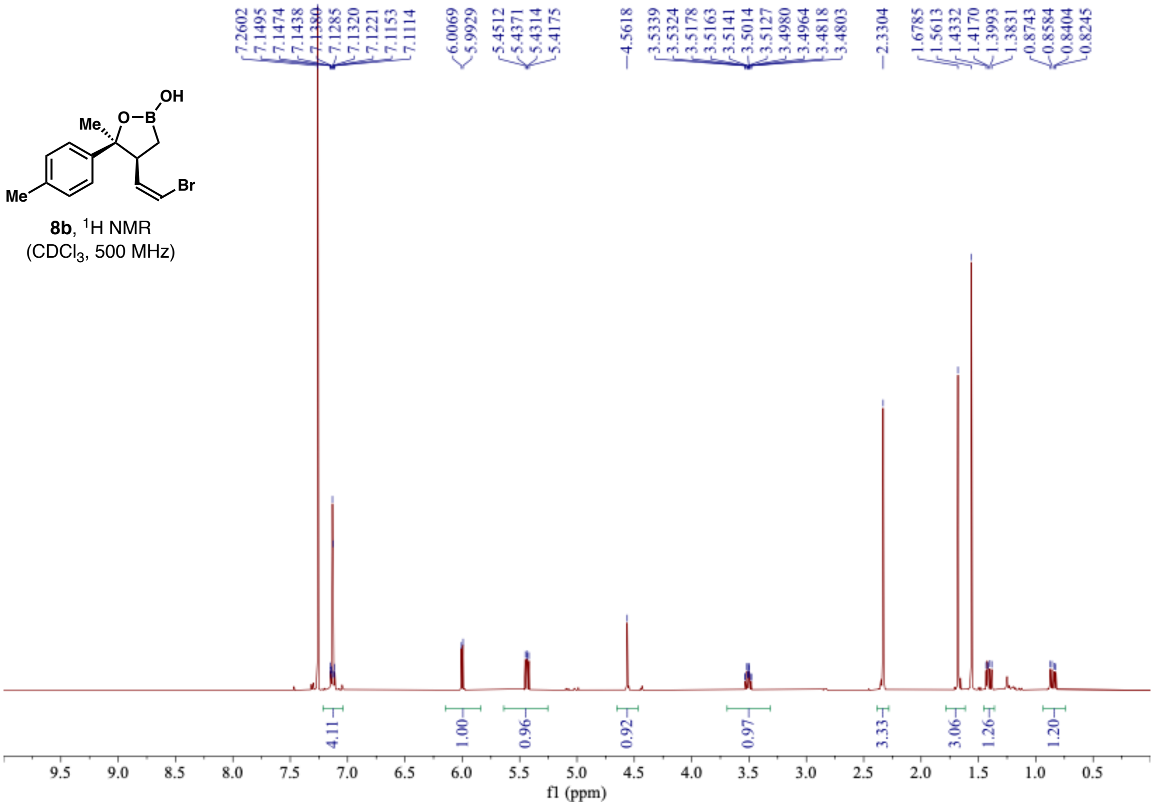

8b, ^{1}H NMR
($CDCl_3$, 500 MHz)

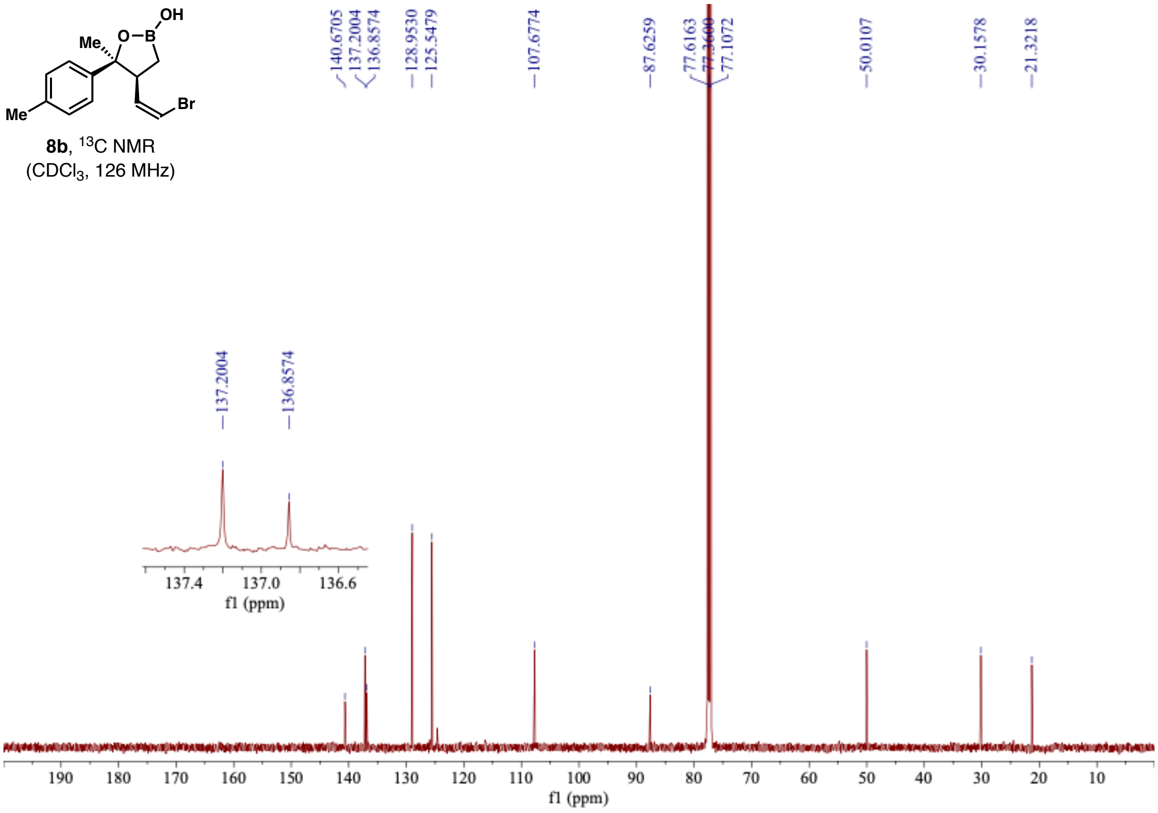

8b, ^{13}C NMR
($CDCl_3$, 126 MHz)

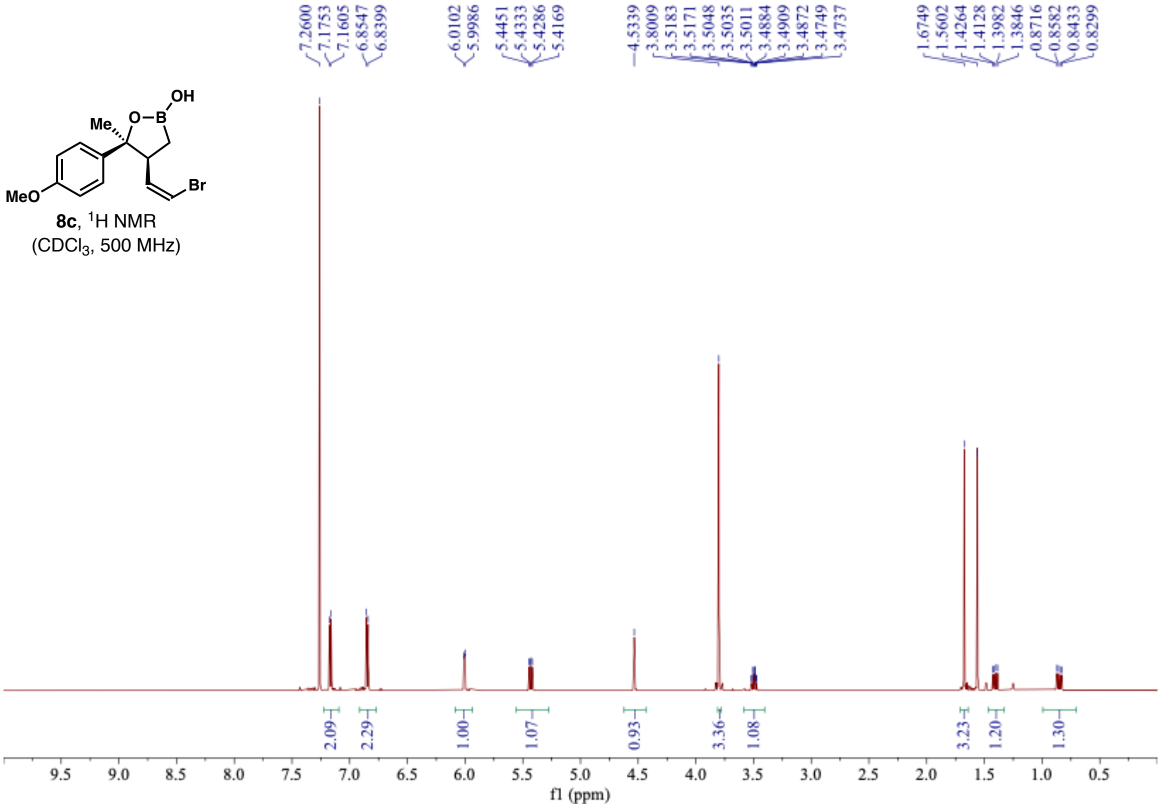

OH
Me
O
B
MeO
Br
8c, 1H NMR
(CDCl3, 500 MHz)
7.2600
7.1753
7.1605
6.8547
6.8399
6.0102
5.9986
5.4451
5.4333
5.4286
5.4169
4.5339
3.8009
3.5183
3.5171
3.5048
3.5035
3.5011
3.4884
3.4909
3.4872
3.4749
3.4737
1.6749
1.5602
1.4264
1.4128
1.3982
1.3846
0.8716
0.8582
0.8433
0.8299
2.09
2.29
1.00
1.07
0.93
3.36
1.08
3.23
1.20
1.30
9.5
9.0
8.5
8.0
7.5
7.0
6.5
6.0
5.5
5.0
4.5
4.0
3.5
3.0
2.5
2.0
1.5
1.0
0.5
f1 (ppm)

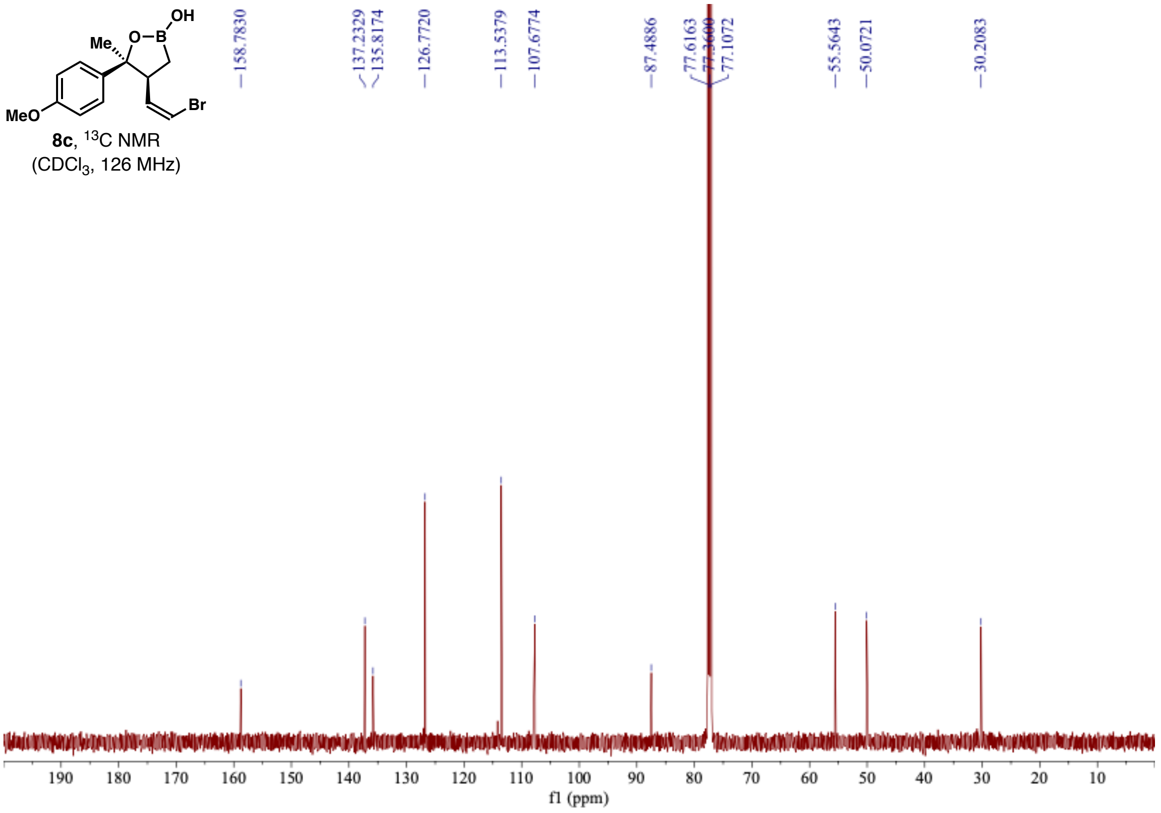

OH
Me
O
B
MeO
Br
8c, 13C NMR
(CDCl3, 126 MHz)
158.7830
137.2329
135.8174
126.7720
113.5379
107.6774
87.4886
77.6163
77.3600
77.1072
55.5643
50.0721
30.2083
190
180
170
160
150
140
130
120
110
100
90
80
70
60
50
40
30
20
10
f1 (ppm)

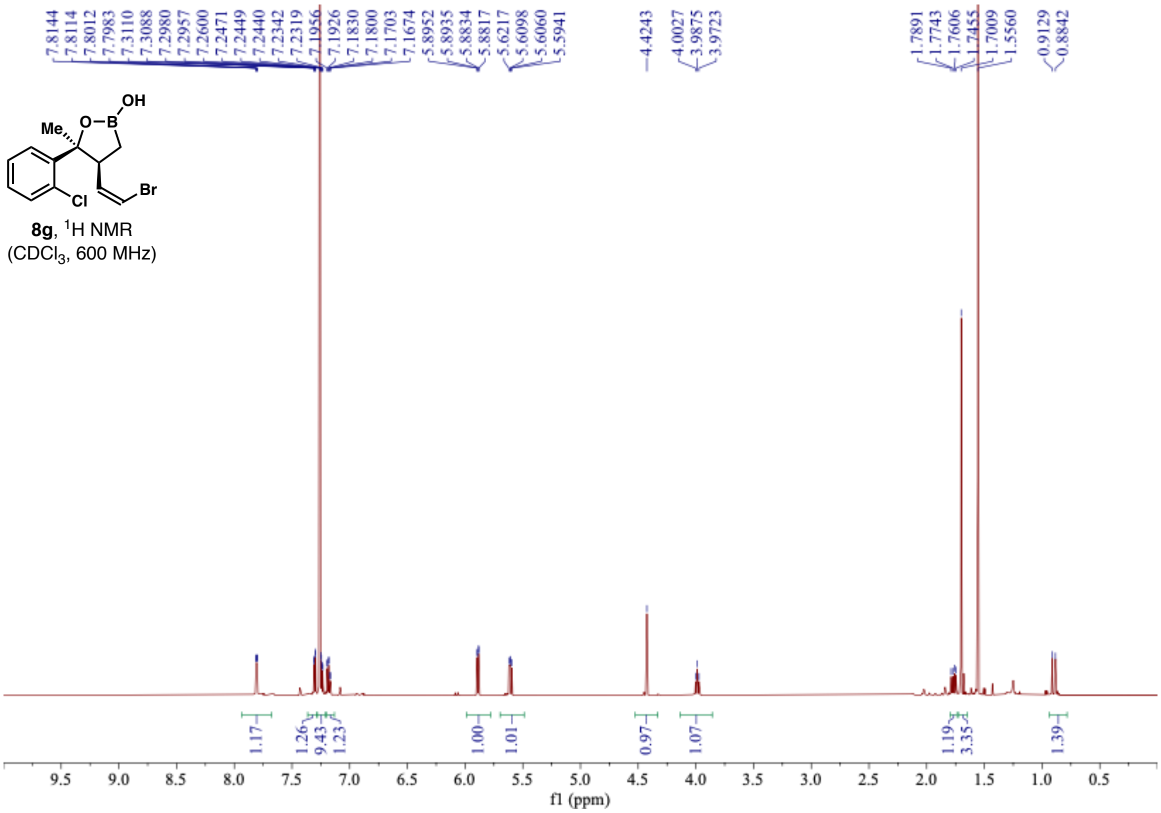

8g, ^{1}H NMR
($CDCl_3$, 600 MHz)

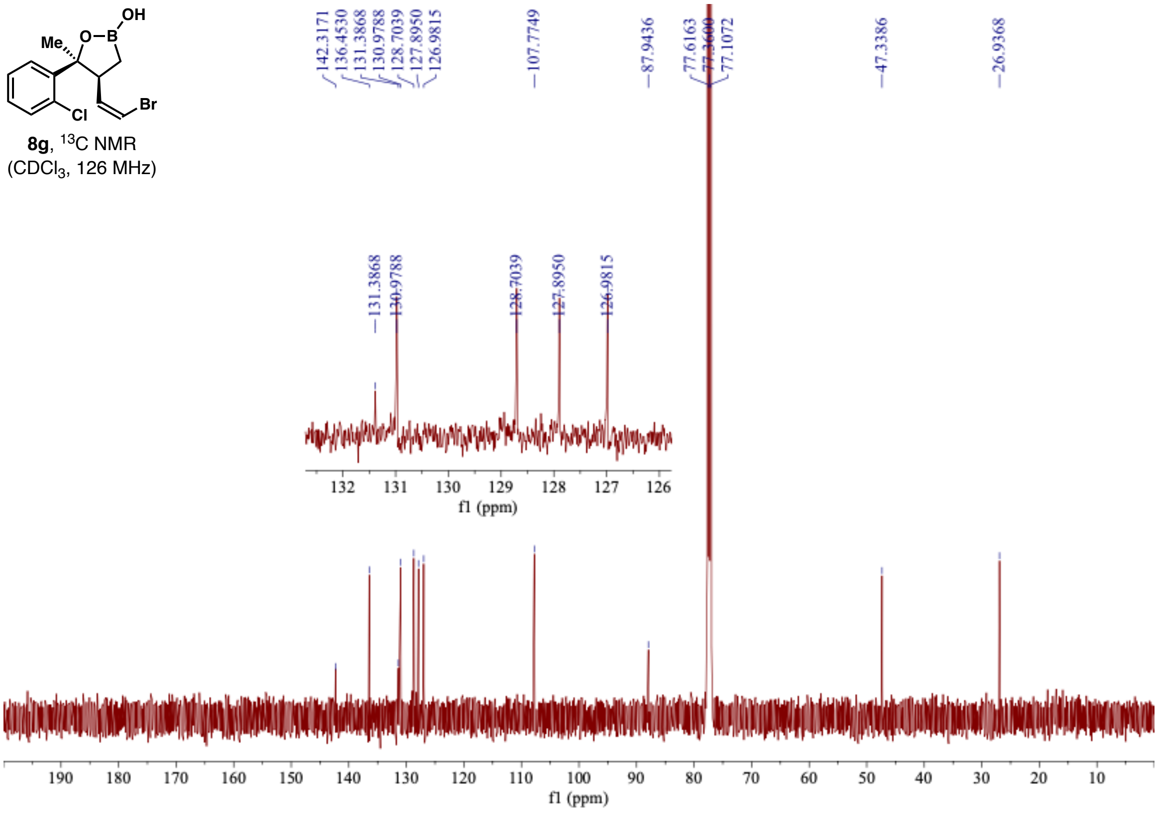

8g, ^{13}C NMR
($CDCl_3$, 126 MHz)

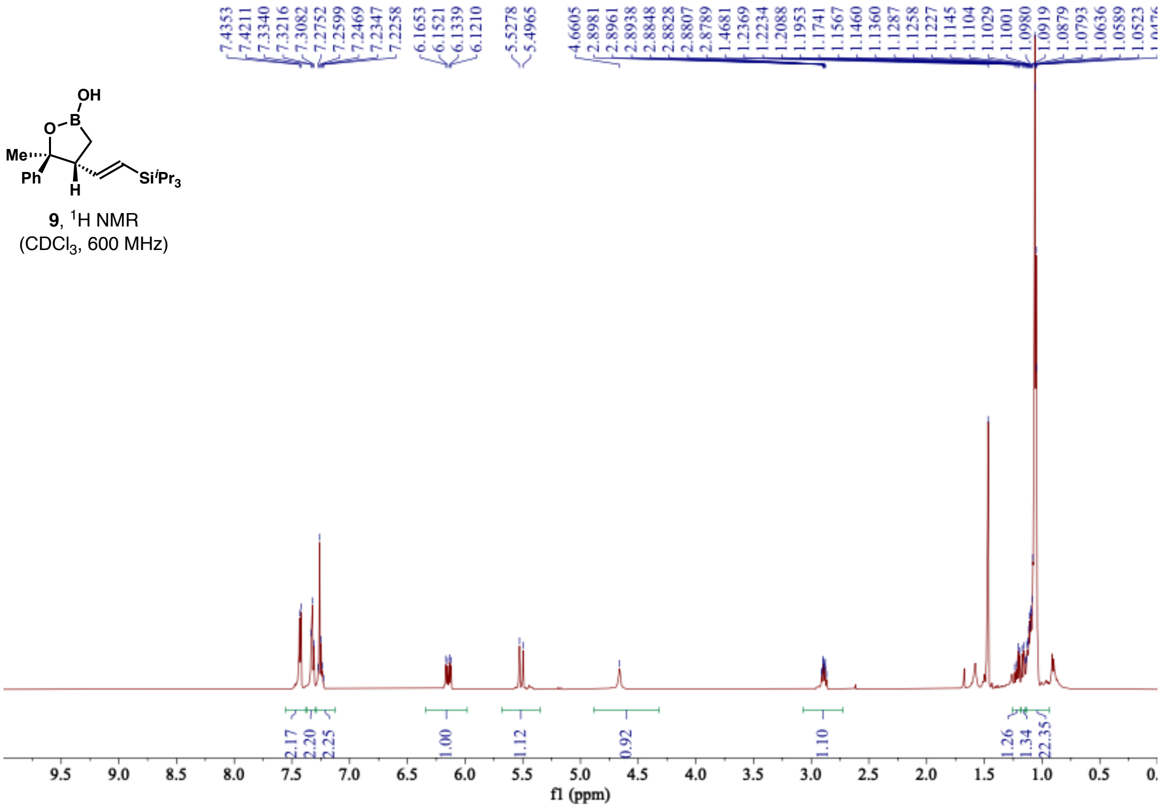

9, ^{1}H NMR
($CDCl_3$, 600 MHz)

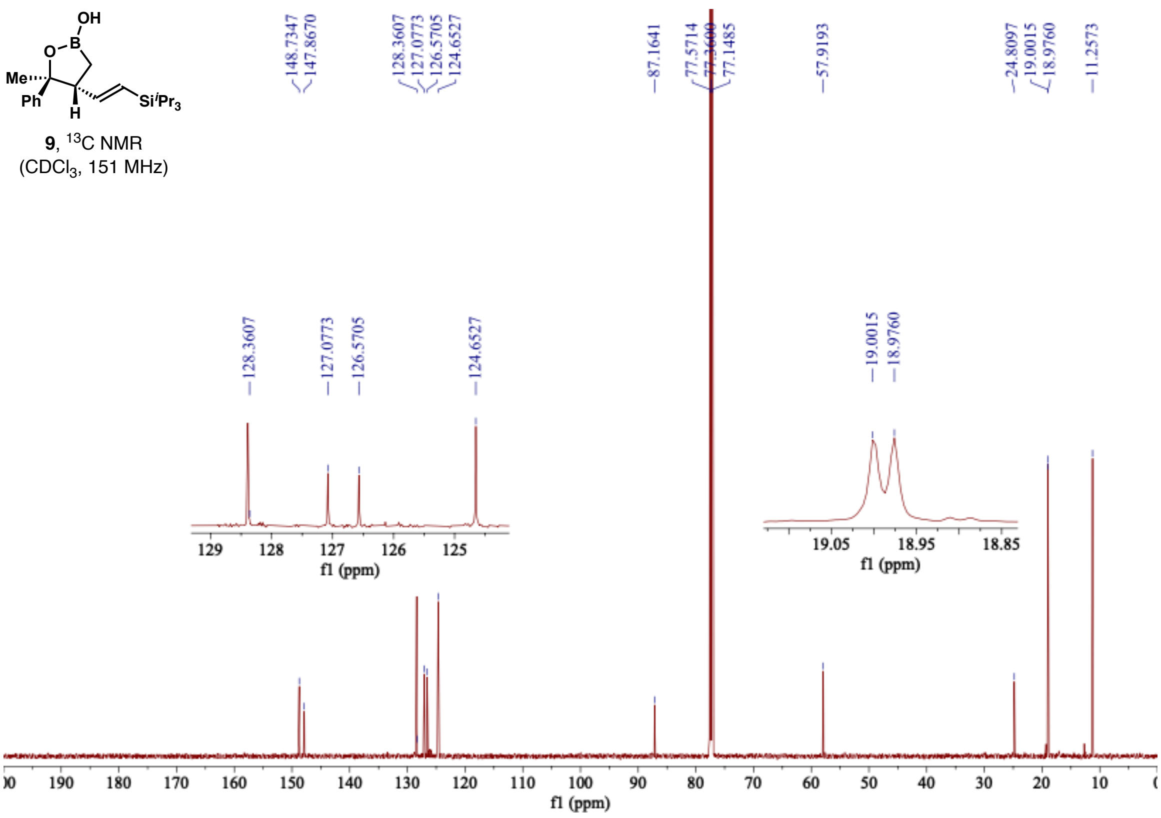

9, ^{13}C NMR
($CDCl_3$, 151 MHz)

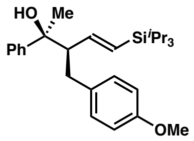

10, ^{1}H NMR
($CDCl_3$, 500 MHz)

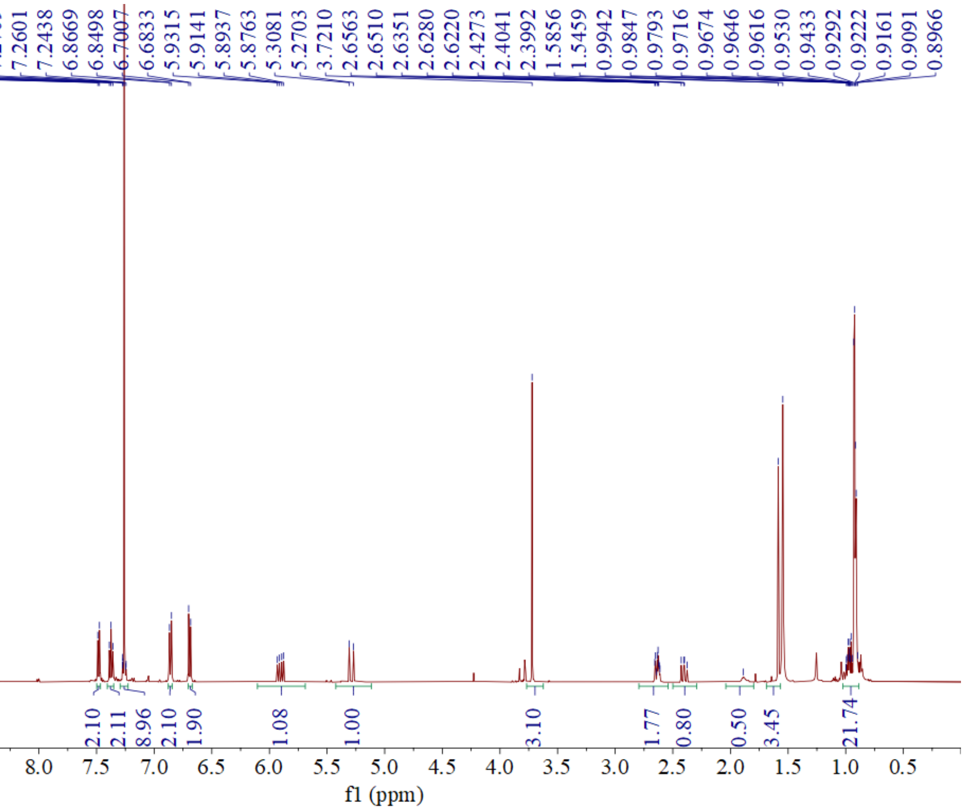

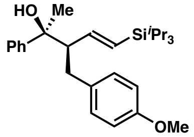

10, ^{13}C NMR
($CDCl_3$, 126 MHz)

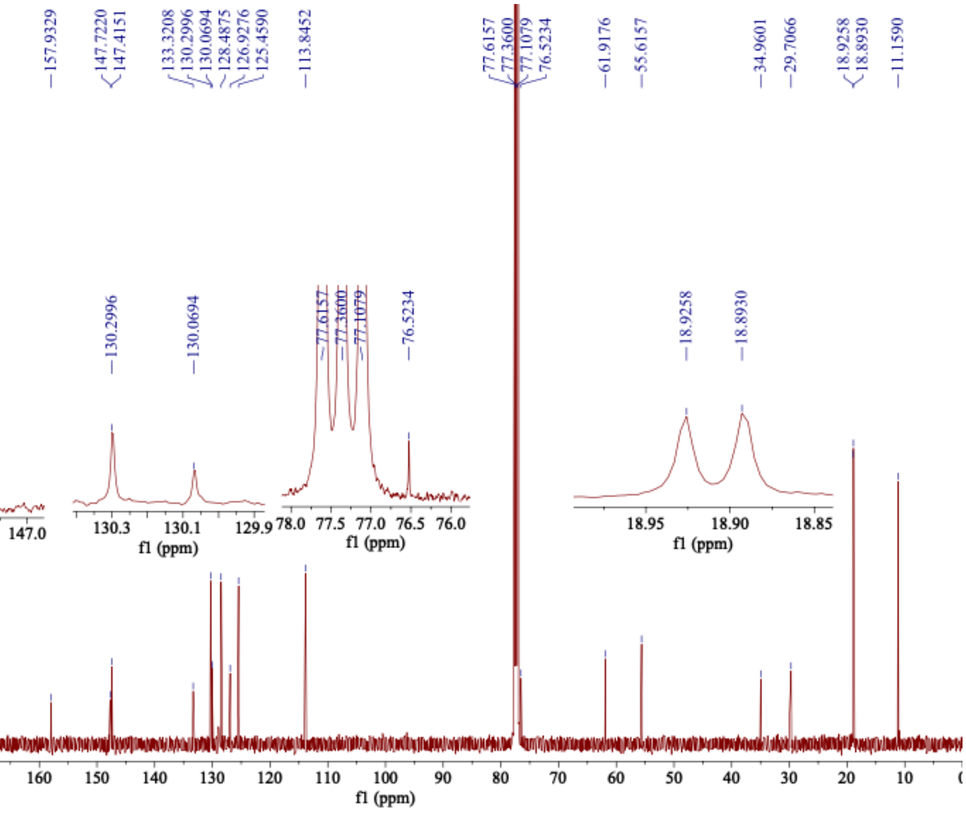

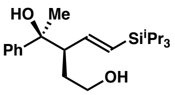

11, ^{1}H NMR
(C_6D_6, 600 MHz)

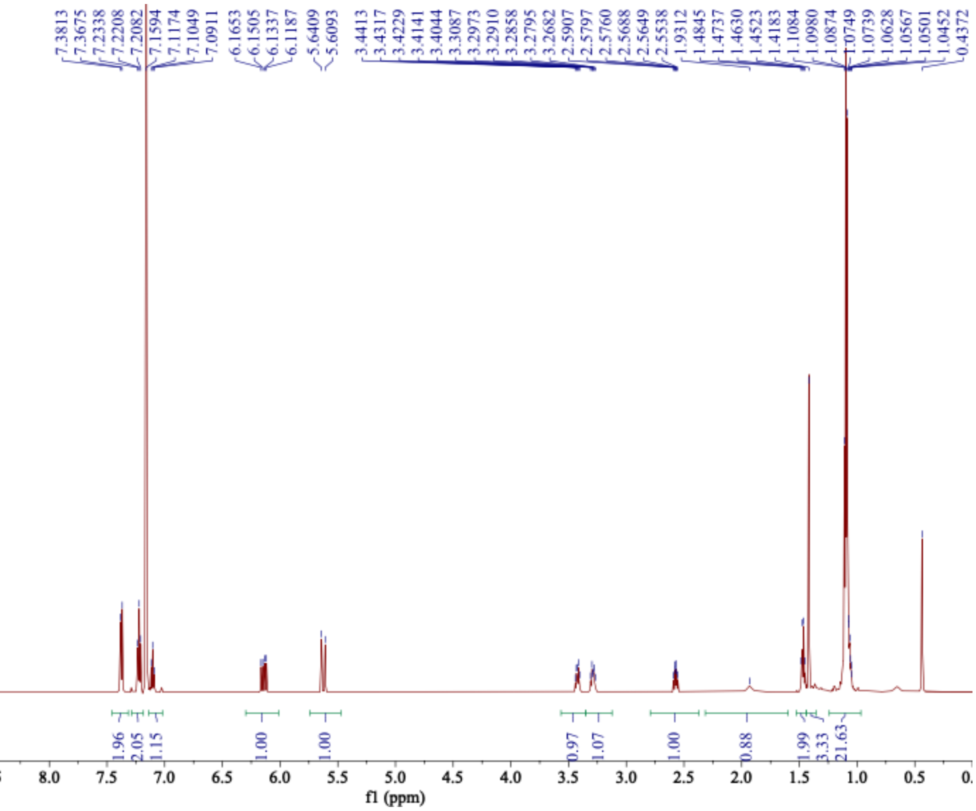

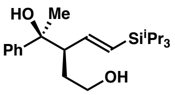

11, ^{13}C NMR
($CDCl_3$, 151 MHz)

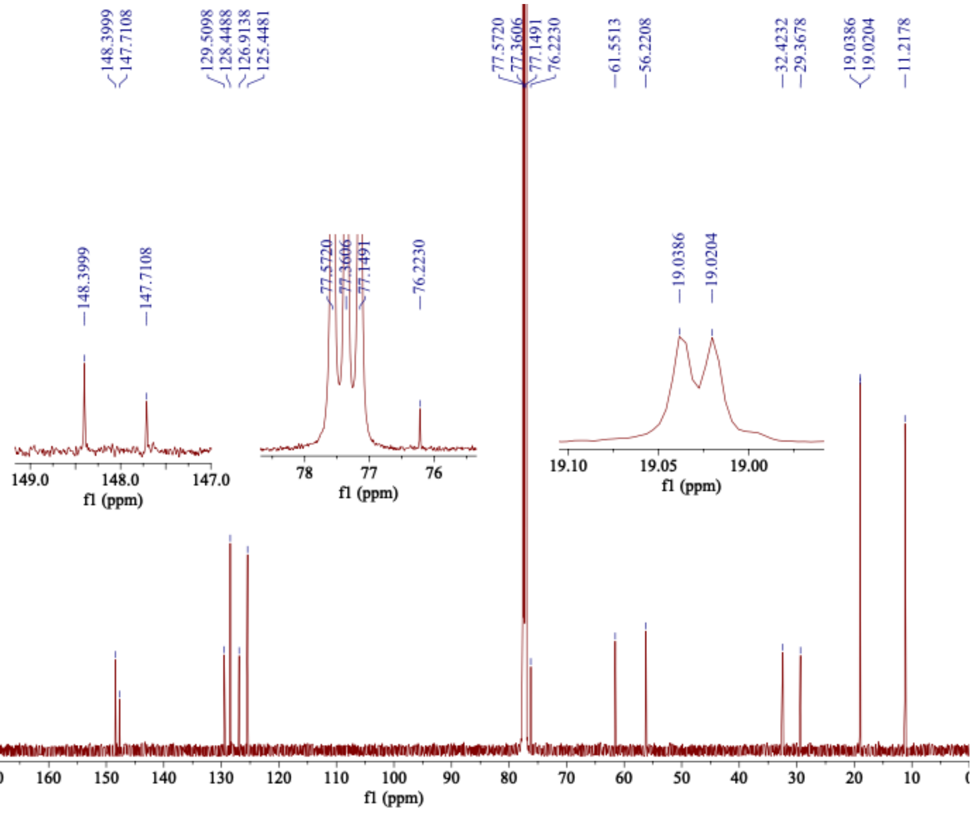

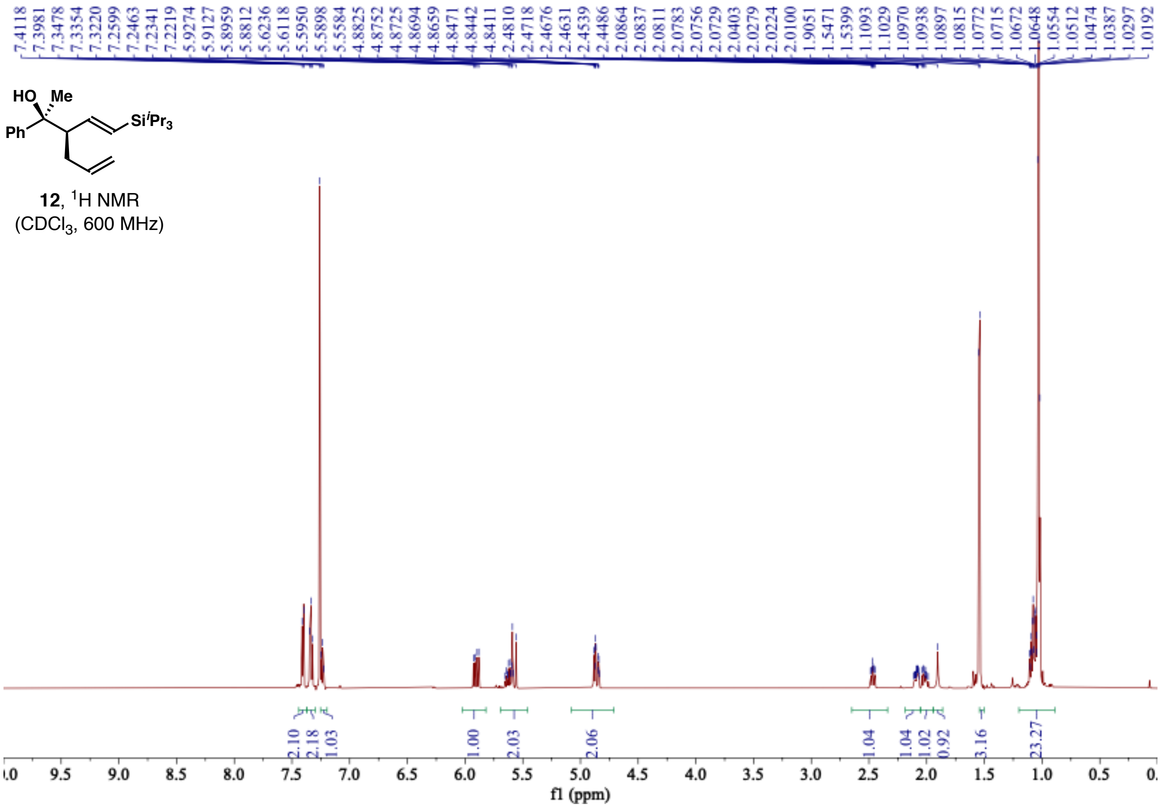

12, ^{1}H NMR
($CDCl_3$, 600 MHz)

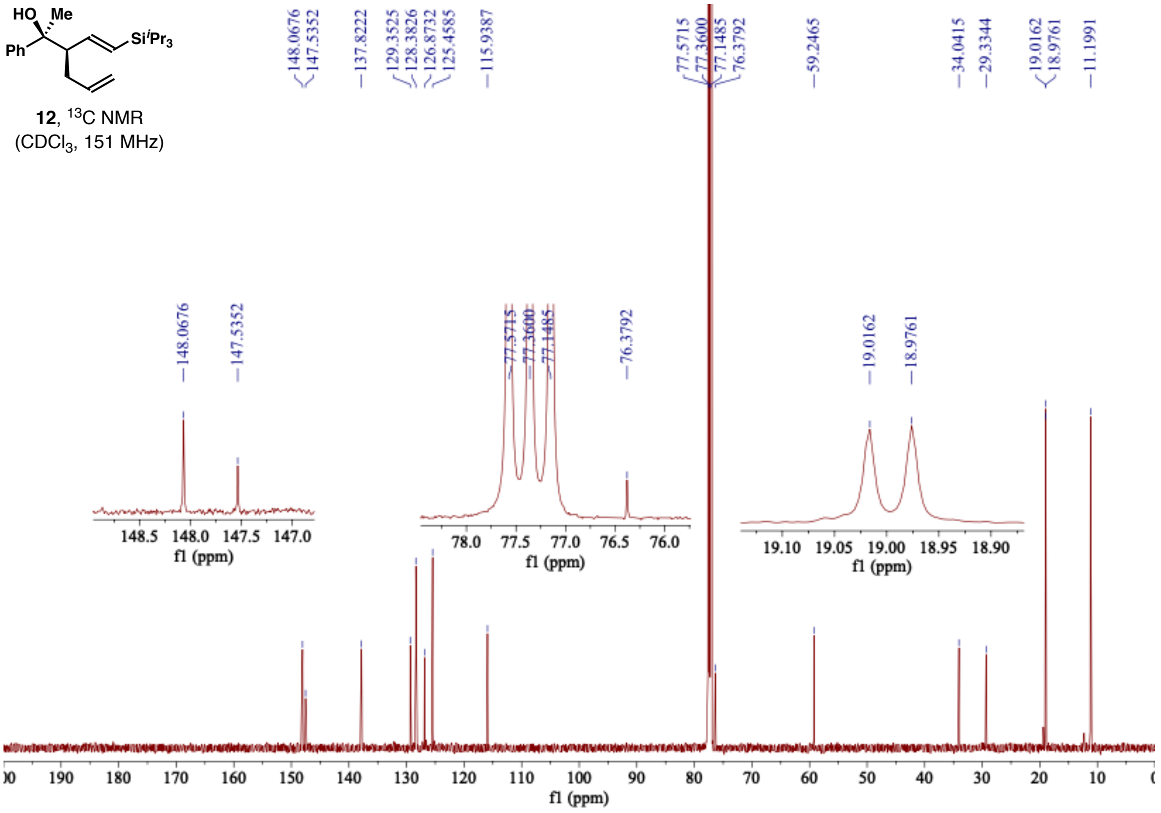

12, ^{13}C NMR
($CDCl_3$, 151 MHz)

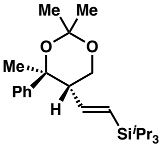

13, ^{1}H NMR
(CDCl$_3$, 500 MHz)

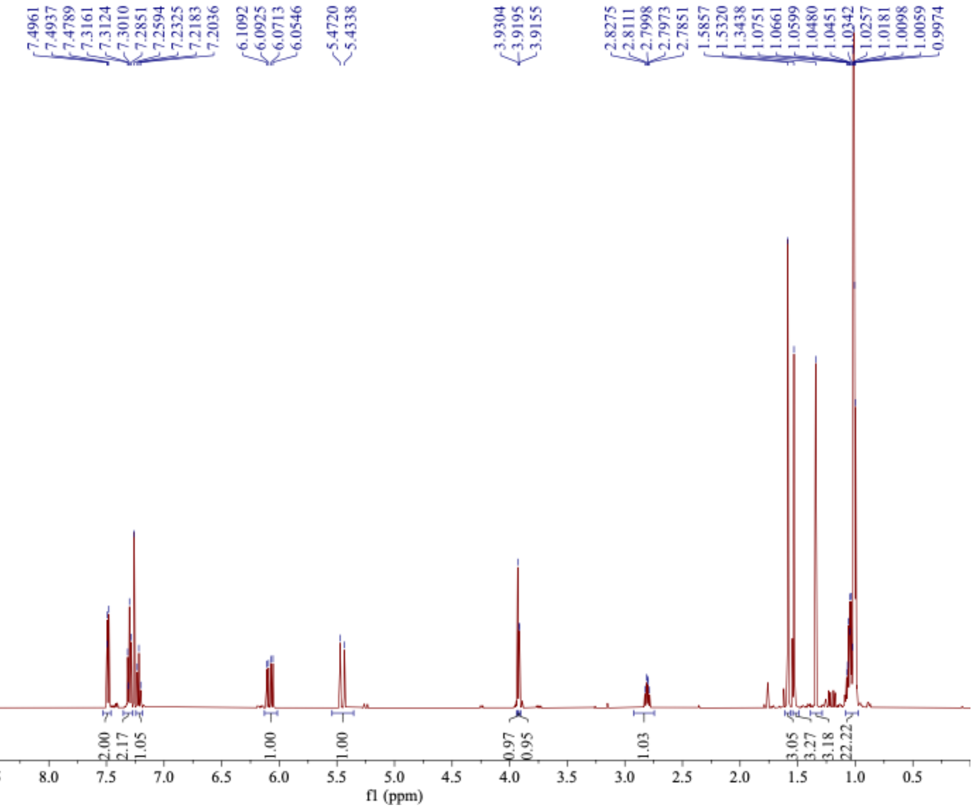

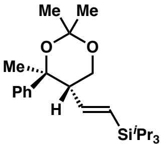

13, ^{13}C NMR
(CDCl$_3$, 126 MHz)

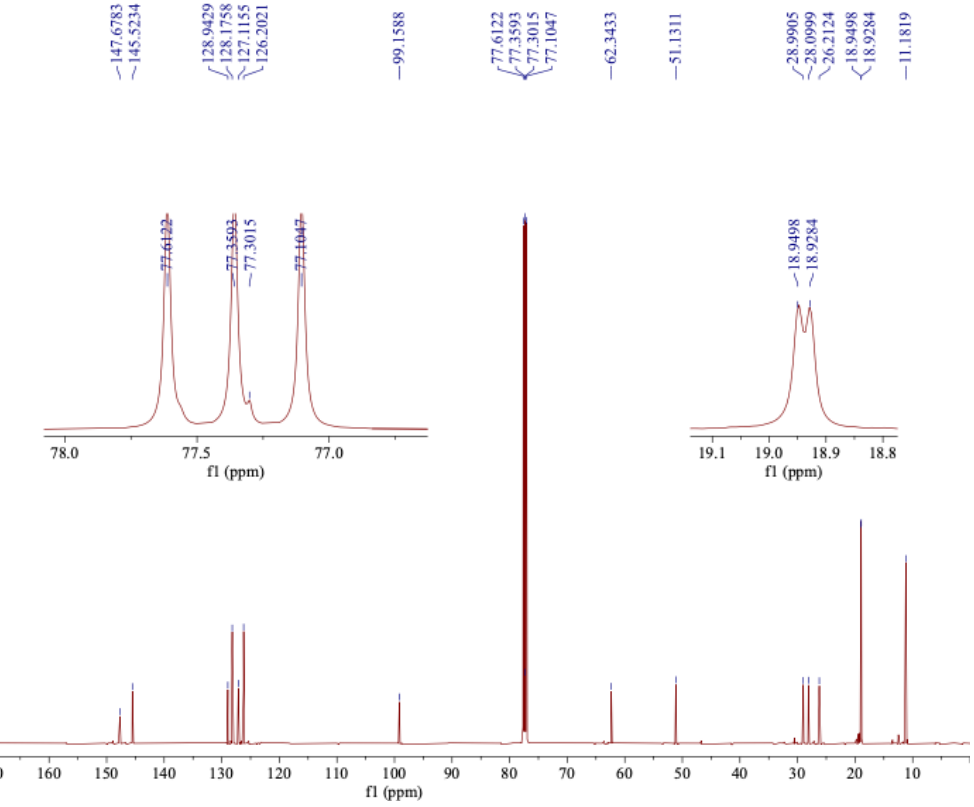

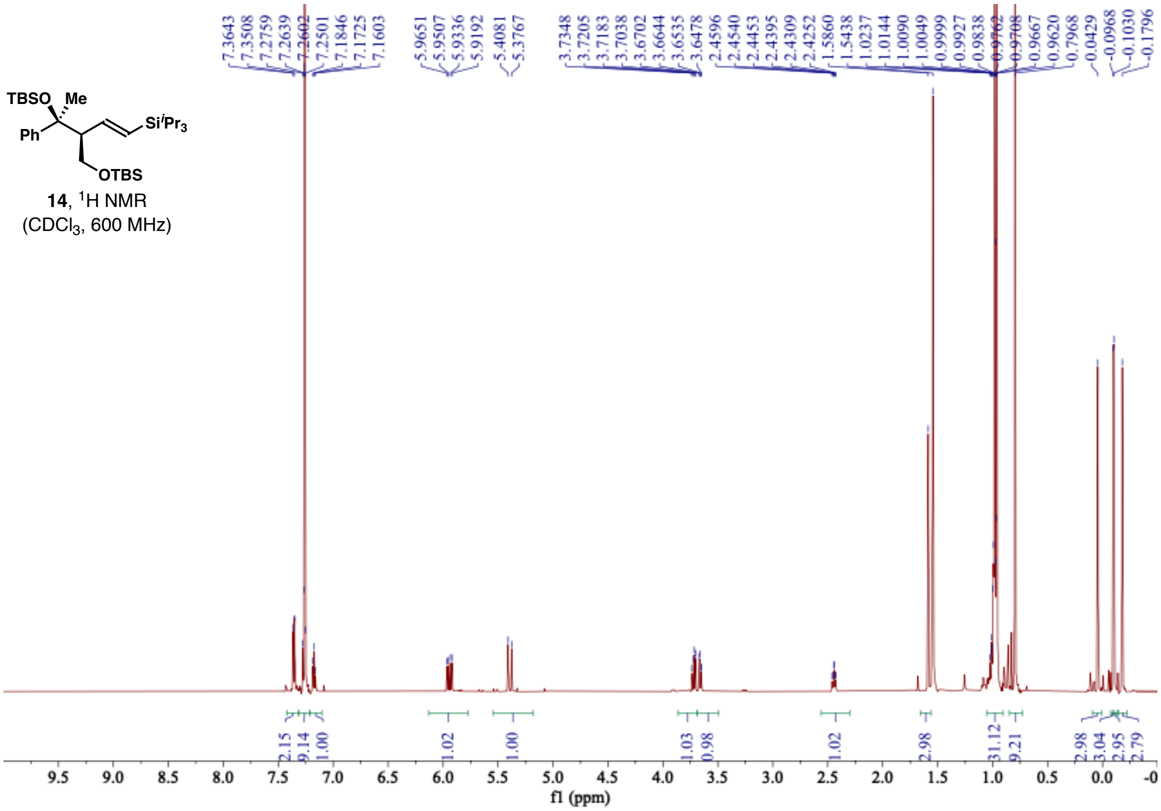

14, ^{1}H NMR (CDCl$_3$, 600 MHz)

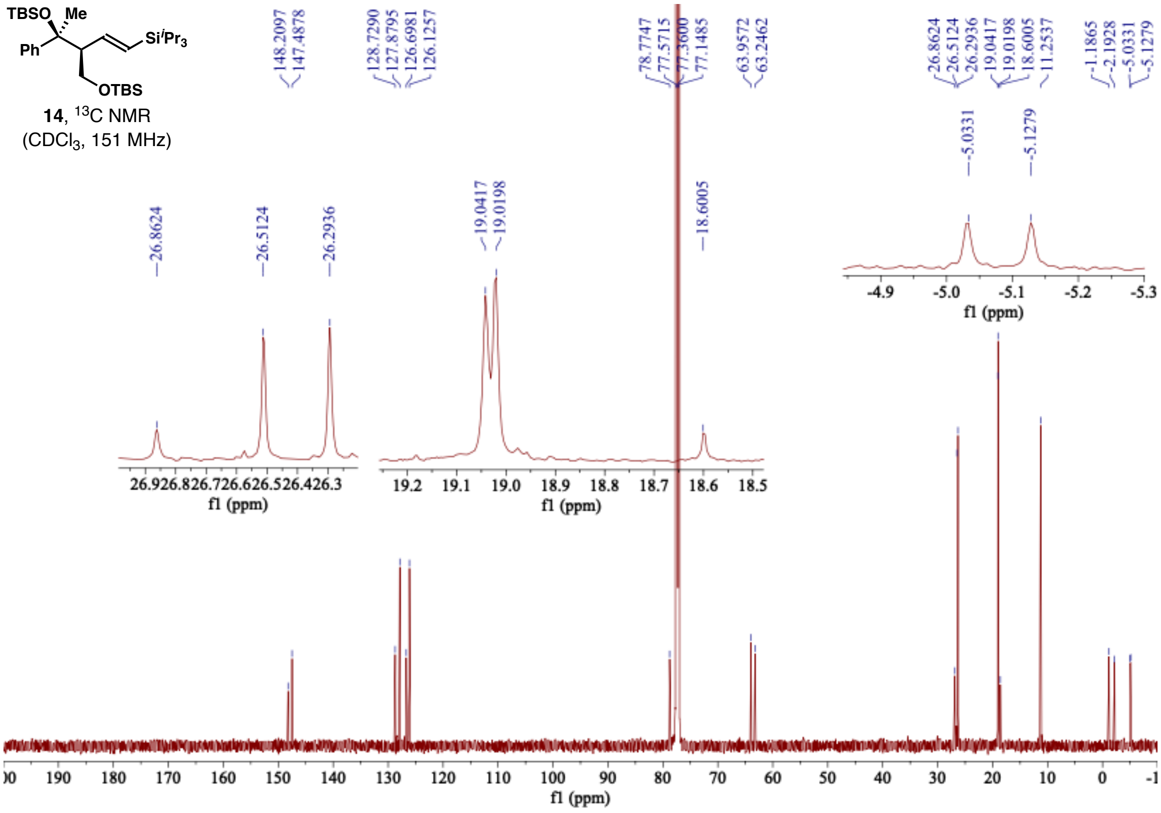

14, ^{13}C NMR (CDCl$_3$, 151 MHz)

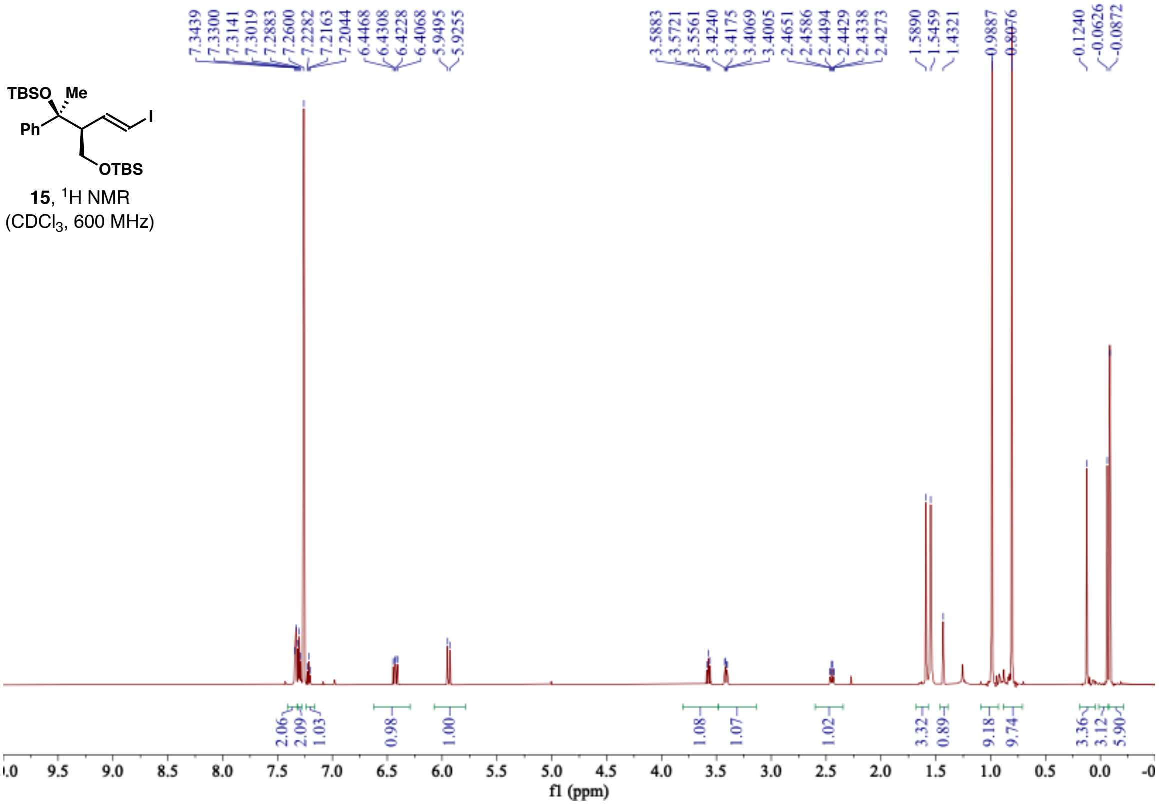
TBSO
Me
Ph
I
OTBS
15, ¹H NMR
(CDCl3, 600 MHz)
f1 (ppm)

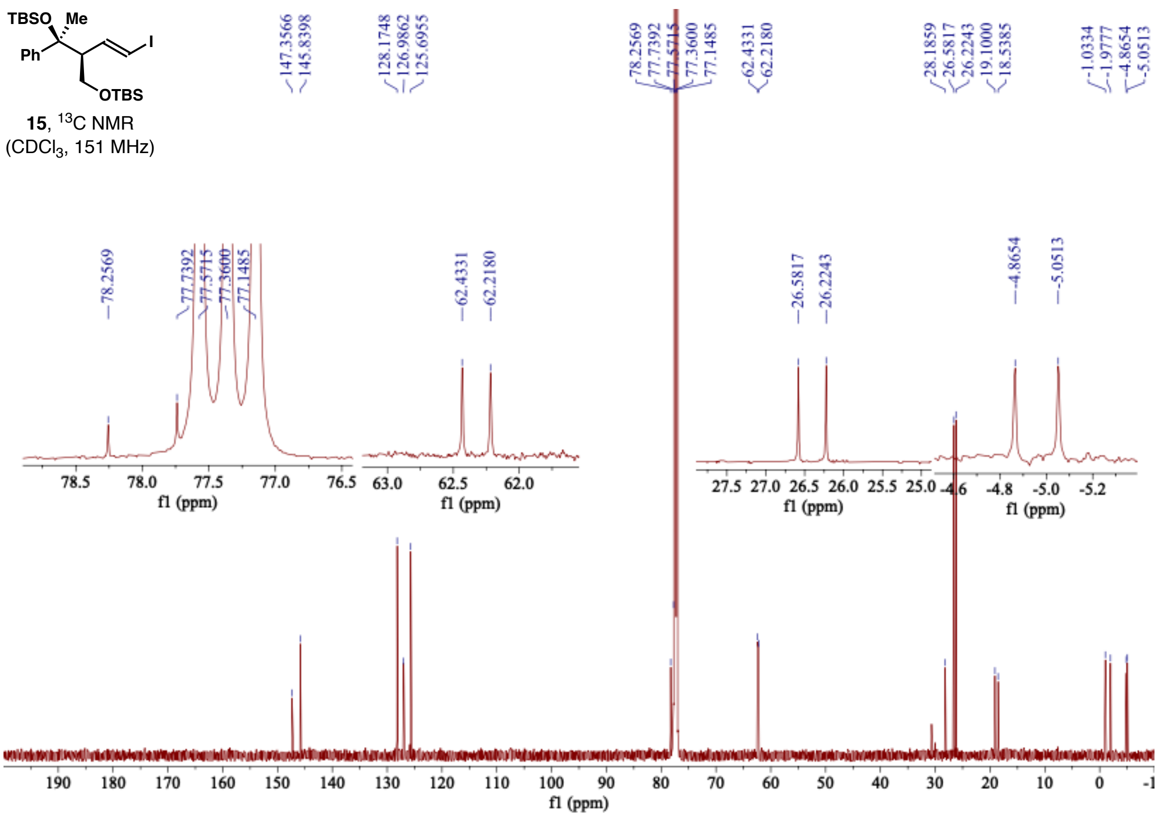
TBSO
Me
Ph
I
OTBS
15, ¹³C NMR
(CDCl3, 151 MHz)
f1 (ppm)

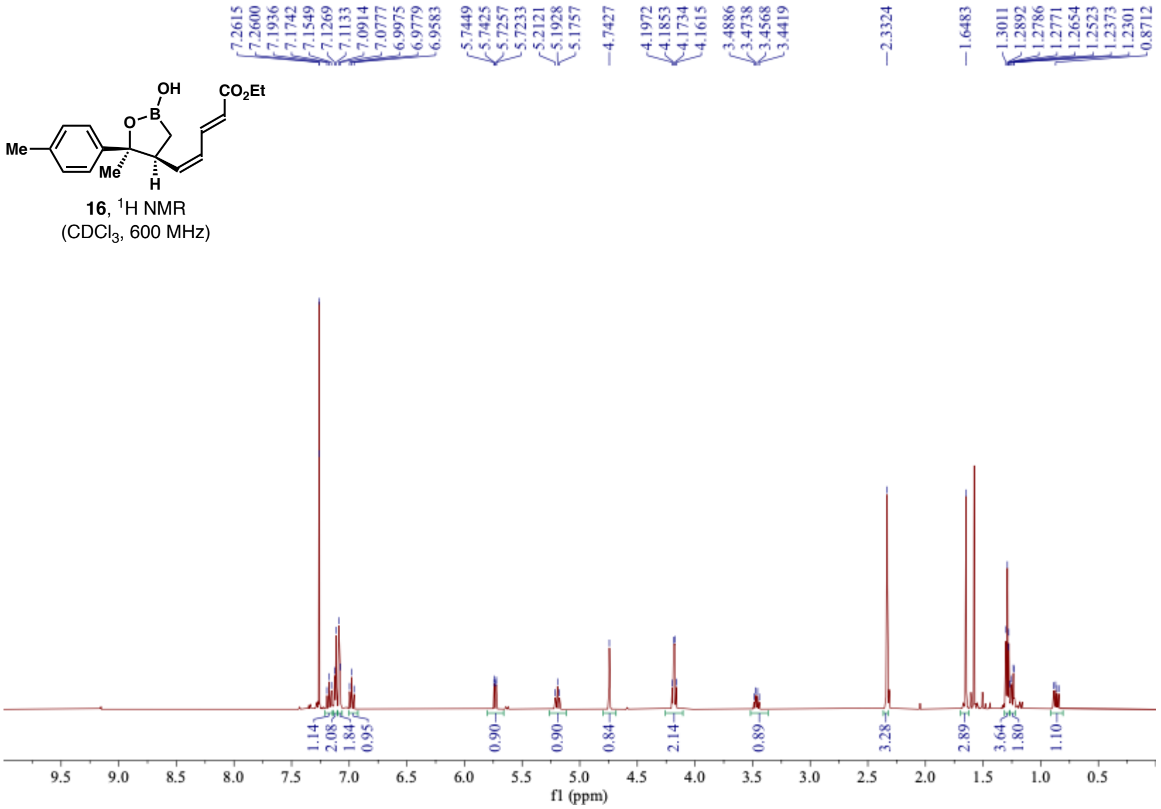
OH
CO2Et
O
B
Me
Me
H
16, 1H NMR
(CDCl3, 600 MHz)
f1 (ppm)

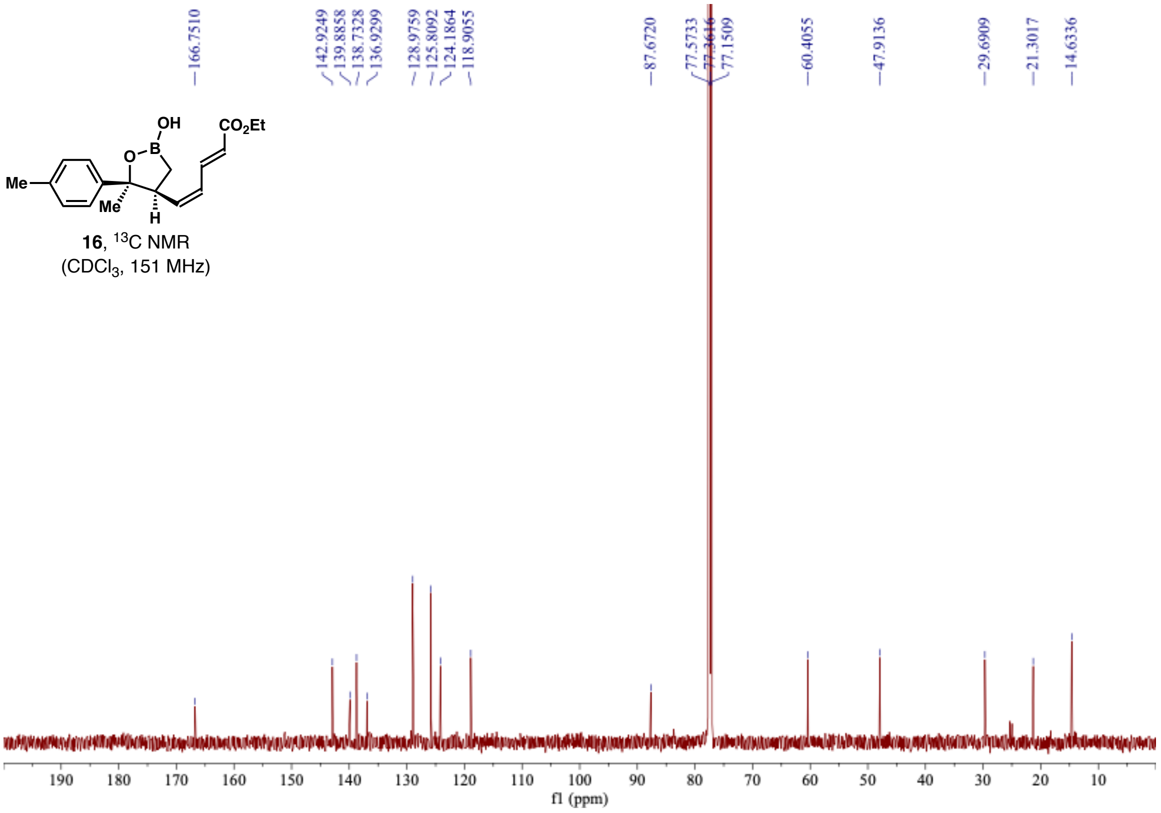
OH
CO2Et
O
B
Me
Me
H
16, 13C NMR
(CDCl3, 151 MHz)
f1 (ppm)

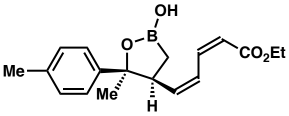

17, ^{1}H NMR
($CDCl_3$, 600 MHz)

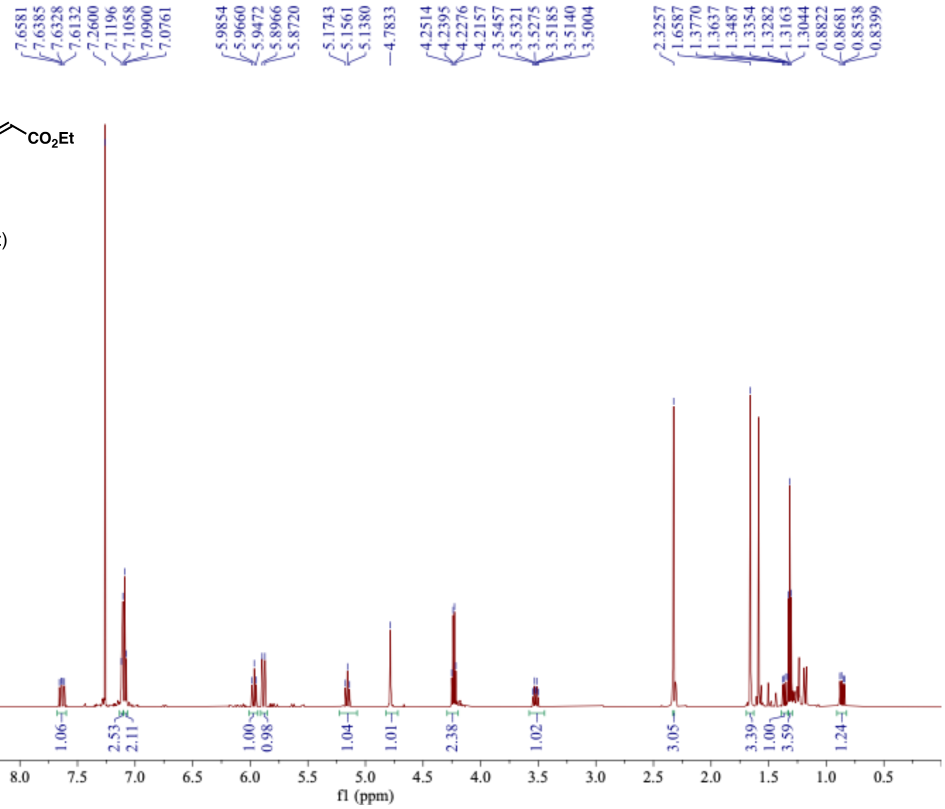

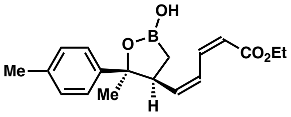

17, ^{13}C NMR
($CDCl_3$, 151 MHz)

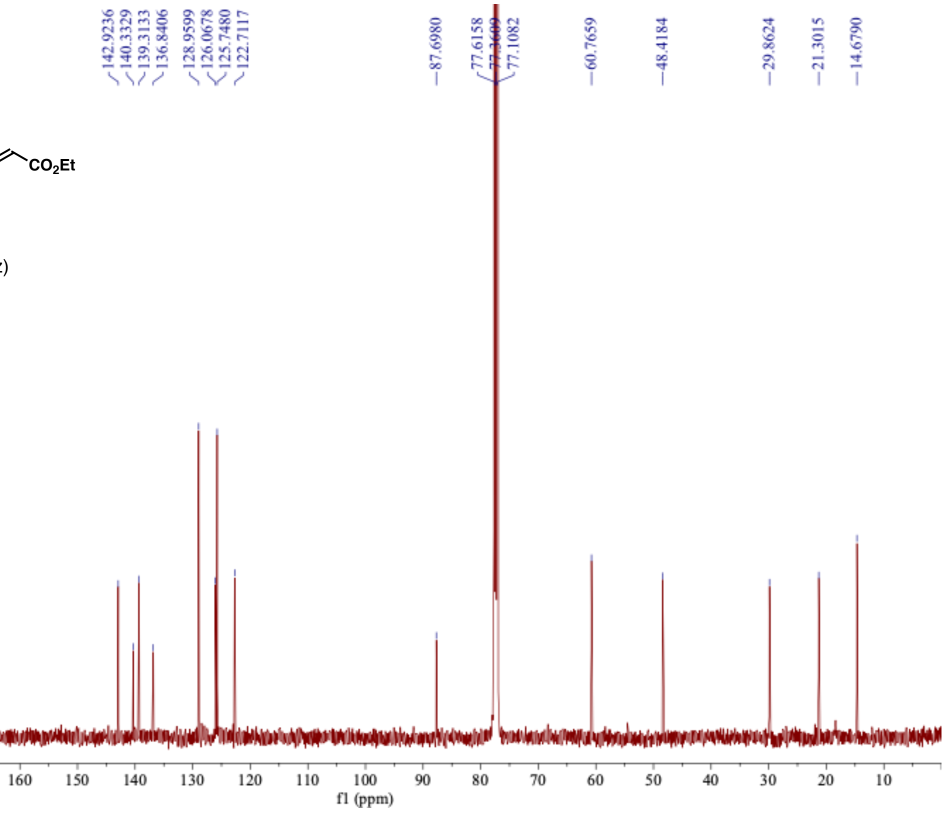

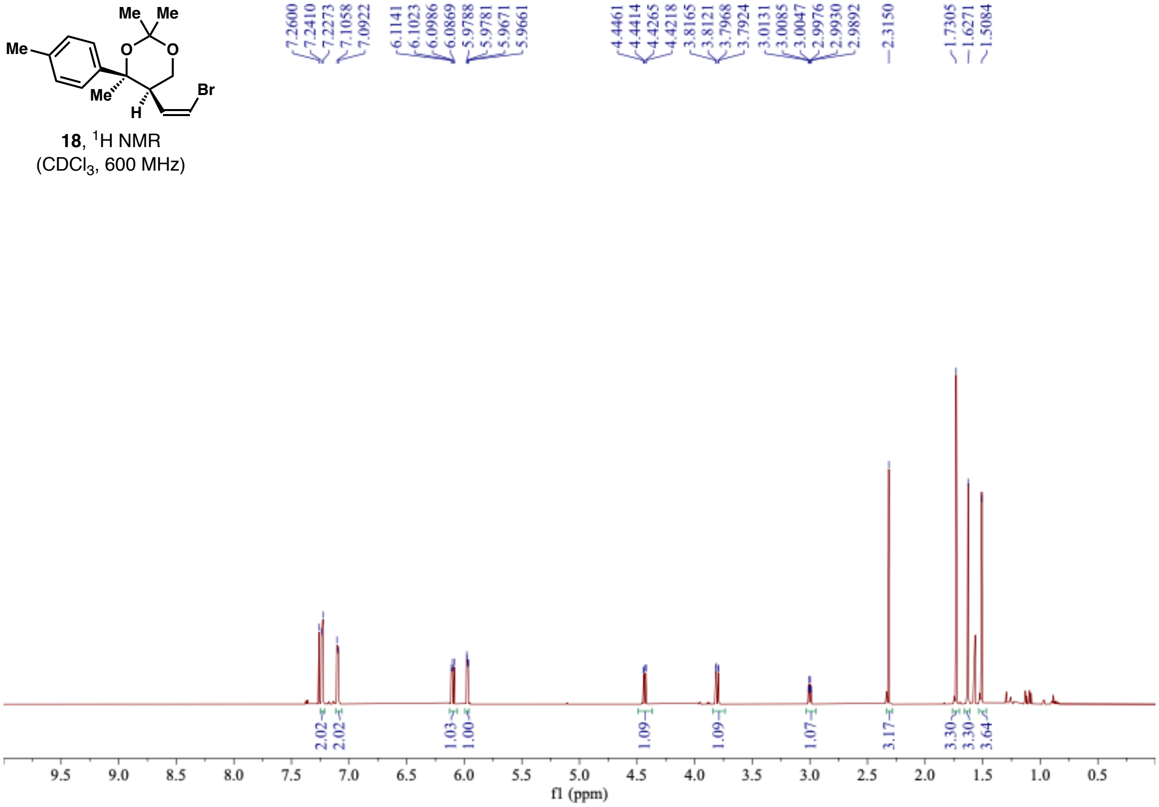

18, ^{1}H NMR
($CDCl_3$, 600 MHz)

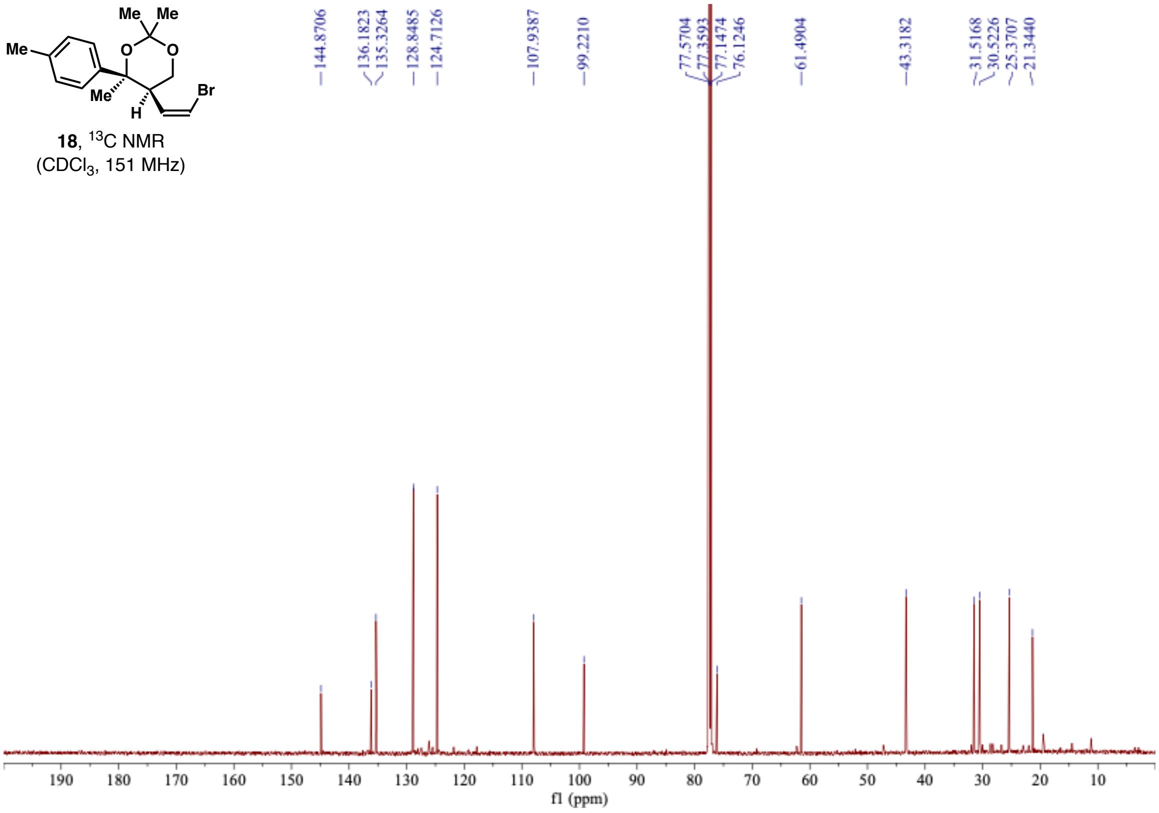

18, ^{13}C NMR
($CDCl_3$, 151 MHz)

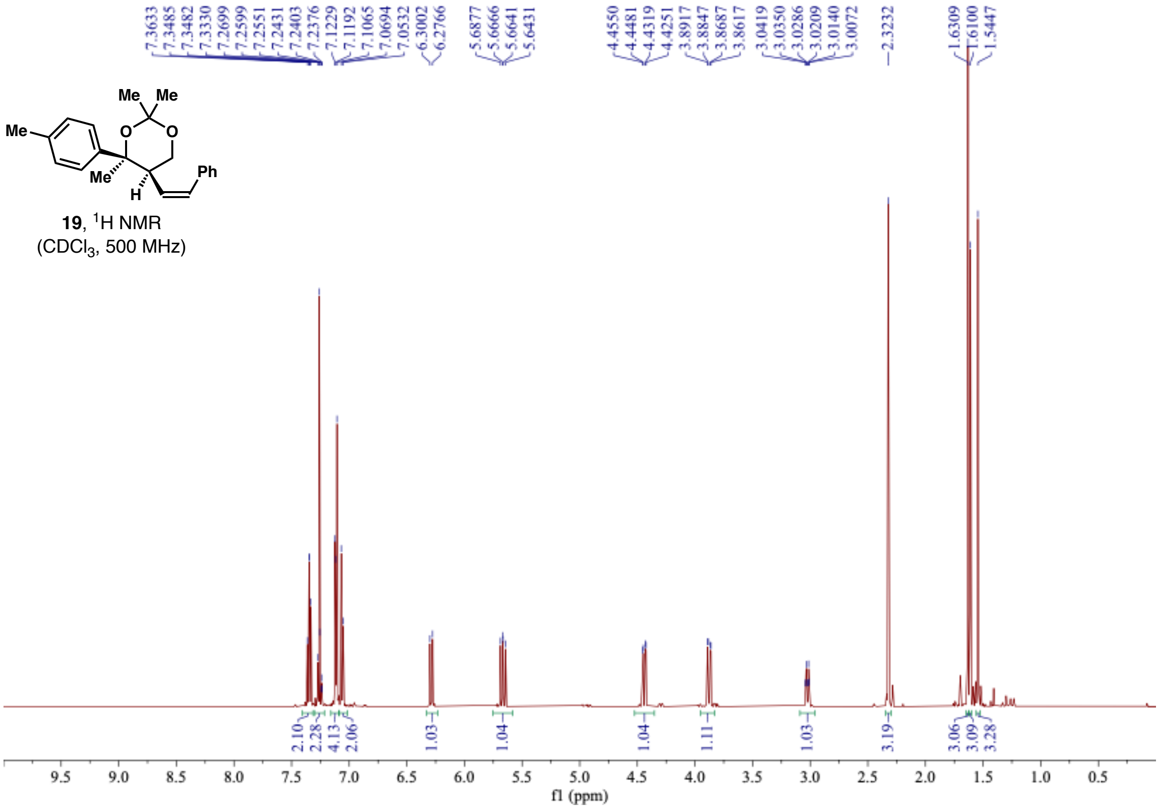

19, ^{1}H NMR (CDCl$_3$, 500 MHz)

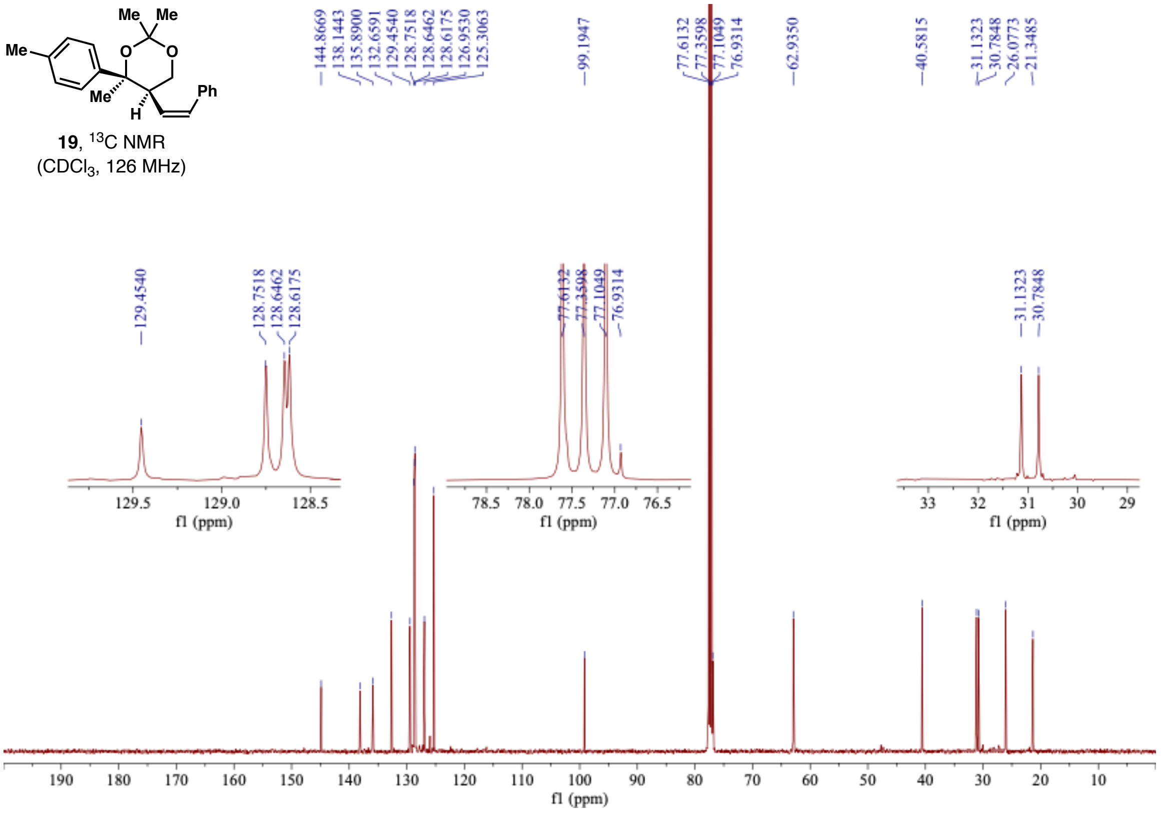

19, ^{13}C NMR (CDCl$_3$, 126 MHz)
