# Supplementary material for: Asymmetric Ketone Diene Coupling via Stereodivergent Copper Catalysis
Source: J Am Chem Soc. 2026 Feb 2;148(5):4899–912. doi: 10.1021/jacs.5c06735 (PMC12903869; doi:10.1021/jacs.5c06735)

Supporting Information -- HPLC spectra of diols 2 and 5

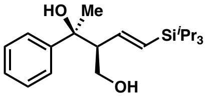

Enantiomeric excess was determined by HPLC analysis to be 94% ee (254 nm, 25 ˚C); t_1 = 4.64 min, t_2 = 5.01 min [(Chiralpak IC) hexane/i-PrOH, 90:10, 1.0 mL/min].

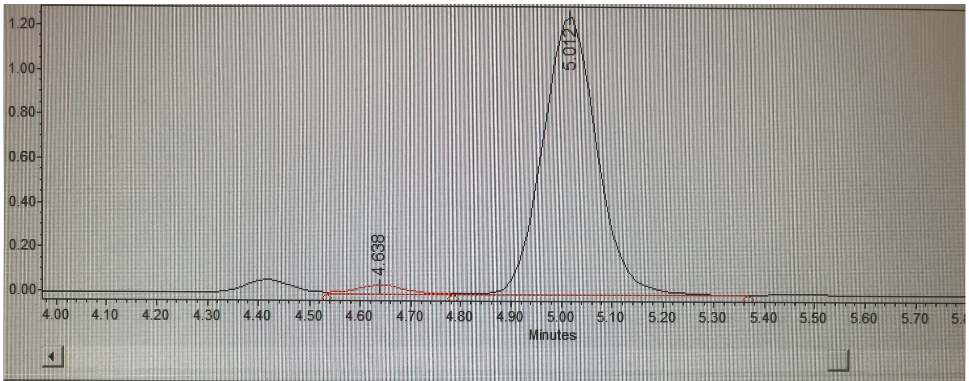

[illegible]	Retention Time (min)	Area (µV*sec)	% Area	Height (µV)	Int Type	Amount	Units	Peak Type	Peak Codes
	4.638	270817	2.84	38819	VV			Unknown	
	5.012	9279020	97.16	1250374	VV			Unknown	

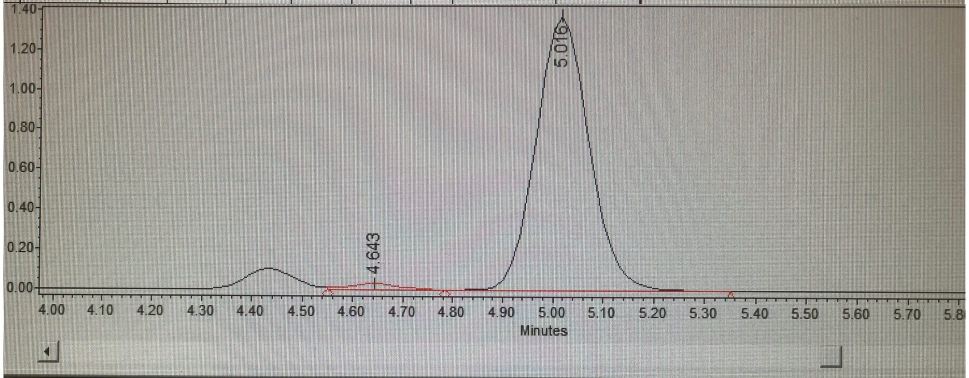

[illegible]	Retention Time (min)	Area (µV*sec)	% Area	Height (µV)	Int Type	Amount	Units	Peak Type	Peak Codes
	4.643	221942	2.17	32262	VV			Unknown	
	5.016	10022323	97.83	1361742	VB			Unknown	

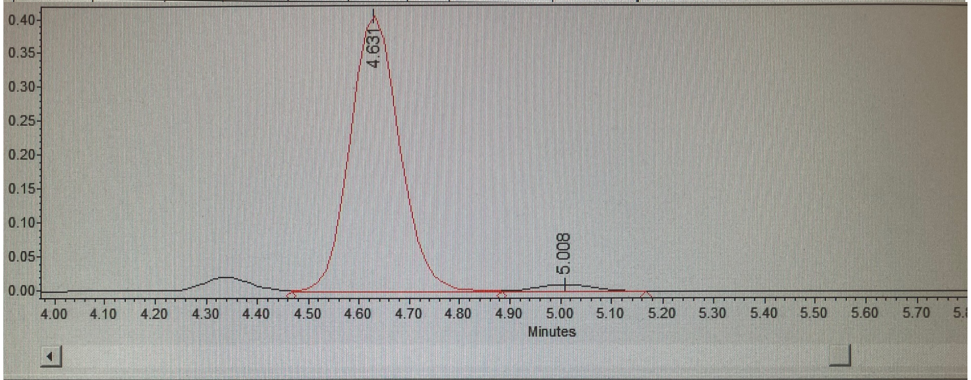

[illegible]	Retention Time (min)	Area (µV*sec)	% Area	Height (µV)	Int Type	Amount	Units	Peak Type	Peak Codes
	4.631	2763767	97.26	403309	VV			Unknown	
	5.008	78004	2.74	10499	VV			Unknown	

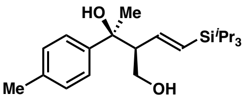

Enantiomeric excess was determined by HPLC analysis to be 93% ee (254 nm, 25 ˚C); t_1 = 5.57 min, t_2 = 6.01 min [(Chiralpak ID) hexane/i-PrOH, 95:5, 1.0 mL/min].

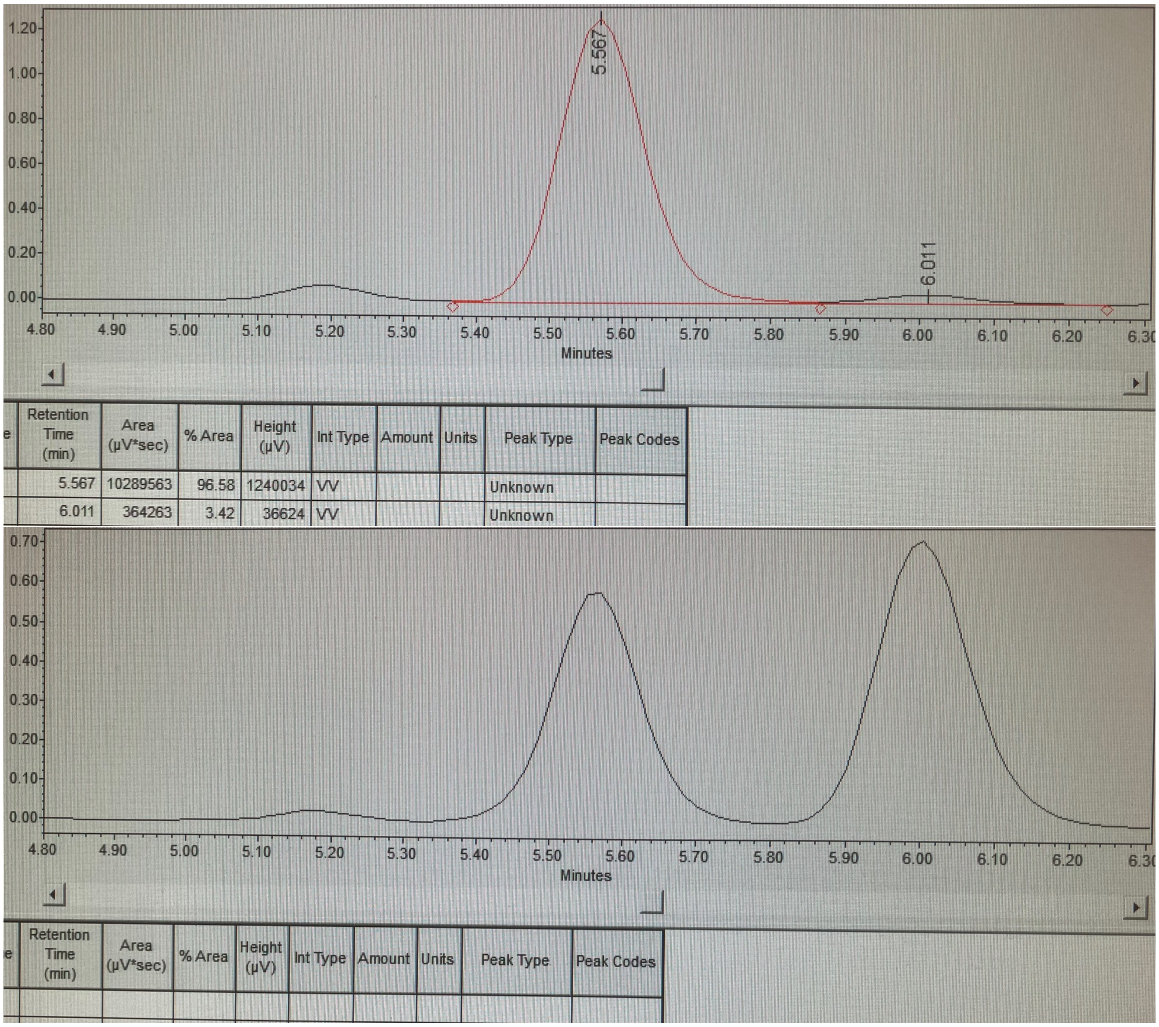

Retention Time (min)	Area (µV*sec)	% Area	Height (µV)	Int Type	Amount	Units	Peak Type	Peak Codes
5.567	10289563	96.58	1240034	VV			Unknown	
6.011	364263	3.42	36624	VV			Unknown	

Retention Time (min)	Area (µV*sec)	% Area	Height (µV)	Int Type	Amount	Units	Peak Type	Peak Codes

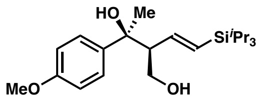

Enantiomeric excess was determined by HPLC analysis to be 94% ee (254 nm, 25 ˚C); t_1 = 8.74 min, t_2 = 9.40 min [(Chiralpak IC) hexane/i-PrOH, 95:5, 1.0 mL/min].

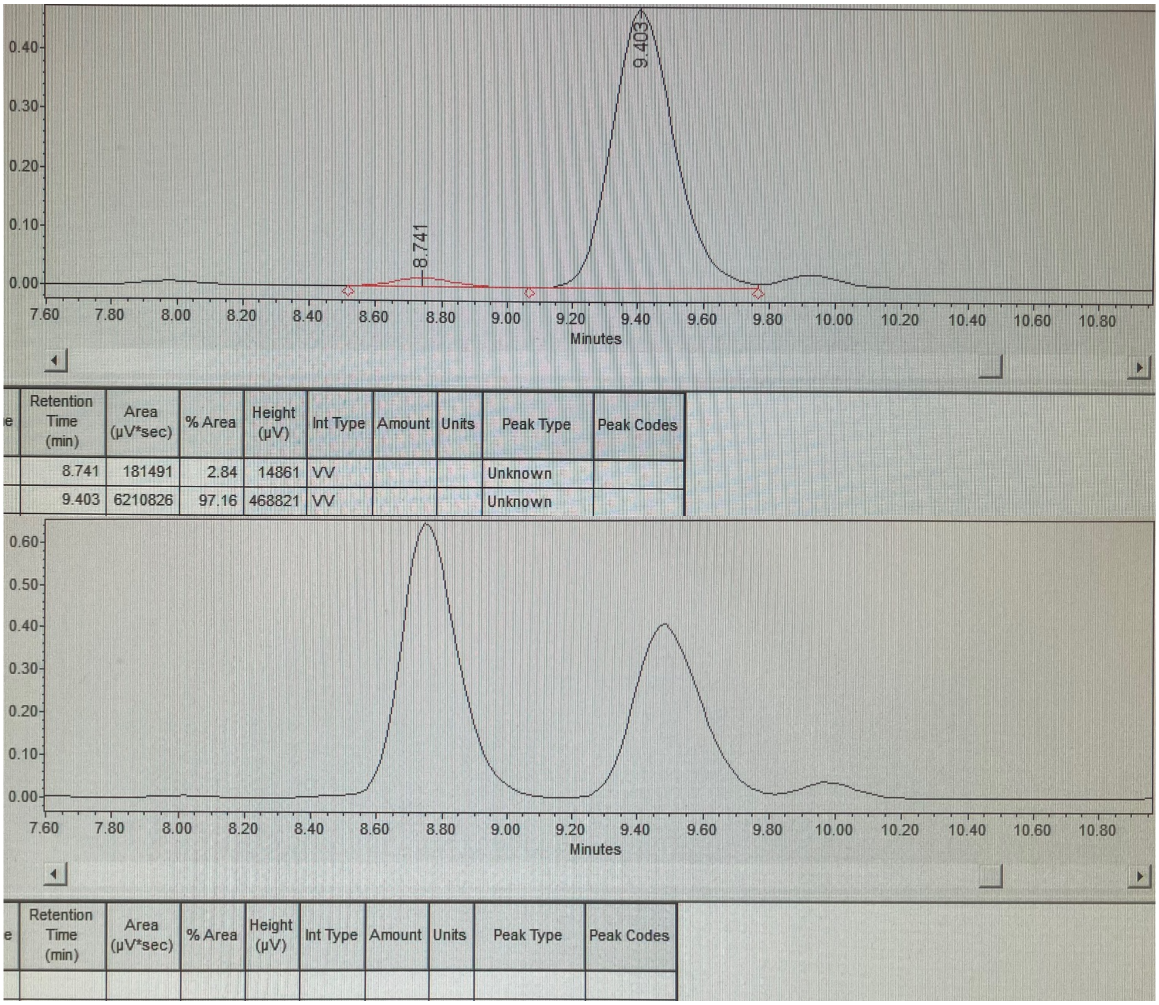

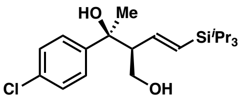

Enantiomeric excess was determined by HPLC analysis to be 95% ee (254 nm, 25 ˚C); t_1 = 4.97 min, t_2 = 5.29 min [(Chiralpak ID) hexane/i-PrOH, 95:5, 1.0 mL/min].

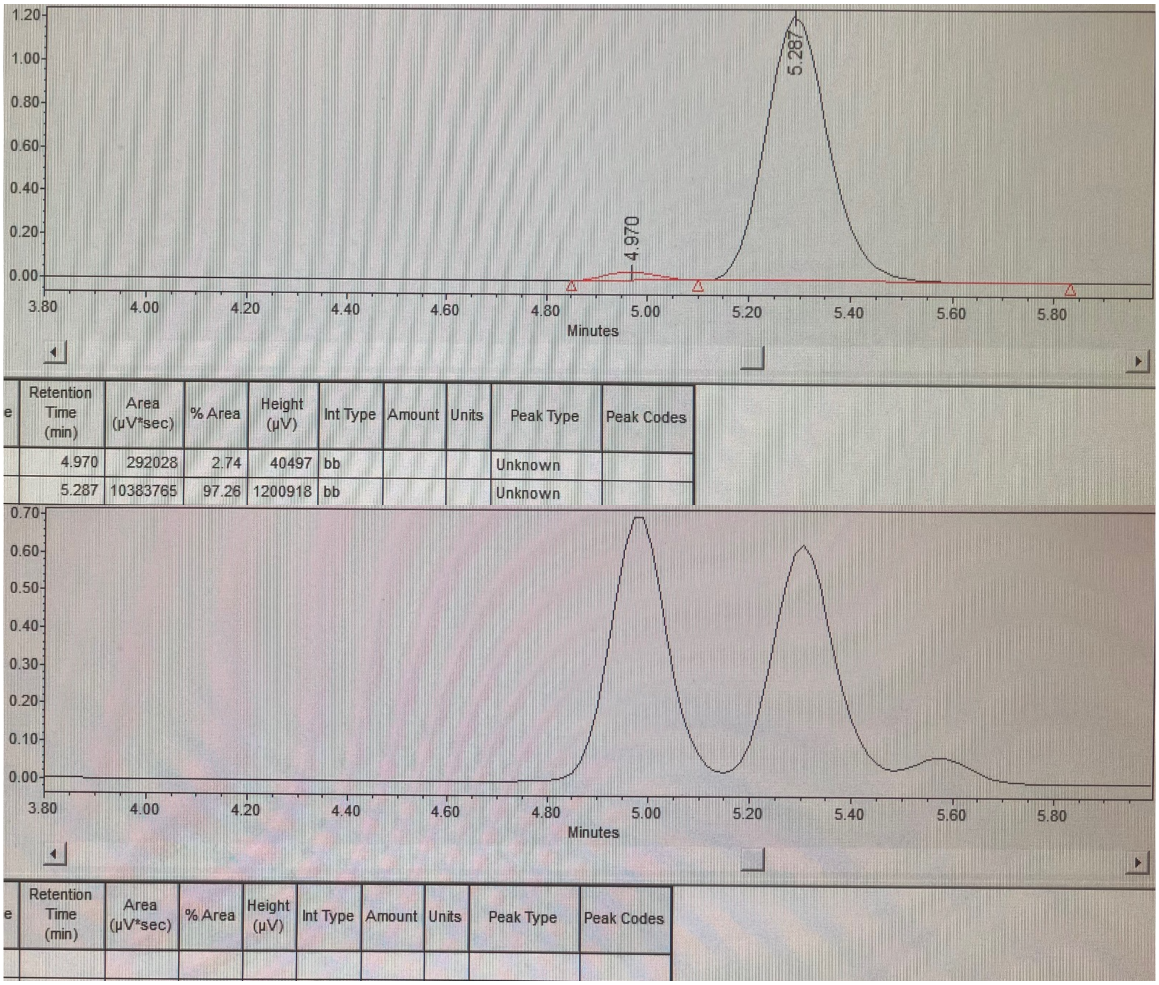

Retention Time (min)	Area (µV*sec)	% Area	Height (µV)	Int Type	Amount	Units	Peak Type	Peak Codes
4.970	292028	2.74	40497	bb			Unknown	
5.287	10383765	97.26	1200918	bb			Unknown	

Retention Time (min)	Area (µV*sec)	% Area	Height (µV)	Int Type	Amount	Units	Peak Type	Peak Codes

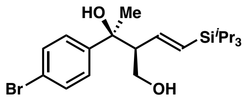

Enantiomeric excess was determined by HPLC analysis to be 93% ee (254 nm, 25 ˚C); t_1 = 6.27 min, t_2 = 6.71 min [(Chiralpak IC) hexane/i-PrOH, 95:5, 1.0 mL/min].

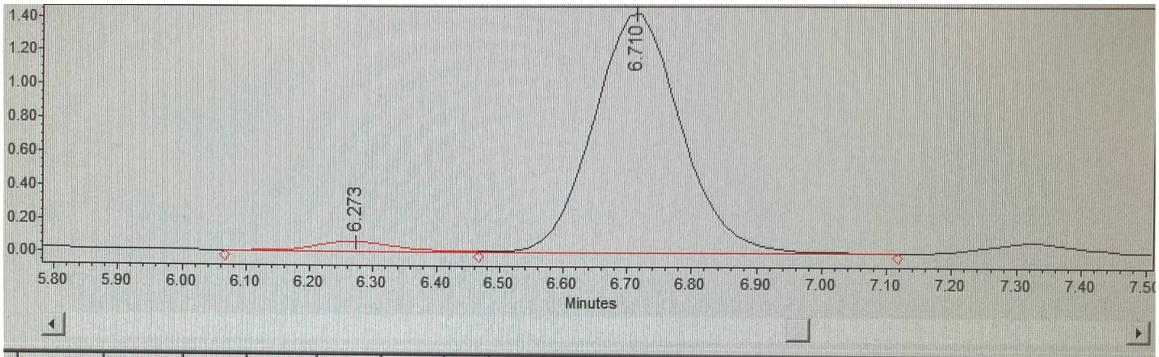

Retention Time (min)	Area (µV*sec)	% Area	Height (µV)	Int Type	Amount	Units	Peak Type	Peak Codes
6.273	509752	3.68	56538	VV			Unknown	
6.710	13331512	96.32	1410956	VV			Unknown	

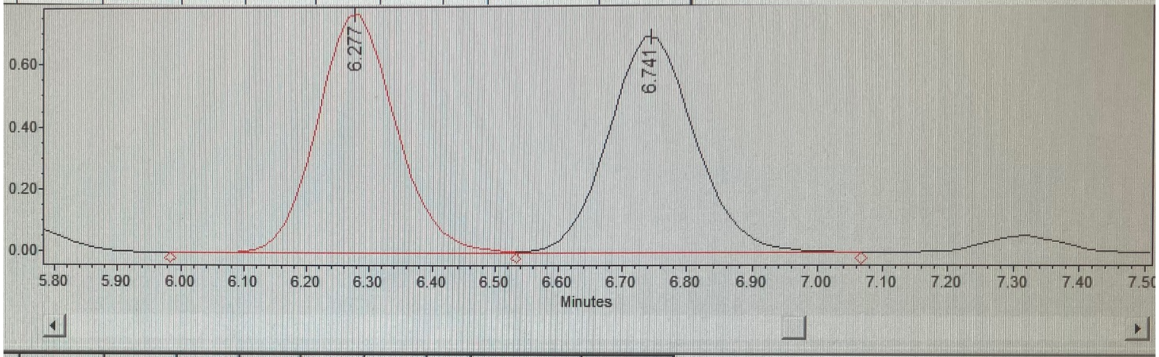

Retention Time (min)	Area (µV*sec)	% Area	Height (µV)	Int Type	Amount	Units	Peak Type	Peak Codes
6.277	6714895	51.00	763999	VV			Unknown	
6.741	6451945	49.00	689902	VV			Unknown	

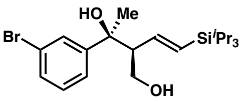

Enantiomeric excess was determined by HPLC analysis to be 90% ee (254 nm, 25 ˚C); t_1 = 8.69 min, t_2 = 9.70 min [(Chiralpak IC) hexane/i-PrOH, 97:3, 1.0 mL/min].

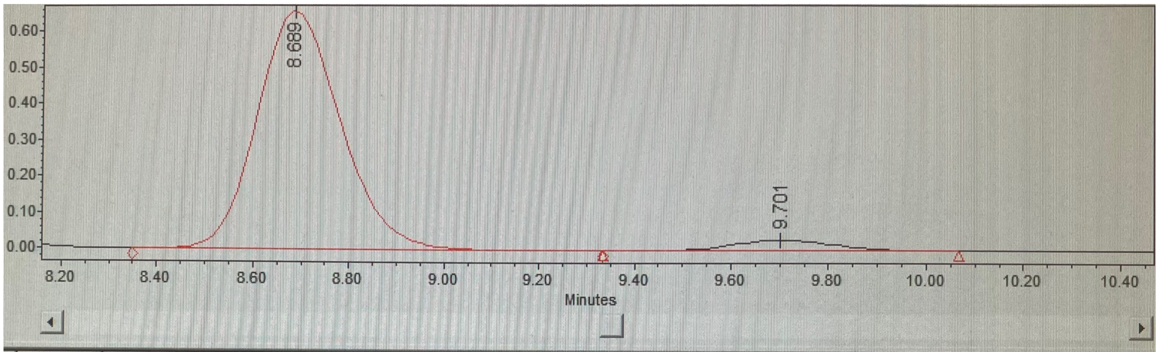

Retention Time (min)	Area (µV*sec)	% Area	Height (µV)	Int Type	Amount	Units	Peak Type	Peak Codes
8.689	7981170	94.98	655432	VV			Unknown	
9.701	422085	5.02	30123	bb			Unknown	

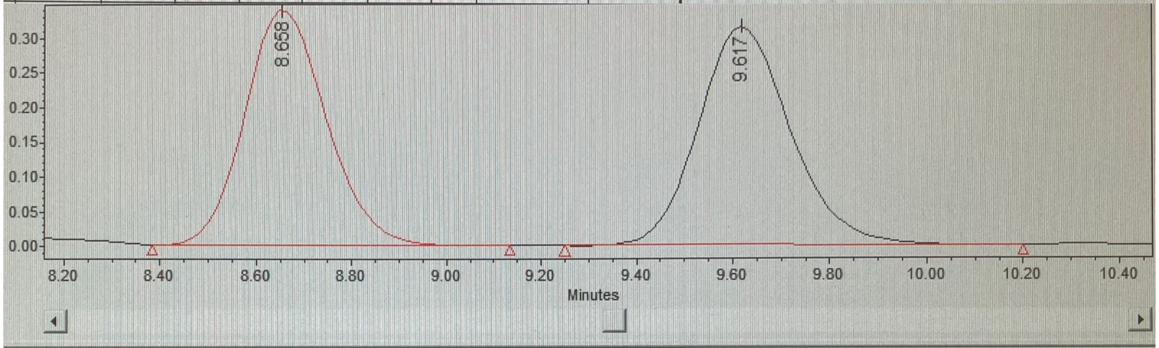

Retention Time (min)	Area (µV*sec)	% Area	Height (µV)	Int Type	Amount	Units	Peak Type	Peak Codes
8.658	4022127	49.27	339273	bb			Unknown	
9.617	4141462	50.73	313276	bb			Unknown	

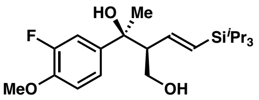

Enantiomeric excess was determined by HPLC analysis to be 91% ee (254 nm, 25 ˚C); t_1 = 8.52 min, t_2 = 9.13 min [(Chiralpak IC) hexane/i-PrOH, 95:5, 1.0 mL/min].

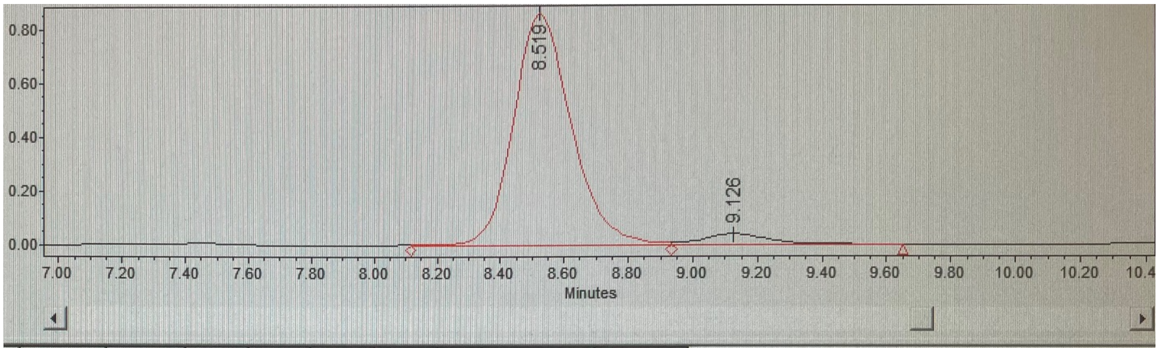

e	Retention Time (min)	Area (µV*sec)	% Area	Height (µV)	Int Type	Amount	Units	Peak Type	Peak Codes
	8.519	10758097	95.44	855392	VV			Unknown	
	9.126	513672	4.56	37742	VB			Unknown	

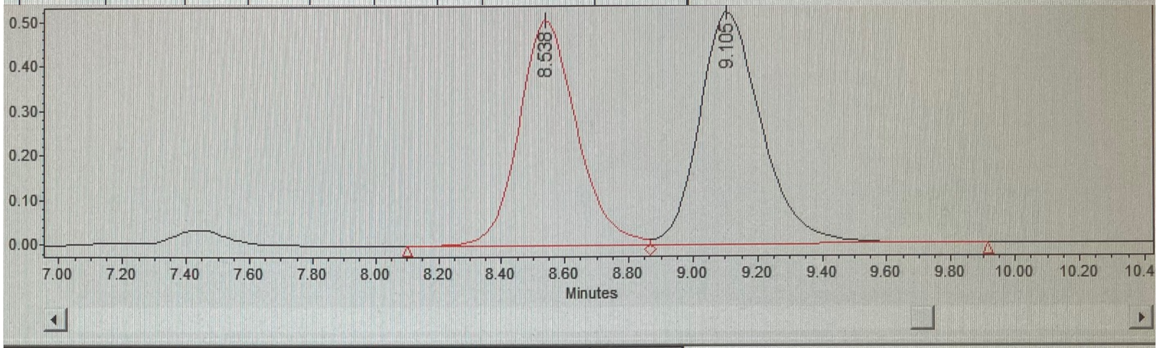

e	Retention Time (min)	Area (µV*sec)	% Area	Height (µV)	Int Type	Amount	Units	Peak Type	Peak Codes
	8.538	6269894	47.81	500208	BV			Unknown	
	9.105	6844757	52.19	515098	VB			Unknown	

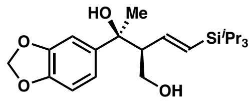

Enantiomeric excess was determined by HPLC analysis to be 95% ee (254 nm, 25 ˚C); t_1 = 10.1 min, t_2 = 13.4 min [(Chiralpak IC) hexane/i-PrOH, 95:5, 1.0 mL/min].

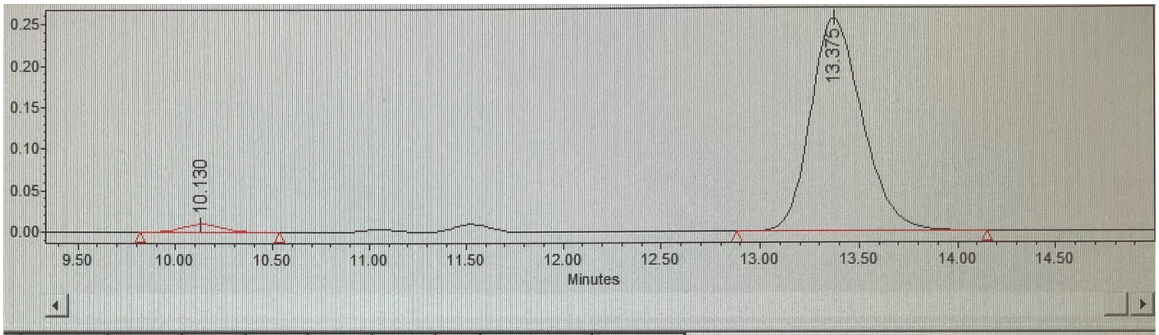

e	Retention Time (min)	Area (µV*sec)	% Area	Height (µV)	Int Type	Amount	Units	Peak Type	Peak Codes
	10.130	130701	2.72	9503	bb			Unknown	
	13.375	4666118	97.28	255958	BB			Unknown	

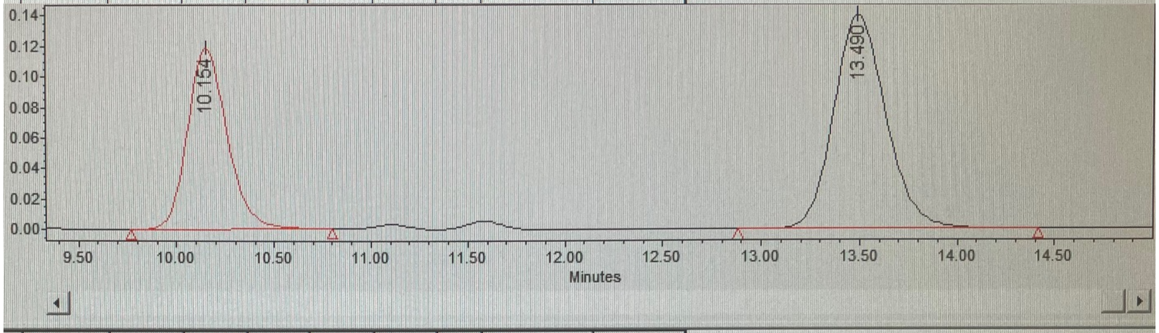

e	Retention Time (min)	Area (µV*sec)	% Area	Height (µV)	Int Type	Amount	Units	Peak Type	Peak Codes
	10.154	1669077	39.20	118775	bb			Unknown	
	13.490	2588770	60.80	140111	bb			Unknown	

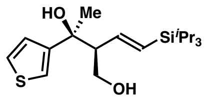

Enantiomeric excess was determined by HPLC analysis to be 95% ee (254 nm, 25 ˚C); t_1 = 6.72 min, t_2 = 7.32 min [(Chiralpak IC) hexane/i-PrOH, 95:5, 1.0 mL/min].

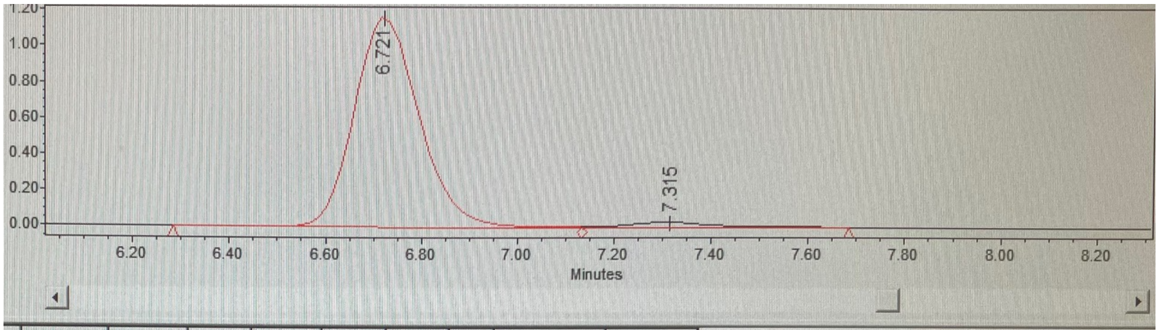

[illegible]	Retention Time (min)	Area (µV*sec)	% Area	Height (µV)	Int Type	Amount	Units	Peak Type	Peak Codes
	6.721	10901353	97.51	1159237	BV			Unknown	
	7.315	277942	2.49	27248	VB			Unknown	

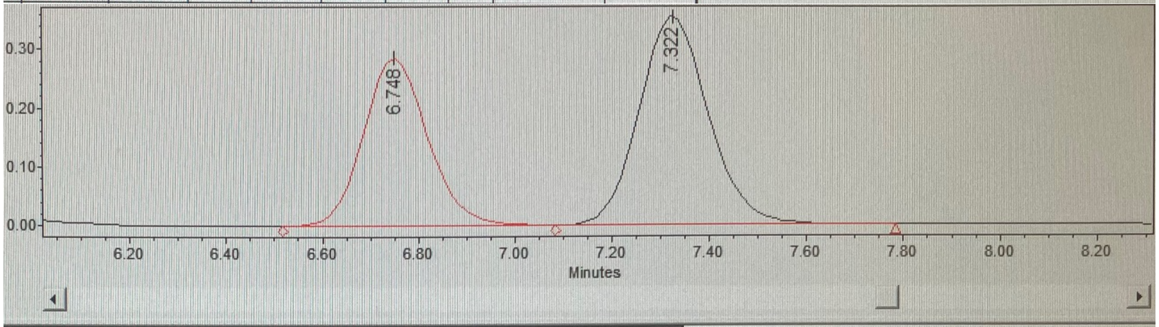

[illegible]	Retention Time (min)	Area (µV*sec)	% Area	Height (µV)	Int Type	Amount	Units	Peak Type	Peak Codes
	6.748	2652286	43.08	284382	VV			Unknown	
	7.322	3505004	56.92	353990	VB			Unknown	

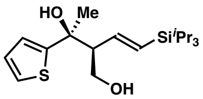

Enantiomeric excess was determined by HPLC analysis to be 95% ee (254 nm, 25 ˚C); t_1 = 6.91 min, t_2 = 7.54 min [(Chiralpak IC) hexane/i-PrOH, 95:5, 1.0 mL/min].

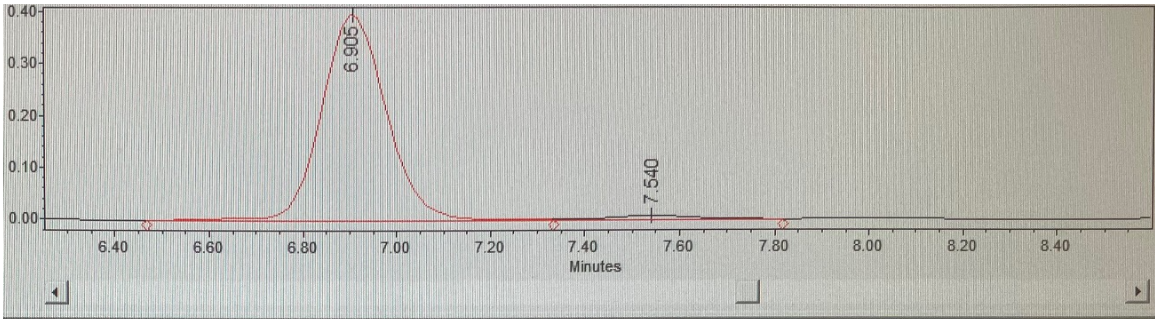

ne	Retention Time (min)	Area (µV*sec)	% Area	Height (µV)	Int Type	Amount	Units	Peak Type	Peak Codes
	6.905	3758714	97.69	395059	VV			Unknown	
	7.540	88775	2.31	8283	VV			Unknown	

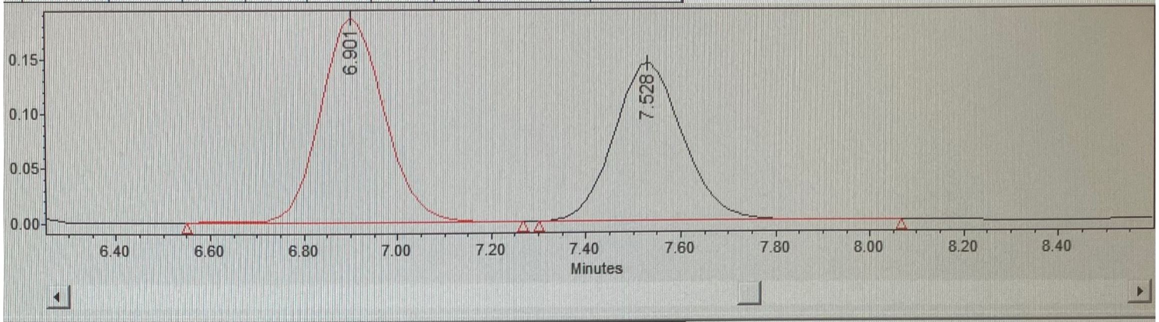

ne	Retention Time (min)	Area (µV*sec)	% Area	Height (µV)	Int Type	Amount	Units	Peak Type	Peak Codes
	6.901	1757880	54.73	186308	bb			Unknown	
	7.528	1453973	45.27	143713	bb			Unknown	

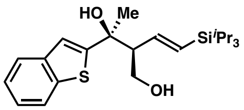

Enantiomeric excess was determined by HPLC analysis to be 93% ee (254 nm, 25 ˚C); t_1 = 7.03 min, t_2 = 7.60 min [(Chiralpak ID) hexane/i-PrOH, 95:5, 1.0 mL/min].

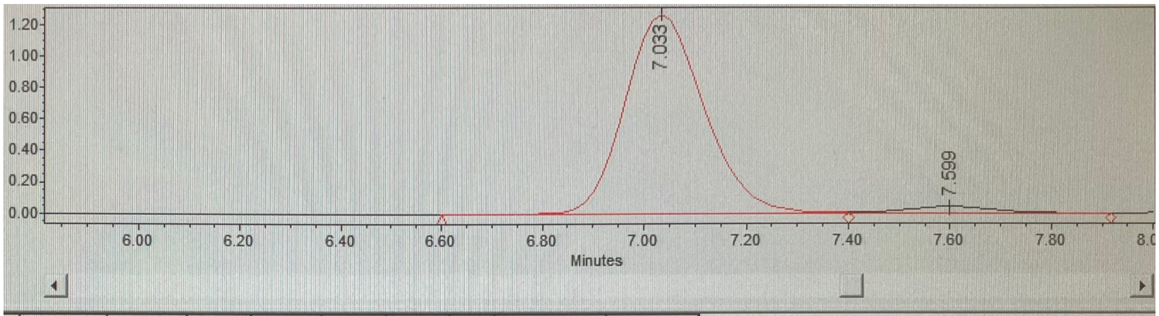

Retention Time (min)	Area (μV*sec)	% Area	Height (μV)	Int Type	Amount	Units	Peak Type	Peak Codes
7.033	13566161	96.46	1267592	BV			Unknown	
7.599	498513	3.54	41150	VV			Unknown	

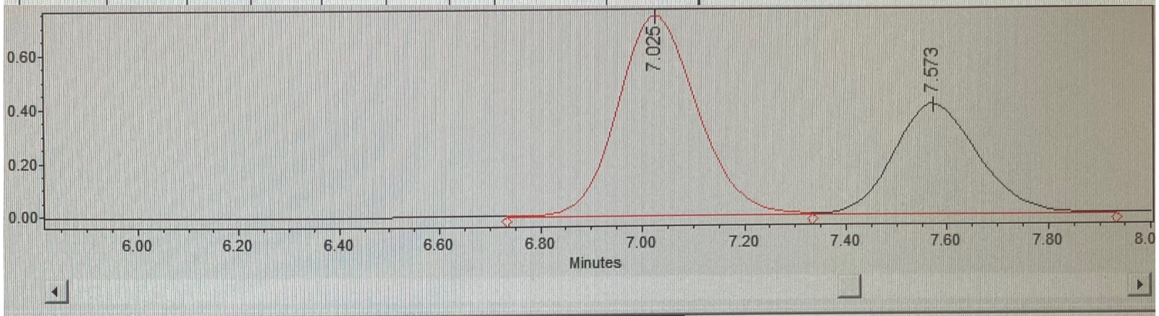

Retention Time (min)	Area (μV*sec)	% Area	Height (μV)	Int Type	Amount	Units	Peak Type	Peak Codes
7.025	7995757	62.62	742525	VV			Unknown	
7.573	4772425	37.38	409927	VV			Unknown	

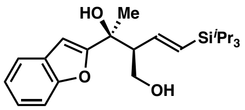

Enantiomeric excess was determined by HPLC analysis to be 90% ee (254 nm, 25 ˚C); t_1 = 7.81 min, t_2 = 8.76 min [(Chiralpak IC) hexane/i-PrOH, 95:5, 1.0 mL/min].

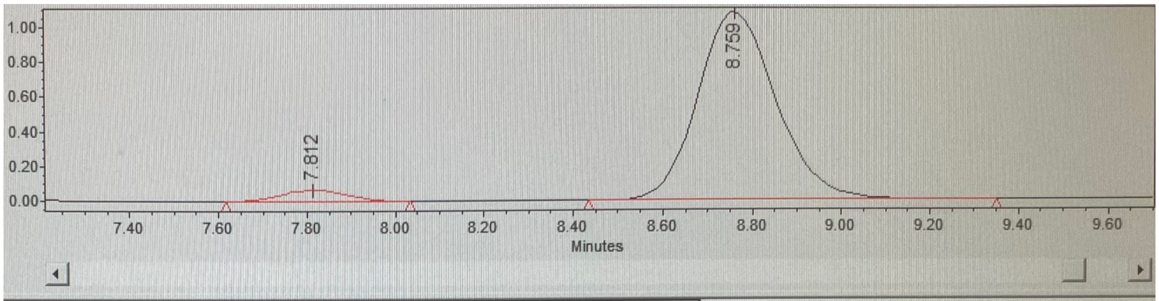

ne	Retention Time (min)	Area (µV*sec)	% Area	Height (µV)	Int Type	Amount	Units	Peak Type	Peak Codes
	7.812	689610	5.00	66775	bb			Unknown	
	8.759	13090518	95.00	1079194	bb			Unknown	

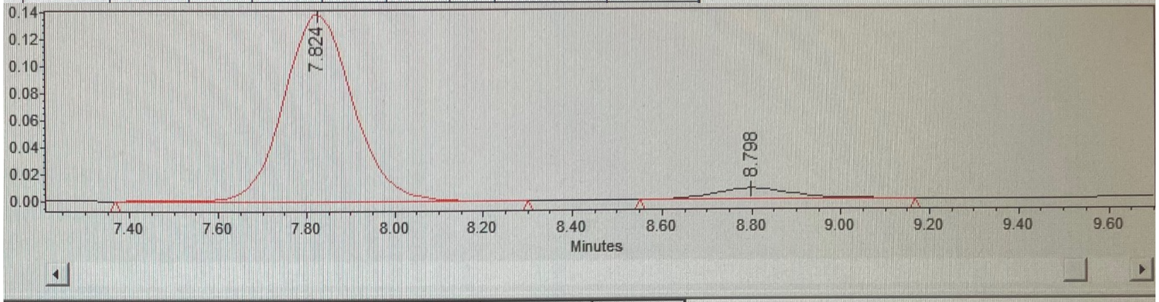

ne	Retention Time (min)	Area (µV*sec)	% Area	Height (µV)	Int Type	Amount	Units	Peak Type	Peak Codes
	7.824	1497923	94.00	137442	bb			Unknown	
	8.798	95639	6.00	7843	bb			Unknown	

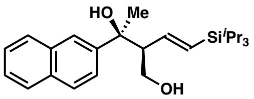

Enantiomeric excess was determined by HPLC analysis to be 94% ee (254 nm, 25 ˚C); t_1 = 6.30 min, t_2 = 9.48 min [(Chiralpak IC) hexane/i-PrOH, 90:10, 1.0 mL/min].

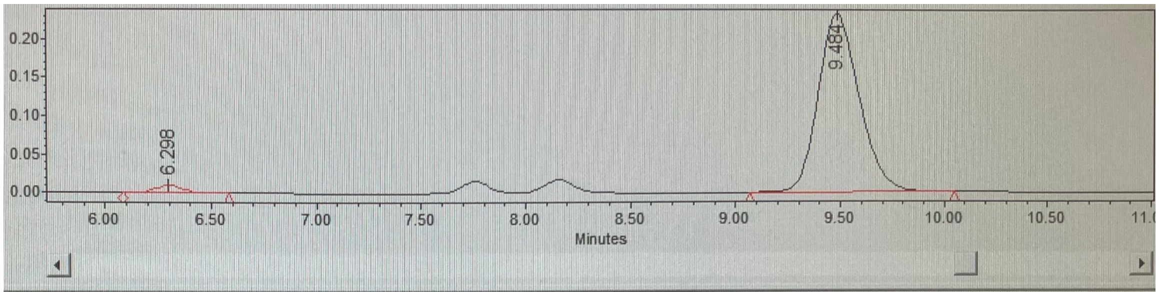

Retention Time (min)	Area (µV*sec)	% Area	Height (µV)	Int Type	Amount	Units	Peak Type	Peak Codes
6.298	91349	2.78	10113	VB			Unknown	
9.484	3198190	97.22	234847	BB			Unknown	

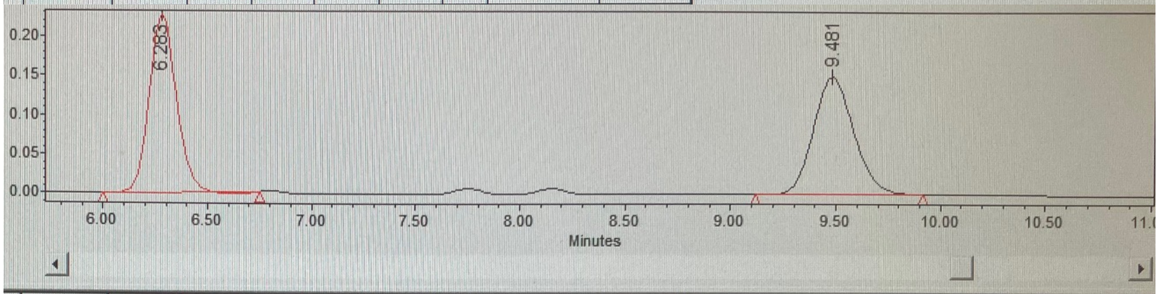

Retention Time (min)	Area (µV*sec)	% Area	Height (µV)	Int Type	Amount	Units	Peak Type	Peak Codes
6.283	2049207	50.33	226426	bb			Unknown	
9.481	2021979	49.67	150999	bb			Unknown	

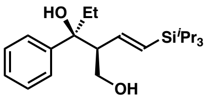

Enantiomeric excess was determined by HPLC analysis to be 90% ee (254 nm, 25 ˚C); t_1 = 5.20 min, t_2 = 5.49 min [(Chiralpak IC) hexane/i-PrOH, 95:5, 1.0 mL/min].

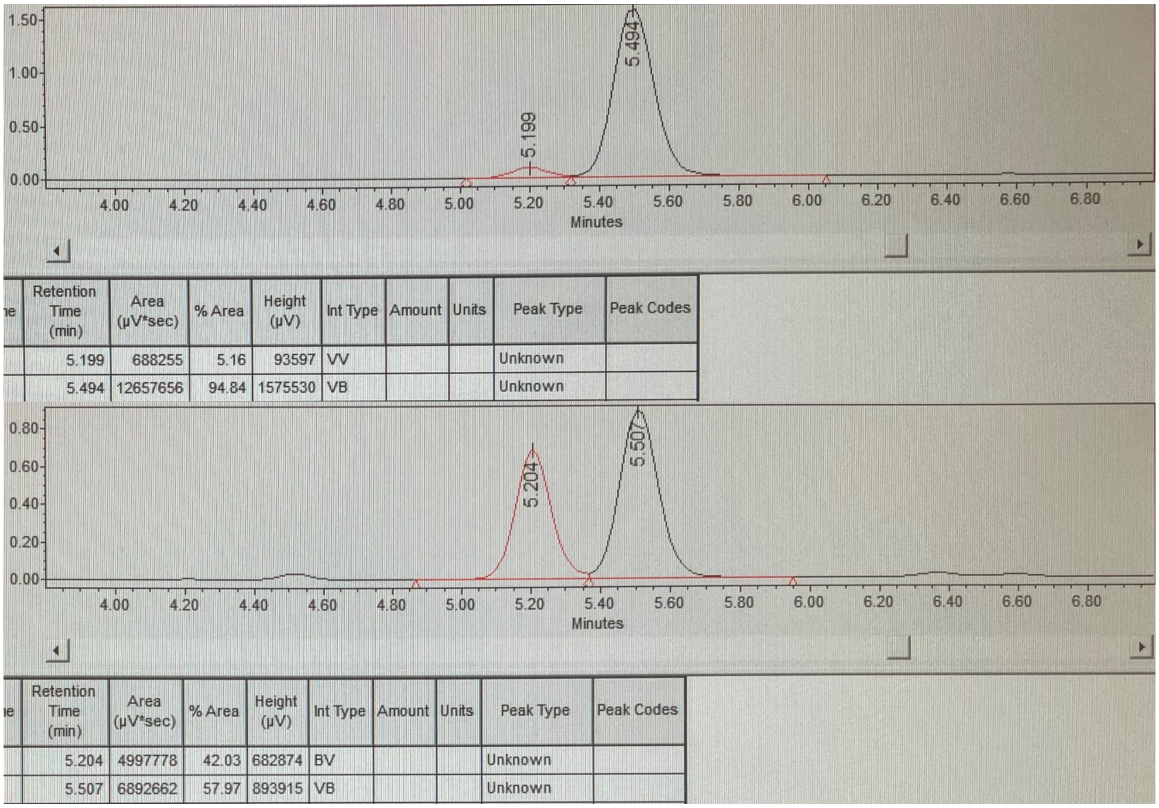

Retention Time (min)	Area (µV*sec)	% Area	Height (µV)	Int Type	Amount	Units	Peak Type	Peak Codes
5.199	688255	5.16	93597	VV			Unknown	
5.494	12657656	94.84	1575530	VB			Unknown	

Retention Time (min)	Area (µV*sec)	% Area	Height (µV)	Int Type	Amount	Units	Peak Type	Peak Codes
5.204	4997778	42.03	682874	BV			Unknown	
5.507	6892662	57.97	893915	VB			Unknown	

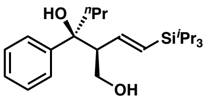

Enantiomeric excess was determined by HPLC analysis to be 85% ee (254 nm, 25 ˚C); t_1 = 5.05 min, t_2 = 5.38 min [(Chiralpak IC) hexane/i-PrOH, 95:5, 1.0 mL/min].

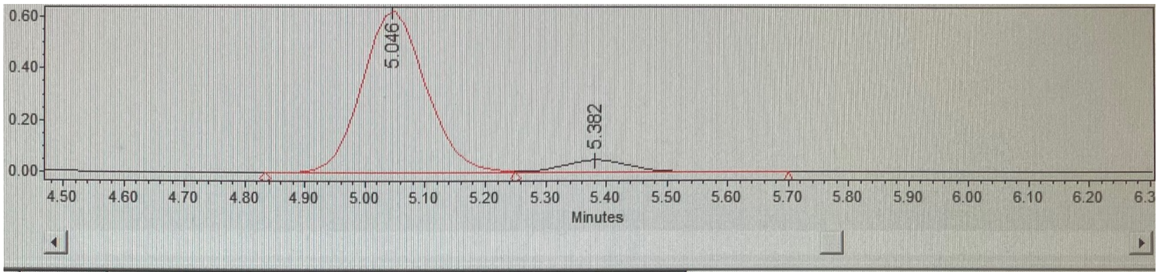

e	Retention Time (min)	Area (µV*sec)	% Area	Height (µV)	Int Type	Amount	Units	Peak Type	Peak Codes
	5.046	4607101	92.52	622744	VV			Unknown	
	5.382	372225	7.48	47371	VB			Unknown	

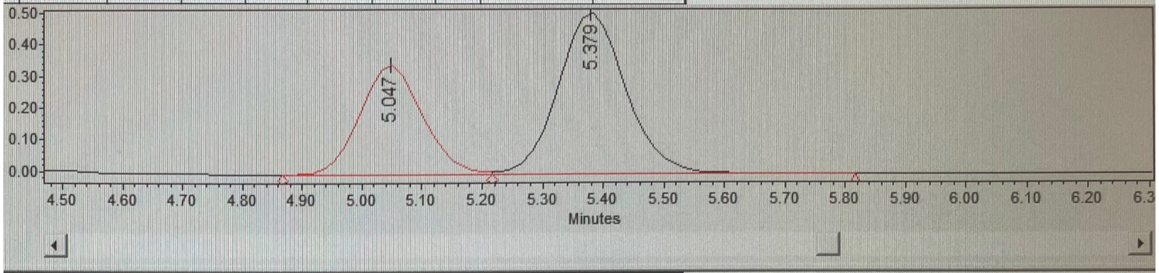

e	Retention Time (min)	Area (µV*sec)	% Area	Height (µV)	Int Type	Amount	Units	Peak Type	Peak Codes
	5.047	2519138	39.00	342045	VV			Unknown	
	5.379	3940433	61.00	505401	VB			Unknown	

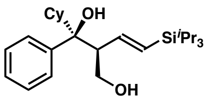

Enantiomeric excess was determined by HPLC analysis to be 96% ee (254 nm, 25 ˚C); t_1 = 4.41 min, t_2 = 4.95 min [(Chiralpak ID) hexane/i-PrOH, 95:5, 1.0 mL/min].

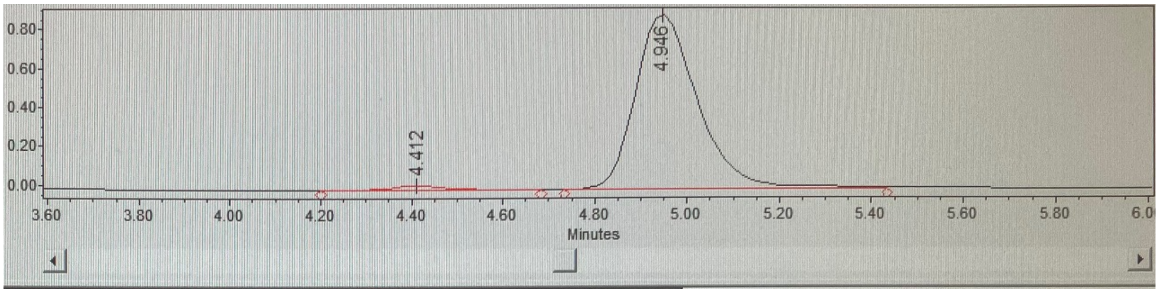

e	Retention Time (min)	Area (µV*sec)	% Area	Height (µV)	Int Type	Amount	Units	Peak Type	Peak Codes
	4.412	159038	1.88	20866	VV			Unknown	
	4.946	8281184	98.12	890158	VV			Unknown	

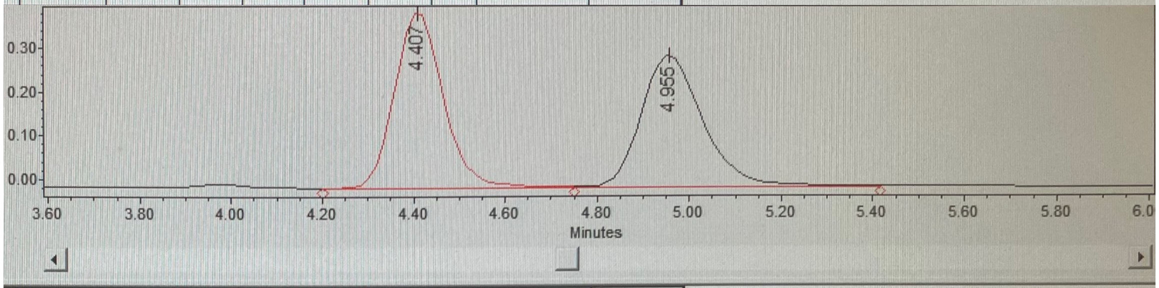

e	Retention Time (min)	Area (µV*sec)	% Area	Height (µV)	Int Type	Amount	Units	Peak Type	Peak Codes
	4.407	2957427	51.48	400598	VV			Unknown	
	4.955	2787714	48.52	301237	VV			Unknown	

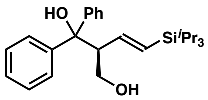

Enantiomeric excess was determined by HPLC analysis to be 97% ee (254 nm, 25 ˚C); t_1 = 8.00 min, t_2 = 10.2 min [(Chiralpak IC) hexane/i-PrOH, 95:5, 1.0 mL/min].

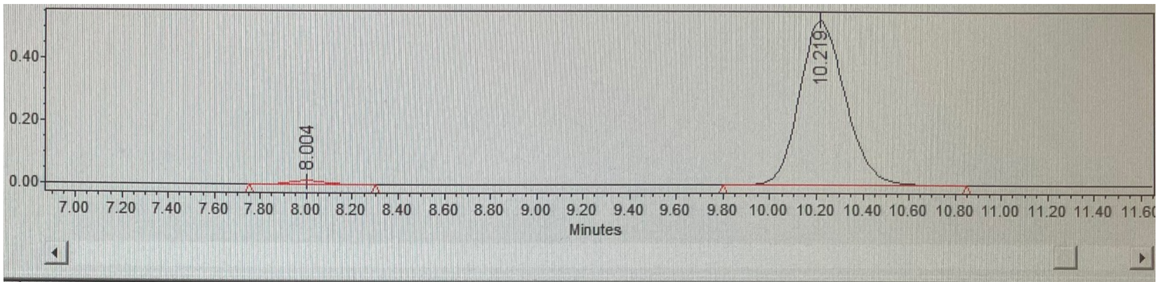

e	Retention Time (min)	Area (µV*sec)	% Area	Height (µV)	Int Type	Amount	Units	Peak Type	Peak Codes
	8.004	128812	1.75	12011	bb			Unknown	
	10.219	7246384	98.25	529622	BB			Unknown	

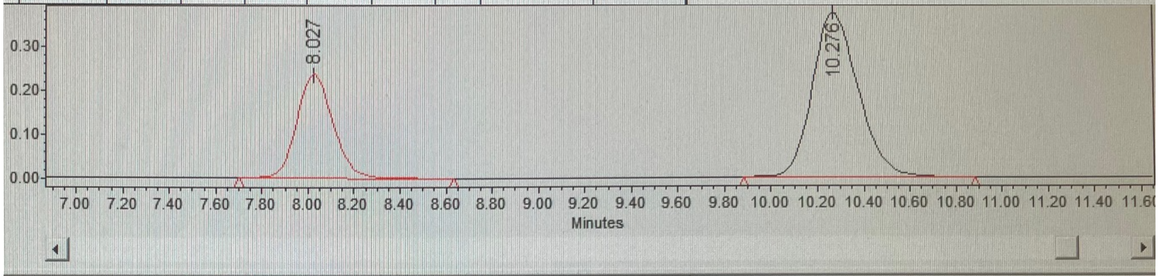

e	Retention Time (min)	Area (µV*sec)	% Area	Height (µV)	Int Type	Amount	Units	Peak Type	Peak Codes
	8.027	2549680	33.20	235309	bb			Unknown	
	10.276	5130364	66.80	374248	bb			Unknown	

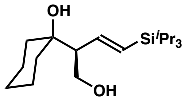

Enantiomeric excess was determined by HPLC analysis to be 96% ee (254 nm, 25 ˚C); t_1 = 7.45 min, t_2 = 9.08 min [(Chiralpak ID) hexane/i-PrOH, 95:5, 1.0 mL/min].

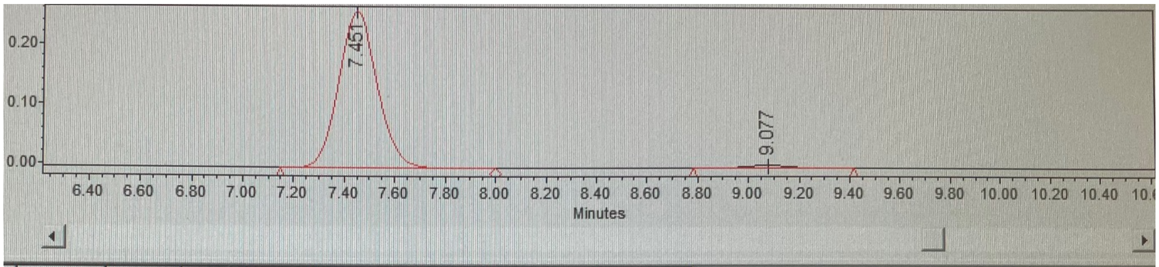

Retention Time (min)	Area (µV*sec)	% Area	Height (µV)	Int Type	Amount	Units	Peak Type	Peak Codes
7.451	2791879	97.79	260888	BV			Unknown	
9.077	63016	2.21	4958	BB			Unknown	

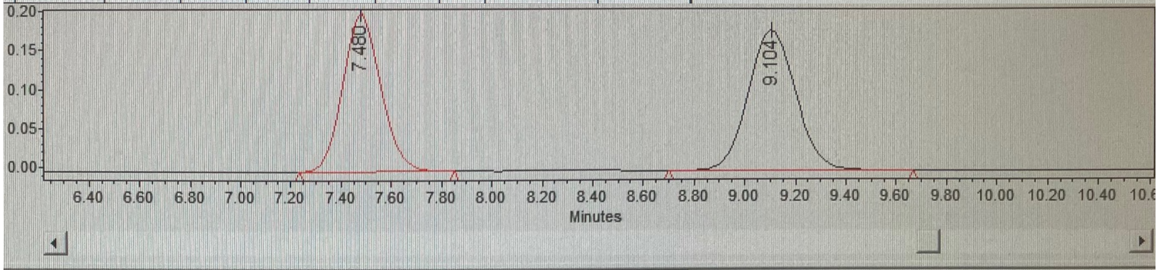

Retention Time (min)	Area (µV*sec)	% Area	Height (µV)	Int Type	Amount	Units	Peak Type	Peak Codes
7.480	2130325	48.04	199096	bb			Unknown	
9.104	2304429	51.96	178330	bb			Unknown	

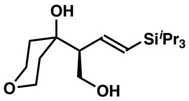

Enantiomeric excess was determined by HPLC analysis to be 99% ee (254 nm, 25 ˚C); t_1 = 11.0 min, t_2 = 12.4 min [(Chiralpak ID) hexane/i-PrOH, 95:5, 1.0 mL/min].

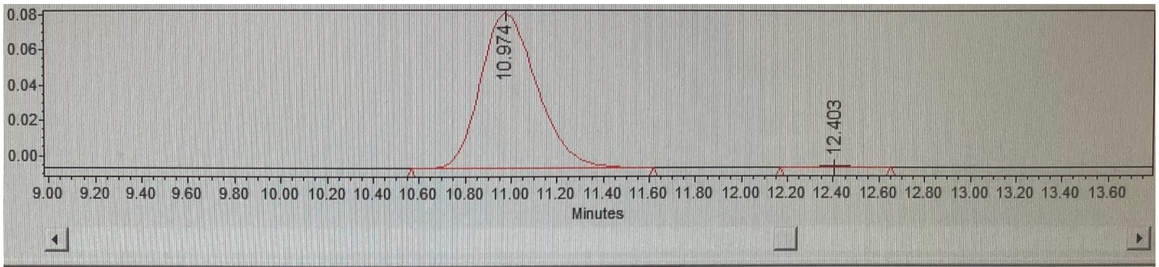

e	Retention Time (min)	Area (µV*sec)	% Area	Height (µV)	Int Type	Amount	Units	Peak Type	Peak Codes
	10.974	1489441	99.32	87493	BB			Unknown	
	12.403	10169	0.68	649	BB			Unknown	

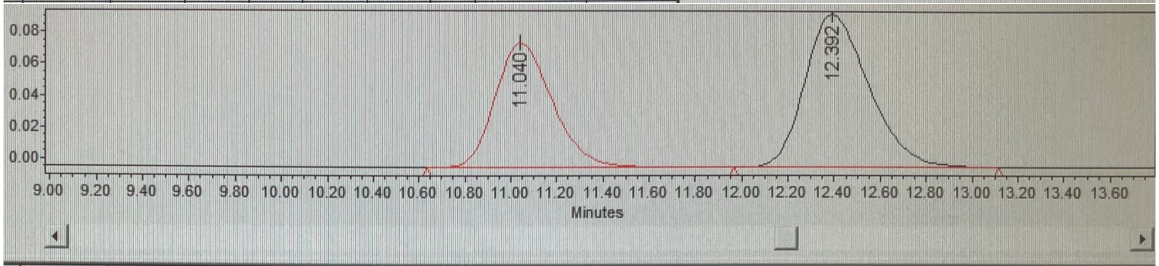

e	Retention Time (min)	Area (µV*sec)	% Area	Height (µV)	Int Type	Amount	Units	Peak Type	Peak Codes
	11.040	1357779	42.06	78702	BB			Unknown	
	12.392	1870606	57.94	96701	BB			Unknown	

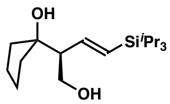

Enantiomeric excess was determined by HPLC analysis to be 98% ee (254 nm, 25 ˚C); t_1 = 7.92 min, t_2 = 9.54 min [(Chiralpak ID) hexane/i-PrOH, 95:5, 1.0 mL/min].

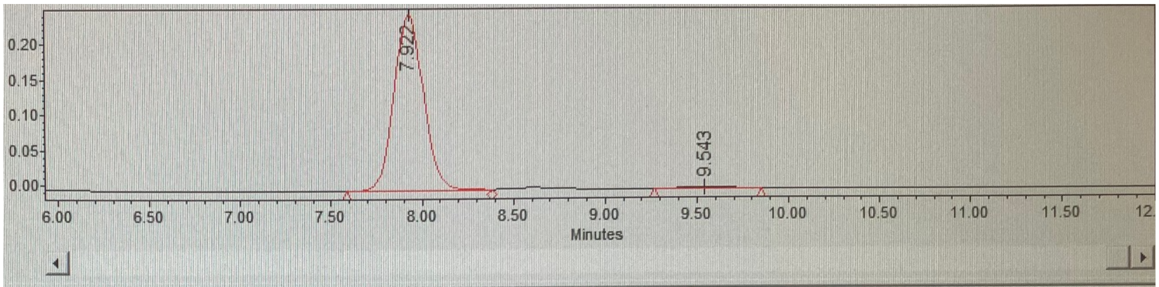

ne	Retention Time (min)	Area (µV*sec)	% Area	Height (µV)	Int Type	Amount	Units	Peak Type	Peak Codes
	7.922	2774070	99.09	249387	BV			Unknown	
	9.543	25409	0.91	1975	BB			Unknown	

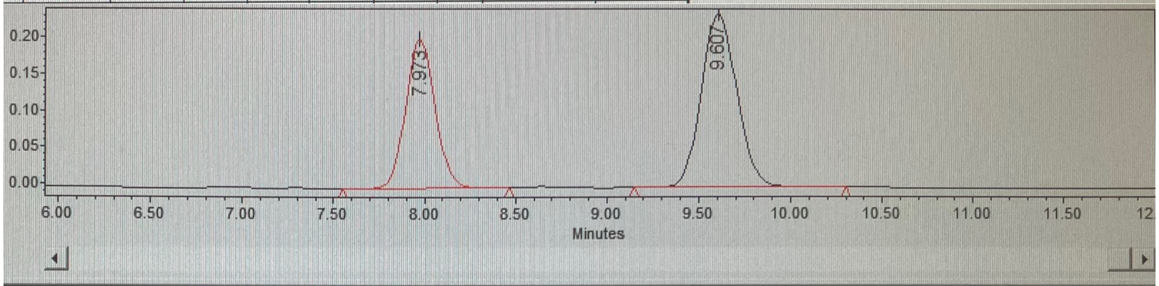

e	Retention Time (min)	Area (µV*sec)	% Area	Height (µV)	Int Type	Amount	Units	Peak Type	Peak Codes
	7.973	2269525	41.33	203357	bb			Unknown	
	9.607	3221293	58.67	238467	bb			Unknown	

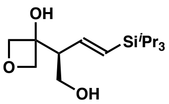

Enantiomeric excess was determined by HPLC analysis to be 96% ee (254 nm, 25 ˚C); t_1 = 6.49 min, t_2 = 7.12 min [(Chiralpak IC) hexane/i-PrOH, 95:5, 1.0 mL/min].

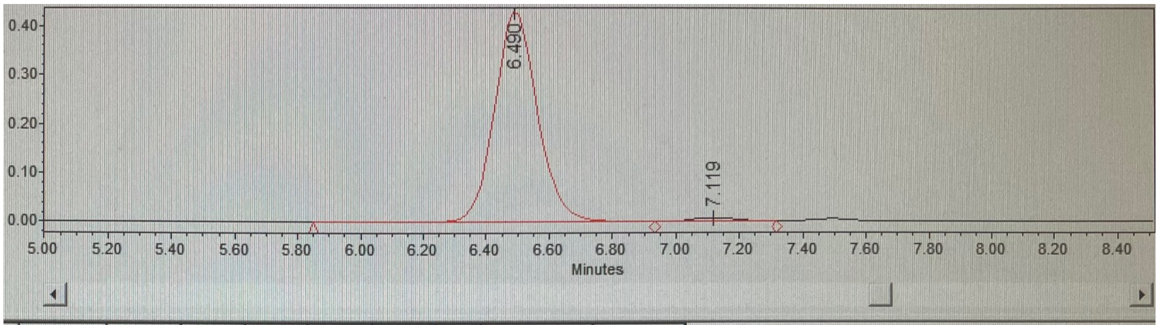

e	Retention Time (min)	Area (µV*sec)	% Area	Height (µV)	Int Type	Amount	Units	Peak Type	Peak Codes
	6.490	4071924	97.94	429299	BV			Unknown	
	7.119	85726	2.06	8256	VV			Unknown	

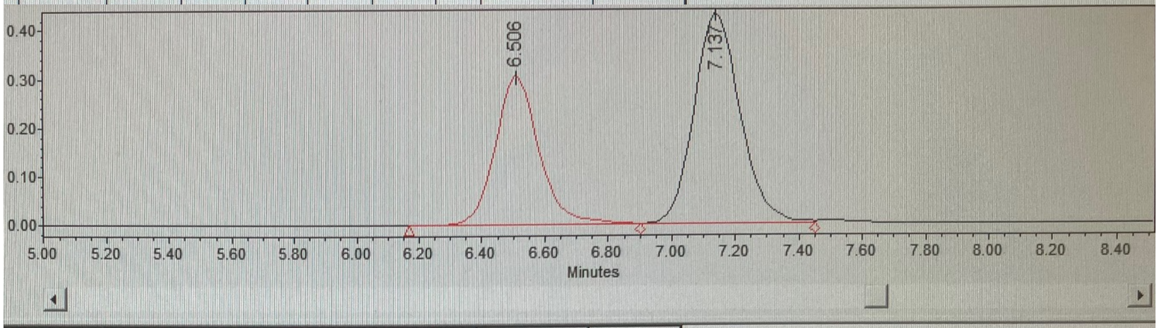

e	Retention Time (min)	Area (µV*sec)	% Area	Height (µV)	Int Type	Amount	Units	Peak Type	Peak Codes
	6.506	2948794	40.46	303611	BV			Unknown	
	7.137	4339785	59.54	429227	VV			Unknown	

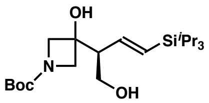

Enantiomeric excess was determined by HPLC analysis to be 94% ee (254 nm, 25 ˚C); t_1 = 8.38 min, t_2 = 9.77 min [(Chiralpak IG) hexane/i-PrOH, 95:5, 1.0 mL/min].

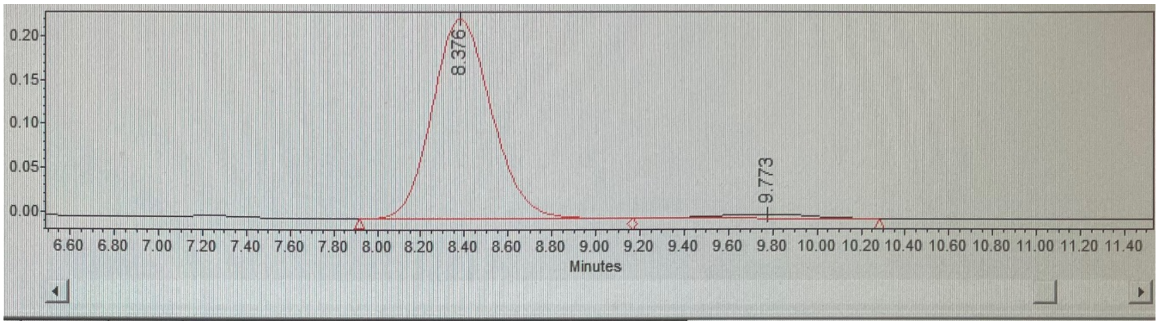

ne	Retention Time (min)	Area (µV*sec)	% Area	Height (µV)	Int Type	Amount	Units	Peak Type	Peak Codes
	8.376	4307497	96.86	227976	BV			Unknown	
	9.773	139596	3.14	4804	VB			Unknown	

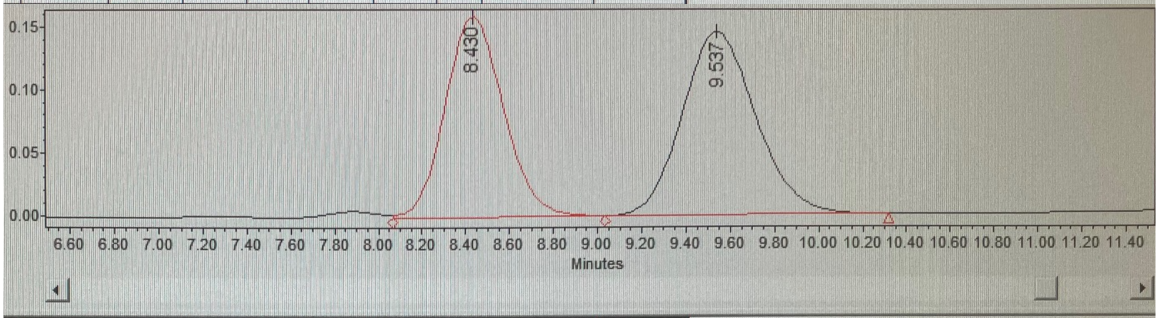

ne	Retention Time (min)	Area (µV*sec)	% Area	Height (µV)	Int Type	Amount	Units	Peak Type	Peak Codes
	8.430	2923544	46.49	158633	VV			Unknown	
	9.537	3364428	53.51	145428	VB			Unknown	

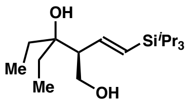

Enantiomeric excess was determined by HPLC analysis to be 92% ee (254 nm, 25 ˚C); t_1 = 7.09 min, t_2 = 10.0 min [(Chiralpak IG) hexane/i-PrOH, 95:5, 1.0 mL/min].

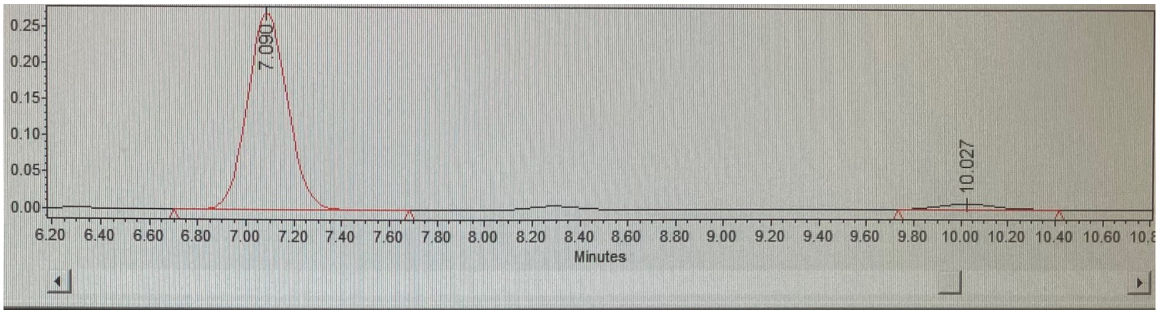

ne	Retention Time (min)	Area (µV*sec)	% Area	Height (µV)	Int Type	Amount	Units	Peak Type	Peak Codes
	7.090	3123865	96.04	269404	bb			Unknown	
	10.027	128866	3.96	7509	bb			Unknown	

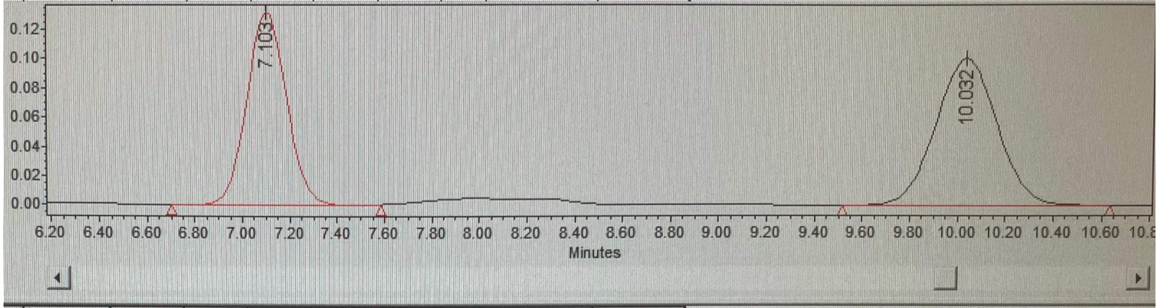

ne	Retention Time (min)	Area (µV*sec)	% Area	Height (µV)	Int Type	Amount	Units	Peak Type	Peak Codes
	7.103	1516365	45.92	131201	bb			Unknown	
	10.032	1785589	54.08	100318	bb			Unknown	

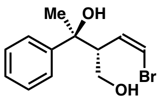

Enantiomeric excess was determined by HPLC analysis to be 98% ee (254 nm, 25 ˚C); t_1 = 8.10 min, t_2 = 9.37 min [(Chiralpak IA) hexane/i-PrOH, 90:10, 1.0 mL/min];

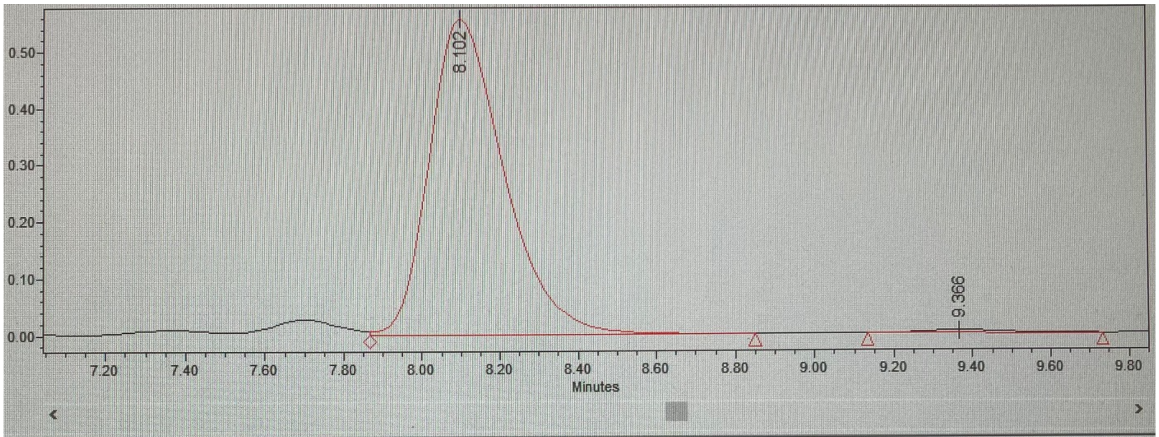

e	Retention Time (min)	Area (µV*sec)	% Area	Height (µV)	Int Type	Amount	Units	Peak Type	Peak Codes
	8.102	7437488	99.10	558116	VB			Unknown	
	9.366	67384	0.90	4882	BB			Unknown	

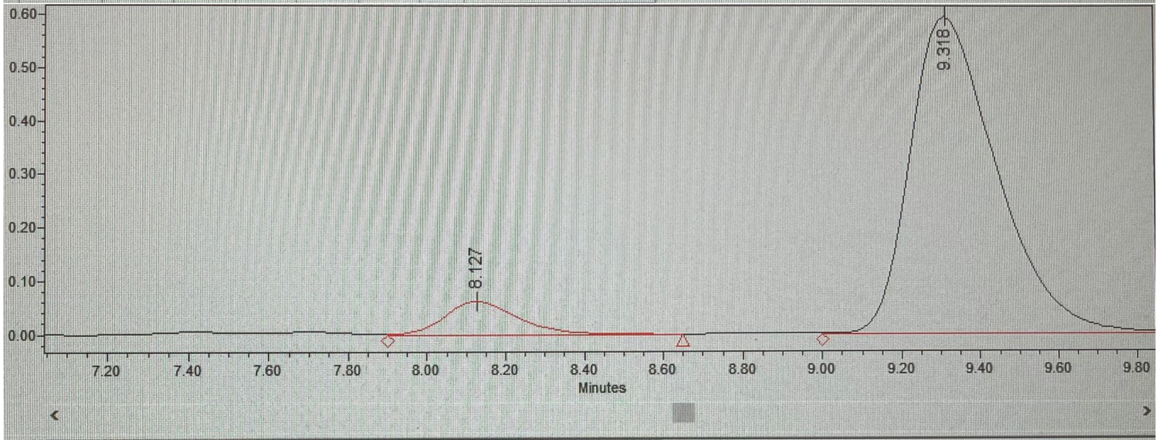

e	Retention Time (min)	Area (µV*sec)	% Area	Height (µV)	Int Type	Amount	Units	Peak Type	Peak Codes
	8.127	810753	8.16	63752	VB			Unknown	
	9.318	9126200	91.84	596879	VV			Unknown	

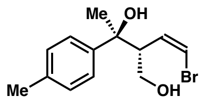

Enantiomeric excess was determined by HPLC analysis to be 98% ee (254 nm, 25 ˚C); t_1 = 7.45 min, t_2 = 9.75 min [(Chiralpak IA) hexane/i-PrOH, 90: 10, 1.0 mL/min].

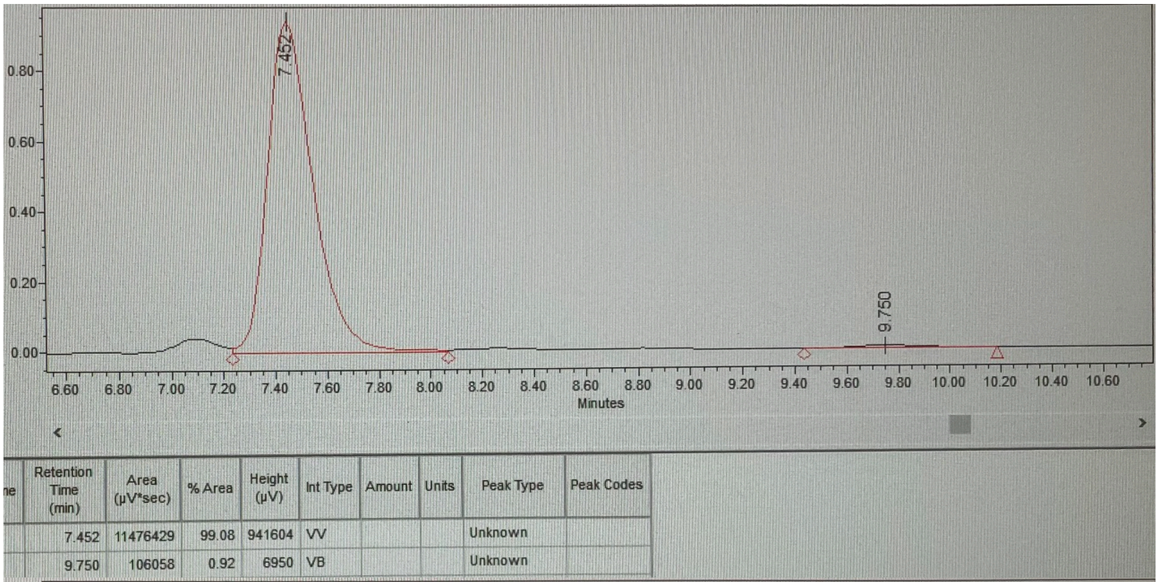

Retention Time (min)	Area (μV*sec)	% Area	Height (μV)	Int Type	Amount	Units	Peak Type	Peak Codes
7.452	11476429	99.08	941604	VV			Unknown	
9.750	106058	0.92	6950	VB			Unknown	

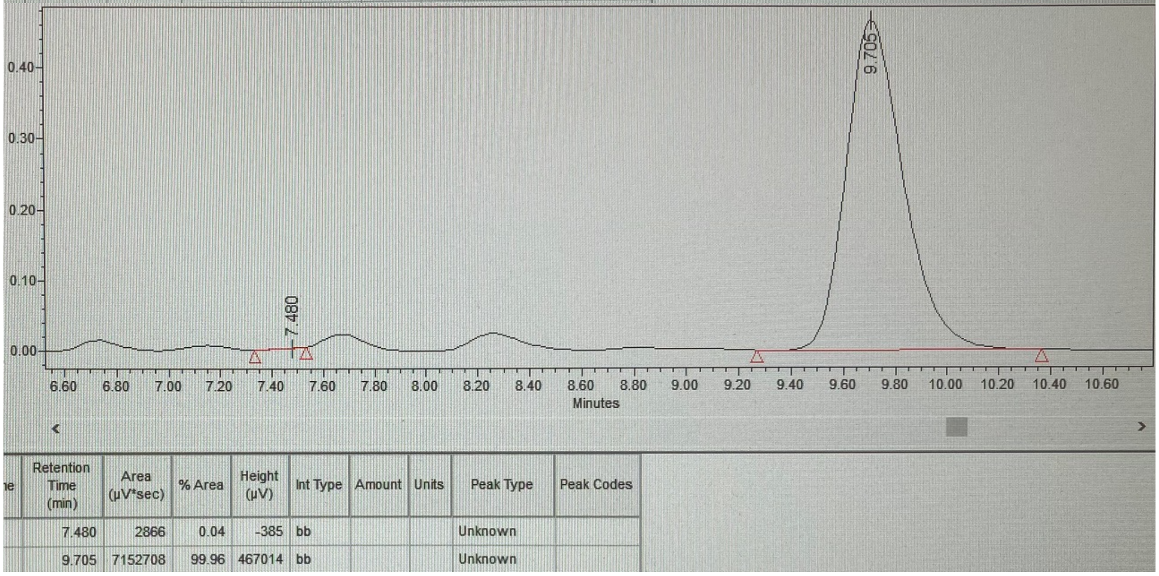

Retention Time (min)	Area (μV*sec)	% Area	Height (μV)	Int Type	Amount	Units	Peak Type	Peak Codes
7.480	2866	0.04	-385	bb			Unknown	
9.705	7152708	99.96	467014	bb			Unknown	

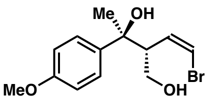

Enantiomeric excess was determined by HPLC analysis to be 96% ee (254 nm, 25 ˚C); t_1 = 9.85 min, t_2 = 10.9 min [(Chiralpak ID) hexane/i-PrOH, 90: 10, 1.0 mL/min].

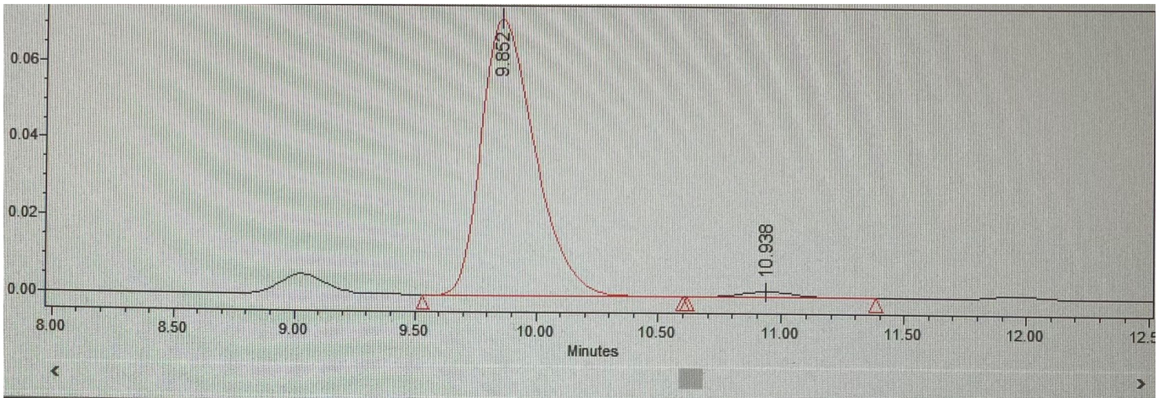

Retention Time (min)	Area (μV*sec)	% Area	Height (μV)	Int Type	Amount	Units	Peak Type	Peak Codes
9.852	1097306	97.86	71871	bb			Unknown	
10.938	23983	2.14	1531	bb			Unknown	

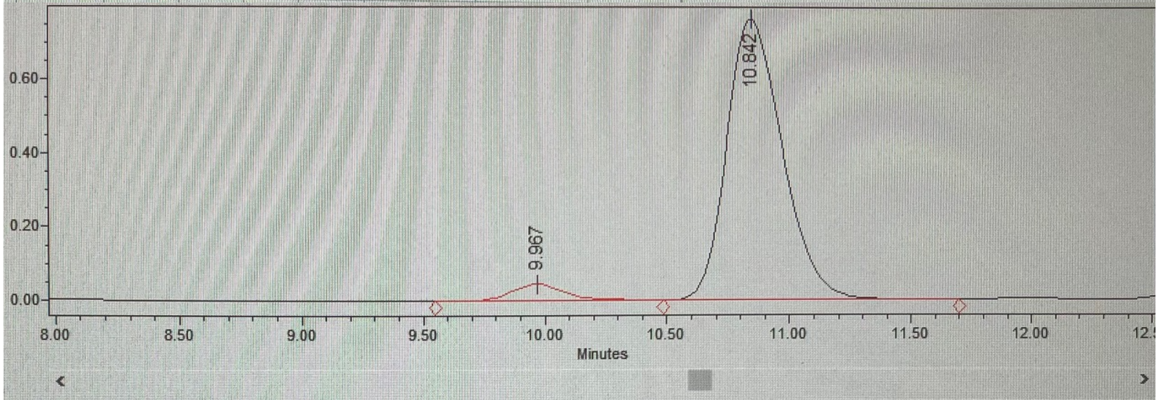

Retention Time (min)	Area (μV*sec)	% Area	Height (μV)	Int Type	Amount	Units	Peak Type	Peak Codes
9.967	773291	5.95	47062	VV			Unknown	
10.842	12212608	94.05	766831	VV			Unknown	

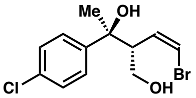

Enantiomeric excess was determined by HPLC analysis to be 98% ee (254 nm, 25 ˚C); t_1 = 8.31 min, t_2 = 12.1 min [(Chiralpak IA) hexane/i-PrOH, 90: 10, 1.0 mL/min].

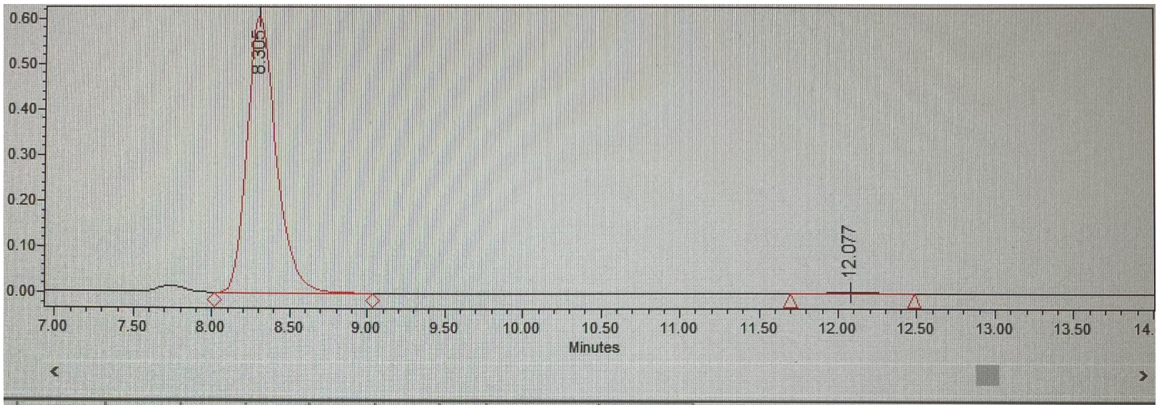

Retention Time (min)	Area (µV*sec)	% Area	Height (µV)	Int Type	Amount	Units	Peak Type	Peak Codes
8.305	7942142	99.08	605916	VV			Unknown	
12.077	73836	0.92	4060	BB			Unknown	

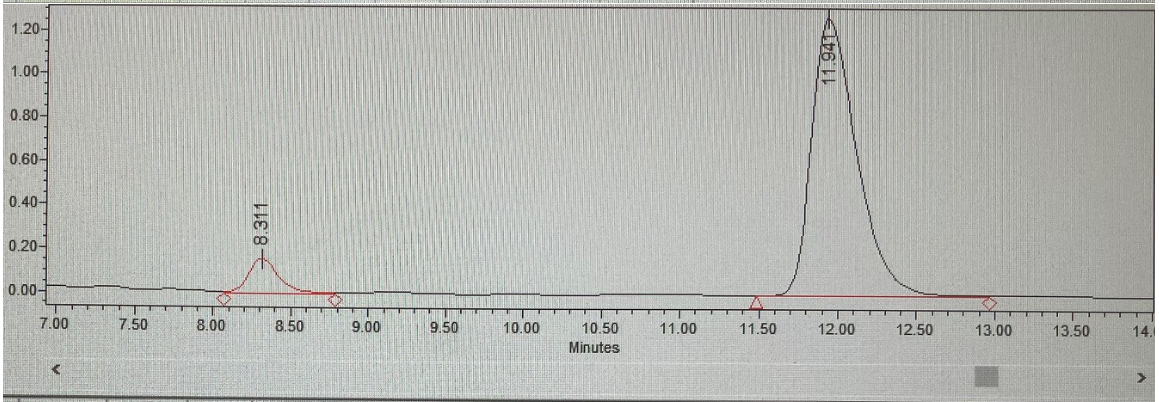

Retention Time (min)	Area (µV*sec)	% Area	Height (µV)	Int Type	Amount	Units	Peak Type	Peak Codes
8.311	2107793	7.74	157595	VV			Unknown	
11.941	25119808	92.26	1269455	BV			Unknown	

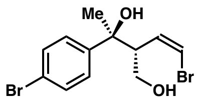

Enantiomeric excess was determined by HPLC analysis to be 98% ee (254 nm, 25 ˚C); t_1 = 10.4 min, t_2 = 13.1 min [(Chiralpak ID) hexane/i-PrOH, 95: 5, 1.0 mL/min].

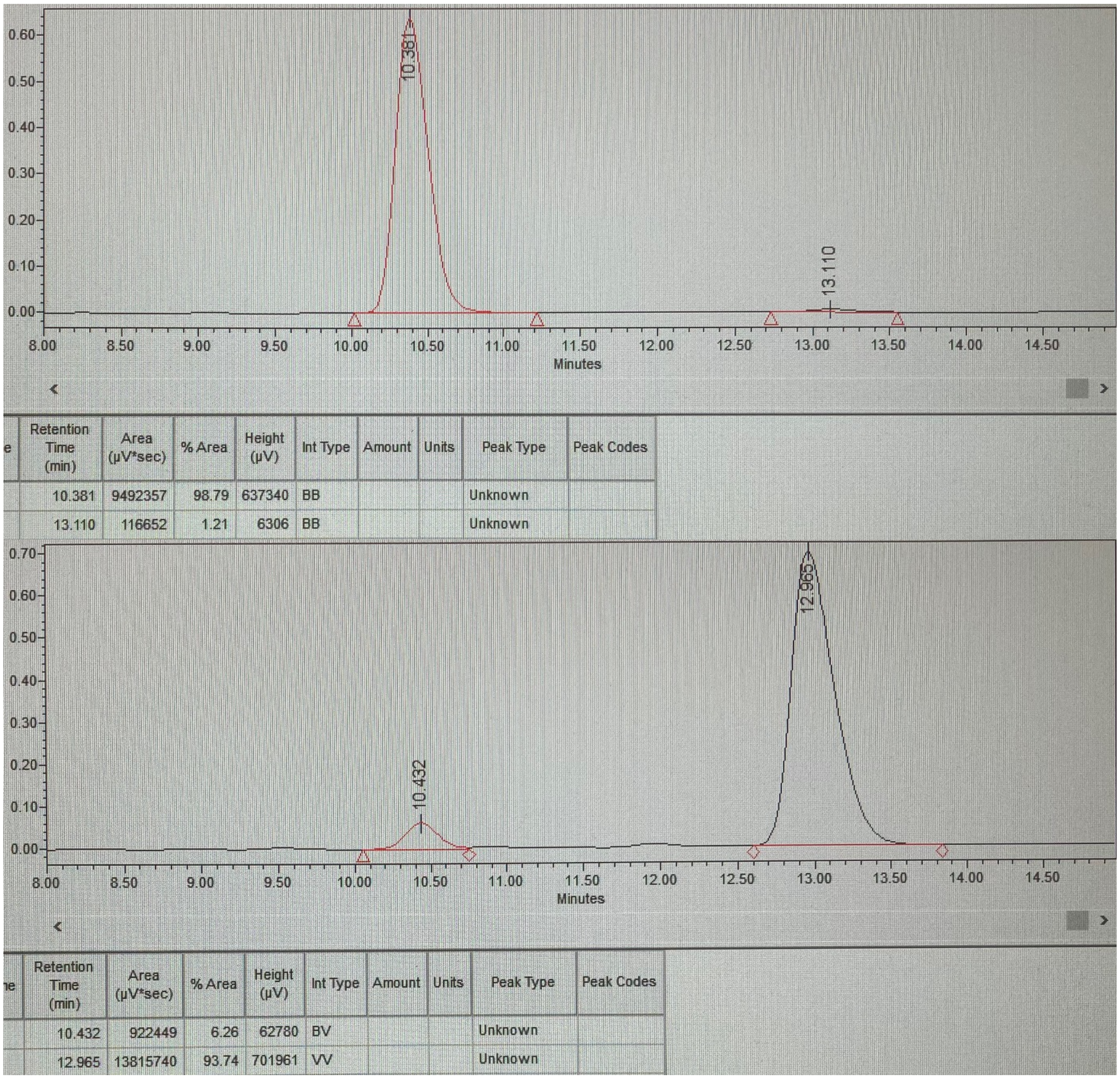

Retention Time (min)	Area (μV*sec)	% Area	Height (μV)	Int Type	Amount	Units	Peak Type	Peak Codes
10.381	9492357	98.79	637340	BB			Unknown	
13.110	116652	1.21	6306	BB			Unknown	

Retention Time (min)	Area (μV*sec)	% Area	Height (μV)	Int Type	Amount	Units	Peak Type	Peak Codes
10.432	922449	6.26	62780	BV			Unknown	
12.965	13815740	93.74	701961	VV			Unknown	

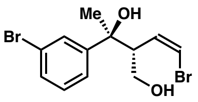

Enantiomeric excess was determined by HPLC analysis to be 98% ee (254 nm, 25 ˚C); t_1 = 12.0 min, t_2 = 15.6 min [(Chiralpak IA) hexane/i-PrOH, 95: 5, 1.0 mL/min].

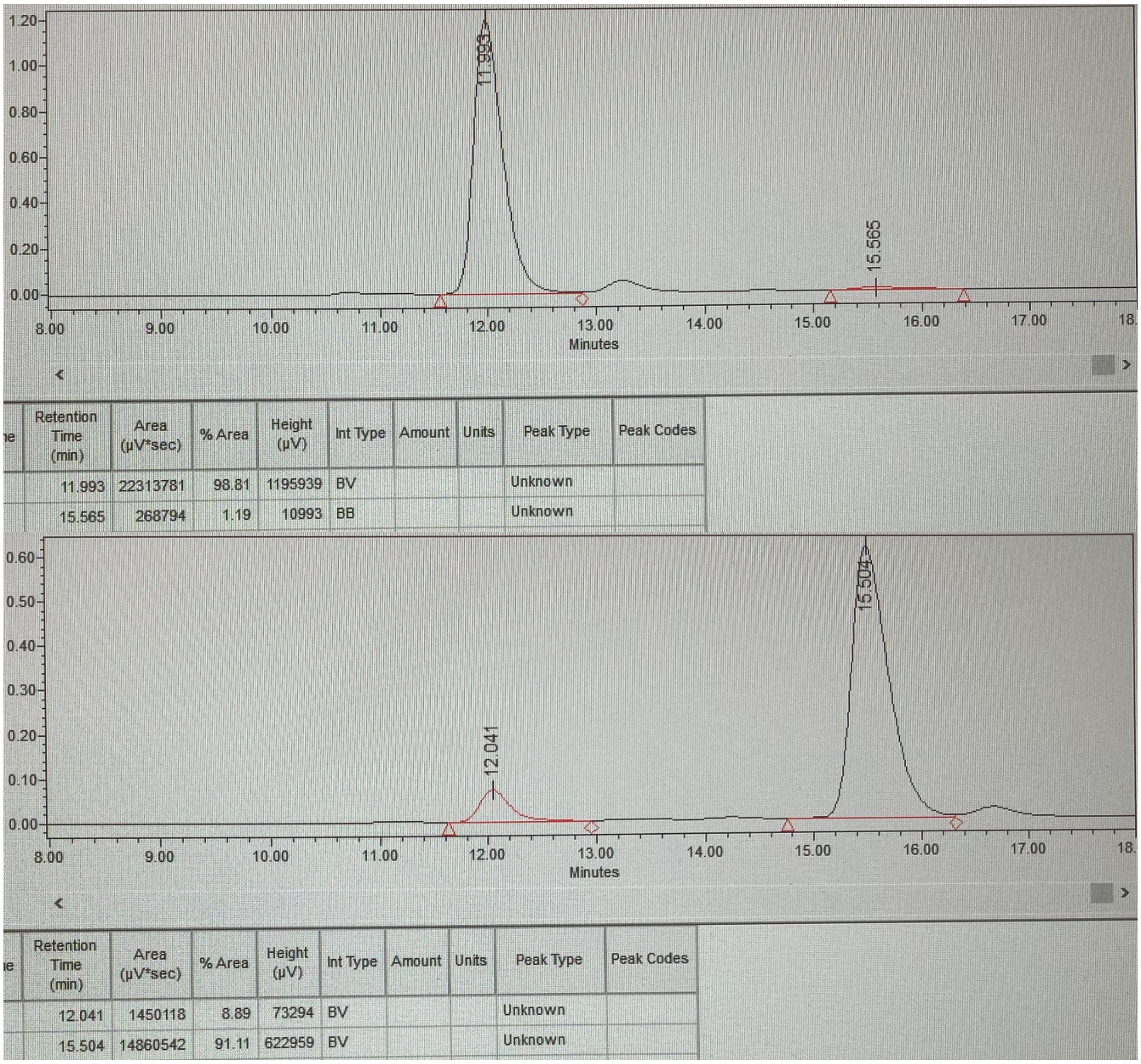

ne	Retention Time (min)	Area (µV*sec)	% Area	Height (µV)	Int Type	Amount	Units	Peak Type	Peak Codes
	11.993	22313781	98.81	1195939	BV			Unknown	
	15.565	268794	1.19	10993	BB			Unknown	

ne	Retention Time (min)	Area (µV*sec)	% Area	Height (µV)	Int Type	Amount	Units	Peak Type	Peak Codes
	12.041	1450118	8.89	73294	BV			Unknown	
	15.504	14860542	91.11	622959	BV			Unknown	

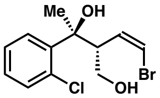

Enantiomeric excess was determined by HPLC analysis to be 99% ee (254 nm, 25 ˚C); t_1 = 11.0 min, t_2 = 14.2 min [(Chiralpak IA) hexane/i-PrOH, 95: 5, 1.0 mL/min].

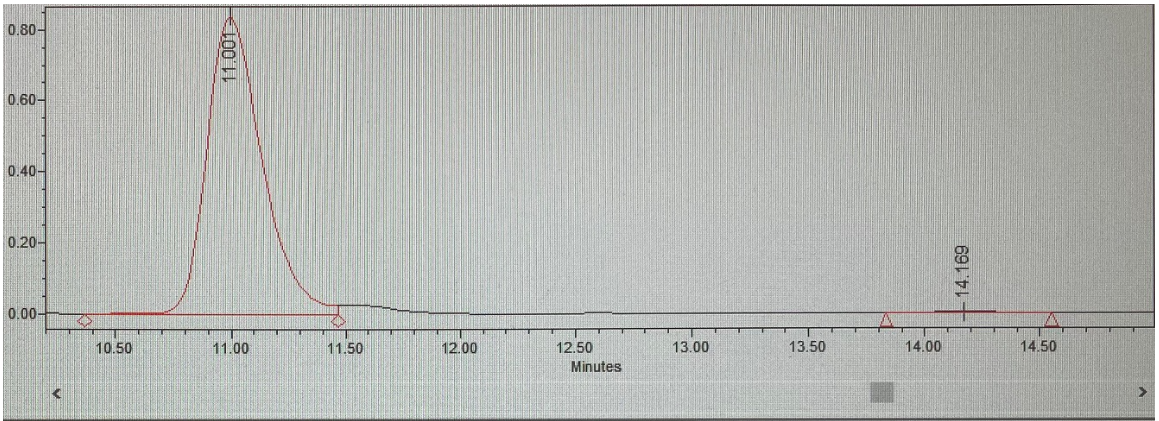

e	Retention Time (min)	Area (µV*sec)	% Area	Height (µV)	Int Type	Amount	Units	Peak Type	Peak Codes
	11.001	13969935	99.64	837502	VV			Unknown	
	14.169	50566	0.36	2748	BB			Unknown	

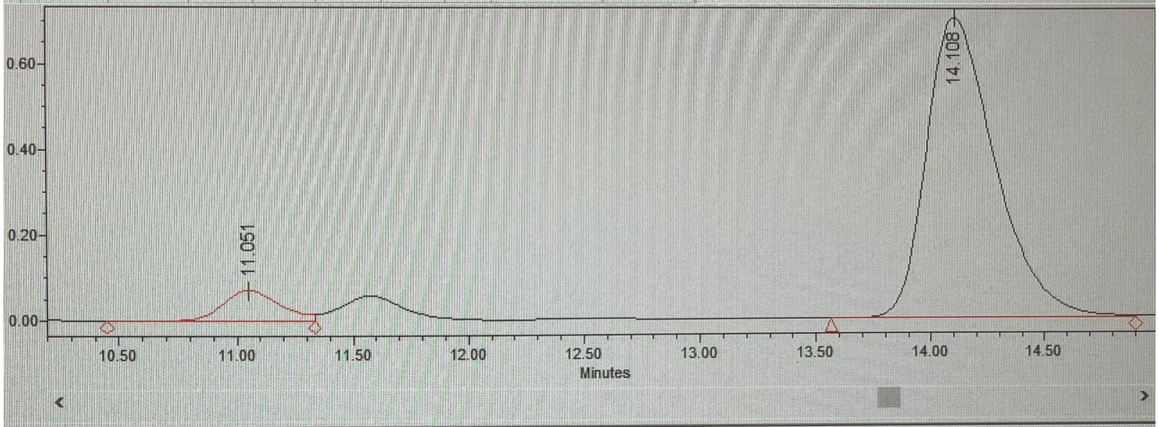

ie	Retention Time (min)	Area (µV*sec)	% Area	Height (µV)	Int Type	Amount	Units	Peak Type	Peak Codes
	11.051	1201823	7.45	72274	VV			Unknown	
	14.108	14925295	92.55	711715	BV			Unknown	

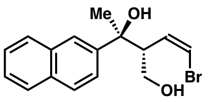

Enantiomeric excess was determined by HPLC analysis to be 98% ee (254 nm, 25 ˚C); t_1 = 8.89 min, t_2 = 12.3 min [(Chiralpak IA) hexane/i-PrOH, 90:10, 1.0 mL/min]

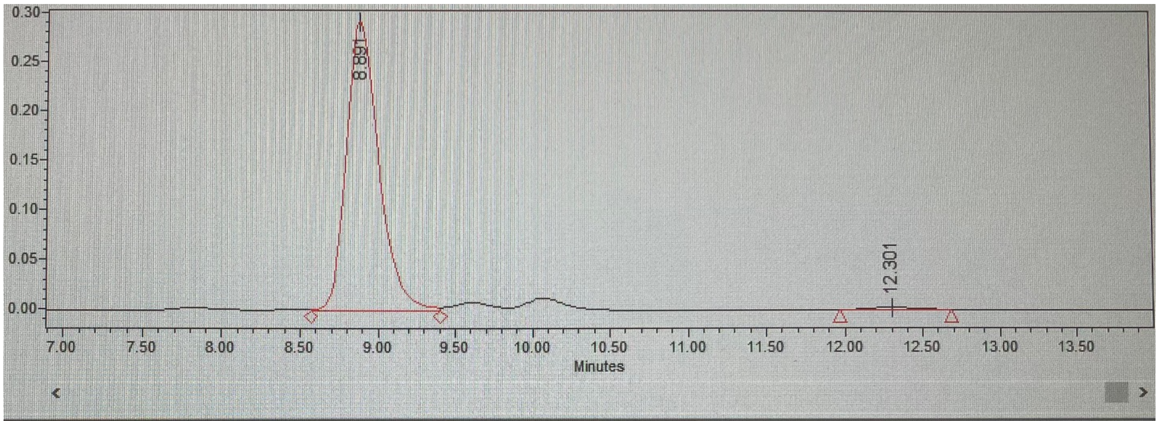

e	Retention Time (min)	Area (µV*sec)	% Area	Height (µV)	Int Type	Amount	Units	Peak Type	Peak Codes
	8.891	4240844	99.08	292198	VV			Unknown	
	12.301	39225	0.92	2137	BB			Unknown	

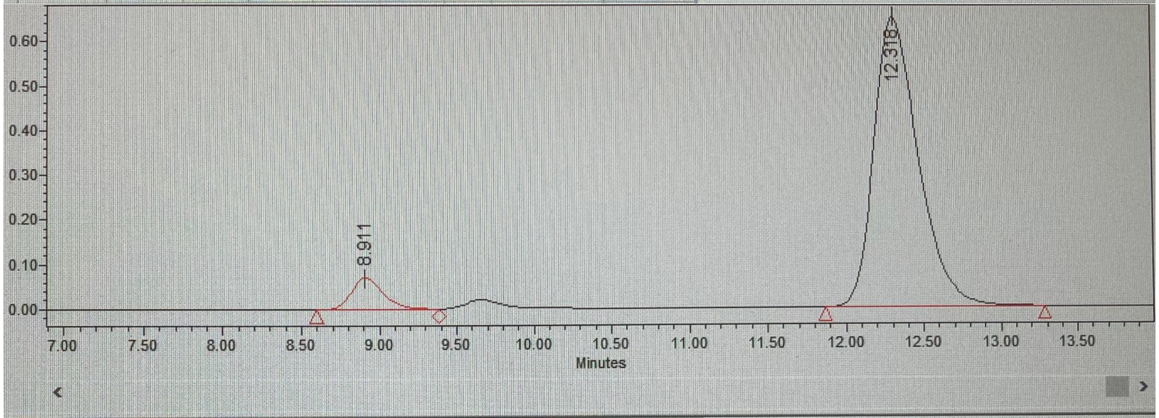

e	Retention Time (min)	Area (µV*sec)	% Area	Height (µV)	Int Type	Amount	Units	Peak Type	Peak Codes
	8.911	1026460	7.32	70739	BV			Unknown	
	12.318	12999348	92.68	654344	BB			Unknown	

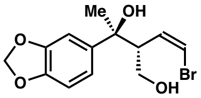

Enantiomeric excess was determined by HPLC analysis to be 99% ee (254 nm, 25 ˚C); t_1 = 13.3 min, t_2 = 14.4 min [(Chiralpak IA) hexane/i-PrOH, 90: 10, 1.0 mL/min].

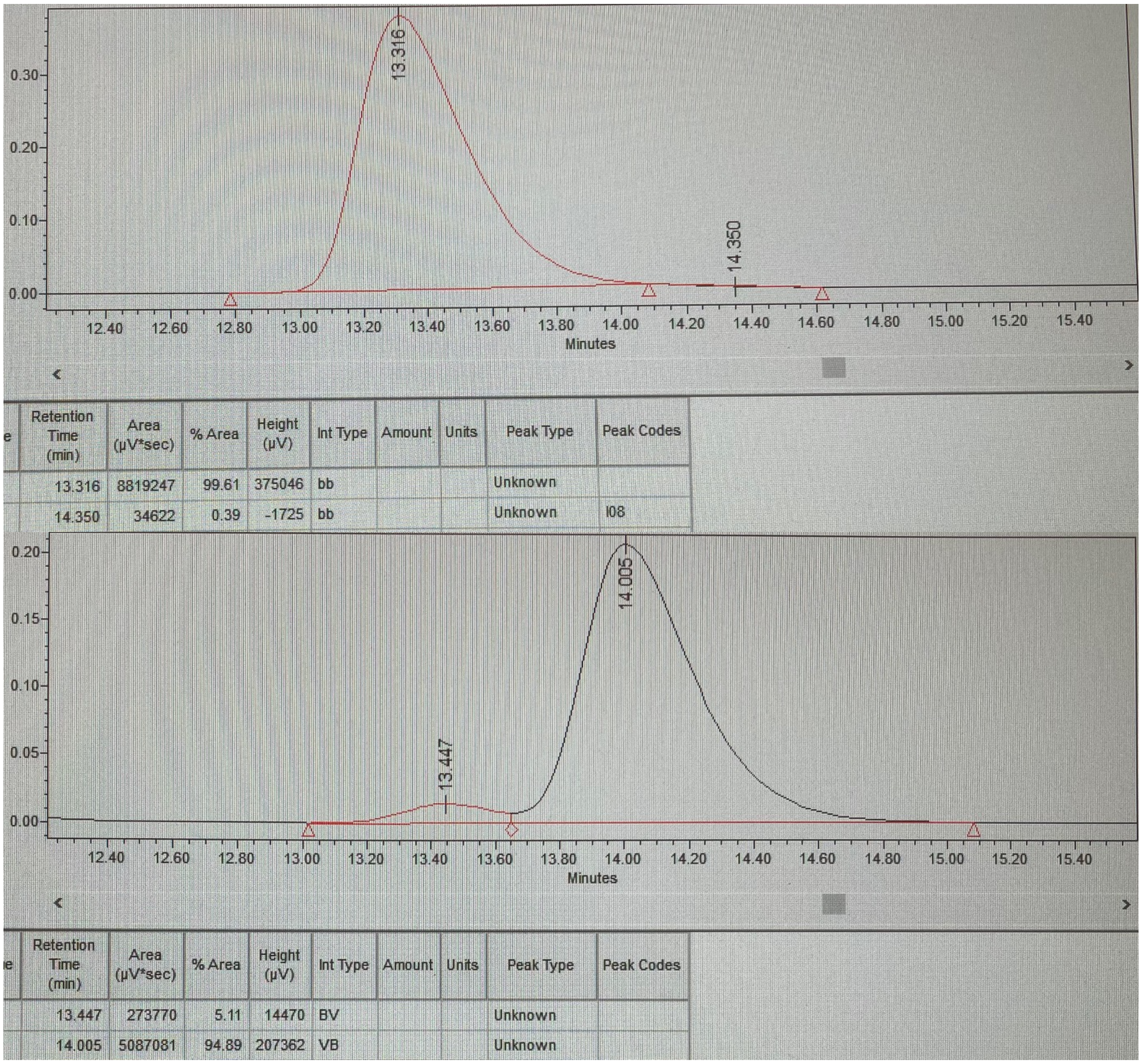

Retention Time (min)	Area (µV*sec)	% Area	Height (µV)	Int Type	Amount	Units	Peak Type	Peak Codes
13.316	8819247	99.61	375046	bb			Unknown	
14.350	34622	0.39	-1725	bb			Unknown	I08

Retention Time (min)	Area (µV*sec)	% Area	Height (µV)	Int Type	Amount	Units	Peak Type	Peak Codes
13.447	273770	5.11	14470	BV			Unknown	
14.005	5087081	94.89	207362	VB			Unknown	

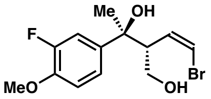

Enantiomeric excess was determined by HPLC analysis to be 98% ee (254 nm, 25 ˚C); t_1 = 20.1 min, t_2 = 21.7 min [(Chiralpak ID) hexane/i-PrOH, 95: 5, 1.0 mL/min].

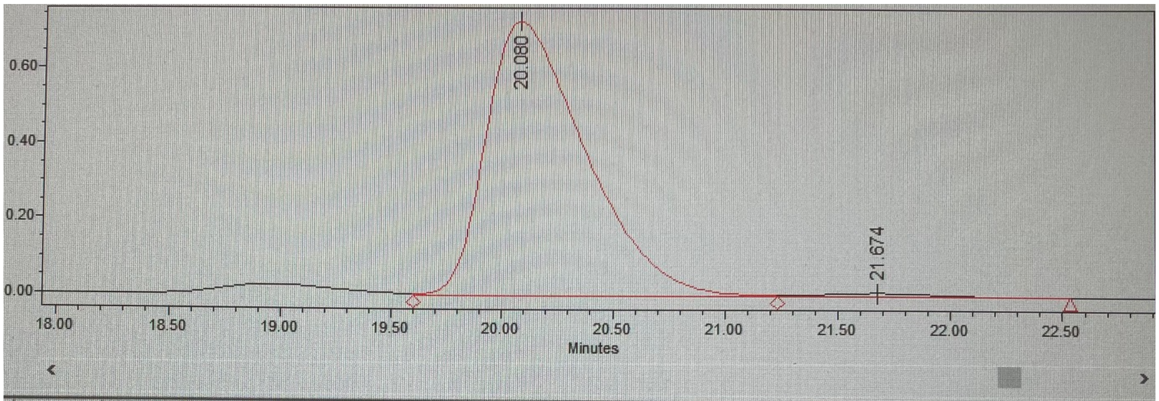

Retention Time (min)	Area (µV*sec)	% Area	Height (µV)	Int Type	Amount	Units	Peak Type	Peak Codes
20.080	22656465	98.72	726532	VV			Unknown	
21.674	294681	1.28	8933	VB			Unknown	

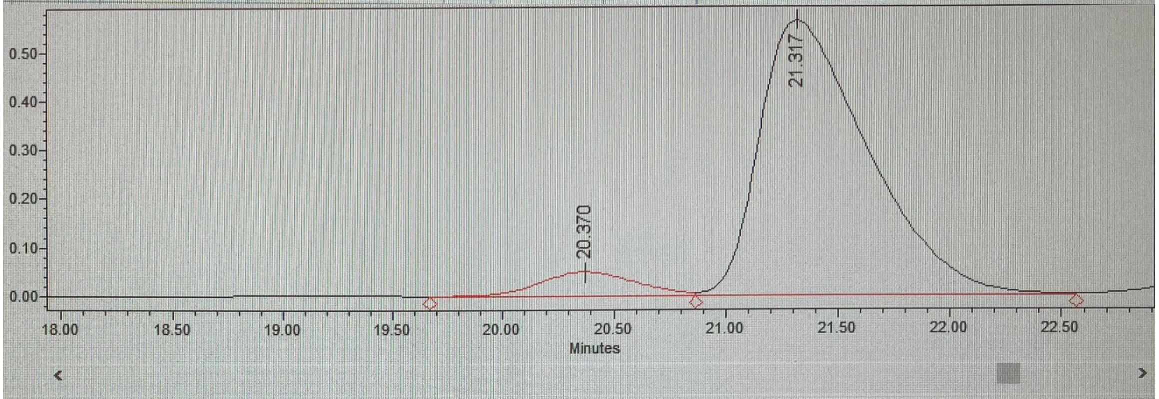

Retention Time (min)	Area (µV*sec)	% Area	Height (µV)	Int Type	Amount	Units	Peak Type	Peak Codes
20.370	1491081	7.32	49657	VV			Unknown	
21.317	18888646	92.68	561551	VV			Unknown	

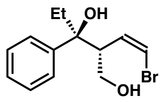

Enantiomeric excess was determined by HPLC analysis to be 96% ee (254 nm, 25 ˚C); t_1 = 12.8 min, t_2 = 15.1 min [(Chiralpak IA) hexane/i-PrOH, 95: 5, 1.0 mL/min].

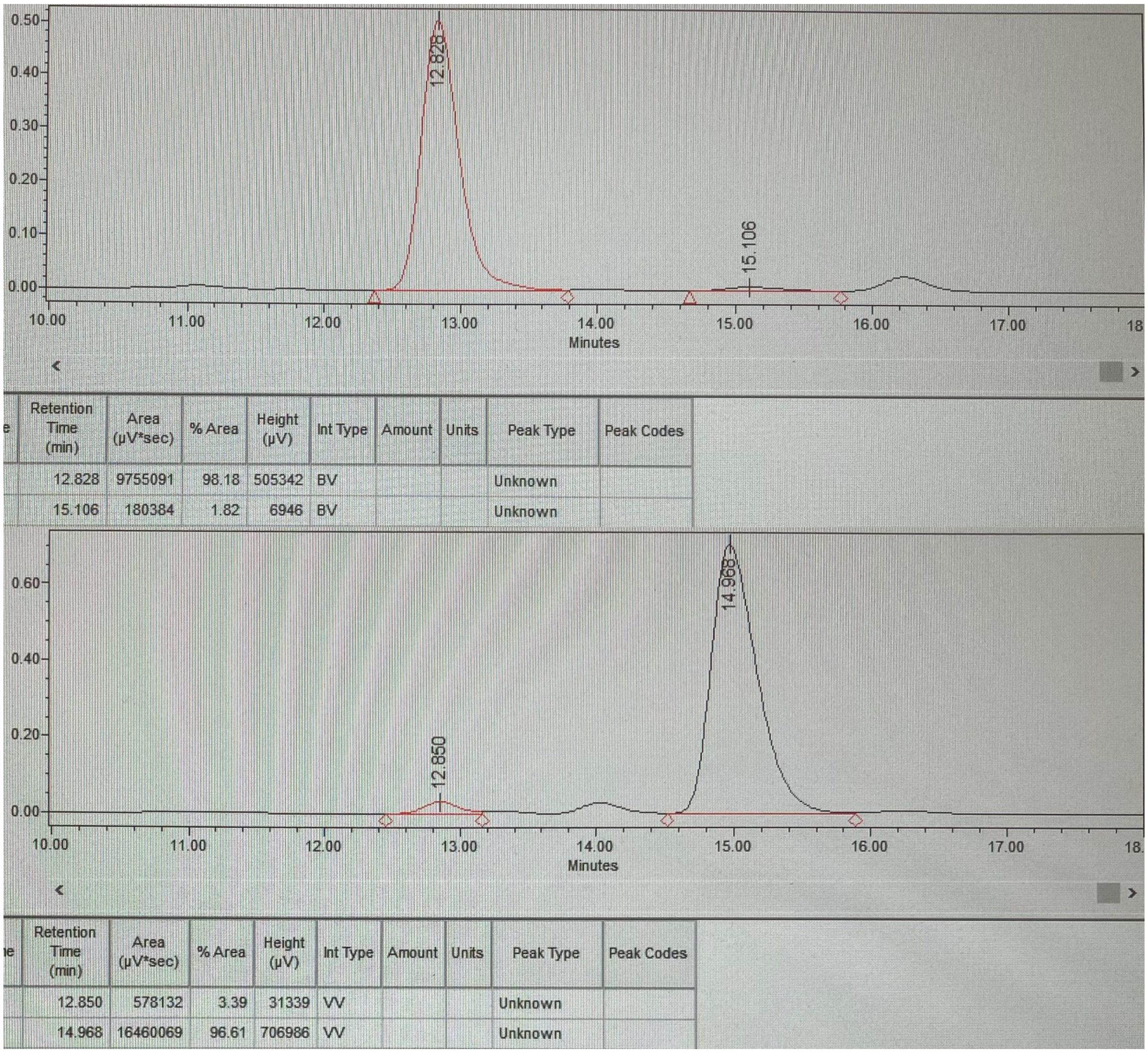

Retention Time (min)	Area (µV*sec)	% Area	Height (µV)	Int Type	Amount	Units	Peak Type	Peak Codes
12.828	9755091	98.18	505342	BV			Unknown	
15.106	180384	1.82	6946	BV			Unknown	

Retention Time (min)	Area (µV*sec)	% Area	Height (µV)	Int Type	Amount	Units	Peak Type	Peak Codes
12.850	578132	3.39	31339	VV			Unknown	
14.968	16460069	96.61	706986	VV			Unknown	

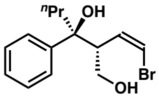

Enantiomeric excess was determined by HPLC analysis to be 97% ee (254 nm, 25 ˚C); t_1 = 11.6 min, t_2 = 13.1 min [(Chiralpak IA) hexane/i-PrOH, 95: 5, 1.0 mL/min].

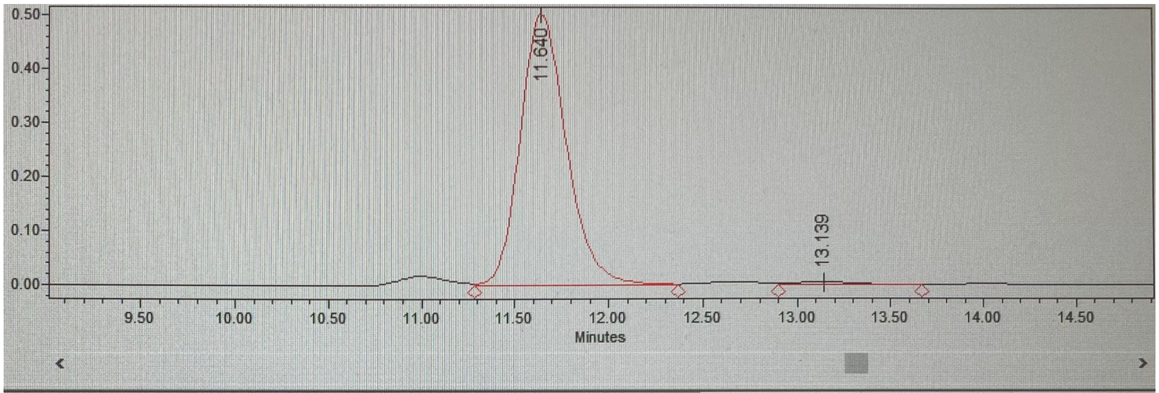

ie	Retention Time (min)	Area (µV*sec)	% Area	Height (µV)	Int Type	Amount	Units	Peak Type	Peak Codes
	11.640	8672788	98.73	504860	VV			Unknown	
	13.139	111182	1.27	4966	VV			Unknown	

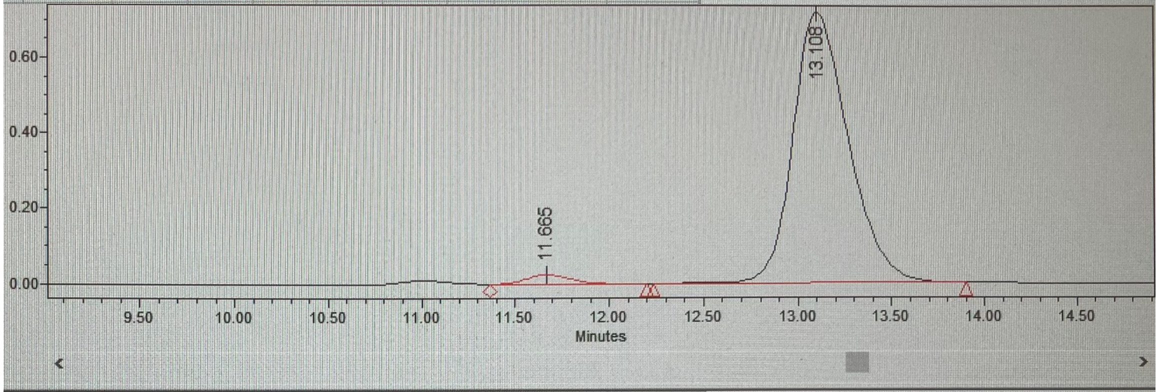

ie	Retention Time (min)	Area (µV*sec)	% Area	Height (µV)	Int Type	Amount	Units	Peak Type	Peak Codes
	11.665	428642	2.80	25831	VB			Unknown	
	13.108	14903651	97.20	723981	BB			Unknown	

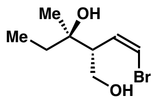

Enantiomeric excess was determined by HPLC analysis to be 99% ee (254 nm, 25 ˚C); t_1 = 8.99 min, t_2 = 15.2 min [(Chiralpak ID) hexane/i-PrOH, 95:5, 1.0 mL/min].

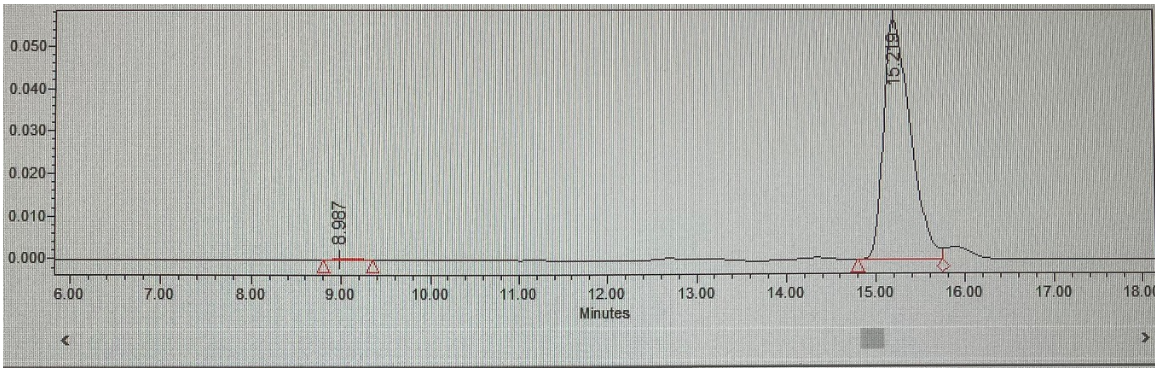

e	Retention Time (min)	Area (µV*sec)	% Area	Height (µV)	Int Type	Amount	Units	Peak Type	Peak Codes
	8.987	6072	0.51	363	bb			Unknown	
	15.219	1187668	99.49	56692	BV			Unknown	

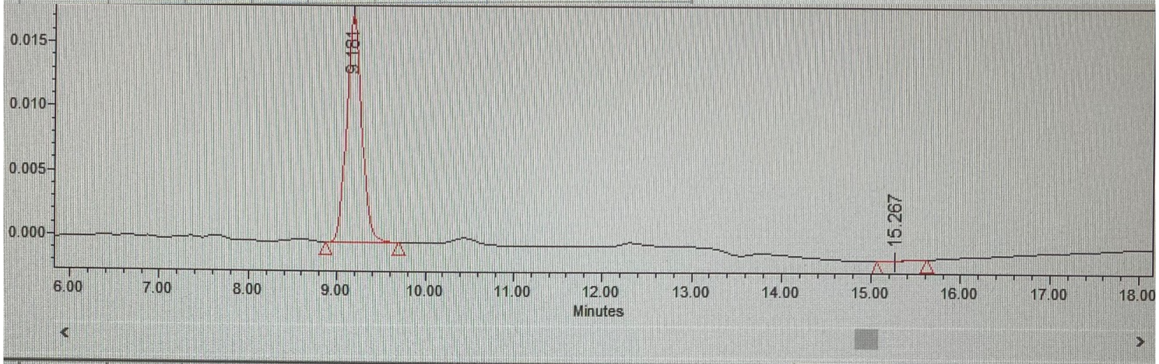

e	Retention Time (min)	Area (µV*sec)	% Area	Height (µV)	Int Type	Amount	Units	Peak Type	Peak Codes
	9.181	210243	99.76	17602	bb			Unknown	
	15.267	497	0.24	-29	bb			Unknown	I08

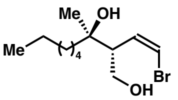

Enantiomeric excess was determined by HPLC analysis to be > 99% ee (254 nm, 25 ˚C); t_1 = 6.62 min, t_2 = 9.30 min [(Chiralpak ID) hexane/i-PrOH, 95:5, 1.0 mL/min].

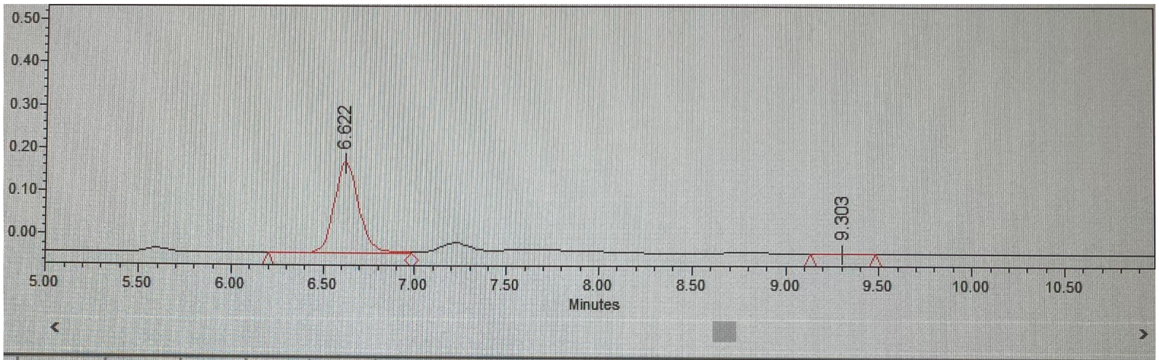

Retention Time (min)	Area (µV*sec)	% Area	Height (µV)	Int Type	Amount	Units	Peak Type	Peak Codes
6.622	1969137	99.92	211231	BV			Unknown	
9.303	1629	0.08	154	bb			Unknown	

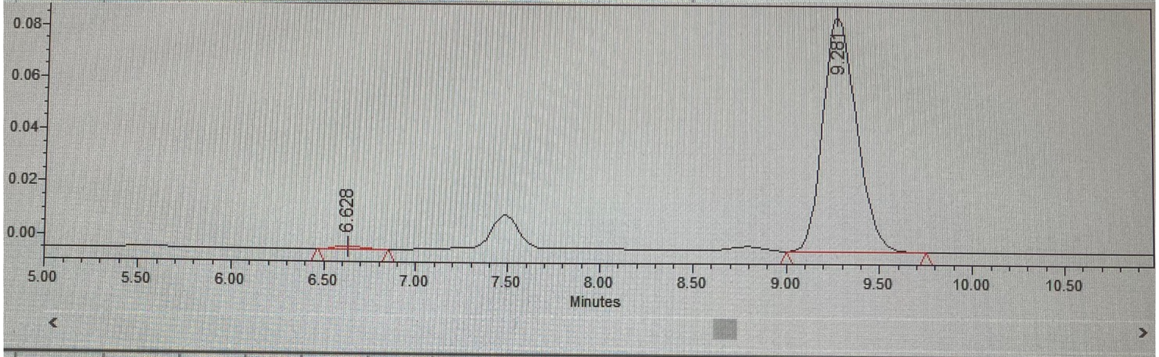

Retention Time (min)	Area (µV*sec)	% Area	Height (µV)	Int Type	Amount	Units	Peak Type	Peak Codes
6.628	9778	0.86	1145	BB			Unknown	
9.281	1126994	99.14	89384	BB			Unknown	

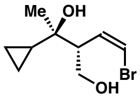

Enantiomeric excess was determined by HPLC analysis to be > 99% ee (254 nm, 25 ˚C); t_1 = 6.02 min, t_2 = 7.75 min [(Chiralpak ID) hexane/i-PrOH, 90:10, 1.0 mL/min].

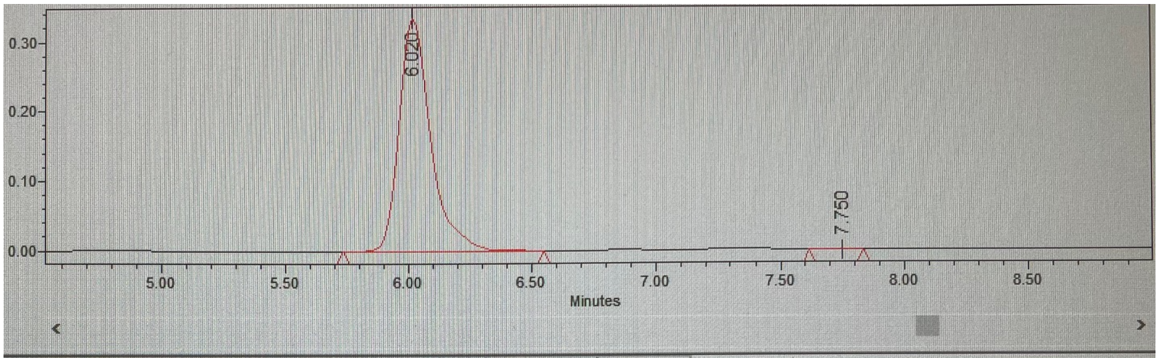

Retention Time (min)	Area (µV*sec)	% Area	Height (µV)	Int Type	Amount	Units	Peak Type	Peak Codes
6.020	2953697	99.93	333548	BB			Unknown	
7.750	2215	0.07	337	Bb			Unknown	I08

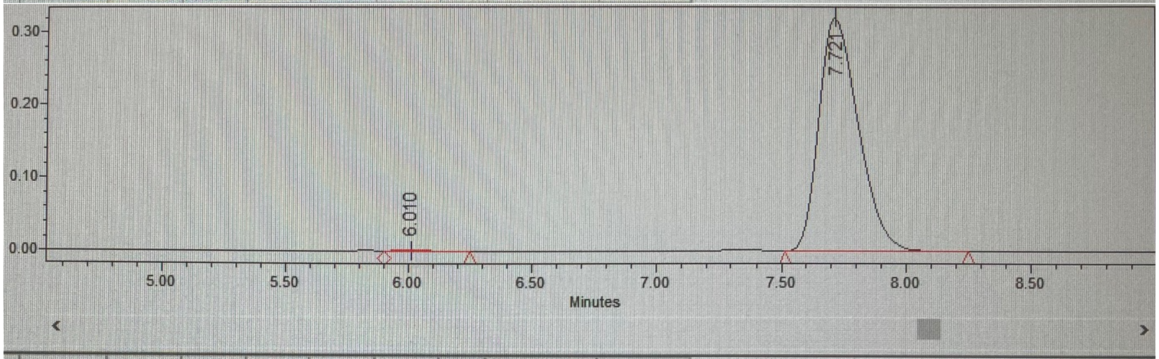

Retention Time (min)	Area (µV*sec)	% Area	Height (µV)	Int Type	Amount	Units	Peak Type	Peak Codes
6.010	21728	0.62	2189	VB			Unknown	
7.721	3504332	99.38	320450	BB			Unknown	

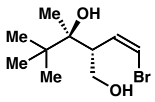

Enantiomeric excess was determined by HPLC analysis to be > 99% ee (254 nm, 25 ˚C); t_1 = 5.52 min, t_2 = 6.25 min [(Chiralpak IA) hexane/i-PrOH, 90:10, 1.0 mL/min].

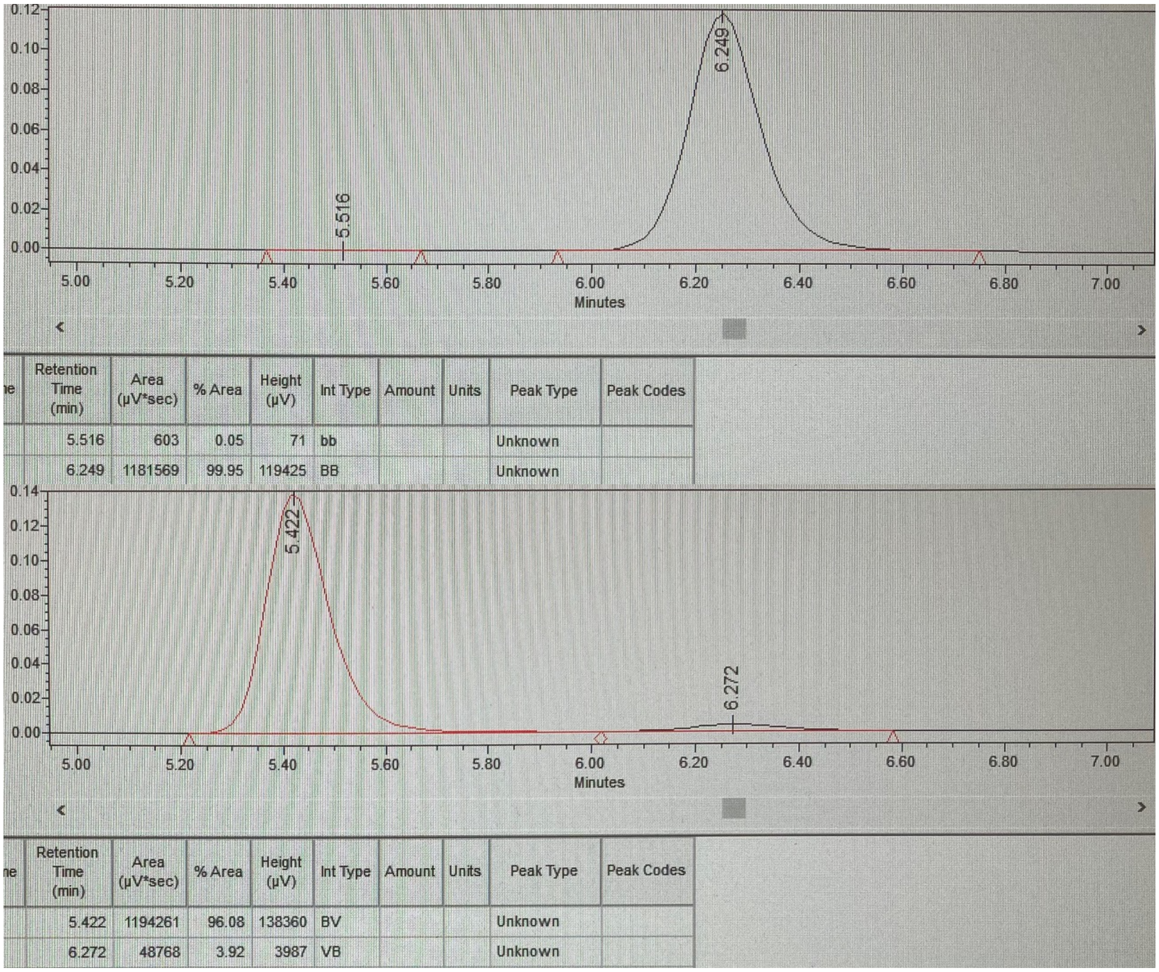

Supplement: Supplementary file 10 [file ja5c06735_si_010.pdf]
